# New Hydrazinothiazole Derivatives of Usnic Acid as Potent Tdp1 Inhibitors

**DOI:** 10.3390/molecules24203711

**Published:** 2019-10-15

**Authors:** Aleksander S. Filimonov, Arina A. Chepanova, Olga A. Luzina, Alexandra L. Zakharenko, Olga D. Zakharova, Ekaterina S. Ilina, Nadezhda S. Dyrkheeva, Maxim S. Kuprushkin, Anton V. Kolotaev, Derenik S. Khachatryan, Jinal Patel, Ivanhoe K.H. Leung, Raina Chand, Daniel M. Ayine-Tora, Johannes Reynisson, Konstantin P. Volcho, Nariman F. Salakhutdinov, Olga I. Lavrik

**Affiliations:** 1 N. N. Vorozhtsov Novosibirsk Institute of Organic Chemistry, Siberian Branch of the Russian Academy of Sciences, 9, Akademika Lavrentieva Ave., 630090 Novosibirsk, Russia; alfil@nioch.nsc.ru (A.S.F.); luzina@nioch.nsc.ru (O.A.L.); anvar@nioch.nsc.ru (N.F.S.); 2 Novosibirsk Institute of Chemical Biology and Fundamental Medicine, Siberian Branch of the Russian Academy of Sciences, 8, Akademika Lavrentieva Ave., 630090 Novosibirsk, Russia; arinachepanova@mail.ru (A.A.C.); sashaz@niboch.nsc.ru (A.L.Z.); garonna3@mail.ru (O.D.Z.); katya.plekhanova@gmail.com (E.S.I.); elpida80@mail.ru (N.S.D.); kuprummax@gmail.com (M.S.K.); 3 Novosibirsk State University, Pirogova str. 1, 630090 Novosibirsk, Russia; 4 The Federal State Unitary Enterprise, Institute of Chemical Reagents and High Purity Chemical Substances of National Research Centre, Kurchatov Institute, 107076 Moscow, Russia; kolotaev2005@rambler.ru (A.V.K.); derenik-s@yandex.ru (D.S.K.); 5 School of Chemical Sciences, The University of Auckland, Auckland 1142, New Zealand; jpat649@aucklanduni.ac.nz (J.P.); rcha387@aucklanduni.ac.nz (R.C.); dayi479@aucklanduni.ac.nz (D.M.A.-T.); 6 School of Pharmacy and Bioengineering, Keele University, Hornbeam Building, Staffordshire ST5 5BG, UK; j.reynisson@keele.ac.uk

**Keywords:** tyrosyl-DNA phosphodiesterase 1 (Tdp1), topotecan, topoisomerase 1, usnic acid, molecular modeling, synergetic effect, inhibiting activity

## Abstract

Tyrosyl-DNA phosphodiesterase 1 (Tdp1) is a promising therapeutic target in cancer therapy. Combination chemotherapy using Tdp1 inhibitors as a component can potentially improve therapeutic response to many chemotherapeutic regimes. A new set of usnic acid derivatives with hydrazonothiazole pharmacophore moieties were synthesized and evaluated as Tdp1 inhibitors. Most of these compounds were found to be potent inhibitors with IC_50_ values in the low nanomolar range. The activity of the compounds was verified by binding experiments and supported by molecular modeling. The ability of the most effective inhibitors, used at non-toxic concentrations, to sensitize tumors to the anticancer drug topotecan was also demonstrated. The order of administration of the inhibitor and topotecan on their synergistic effect was studied, suggesting that prior or simultaneous introduction of the inhibitor with topotecan is the most effective.

## 1. Introduction

The use of DNA repair enzyme inhibitors as an adjuvant therapy to treat oncological disorders is one of the breakthroughs in modern cancer therapies [1,2]. Inhibition of DNA repair enzymes increases the effectiveness of chemotherapy drugs, especially for drug-resistant tumors. Tyrosyl-DNA phosphodiesterase 1 (Tdp1) is one of the most important DNA repair enzymes that plays a key role in removing DNA damage caused by clinically-used antitumor drugs. Tdp1 belongs to the phospholipase D superfamily [3] and repairs DNA damage at the 3’-end, in particular, it hydrolyzes topomerase-cleavage complexes [4] that are formed by the action of topoisomerase 1 (Top1) inhibitors such as camptothecin and its derivatives topotecan and irinotecan.

**Figure 1:**
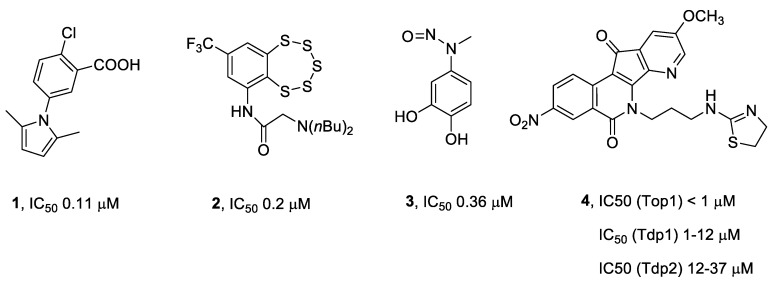
Structure of some of the most potent synthetic Tdp1 inhibitors that have been reported to date.

Several potent small molecule inhibitors of Tdp1 that inhibit the enzyme at micro- and submicromolar levels have been reported [5,6,7]. These Tdp1 inhibitors include compounds of different structural classes from synthetic, natural product, and semi-synthetic origins [5,6,7]. Amongst the most potent of the synthetic Tdp1 inhibitors are a pyrrole derivative **1** [8], benzopentathiepine **2** [9], and methyl-3,4-dephostatin **3** [10] (Figure 1). Pan-DNA repair enzyme inhibitors have also been developed. For example, compound **4** (7-azaisoquinoline derivative) inhibits three DNA repair enzymes (Top1, Tdp1, and Tdp2) [11]. 

**Figure 2:**
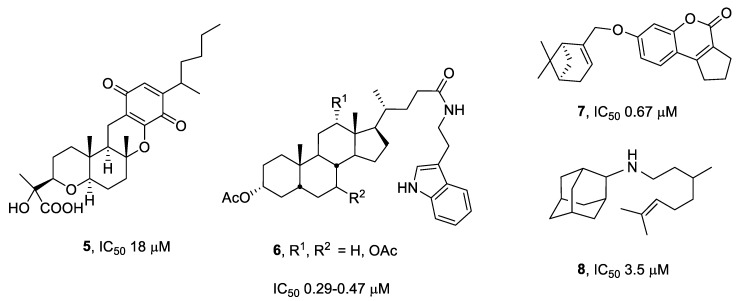
Structure of Tdp1 inhibitors that originated from natural sources and their derivatives.

In addition, Tdp1 inhibitors have also been found from natural sources. It has been reported that natural products that are isolated from anamorphic fungus (e.g., compound **5**, Figure 2) [12], triptamine derivatives of bile acids **6** [13], and monoterpenoid derivatives such as **7** and **8** that are attached to 7-hydroxycoumarin [14] or adamantane fragments [15,16,17] all show inhibitory activity against Tdp1 in the micromolar and submicromolar concentration ranges.

**Figure 3:**
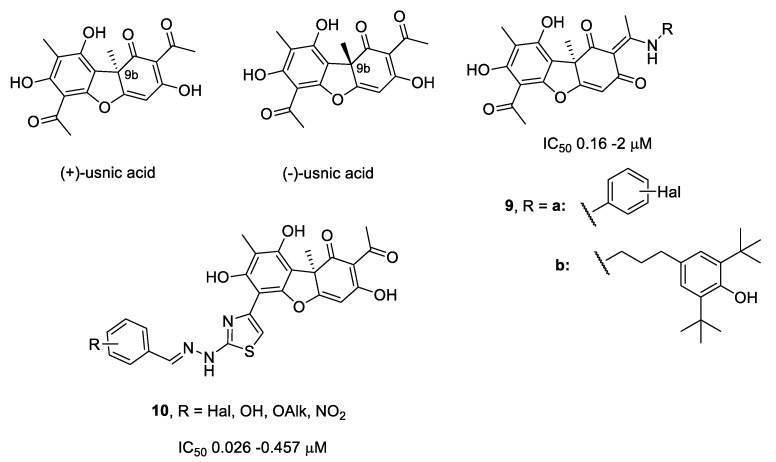
Structures of usnic acid and its derivatives that inhibit Tdp1.

Our previous works [18,19,20,21] revealed that derivatives of usnic acid inhibit Tdp1 in the micro- to nanomolar concentration ranges (Figure 3). Usnic acid (2,6-diacetyl-7,9-dihydroxy-8,9b-dimethyl-1,3(2*H*,9b*H*)-dibenzo-furandione) is a lichen metabolite and exists in two enantiomeric forms, depending on the position of the methyl group at the chiral atom C_9b_. The biological activity of both enantiomers is often very different [22,23,24,25]. Usnic acid is of interest as a platform for creating new pharmacological agents for several reasons. First, usnic acid has a broad spectrum of intrinsic biological properties. Second, it has a unique structure with a dibenzofuran backbone containing a large number of different functional groups available for modification. Third, usnic acid is widespread in different types of lichens. Finally, the procedure for the isolation of usnic acid from raw materials is simple, and the optical purity of the extracted compound is often very high. Usnic acid refers to substances of class III toxicity (moderately toxic) with an LD_50_ in mice of 838 mg/kg (Registry of Toxic Effects of Chemical Substances) and cytotoxicity in range of 10–100 µM [23]. Numerous studies indicate a significant decrease in the cytotoxicity of compounds (3–10 times) during the transition from the parent usnic acid to its derivatives [22,23,24].

For enaminic derivatives of usnic acid **9a**,**b**, they were found to inhibit Tdp1 at concentrations of 0.16–2 µM. In vitro experiments with cell cultures confirmed that they show a synergistic effect with Top1 inhibitors, leading to an order of a magnitude increase in the cytotoxic effect of the antitumor drug topotecan [18]. In vivo experiments have shown that Tdp1 inhibitor **9b** reduces metastasis growth in Lewis lung carcinoma models [20]. Derivatives of usnic acid containing arylidenehydrazinothiazole substituent (**10**) are also very potent inhibitors of Tdp1 (IC_50_ values of 0.026–0.457 µM) [19]. The acute toxicity of compounds of the class of hydrazinothiazole usnic acid derivatives was previously assessed by us in [19], where numerous animal experiments confirmed not only the safety of the doses of the inhibitor used in anticancer therapy, but also the absence of any increase in the general toxic effect of topotecan in mice. It is also known that compound **10** (R = Br, Figure 3) has a LD_50_ of more than 5000 mg/kg [19]. The addition of *para*-bromobenzylidene substituent in the thiazole cycle (R = 4-Br) enhanced the cytotoxic effect of topotecan during the combined action on the MCF-7 cell line. 

Studies on a purified enzyme showed that the structure of the aryl substituent in hydrazinothiazole derivatives of usnic acid significantly affects the inhibition potency of the compounds [19]. This study was aimed at the synthesis of new hydrazinothiazole derivatives of usnic acid and their activity. As our previous work using computer simulation [19] showed that the arylidenehydrazinothiazole fragment entered the hydrophilic pocket of the enzyme, we therefore reasoned that the replacement of the arylidene fragment with heteroarylidene and the introduction of extended substituents would enhance their efficacy. The presence of a heteroatom in the aromatic system can contribute to greater affinity with the hydrophilic pocket, and the introduction of additional substituents through the linker with heteroatoms can also increase the binding specificity.

In order to identify the structure–activity relationship, the synthesis and screening of new hydrazinothiazole derivatives of usnic acid with varying substituent structures in the hydrazone moiety of the molecule was conducted. The heterocycle size, type of heteroatom, position, and size of various substituents were chosen as the variable parameters. In addition, pairs of enantiomers based on natural (+) - and (−) usnic acids were synthesized to study the effect of the chiral center in the dibenzofuran core for these compounds for the first time. 

## 2. Results and Discussion

### 2.1. Chemistry

Compounds **16a**–**r** and **17a**–**k** were synthesized by a method similar to that developed previously [19]. 

**Scheme 1:**
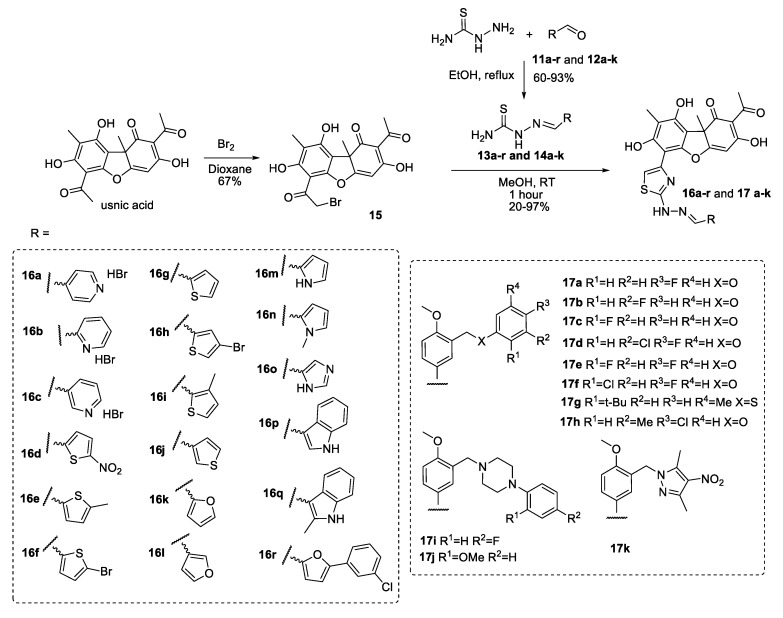
Synthesis of compounds **16a–r** and **17a–k**.

Thiosemicarbazones **13a**–**g** and **13i**–**q** were obtained using a known procedure [26]. Thiosemicarbazones **13h,r** and **14a**–**k** were obtained using the reaction of aldehydes (**11h,r** and **12a**–**k**) with thiosemicarbazide in ethanol (Scheme 1). The precipitate formed was filtered off, washed with water, and then air dried. Thiosemicarbazones **13h,r** and **14a**–**k** were obtained with yields ranging from 60% to 93%. 

*R*-(+)-usnic acid ((+)**-1**) and *S*-(−)-usnic acid ((−)-**1**) were obtained by extraction from a mixture of the lichen genus *Usnea* and *Cladonia stellaris* and used as the starting material [27]. The synthesis of the bromo-substituted derivative **15** was performed by a reaction with bromine in dioxane [28]. New derivatives of usnic acid hydrazonothiazoles **16a**–**r** and **17a**–**k** were synthesized by the reaction of the bromo-substituted derivative **15** with thiosemicarbazones **13a**–**r** and **14a**–**k** in methanol. The precipitate was filtered off, dissolved in methylene chloride, washed with sodium bicarbonate solution, and the organic phase was evaporated (Scheme 1). Thus, usnic acid derivatives **16a**–**r** and **17a**–**k** were obtained with yields from 20% to 97%. 

### 2.2. Biology

#### 2.2.1. Structure–Activity Relationship Analysis

We first studied the inhibition potency of our compounds to Tdp1 by using a real-time oligonucleotide biosensor assay that we have previously developed [9]. The assay was based on the ability of Tdp1 to remove the fluorescence quencher from the 3’-end of DNA. The substrate is a 16-mer single-stranded oligonucleotide containing both a 5’-FAM fluorophore and a 3’-BHQ1 quencher. The testing results are presented in Table 1 and Table 2.

An analysis of the data in Table 1 shows that although the replacement of the arylidene fragment with heteroarylidene did not lead to a significant increase in activity, most of the synthesized new compounds were highly active Tdp1 inhibitors, with active concentrations between 20–200 nM, with the exception of **16b**, **16c**, and **16o**. The size of the heterocyclic fragment and the nature of the heteroatom did not significantly affect the inhibitory activity. The formation of hydrobromide in the case of compounds **16a**–**c** and **16o** led to a decrease in inhibitory activity. The presence of a bromine atom (**16f**,**h**) or bulky aromatic fragment as a substituent in the heterocycle (**16r**) led to an increase in inhibitory activity. The most active compounds from this set contained pyrrole (**16m**) or the *N*-methylpyrrole substituent (**16n**) in the hydrazone fragment of the (−)-enantiomer. A comparison of the data on the inhibitory activity of pairs of enantiomers did not allow us to reliably judge the role of the configuration of the optical center in the binding of the compound to the enzyme. For seven pairs of compounds, the inhibitory concentration was comparable (it was considered comparable if the concentration of one of the pairs was no more than two times the concentration of the other). For five pairs of compounds, a higher activity was observed for the (+)-enantiomers, while in other cases, (−)-enantiomers were more active. 

The following series of compounds was synthesized from benzaldehydes with an extended substituent. 

In general, we can say that the increase in the size of the substituent led to very good inhibitory activity in the concentration range of 18–122 nM. The presence of a halogen atom as a substituent in the terminal aromatic ring, as a rule, increases the inhibitory activity of the compounds. At the same time, the location and nature of the halogen atom as well as the number of halogen atoms in the terminal aromatic ring did not significantly affect the level of inhibitory activity. Replacing the O-/S- methylene linker (**17a**–**h**) with a piperazinemethylene linker (**17i**, **17j**) also did not have significant effect. As the configuration of the optical center of the core of usnic acid is not of fundamental importance, we can only note a slight tendency to increase the inhibitory activity of (−)-enantiomers for compounds containing a chlorine atom in the terminal aromatic ring (**17d**,**f**,**h**).

The binding of the synthesized compounds to recombinant Tdp1 was also tested (Table 1 and Table 2) by using an intrinsic tryptophan fluorescence quenching assay [29]. All of the tested compounds were found to bind to Tdp1, confirming the inhibition assays that the compounds are targeting Tdp1. Interestingly, the binding constant values (*K*_D_) of the compounds were all found to be in the mid-μM region. The discrepancy between the binding and inhibition assays may be due to the large substrate binding site that Tdp1 possesses, which contains both a DNA binding pocket and a peptide binding pocket as well as the active site. As our compounds are aromatic in nature, there is a possibility that they may bind to both the active site as well as non-specifically to the DNA/peptide binding pockets. Nonetheless, our results confirmed that the compounds were indeed binders of Tdp1, thus complementing our inhibition data.

#### 2.2.2. Synergistic Effect

Top1 poisons are used as anticancer drugs for the treatment of a wide range of oncological diseases [30,31]. Since Tdp1 is involved in the removal of DNA damage caused by Top1 poisons, it is believed that Tdp1 is responsible for the drug resistance of some cancers [32]. Thus, a combination of Top1 poisons and Tdp1 inhibitors could improve the effectiveness of chemotherapy.

**Figure 4:**
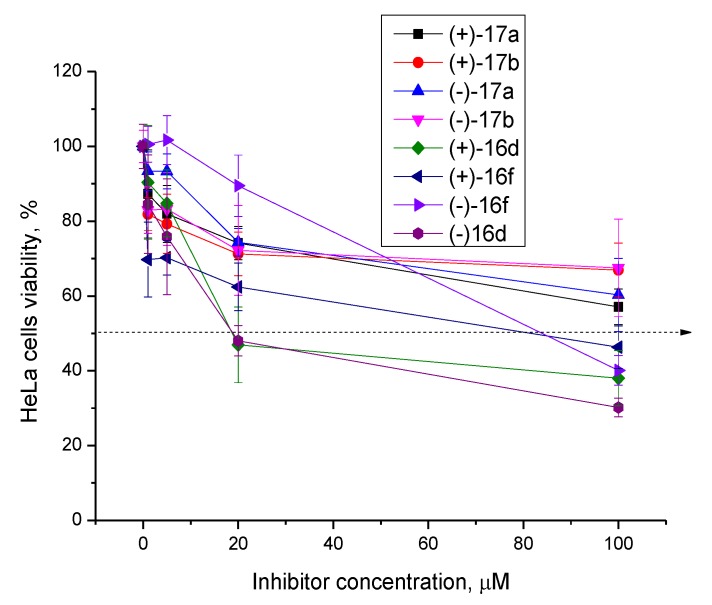
The cytotoxicity of the hydrazinothiazole derivatives according to the MTT test.

First, we studied the intrinsic cytotoxicity of the compounds against HeLa cells (cervical carcinoma). For experiments on cells, we selected compounds which combined high inhibitory activity with good chemical accessibility. The MTT test data are shown in Figure 4 for compounds from both the heteroarylidene-hydrazinotiazole and arylidene-hydrazinotiazole subclasses. The arylidene-hydrazinothiazole usnic acid derivatives have low toxicity (Figure 4). Heteroarylidene-hydrazinothiazole derivatives (+)- and (−)-**16f**, containing a bromothiophene residue, also have low toxicity (CC_50_ about 80 µM), while the **16d** pair, containing a nitro group that is a known toxicophore [33], suppressed cell growth at a concentration of 20 µM. Based on these data, a 5 µM concentration of Tdp1 inhibitors was chosen for the next experiment.

Since the ligands are intended for an adjuvant therapy, their own low toxicity and the absence of additional side effects are essential. Thus, with the exception of a few compounds, usnic acid hydrazinotiazole derivatives have low cytotoxicity and are suitable for further research.

**Figure 5:**
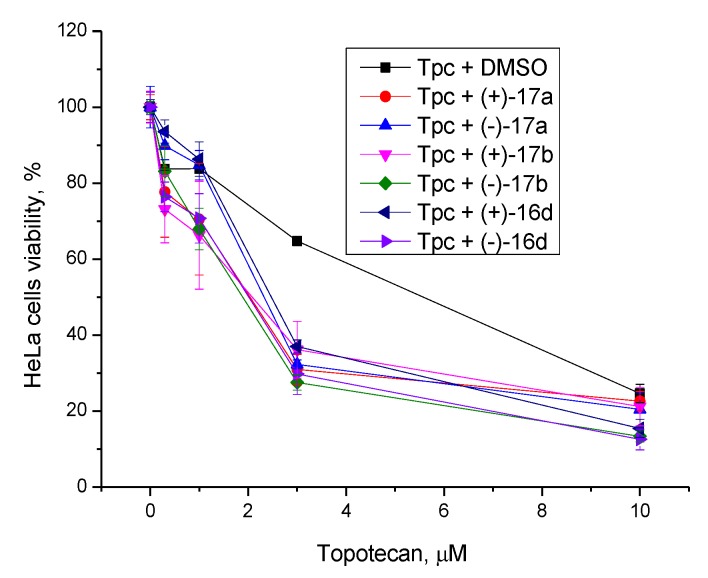
Dose-dependent action of topotecan (Tpc) in combination with usnic acid derivatives on HeLa cell viability using the MTT method.

Next, we studied the effect of Tdp1 inhibitors on the cytotoxicity effect of topotecan, the Top1 poison used in clinical settings. We used non-toxic concentrations of usnic acid derivatives (5 µM) and different concentrations of topotecan. The Tdp1 inhibitors increased the cytotoxic effect of topotecan approximately two to threefold (Figure 5) and the CC_50_ values decreased from 6 μM to 2–3 μM. The sensitizing effect of the compounds did not depend on the structure of the substituent in the hydrazinothiazole moiety.

**Figure 6:**
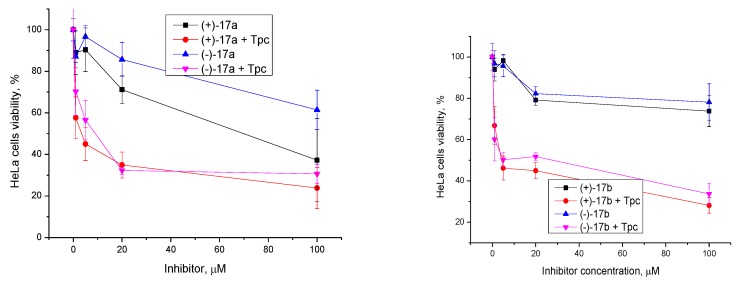
Dose-dependent action of usnic acid derivatives in combination with topotecan (Tpc) on HeLa cell viability using the MTT method.

In order to derive the optimal concentration for the inhibitors to provide the maximum sensitizing effect, but remaining non-toxic, the concentrations with topotecan at 2 µM, were varied. Topotecan increased the cytotoxicity of the ligands dramatically from >70 µM to 5–10 µM (Figure 6). 

We found the optimal compound concentrations of 20 µM. With topotecan at 2 µM, a pronounced cytotoxic effect was observed at this concentration for the ligands, while in the absence of topotecan, such concentrations were almost non-toxic. 

**Figure 7:**
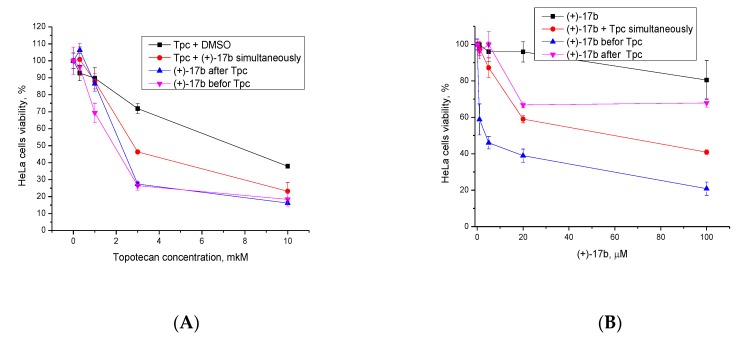
Dose-dependent action of (**A**) 2 µM topotecan in combination with 20 µM (+)-**17b** and (**B**) 20 µM (+)-**17b** in combination with 2 µM topotecan (Tpc) on HeLa cell viability using the MTT method.

Since there is no information on the rate and efficiency of the penetration of compounds into the cells, we studied the effect of the order of administration of (+)-**17b** and topotecan on their synergistic effect. Figure 7A shows the dependence of cell survival on topotecan concentration at 20 μM of (+)-**17b**: topotecan and the Tdp1 inhibitor were applied to the wells simultaneously, pre-mixed (red graph); first topotecan, then after 30 minutes (+)-**17b** (blue graph); first (+)-**17b**, after 30 minutes topotecan (pink graph). The data showed that the simultaneous administration of drugs reduced the synergistic effect and that the sequence of separately administrating the drugs did not have an effect. 

When varying the concentration of (+)-**17b** with 2 µM of topotecan, the administration of (+)-**17b** after topotecan (pink graph, Figure 7B) led to worse results when compared with simultaneous administration (red graph) and even more so when compared with the administration first with the Tdp1 inhibitor, and then the Top1 inhibitor (blue graph).

It is likely that a more pronounced effect occurs when first suppressing the ability of cells for repair, and subsequently causes damage. Apparently, the administration of topotecan first gives cells time to repair part of the DNA damage. 

### 2.3. Molecular Modeling

The fifty-eight molecules (**16a**–**r**, **17a**–**k**) were docked into the binding site of Tdp1 (PDB ID: 6DIE, resolution 1.78 Å) [34] both with and without water molecules (see further details in the Methodology section). The co-crystallized ligand benzene-1, 2, 4-tricarboxylic acid was removed, re-docked, and compared with the co-crystallized structure. The Astex statistical potential (ASP) scoring function obtained an average root-mean-square deviation (RMSD) of 2.896 Å, improved piecewise linear potential (ChemPLP) of 2.818 Å, a ChemScore (CS) of 0.613 Å, and a GoldScore (GS) of 9.596 Å when docked with the crystalline water molecules. Meanwhile, ASP obtained an average RMSD of 2.868 Å, ChemPLP of 4.422 Å, CS of 4.557 Å, and GS of 4.024 Å when docked without the water molecules (see Table S17 in the Supplementary Materials). Docking with the water molecules obtained higher scores than without water (see Table S18 in the Supplementary Materials). Better results were obtained when keeping the crystalline water molecules in the docking scaffold. The new 6DIE holo-crystal structure now allows for more robust modeling of TDP1 inhibitors when compared to prior apo-structures. When the scores, with and without the crystalline water, were correlated to both the IC_50_ and K_D_ values, a weak trend was seen with R^2^ values in the range of ~0.1. However, in all cases, the slope of the fitted line had a negative value (i.e., higher scores resulted in ligands that are more potent in general).

**Figure 8:**
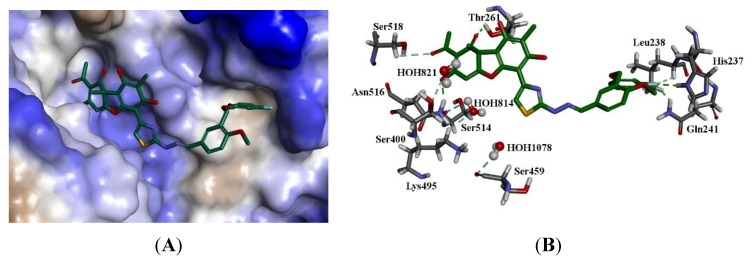
The docked configuration of (+)-**17b** in the binding site of Tdp1 as predicted using the ChemScore scoring function. (**A**) The protein surface is rendered. The ligand occupies the binding pocket. Blue depicts a hydrophilic region on the surface; brown depicts hydrophobic region and whites shows neutral areas. (**B**) Hydrogen bonds are shown as green lines between Thr261, Asn516. The fluoride moiety interacts with Gln241 and His237. The water molecules also form hydrogen bonds with Ser459, Ser514, and Lys495.

The modeling showed that all of the ligands had a plausible binding mode. Considering (+)-**17b**, the most active molecule, the dibenzofuran and the thiazole moieties occupy the hydrophilic binding region, which contains amino acids such as glutamic acid and threonine, whilst the anisole and the fluorobenzene group occupy the hydrophobic region with amino acids such as isoleucine, leucine, and phenylalanine. The carbonyl on the dibenzofuran moiety forms a hydrogen bond with the side chain hydroxyl group of threonine (Thr261). A phenyl group on this moiety also forms a hydrogen bond with the side chain amine group of asparagine (Asn516). The fluoride group of this molecule also interacts with the side chain amine of glutamine (Gln241) and the imidazole group of histidine (His237). The imidazole groups of the catalytic histidine amino acid residues His263 and His 493 are blocked by the ligands, explaining the effective inhibition of Tdp1 [10,13,14,15,35]. The co-crystalized ligand benzene-1,2,4-tricarboxylic acid forms hydrogen bonds with His493 and blocks access to His263, explaining its efficacy. It also forms hydrogen bonds with Lys265, Ser399, and Lys495, which were not seen for (+)-**17b.** This further strengthens the argument that blocking access to the His263/493 catalytic pair is paramount in developing effective Tdp1 inhibitors. The binding of (+)-**17b** is shown in Figure 8. Interestingly, very similar scores were predicted for both the enantiomers, reflecting the findings from the activity and affinity experiments (i.e., the binding pocket can accommodate both forms). 

### 2.4. Chemical Space

In order to check the compatibility of the ligands with biological systems, their physicochemical parameters were derived. The calculated molecular descriptors (MW (molecular weight), log *P* (water–octanol partition coefficient), HD (hydrogen bond donors), HA (hydrogen bond acceptors), PSA (polar surface area), and RB (rotatable bonds)) are given in Table S19 in the Supplementary Materials. MW, HA, PSA, and RB were within the drug-like and known drug space. The log *P* values ranged from lead-like to just beyond the known drug space, while HD was within the lead-like space. The lead-like chemical space is defined as relatively small parameters based on lead compounds, which can be developed further and gain both weight and lipophilicity. The drug-like chemical space is based on orally bioavailable drug candidates, and finally, the known drug space (KDS) encompasses all small molecules in clinical use (for further discussion and use of these regions in chemical space see [36] and Table S20). The molecular weight of the ligands was between 492.5 and 752.8 g mol^−1^ and the log *P* values were in the range of 1.5 and 7.1, which was just beyond the KDS region. The ligands had PSA values between 132.5 and 198.3, reaching beyond the KDS. Therefore, it can be concluded that the ligands were relatively large and upon further development, could not be made much larger.

To gauge the balance of the molecular descriptors of the ligands and therefore their compatibility with biological systems, the known drug index (KDI) was calculated. This method is based on the statistical analysis of drugs in clinical use (KDS) and the weighted indexes for each of the six descriptors used. Both the summation (KDI_2a_) and multiplication (KDI_2b_) methods were used [37]. KDI_2a_ is more forgiving than its KDI_2a_ counterpart, since a poor index for one parameter can be compensated for, but when multiplied will result in a poor score as the multiplication of small numbers will lead to small numbers. The KDI_2a_ values ranged from 2.4 to 5.5 with a theoretical maximum of 6 and the average of 4.08 for known drugs. KDI_2b_ ranged from 0.0 to 0.57, with a theoretical maximum of 1 and with a KDS average of 0.18. This indicates that some of the ligands were relatively well balanced. The most potent ligand (+)-**17b** had a KDI_2a_ of 3.41 and KDI_2b_ of 0.01; the balance of the molecular descriptors is relatively good compared to known drugs with the exception of MW (657.7 g mol^−1^), which resulted in a very low number for KDI_2b_, but a good value for KDI_2a_.

## 3. Materials and Methods

### 3.1. Chemistry

The analytical and spectral studies were conducted at the Chemical Service Center for the collective use of Siberian Branch of the Russian Academy of Science.

The ^1^H and ^13^C-NMR spectra for solutions of the compounds in CDCl_3_ were recorded on a Bruker AV-400 spectrometer (400.13 and 100.61 MHz, respectively). The residual signals of the solvent were used as references (δ_H_ 2.48, δ_C_ 39.52 for DMSO-*d6* and δ_H_ 7.27, δ_C_ 77.1 for CDCl_3_). The mass spectra (70 eV) were recorded on a DFS Thermo Scientific high-resolution mass spectrometer. The melting points were measured using a Kofler heating stage. The specific rotation was determined on a PolAAr 3005 (Optical Activity Ltd, England) and provided in (deg × mL) × (g × dm)^–1^, whereas the concentration of the solutions is shown in g × (100 × mL)^–1^. Merck silica gel (63–200 μ) was used for the column chromatography. Thin-layer chromatography was performed on TLC Silica gel 60F_254_ (Merck KGaA, Darmstadt, Germany).

The atom numbers in the compound are provided for the assignment of signals in the NMR spectra and are different from the numeration in the nomenclature name. The target compounds reported in this manuscript had a purity of at least 99% (HPLC). All chemicals were used as described unless otherwise noted. Reagent-grade solvents were redistilled prior to use. Synthetic starting materials, reagents, and solvents were purchased from Sigma-Aldrich, Acros Organics, and AlfaAesar.

(*R*)-(+)-Usnic acid **1** (α_D_ +478 (c 0.1, CHCl_3_)) was isolated from a mixture of lichens of the *genus Usnea* through the procedure developed by Salakhutdinov et al. [27]. (*S*)-(−)-Usnic acid **1** (α_D_ −456 (c 0.1, CHCl_3_)) was isolated from *Cladonia stellaris*, by the same procedure [27].

Aldehydes **12a**–**k** were obtained using a known procedure [38,39].

Compounds (+)-**15** and (−)-**15** were obtained using a known procedure. The spectra of the substances coincided with that in the literature [28,40].

#### 3.1.1. General Procedure for the Synthesis of Compounds **13a**–**r** and **14a**–**k**

A total of 1 mM of the corresponding aldehyde **11a**–**r** and **12a**–**k** was dissolved in 2 mL of ethyl alcohol. The resulting solution was slowly added dropwise with stirring to a solution of 1 mM thiosemicarbazide in 2 mL of distilled water. The precipitate that formed was filtered off, washed with water, then air dried. The thiosemicarbazones **13a**–**r** and **14a**–**k** obtained were isolated in 58–93% yields.

*(E)-2-(Pyridin-4-ylmethylene)hydrazinecarbothioamide* (**13a**): Pale yellow solid with an 84% yield. The spectrum of the substance corresponds to the literature [26]. 

*(E)-2-(Pyridin-2-ylmethylene)hydrazinecarbothioamide* (**13b**): Straw solid with a 65% yield. The spectrum of the substance corresponds to the literature [41]

*(E)-2-(Pyridin-2-ylmethylene)hydrazinecarbothioamide* (**13c**): White solid with a 77% yield. The spectrum of the substance corresponds to the literature [42].

*(E)-2-((5-Nitrothiophen-2-yl)methylene)hydrazinecarbothioamide* (**13d**): Yellow solid with a 76% yield. The spectrum of the substance corresponds to the literature [43].

*(E)-2-((5-Methylthiophen-2-yl)methylene)hydrazinecarbothioamide* (**13e**): Yellow solid with a 92% yield. The spectrum of the substance corresponds to the literature [43].

*(E)-2-((5-Bromothiophen-2-yl)methylene)hydrazinecarbothioamide* (**13f**): Straw solid with a 76% yield. The spectrum of the substance corresponds to the literature [42].

*(E)-2-(Thiophen-2-ylmethylene)hydrazinecarbothioamide* (**13g**): Brown solid with an 89% yield. The spectrum of the substance corresponds to the literature [44].

*(E)-2-((4-Bromothiophen-2-yl)methylene)hydrazinecarbothioamide* (**13h**): Straw solid with a 93% yield. NMR ^1^H (DMSO-*d6*, δ): 1.52 (1H, s, H-3), 7.67 (1H, bs, NH2), 7.74 (1H, s, H-1), 8.16 (1H, s, H-5), 8.24 (1H, bs, NH2), 11.52 (1H, s, NH). SNMR ^13^C (DMSO-*d6*, δ): 109.33 (C-2), 125.95 (C-1), 131.55 (C-3), 135.81 (C-5), 140.08 (C-4), 177.82 (C-6).

*(E)-2-((3-Methylthiophen-2-yl)methylene)hydrazinecarbothioamide* (**13i**): Straw solid with an 84% yield. The spectrum of the substance corresponds to the literature [45].

*(E)-2-((Thiophen-3-yl)methylene)hydrazinecarbothioamide* (**13j**): Straw solid with an 81% yield. The spectrum of the substance corresponds to the literature [46].

*(E)-2-(Furan-2-ylmethylene)hydrazinecarbothioamide* (**13k**): Straw solid with a 60% yield. The spectrum of the substance corresponds to the literature [42].

*(E)-2-(Furan-3-ylmethylene)hydrazinecarbothioamide* (**13l**): Brown solid with 5a 8% yield. The spectrum of the substance corresponds to the literature [44].

*(E)-2-((1H-Pyrrol-2-yl)methylene)hydrazinecarbothioamide* (**13m**): White solid with a 74% yield. The spectrum of the substance corresponds to the literature [42].

*(E)-2-((1-Methyl-1H-pyrrol-2-yl)methylene)hydrazinecarbothioamide* (**13n**): Pale pink solid with a 63% yield. The spectrum of the substance corresponds to the literature [47].

*(E)-2-((1H-Imidazol-5-yl)methylene)hydrazinecarbothioamide* (**13o**): White solid with a 74% yield. The spectrum of the substance corresponds to the literature [48].

*(E)-2-((1H-Indol-3-yl)methylene)hydrazinecarbothioamide* (**13p**): Creamy solid with a 74% yield. The spectrum of the substance corresponds to the literature [49].

*((E)-2-((1H-2-Methylindol-3-yl)methylene)hydrazinecarbothioamide* (**13q**): Straw solid with a 74% yield. The spectrum of the substance corresponds to the literature [49].

*(E)-2-((5-(3-Chlorophenyl)furan-2-yl)methylene)hydrazinecarbothioamide* (**13r**): Yellow solid with a 90% yield. NMR ^1^H (DMSO-*d6*, δ): 7.07 (1H, d, J = 3.57 Hz, AB-system, H-9), 7.23 (1H, d, J = 3.57 Hz, AB-system, H-8), 7.37 (1H, m, H-Ar), 7.45 (1H, t, J = 7.84 Hz, H–Ar), 7.79 (1H, m, H-Ar), 7.89 (1H, t, J = 1.77 Hz, H-Ar), 7.85 (1H, bs, NH2), 7.89 (1H, m, H-2), 7.98 (1H, s, H-11), 8.29 (1H, bs, NH2), 11.53 (1H, s, NH). NMR ^13^C (DMSO-*d6*, δ): 109.66 (C-9), 115.31 (C-8), 122.49 (C-Ar), 123.41 (C-Ar), 127.80 (C-Ar), 130.80 (C-Ar), 131.45 (C-1), 131.68 (C-11), 133.86 (C-3), 149.54 (C-10), 152.83 (C-7), 177.75 (C-12).

*(E)-2-(3-((4-Fluorophenoxy)methyl)-4-methoxybenzylidene)hydrazinecarbothioamide* (**14a**): White amorphous powder with a 75% yield. NMR ^1^H (400 MHz, DMSO-*d6*): see Table 3. NMR ^13^C (100 MHz, DMSO-*d6*): see Table 4.

*(E)-2-(3-((3-Fluorophenoxy)methyl)-4-methoxybenzylidene)hydrazinecarbothioamide* (**14b**): White amorphous powder with a 71% yield. NMR ^1^H (400 MHz, DMSO-*d6*): see Table 3. NMR ^13^C (100 MHz, DMSO-*d6*): see Table 4.

*(E)-2-(3-((2-Fluorophenoxy)methyl)-4-methoxybenzylidene)hydrazinecarbothio amide* (**14c**): White amorphous powder with a 73% yield. NMR ^1^H (400 MHz, DMSO-*d6*): see Table 3. NMR ^13^C (100 MHz, DMSO-*d6*): see Table 4.

*(E)-2-(3-((3-Chloro-4-fluorophenoxy)methyl)-4-methoxybenzylidene)hydrazinecarbothio amide* (**14d**): White amorphous powder with a 60% yield. NMR ^1^H (400 MHz, DMSO-*d6*): see Table 3. NMR ^13^C (100 MHz, DMSO-*d6*): see Table 4.

*(E)-2-(3-((2,4-Difluorophenoxy)methyl)-4-methoxybenzylidene)hydrazinecarbothioamide* (**14e**): White amorphous powder with 68% yield. Spectra NMR ^1^H (400 MHz, DMSO-*d6*): Table 3. Spectra NMR ^13^C (100 MHz, DMSO-*d6*): Table 4.

*(E)-2-(3-((2-Chloro-4-fluorophenoxy)methyl)-4-methoxybenzylidene)hydrazinecarbothio amide* (**14f**): White amorphous powder with an 82% yield. NMR ^1^H (400 MHz, DMSO-*d6*): see Table 3. NMR ^13^C (100 MHz, DMSO-*d6*): see Table 4.

*(E)-2-(3-((2-tert-Butyl-5-methylphenylthio)methyl)-4-methoxybenzylidene)hydrazinecarbo thioamide* (**14g**): White amorphous powder with a 91% yield. NMR ^1^H (400 MHz, DMSO-*d6*): see Table 5. NMR ^13^C (100 MHz, DMSO-*d6*): see Table 6.

*(E)-2-(3-((4-Chloro-3-methylphenoxy)methyl)-4-methoxybenzylidene)hydrazinecarbothio amide* (**14h**): White amorphous powder with an 81% yield. NMR ^1^H (400 MHz, DMSO-*d6*): see Table 5. NMR ^13^C (100 MHz, DMSO-*d6*): see Table 6.

*(E)-2-(3-((4-(4-Fluorophenyl)piperazin-1-yl)methyl)-4-methoxybenzylidene)hydrazinecarbo thioamide* (**14i**): White amorphous powder with an 86% yield. NMR ^1^H (400 MHz, DMSO-*d6*): see Table 5. NMR ^13^C (100 MHz, DMSO-*d6*): see Table 6.

*(E)-2-(4-Methoxy-3-((4-(3-methoxyphenyl)piperazin-1-yl)methyl)benzylidene)hydrazine carbothioamide* (**14j**): White amorphous powder with a 74% yield. NMR ^1^H (400 MHz, DMSO-*d6*): see Table 5. NMR ^13^C (100 MHz, DMSO-*d6*): see Table 6.

*(E)-2-(3-((3,5-Dimethyl-4-nitro-1H-pyrazol-1-yl)methyl)-4-methoxybenzylidene)hydrazinecarbothioamide* (**14k**): White amorphous powder with a 69% yield. NMR ^1^H (400 MHz, DMSO-*d6*): see Table 5. NMR ^13^C (100 MHz, DMSO-*d6*): see Table 6.

#### 3.1.2. General Procedure for the Synthesis of Compounds **16a**–**r** and **17a**–**k**

A mixture of compound (+)-**15** or (−)-**15** (1 mmol, 423 mg) and the corresponding thiosemicarbazone **13a**–**r** or **14a**–**k** (1 mmol) were heated under reflux in 25 mL MeOH for 0.5–2 h. The reaction mixture was cooled and poured onto water (75 mL). After that, the reaction mixture was cooled, the precipitate that formed was filtered off, washed with MeOH, and dried in air. The precipitate (except for **16a**–**c**) was placed in a separatory funnel, 30 mL of methylene chloride was added thereto, a suspension was formed, and 20 mL of a saturated solution of NaHCO_3_ was added. The mixture was shaken vigorously several times until the suspension turned into a completely dark red solution. The resulting solution was separated from the aqueous layer, washed with 20 mL water once, and evaporated on a rotary evaporator. Compounds **16a**–**r** and **17a**–**k** obtained were isolated in 20–97% yields. 

*4-[(1E)-(2-{4-[(1R)-12-Acetyl-3,5,11-trihydroxy-1,4-dimethyl-13-oxo-8-oxatricyclo[7.4.0.0^2,7^]trideca-2(7),3,5,9,11-pentaen-6-yl]-1,3-thiazol-2-yl}hydrazin-1-ylidene)methyl]pyridin-1- ium bromide(+)-16a*: Orange amorphous powder with a 74% yield. M.p.: 191–193 °C dec. NMR ^1^H (400 MHz, DMSO-*d6*): see Table 7. NMR ^13^C (100 MHz, DMSO-*d6*): see Table 8. HRMS: *m/z* 504.1094 [M − HBr]^+^ (calcd. for (C_25_H_20_O_6_N_4_^32^S_1_)^+^: 504.1098). Anal. Calcd. for C_25_H_21_BrO_6_N_4_S_1_ (585.42): Br, 13.65%. Found: Br, 13.58%. 

*4-[(1E)-(2-{4-[(1S)-12-Acetyl-3,5,11-trihydroxy-1,4-dimethyl-13-oxo-8-oxatricyclo[7.4.0.0^2,7^] trideca-2(7),3,5,9,11-pentaen-6-yl]-1,3-thiazol-2-yl}hydrazin-1-ylidene)methyl]pyridin-1- ium bromide(−)-16a*: Orange amorphous powder with a 77% yield. 

*2-[(1E)-(2-{4-[(1R)-12-Acetyl-3,5,11-trihydroxy-1,4-dimethyl-13-oxo-8-oxatricyclo[7.4.0.0^2,7^] trideca-2(7),3,5,9,11-pentaen-6-yl]-1,3-thiazol-2-yl}hydrazin-1-ylidene)methyl]pyridin-1- ium bromide(+)-16b*: Orange amorphous powder with a 62% yield. M.p.: 189–192 °C dec. NMR ^1^H (400 MHz, DMSO-*d6*): see Table 7. NMR ^13^C (100 MHz, DMSO-*d6*): see Table 8. HRMS: *m/z* 504.1089 [M − HBr]^+^ (calcd. for (C_25_H_20_O_6_N_4_^32^S_1_)^+^: 504.1098). Anal. Calcd. for C_25_H_21_BrO_6_N_4_S_1_ (585.42): Br, 13.65%. Found: Br, 13.59%. 

*2-[(1E)-(2-{4-[(1S)-12-Acetyl-3,5,11-trihydroxy-1,4-dimethyl-13-oxo-8-oxatricyclo [7.4.0.0^2,7^] trideca-2(7),3,5,9,11-pentaen-6-yl]-1,3-thiazol-2-yl}hydrazin-1-ylidene)methyl]pyridin-1- ium bromide(−)-16b*: Orange amorphous powder with a 63% yield. 

*3-[(1E)-(2-{4-[(1R)-12-Acetyl-3,5,11-trihydroxy-1,4-dimethyl-13-oxo-8-oxatricyclo[7.4.0.0^2,7^] trideca-2(7),3,5,9,11-pentaen-6-yl]-1,3-thiazol-2-yl}hydrazin-1-ylidene)methyl]pyridin-1- ium bromide(+)-16c*: Orange amorphous powder with a 58% yield. M.p.: 185–188 °C dec. NMR ^1^H (400 MHz, DMSO-*d6*): Table 7. NMR ^13^C (100 MHz, DMSO-*d6*): see Table 8. HRMS: *m/z* 504.1104 [M − HBr]^+^ (calcd. for (C_25_H_20_O_6_N_4_^32^S_1_)^+^: 504.1098). Anal. Calcd. for C_25_H_21_BrO_6_N_4_S_1_ (585.42): Br, 13.65%. Found: Br, 13.62%. 

*3-[(1E)-(2-{4-[(1S)-12-Acetyl-3,5,11-trihydroxy-1,4-dimethyl-13-oxo-8-oxatricyclo[7.4.0.0^2,7^]trideca-2(7),3,5,9,11-pentaen-6-yl]-1,3-thiazol-2-yl}hydrazin-1-ylidene)methyl]pyridin-1- ium bromide(−)-16c*: Orange amorphous powder with a 54% yield. 

*(2R)-4-Acetyl-5,11,13-trihydroxy-2,12-dimethyl-10-{2-[(E)-2-[(5-nitrothiophen-2-yl)methylidene]hydrazin-1-yl]-1,3-thiazol-4-yl}-8-oxatricyclo[7.4.0.0^2,7^]trideca-1(9),4,6,10,12-pentaen-3-one (+)-16d*: Red amorphous powder with a 74% yield. M.p.: 195–197 °C dec. NMR ^1^H (400 MHz, DMSO-*d6*): see Table 7. NMR ^13^C (100 MHz, DMSO-*d6*): see Table 8. HRMS: *m/z* 554.0555 [M]^+^ (calcd. for (C_24_H_18_O_8_N_4_^32^S_2_)^+^: 554.0561). 

*(2S)-4-Acetyl-5,11,13-trihydroxy-2,12-dimethyl-10-{2-[(E)-2-[(5-nitrothiophen-2-yl)methylidene]hydrazin-1-yl]-1,3-thiazol-4-yl}-8-oxatricyclo[7.4.0.0^2,7^]trideca-1(9),4,6,10,12-pentaen-3-one (-)-16*d: Red amorphous powder with a 75% yield. 

*(2R)-4-Acetyl-5,11,13-trihydroxy-2,12-dimethyl-10-{2-[(E)-2-[(5-methylthiophen-2-yl)methylidene]hydrazin-1-yl]-1,3-thiazol-4-yl}-8-oxatricyclo[7.4.0.0^2,7^]trideca-1(9),4,6,10,12-pentaen-3-one (+)-16e*: Brown amorphous powder with a 66% yield. M.p.: 165–167 °C dec. NMR ^1^H (400 MHz, DMSO-*d6*): see Table 7. NMR ^13^C (100 MHz, DMSO-*d6*): see Table 8. HRMS: *m/z* 523.0860 [M]^+^ (calcd. for (C_25_H_21_O_6_N_3_S_2_)^+^: 523.0866). 

*(2S)-4-Acetyl-5,11,13-trihydroxy-2,12-dimethyl-10-{2-[(E)-2-[(5-methylthiophen-2-yl)methylidene]hydrazin-1-yl]-1,3-thiazol-4-yl}-8-oxatricyclo[7.4.0.0^2,7^]trideca-1(9),4,6,10,12-pentaen-3-one (-)-16e*: Brown amorphous powder with a 65% yield. 

*(2R)-4-Acetyl-5,11,13-trihydroxy-2,12-dimethyl-10-{2-[(E)-2-[(5-bromothiophen-2-yl)methylidene]hydrazin-1-yl]-1,3-thiazol-4-yl}-8-oxatricyclo[7.4.0.0^2,7^]trideca-1(9),4,6,10,12-pentaen-3-one (+)-16f*: Brown amorphous powder with a 75% yield. M.p.: 153–155 °C dec. NMR ^1^H (400 MHz, CDCl_3_): see Table 9. Spectra NMR ^13^C (100 MHz, CDCl_3_): see Table 10. HRMS: *m/z* 586.9820 [M]^+^ (calcd. for (C_24_H_18_O_6_N_3_BrS_2_)^+^: 586.9815). 

*(2S)-4-Acetyl-5,11,13-trihydroxy-2,12-dimethyl-10-{2-[(E)-2-[(5-bromothiophen-2-yl)methylidene]hydrazin-1-yl]-1,3-thiazol-4-yl}-8-oxatricyclo[7.4.0.0^2,7^]trideca-1(9),4,6,10,12-pentaen-3-one (–)-16f*: Brown amorphous powder with a 78% yield. 

*(2R)-4-Acetyl-5,11,13-trihydroxy-2,12-dimethyl-10-{2-[(E)-2-[(thiophen-2-yl)methylidene] hydrazin-1-yl]-1,3-thiazol-4-yl}-8-oxatricyclo[7.4.0.0^2,7^]trideca-1(9),4,6,10,12-pentaen-3-one (+)-16g*: Brown amorphous powder with a 71% yield. M.p.: 160–162 °C dec. NMR ^1^H (400 MHz, CDCl_3_): see Table 9. NMR ^13^C (100 MHz, CDCl_3_): see Table 10. HRMS: *m/z* 509.0707 [M]^+^ (calcd. for (C_24_H_19_O_6_N_3_^32^S_2_)^+^: 509.0710). 

*(2S)-4-Acetyl-5,11,13-trihydroxy-2,12-dimethyl-10-{2-[(E)-2-[(thiophen-2-yl)methylidene]hydrazin-1-yl]-1,3-thiazol-4-yl}-8-oxatricyclo[7.4.0.0^2,7^]trideca-1(9),4,6,10,12-pentaen-3-one (−)-16g*: Brown amorphous powder with a 70% yield. 

*(2R)-4-Acetyl-5,11,13-trihydroxy-2,12-dimethyl-10-{2-[(E)-2-[(4-bromothiophen-2-yl)methylidene]hydrazin-1-yl]-1,3-thiazol-4-yl}-8-oxatricyclo[7.4.0.0^2,7^]trideca-1(9),4,6,10,12- pentaen-3-one (+)-16h*: Brown amorphous powder with a 73% yield. M.p.: 176–178 °C dec. NMR ^1^H (400 MHz, CDCl_3_): see Table 9. NMR ^13^C (100 MHz, CDCl_3_): see Table 10. HRMS: *m/z* 586.9807 [M]^+^ (calcd. for (C_24_H_18_O_6_N_3_^79^Br_1_^32^S_1_)^+^: 586.9815). 

*(2S)-4-Acetyl-5,11,13-trihydroxy-2,12-dimethyl-10-{2-[(E)-2-[(4-bromothiophen-2-yl)methylidene]hydrazin-1-yl]-1,3-thiazol-4-yl}-8-oxatricyclo[7.4.0.0^2,7^]trideca-1(9),4,6,10,12-pentaen-3-one (-)-16h*: Brown amorphous powder with a 77% yield. 

*(2R)-4-Acetyl-5,11,13-trihydroxy-2,12-dimethyl-10-{2-[(E)-2-[(3-methylthiophen-2-yl)methylidene]hydrazin-1-yl]-1,3-thiazol-4-yl}-8-oxatricyclo[7.4.0.0^2,7^]trideca-1(9),4,6,10,12-pentaen-3-one (+)-16i*: Brown amorphous powder with a 65% yield. M.p.: 219–220 °C dec. NMR ^1^H (400 MHz, CDCl_3_): see Table 9. NMR ^13^C (100 MHz, CDCl_3_): see Table 10. HRMS: *m/z* 523.0864 [M]^+^ (calcd. for (C_25_H_21_O_6_N_3_S_2_)^+^: 523.0866). 

*(2S)-4-Acetyl-5,11,13-trihydroxy-2,12-dimethyl-10-{2-[(E)-2-[(3-methylthiophen-2-yl) methylidene]hydrazin-1-yl]-1,3-thiazol-4-yl}-8-oxatricyclo[7.4.0.0^2,7^]trideca-1(9),4,6,10,12-pentaen-3-one (-)-16i*: Brown amorphous powder with a 67% yield. 

*(2R)-4-Acetyl-5,11,13-trihydroxy-2,12-dimethyl-10-{2-[(E)-2-[(thiophen-3-yl)methylidene] hydrazin-1-yl]-1,3-thiazol-4-yl}-8-oxatricyclo[7.4.0.0^2,7^]trideca-1(9),4,6,10,12-pentaen-3-one amine (+)-16j*: Brown amorphous powder with a 67% yield. M.p.: 152–155 °C dec. NMR ^1^H (400 MHz, CDCl_3_): see Table 9. NMR ^13^C (100 MHz, CDCl_3_): see Table 10. HRMS: *m/z* 509.0705 [M]^+^ (calcd. for ((C_24_H_19_O_6_N^32^S_2_)^+∙^: 509.0710). 

*(2S)-4-Acetyl-5,11,13-trihydroxy-2,12-dimethyl-10-{2-[(E)-2-[(thiophen-3-yl)methylidene] hydrazin-1-yl]-1,3-thiazol-4-yl}-8-oxatricyclo[7.4.0.0^2,7^]trideca-1(9),4,6,10,12-pentaen-3-one amine (-)-16j*: Brown amorphous powder with a 70% yield. 

*(2R)-4-Acetyl-5,11,13-trihydroxy-2,12-dimethyl-10-{2-[(E)-2-[(furan-2-yl)methylidene] hydrazin-1-yl]-1,3-thiazol-4-yl}-8-oxatricyclo[7.4.0.0^2,7^]trideca-1(9),4,6,10,12-pentaen-3-one (+)-16k*: Brown amorphous powder with a 65% yield. M.p.: 152–154 °C dec. NMR ^1^H (400 MHz, CDCl_3_): see Table 11. NMR ^13^C (100 MHz, CDCl_3_): see Table 12. HRMS: *m/z* 493.0942 [M]^+^ (calcd. for (C_24_H_19_O_7_N_3_^32^S_1_)^+^: 493.0938). 

*(2S)-4-Acetyl-5,11,13-trihydroxy-2,12-dimethyl-10-{2-[(E)-2-[(furan-2-yl)methylidene] hydrazin-1-yl]-1,3-thiazol-4-yl}-8-oxatricyclo[7.4.0.0^2,7^]trideca-1(9),4,6,10,12-pentaen-3-one (-)-16k*: Brown amorphous powder with a 63% yield. 

*(2R)-4-Acetyl-5,11,13-trihydroxy-2,12-dimethyl-10-{2-[(E)-2-[(furan-3-yl)methylidene] hydrazin-1-yl]-1,3-thiazol-4-yl}-8-oxatricyclo[7.4.0.0^2,7^]trideca-1(9),4,6,10,12-pentaen-3-one (+)-16l*: Brown amorphous powder with a 78% yield. M.p.: 155–157 °C dec. NMR ^1^H (400 MHz, CDCl_3_): see Table 11. NMR ^13^C (100 MHz, CDCl_3_): see Table 12. HRMS: *m/z* 493.0941 [M]^+^ (calcd. for (C_24_H_19_O_7_N_3_^32^S_1_)^+^: 493.0938). 

*(2S)-4-Acetyl-5,11,13-trihydroxy-2,12-dimethyl-10-{2-[(E)-2-[(furan-3-yl)methylidene] hydrazin-1-yl]-1,3-thiazol-4-yl}-8-oxatricyclo[7.4.0.0^2,7^]trideca-1(9),4,6,10,12-pentaen-3-one (-)-16l*: Brown amorphous powder with a 75% yield. 

*(2R)-4-Acetyl-5,11,13-trihydroxy-2,12-dimethyl-10-{2-[(E)-2-[(1H-pyrrol-2-yl)methylidene] hydrazin-1-yl]-1,3-thiazol-4-yl}-8-oxatricyclo[7.4.0.0^2,7^]trideca-1(9),4,6,10,12-pentaen-3-one (+)-16m*: Brown amorphous powder with a 21% yield. M.p.: 155–157 °C dec. NMR ^1^H (400 MHz, DMSO-*d6*): see Table 11. NMR ^13^C (100 MHz, DMSO-*d6*): see Table 12. HRMS: *m/z* 492.1090 [M]^+^ (calcd. for (C_24_H_20_O_6_N_4_^32^S_1_)^+^: 492.1080). 

*(2S)-4-Acetyl-5,11,13-trihydroxy-2,12-dimethyl-10-{2-[(E)-2-[(1H-pyrrol-2-yl)methylidene] hydrazin-1-yl]-1,3-thiazol-4-yl}-8-oxatricyclo[7.4.0.0^2,7^]trideca-1(9),4,6,10,12-pentaen-3-one (-)-16m*: Brown amorphous powder with a 20% yield. 

*(2R)-4-Acetyl-5,11,13-trihydroxy-2,12-dimethyl-10-{2-[(E)-2-[(1-methyl-pyrrol-2-yl) methylidene]hydrazin-1-yl]-1,3-thiazol-4-yl}-8-oxatricyclo[7.4.0.0^2,7^]trideca-1(9),4,6,10,12-pentaen-3-one (+)-16n*: Brown amorphous powder with an 80% yield. M.p.: 196–198 °C dec. NMR ^1^H (400 MHz, CDCl_3_): see Table 11. NMR ^13^C (100 MHz, CDCl_3_): see Table 12. HRMS: *m/z* 506.1258 [M]^+^ (calcd. for (C_25_H_22_O_6_N_4_S_1_)^+^: 506.1255). 

*(2S)-4-Acetyl-5,11,13-trihydroxy-2,12-dimethyl-10-{2-[(E)-2-[(1-methyl-pyrrol-2-yl) methylidene]hydrazin-1-yl]-1,3-thiazol-4-yl}-8-oxatricyclo[7.4.0.0^2,7^]trideca-1(9),4,6,10,12-pentaen-3-one (−)-16n*: Brown amorphous powder with an 81% yield. 

*5-[(1E)-(2-{4-[(1R)-12-Acetyl-3,5,11-trihydroxy-1,4-dimethyl-13-oxo-8-oxatricyclo[7.4.0.0^2,7^]trideca-2(7),3,5,9,11-pentaen-6-yl]-1,3-thiazol-2-yl}hydrazin-1-ylidene)methyl]-1H-imidazol-1-ium bromide(+)-16o*: Brown amorphous powder with a 79% yield. M.p.: 168–172 °C dec. NMR ^1^H (400 MHz, DMSO-*d6*): see Table 11. NMR ^13^C (100 MHz, DMSO-*d6*): see Table 12. HRMS: *m/z* 493.1045 [M]^+^ (calcd. for (C_23_H_19_O_6_N_5_S_1_)^+^: 493.1051). Anal. Calcd. for C_25_H_21_BrO_6_N_4_S_1_ (585.42): Br, 13.91%. Found: Br, 13.90%. 

*5-[(1E)-(2-{4-[(1S)-12-Acetyl-3,5,11-trihydroxy-1,4-dimethyl-13-oxo-8-oxatricyclo[7.4.0.0^2,7^]trideca-2(7),3,5,9,11-pentaen-6-yl]-1,3-thiazol-2-yl}hydrazin-1-ylidene)methyl]-1H-imidazol-1-ium bromide(−)-16o*: Brown amorphous powder with a 79% yield. 

*(2R)-4-Acetyl-5,11,13-trihydroxy-2,12-dimethyl-10-{2-[(E)-2-[(1H-indole-3-yl)methylidene] hydrazin-1-yl]-1,3-thiazol-4-yl}-8-oxatricyclo[7.4.0.0^2,7^]trideca-1(9),4,6,10,12-pentaen-3-one (+)-16p*: Brown amorphous powder with an 80% yield. M.p.: 191–192 °C dec. NMR ^1^H (400 MHz, DMSO-*d6*): see Table 13. NMR ^13^C (100 MHz, DMSO-*d6*): see Table 14. HRMS: *m/z* 542.1243 [M]^+^ (calcd. for (C_28_H_22_O_6_N_4_^32^S_1_)^+^: 542.1255). 

*(2S)-4-Acetyl-5,11,13-trihydroxy-2,12-dimethyl-10-{2-[(E)-2-[(1H-indole-3-yl)methylidene] hydrazin-1-yl]-1,3-thiazol-4-yl}-8-oxatricyclo[7.4.0.0^2,7^]trideca-1(9),4,6,10,12-pentaen-3-one (-)-16p*: Brown amorphous powder with a 79% yield. 

*(2R)-4-Acetyl-5,11,13-trihydroxy-2,12-dimethyl-10-{2-[(E)-2-[(1H-2-methyl-indole-3-yl) methylidene]hydrazin-1-yl]-1,3-thiazol-4-yl}-8-oxatricyclo[7.4.0.0^2,7^]trideca-1(9),4,6,10,12-pentaen-3-one (+)-16q*: Brown amorphous powder with an 89% yield. M.p.: 186–189 °C dec. NMR ^1^H (400 MHz, DMSO-*d6*): see Table 13. NMR ^13^C (100 MHz, DMSO-*d6*): see Table 14. HRMS: *m/z* 556.1415 [M]^+^ (calcd. for (C_29_H_24_O_6_N_4_S_1_)^+^: 556.1411). 

*(2S)-4-Acetyl-5,11,13-trihydroxy-2,12-dimethyl-10-{2-[(E)-2-[(1H-3-methyl-indole-3-yl) methylidene]hydrazin-1-yl]-1,3-thiazol-4-yl}-8-oxatricyclo[7.4.0.0^2,7^]trideca-1(9),4,6,10,12-pentaen-3-one (-)-16q*: Brown amorphous powder with an 88% yield. 

*(2R)-4-Acetyl-10-{2-[(E)-2-{[5-(3-chlorophenyl)furan-2-yl]methylidene}hydrazin-1-yl]-1,3-thiazol-4-yl}-5,11,13-trihydroxy-2,12-dimethyl-8-oxatricyclo[7.4.0.0^2,7^]trideca-1(9),4,6,10,12-pentaen-3-one (+)-16r*: Brown amorphous powder with an 89% yield. M.p.: 148–151 °C dec. NMR ^1^H (400 MHz, DMSO-*d6*): see Table 13. NMR ^13^C (100 MHz, DMSO-*d6*): see Table 14. HRMS: *m/z* 603.0860 [M]^+^ (calcd. for (C_30_H_22_ClO_7_N_3_S_1_)^+^: 603.0867). 

*(2S)-4-Acetyl-10-{2-[(E)-2-{[5-(3-chlorophenyl)furan-2-yl]methylidene}hydrazin-1-yl]-1,3-thiazol-4-yl}-5,11,13-trihydroxy-2,12-dimethyl-8-oxatricyclo[7.4.0.0^2,7^]trideca-1(9),4,6,10,12-pentaen-3-one (-)-16r)*: Brown amorphous powder in 87% yield. *(2R)-4-Acetyl-10-{2-[(E)-2-({3-[(4-fluorophenoxy)methyl]-4-methoxyphenyl}methylidene)hydrazin-1-yl]-1,3-thiazol-4-yl}-5,11,13-trihydroxy-2,12-dimethyl-8-oxatricyclo[7.4.0.0^2,7^]trideca-1(9),4,6,10,12-pentaen-3-one (+)-17a*: Orange amorphous powder with a 79% yield. M.p.: 120–122 °C dec. NMR ^1^H (400 MHz, CDCl_3_): see Table 15. NMR ^13^C (100 MHz, CDCl_3_): see Table 16. HRMS: *m/z* 657.1557 [M]^+^ (calcd. for (C_34_H_28_O_8_N_3_F_1_^32^S_1_)^+^: 657.1576). 

*(2S)-4-Acetyl-10-{2-[(E)-2-({3-[(4-fluorophenoxy)methyl]-4-methoxyphenyl}methylidene)hydrazin-1-yl]-1,3-thiazol-4-yl}-5,11,13-trihydroxy-2,12-dimethyl-8-oxatricyclo[7.4.0.0^2,7^]trideca-1(9),4,6,10,12-pentaen-3-one (-)-17a*: Orange amorphous powder with a 77% yield. 

*(2R)-4-Acetyl-10-{2-[(E)-2-({3-[(3-fluorophenoxy)methyl]-4-methoxyphenyl}methylidene)hydrazin-1-yl]-1,3-thiazol-4-yl}-5,11,13-trihydroxy-2,12-dimethyl-8-oxatricyclo[7.4.0.0^2,7^]trideca-1(9),4,6,10,12-pentaen-3-one (+)-17b*: Orange amorphous powder with a 70% yield. M.p.: 119–122 °C dec. NMR ^1^H (400 MHz, CDCl_3_): see Table 15. NMR ^13^C (100 MHz, CDCl_3_): see Table 16. HRMS: *m/z* 657.1590 [M]^+^ (calcd. for (C_34_H_28_O_8_N_3_F_1_^32^S_1_)^+^: 657.1576). 

*(2S)-4-Acetyl-10-{2-[(E)-2-({3-[(3-fluorophenoxy)methyl]-4-methoxyphenyl}methylidene)hydrazin-1-yl]-1,3-thiazol-4-yl}-5,11,13-trihydroxy-2,12-dimethyl-8-oxatricyclo[7.4.0.0^2,7^]trideca-1(9),4,6,10,12-pentaen-3-one (-)-17b*: Orange amorphous powder with a 65% yield. 

*(2R)-4-Acetyl-10-{2-[(E)-2-({3-[(2-fluorophenoxy)methyl]-4-methoxyphenyl}methylidene)hydrazin-1-yl]-1,3-thiazol-4-yl}-5,11,13-trihydroxy-2,12-dimethyl-8-oxatricyclo[7.4.0.0^2,7^]trideca-1(9),4,6,10,12-pentaen-3-one (+)-17c*: Orange amorphous powder with a 94% yield. M.p.: 118–121 °C dec. NMR ^1^H (400 MHz, CDCl_3_): see Table 15. NMR ^13^C (100 MHz, CDCl_3_): see Table 16. HRMS: *m/z* 657.1590 [M]^+^ (calcd. for (C_34_H_28_O_8_N_3_F_1_^32^S_1_)^+^: 657.1576). 

*(2S)-4-Acetyl-10-{2-[(E)-2-({3-[(2-fluorophenoxy)methyl]-4-methoxyphenyl}methylidene)hydrazin-1-yl]-1,3-thiazol-4-yl}-5,11,13-trihydroxy-2,12-dimethyl-8-oxatricyclo[7.4.0.0^2,7^]trideca-1(9),4,6,10,12-pentaen-3-one (-)-17c*: Orange amorphous powder with a 97% yield. 

*(2R)-4-Acetyl-10-{2-[(E)-2-({3-[(3-chloro-4-fluorophenoxy)methyl]-4-methoxyphenyl}methylidene)hydrazin-1-yl]-1,3-thiazol-4-yl}-5,11,13-trihydroxy-2,12-dimethyl-8-oxatricyclo[7.4.0.0^2,7^]trideca-1(9),4,6,10,12-pentaen-3-one (+)-17d*: Orange amorphous powder with a 95% yield. M.p.: 123–126 °C dec. NMR ^1^H (400 MHz, CDCl_3_): see Table 15. NMR ^13^C (100 MHz, CDCl_3_): see Table 16. HRMS: *m/z* 691.1194 [M]^+^ (calcd. for (C_34_H_27_O_8_N_3_^35^Cl_1_F_1_^32^S_1_)^+^: 691.1186). 

*(2S)-4-Acetyl-10-{2-[(E)-2-({3-[(3-chloro-4-fluorophenoxy)methyl]-4-methoxyphenyl}methylidene)hydrazin-1-yl]-1,3-thiazol-4-yl}-5,11,13-trihydroxy-2,12-dimethyl-8-oxatricyclo[7.4.0.0^2,7^]trideca-1(9),4,6,10,12-pentaen-3-one (-)-17d*: Orange amorphous powder with a 97% yield. 

*(2R)-4-Acetyl-10-{2-[(E)-2-({3-[(3,4-difluorophenoxy)methyl]-4-methoxyphenyl}methylidene)hydrazin-1-yl]-1,3-thiazol-4-yl}-5,11,13-trihydroxy-2,12-dimethyl-8-oxatricyclo[7.4.0.0^2,7^]trideca-1(9),4,6,10,12-pentaen-3-one (+)-17e*: Orange amorphous powder with a 96% yield. M.p.: 127–130 °C dec. NMR ^1^H (400 MHz, CDCl_3_): see Table 15. NMR ^13^C (100 MHz, CDCl_3_): see Table 16. HRMS: *m/z* 675.1474 [M]^+^ (calcd. for (C_34_H_27_O_8_N_3_F_2_^32^S_1_)^+^: 675.1481). 

*(2S)-4-Acetyl-10-{2-[(E)-2-({3-[(3,4-difluorophenoxy)methyl]-4-methoxyphenyl}methylidene)hydrazin-1-yl]-1,3-thiazol-4-yl}-5,11,13-trihydroxy-2,12-dimethyl-8-oxatricyclo[7.4.0.0^2,7^]trideca-1(9),4,6,10,12-pentaen-3-one (-)-17e*: Orange amorphous powder with a 93% yield. 

*(2R)-4-Acetyl-10-{2-[(E)-2-({3-[(4-chloro-3-fluorophenoxy)methyl]-4-methoxyphenyl}methylidene)hydrazin-1-yl]-1,3-thiazol-4-yl}-5,11,13-trihydroxy-2,12-dimethyl-8-oxatricyclo[7.4.0.0^2,7^]trideca-1(9),4,6,10,12-pentaen-3-one (+)-17f*: Orange amorphous powder with a 95% yield. M.p.: 129–133 °C dec. NMR ^1^H (400 MHz, CDCl_3_): see Table 15. NMR ^13^C (100 MHz, CDCl_3_): see Table 16. HRMS: *m/z* 691.1195 [M]^+^ (calcd. for (C_34_H_27_O_8_N_3_^35^Cl_1_F_1_^32^S_1_)^+^: 691.1186). 

*(2S)-4-Acetyl-10-{2-[(E)-2-({3-[(4-chloro-3-fluorophenoxy)methyl]-4-methoxyphenyl}methylidene)hydrazin-1-yl]-1,3-thiazol-4-yl}-5,11,13-trihydroxy-2,12-dimethyl-8-oxatricyclo[7.4.0.0^2,7^]trideca-1(9),4,6,10,12-pentaen-3-one (-)-17f*: Orange amorphous powder with a 94% yield. 

*(2R)-4-Acetyl-10-{2-[(E)-2-[(3-{[(2-tert-butyl-5-methylphenyl)sulfanyl]methyl}-4-methoxyphenyl)methylidene]hydrazin-1-yl]-1,3-thiazol-4-yl}-5,11,13-trihydroxy-2,12-dimethyl-8-oxatricyclo[7.4.0.0^2,7^]trideca-1(9),4,6,10,12-pentaen-3-one (+)-17g*: Orange amorphous powder with a 92% yield. M.p.: 103–105 °C dec. NMR ^1^H (400 MHz, CDCl_3_): see Table 17. NMR ^13^C (100 MHz, CDCl_3_): see Table 18. HRMS: *m/z* 725.2230 [M]^+^ (calcd. for (C_39_H_39_O_7_N_3_^32^S_2_)^+^: 725.2224). 

*(2S)-4-Acetyl-10-{2-[(E)-2-[(3-{[(2-tert-butyl-5-methylphenyl)sulfanyl]methyl}-4-methoxyphenyl)methylidene]hydrazin-1-yl]-1,3-thiazol-4-yl}-5,11,13-trihydroxy-2,12-dimethyl-8-oxatricyclo[7.4.0.0^2,7^]trideca-1(9),4,6,10,12-pentaen-3-one (-)-17g*: Orange amorphous powder with a 90% yield. 

*(2R)-4-Acetyl-10-{2-[(E)-2-({3-[(4-chloro-3-methylphenoxy)methyl]-4-methoxyphenyl}methylidene)hydrazin-1-yl]-1,3-thiazol-4-yl}-5,11,13-trihydroxy-2,12-dimethyl-8-oxatricyclo[7.4.0.0^2,7^]trideca-1(9),4,6,10,12-pentaen-3-one (+)-17h*: Orange amorphous powder with a 56% yield. M.p.: 125–127 °C dec. NMR ^1^H (400 MHz, CDCl_3_): see Table 17. NMR ^13^C (100 MHz, CDCl_3_): see Table 18. HRMS: *m/z* 687.1432 [M]^+^ (calcd. for (C_35_H_30_O_8_N_3_^32^S_1_^35^Cl_1_)^+^: 687.1437). 

*(2S)-4-Acetyl-10-{2-[(E)-2-({3-[(4-chloro-3-methylphenoxy)methyl]-4-methoxyphenyl}methylidene)hydrazin-1-yl]-1,3-thiazol-4-yl}-5,11,13-trihydroxy-2,12-dimethyl-8-oxatricyclo[7.4.0.0^2,7^]trideca-1(9),4,6,10,12-pentaen-3-one (-)-17h*: Orange amorphous powder with a 51% yield. 

*(2R)-4-Acetyl-10-{2-[(E)-2-[(3-{[4-(4-fluorophenyl)piperazin-1-yl]methyl}-4-methoxyphenyl)methylidene]hydrazin-1-yl]-1,3-thiazol-4-yl}-5,11,13-trihydroxy-2,12-dimethyl-8-oxatricyclo[7.4.0.0^2,7^]trideca-1(9),4,6,10,12-pentaen-3-one (+)-17i*: Orange amorphous powder with a 91% yield. M.p.: 175–179 °C dec. NMR ^1^H (400 MHz, CDCl_3_): see Table 17. NMR ^13^C (100 MHz, CDCl_3_): see Table 18. HRMS: *m/z* 725.2274 [M]^+^ (calcd. for (C_38_H_36_O_7_N_5_F_1_S_1_)^+^: 725.2314). 

*(2S)-4-Acetyl-10-{2-[(E)-2-[(3-{[4-(4-fluorophenyl)piperazin-1-yl]methyl}-4-methoxyphenyl)methylidene]hydrazin-1-yl]-1,3-thiazol-4-yl}-5,11,13-trihydroxy-2,12-dimethyl-8-oxatricyclo[7.4.0.0^2,7^]trideca-1(9),4,6,10,12-pentaen-3-one (-)-17i*: Orange amorphous powder with an 85% yield. 

*(2R)-4-Acetyl-10-{2-[(E)-2-[(3-{[4-(2-methoxyphenyl)piperazin-1-yl]methyl}-4-methoxyphenyl)methylidene]hydrazin-1-yl]-1,3-thiazol-4-yl}-5,11,13-trihydroxy-2,12-dimethyl-8-oxatricyclo[7.4.0.0^2,7^]trideca-1(9),4,6,10,12-pentaen-3-one (+)-17j*: Orange amorphous powder with a 50% yield. M.p.: 171–175 °C dec. NMR ^1^H (400 MHz, CDCl_3_): see Table 17. NMR ^13^C (100 MHz, CDCl_3_): see Table 18. HRMS: *m/z* 737.8204 [M]^+^ (calcd. for (C_39_H_39_O_8_N_5_S_1_)^+^: 737.8207). 

*(2S)-4-Acetyl-10-{2-[(E)-2-[(3-{[4-(2-methoxyphenyl)piperazin-1-yl]methyl}-4-methoxyphenyl)methylidene]hydrazin-1-yl]-1,3-thiazol-4-yl}-5,11,13-trihydroxy-2,12-dimethyl-8-oxatricyclo[7.4.0.0^2,7^]trideca-1(9),4,6,10,12-pentaen-3-one (-)-17j*: Orange amorphous powder with a 53% yield. 

*(2R)-4-Acetyl-10-{2-[(E)-2-({3-[(3,5-dimethyl-4-nitro-1H-pyrazol-1-yl)methyl]-4-methoxyphenyl}methylidene)hydrazin-1-yl]-1,3-thiazol-4-yl}-5,11,13-trihydroxy-2,12-dimethyl-8-oxatricyclo[7.4.0.0^2,7^]trideca-1(9),4,6,10,12-pentaen-3-one (+)-17k*: Orange amorphous powder with an 85% yield. M.p.: 160–165 °C dec. NMR ^1^H (400 MHz, CDCl_3_): see Table 17. NMR ^13^C (100 MHz, CDCl_3_): see Table 18. HRMS: *m/z* 686.1806 [M]^+^ (calcd. for (C_33_H_30_O_9_N_6_^32^S_1_)^+^: 686.1790).

*(2S)-4-Acetyl-10-{2-[(E)-2-({3-[(3,5-dimethyl-4-nitro-1H-pyrazol-1-yl)methyl]-4-methoxyphenyl}methylidene)hydrazin-1-yl]-1,3-thiazol-4-yl}-5,11,13-trihydroxy-2,12-dimethyl-8-oxatricyclo[7.4.0.0^2,7^]trideca-1(9),4,6,10,12-pentaen-3-one (-)-17k*: Orange amorphous powder with an 87% yield.

### 3.2. Real-Time Detection of Tdp1 Activity

A fluorophore quencher-coupled DNA-biosensor for real-time measurement of Tdp1 cleavage activity was recently designed in our laboratory [9]. The biosensor is a 16-mer single-stranded oligonucleotide containing both a 5’-FAM fluorophore donor and a quenching 3’-BHQ1 moiety. 

Recombinant protein Tdp1 was expressed in *EsCherichia coli* (pET 16B plasmid containing Tdp1 cDNA provided by Dr. K.W. Caldecott, University of Sussex, United Kingdom) and isolated as described [3,50]. The reaction mixture in a final volume 200 μL contained Tdp1 reaction buffer (50 mM Tris-HCl, 50 mM NaCl, 7 mM β-mercaptoethanol), 50 nM oligonucleotide substrate, and varied concentrations of potential inhibitor. Purified Tdp1 was added in a final concentration of 1.5 nM.

The reaction mixtures were incubated at a constant temperature of 26 °C in a POLARstar OPTIMA fluorimeter, BMG LABTECH, GmbH, to measure fluorescence intensity every minute (Ex485/Em520 nm). Tdp1 inhibition was calculated by comparing the rate of increase in fluorescence of the biosensor in the presence of the compound to that of DMSO (1.5%) control wells. The measurements were carried out in at least two independent experiments. IC_50_ values were determined using a six-point concentration response curve. The data were imported into the MARS Data Analysis 2.0 program (BMG LABTECH) and the slope during the linear phase (data from 0 to 7 min) was calculated.

### 3.3. Binding Assay

pET-28a(+) plasmid encoding recombinant Tdp1 (residues 149–608) was obtained from GenScript. *E. coli* BL21(DE3) was used for recombinant protein production. Protein production was induced with 1 mM IPTG at 28 °C with overnight incubation. Tdp1 was purified using affinity and size exclusion chromatography. Fluorescence was measured using a PerkinElmer EnSpire Multimode Reader. Tdp1 concentration was 10 μM with varying compound concentrations. The buffer was 20 mM Tris and 250 mM NaCl (pH 8). The excitation wavelength was 280 nm and intrinsic fluorescence was measured at 350 nm. Control experiments with Tdp1 or the compound on its own were also conducted. Background fluorescence arising from the compounds was subtracted from the final spectrum. The total volume per well was 30 μL. The dissociation constant (*K*_D_) was calculated using Equation (1), which takes non-specific binding into account.

y = *B*_*max*_x/(*K*_D_ + x) + *N*_*s*_x (1)

where y denotes the changes in fluorescence intensity; x denotes the concentration of the compounds; *B*_max_ denotes the maximum changes in fluorescence intensity; and *N*_s_ is a scaling factor for non-specific binding. Experiments were conducted in triplicate.

### 3.4. Cytotoxicity Assay

Analysis of the intrinsic toxicity of the compounds was examined against human cell line HeLa (cervical cancer) using a standard MTT test [51] by colorimetric measurement of the amount of formazan converted from 3-(4,5-dimethylthiazol-2-yl)-2,5-diphenyl-2H-tetrazolium bromide in cells exposed to the compounds. The cells were grown in Iscove’s Modified Dulbecco’s Medium (IMDM), with 40 μg/mL gentamicin, 50 IU/mL penicillin, 50 μg/mL streptomycin (MP Biomedicals), and in the presence of 10% fetal bovine serum (Biolot) in a 5% CO_2_ atmosphere. After formation of a 30–50% monolayer, tested compounds were added to the medium (the volume of added reagents was 1/100 of the total volume of the culture medium, the amount of DMSO was 1% of the final volume), and the cell culture was monitored for three days. Control cells were grown in the presence of 1% DMSO. The measurements were carried out in three parallel experiments.

### 3.5. Modeling and Screening

The compounds were docked against the crystal structure of Tdp1 (PDB ID: 6DIE, resolution 1.78 Å) [34], which was obtained from the Protein Data Bank (PDB) [52,53]. The Scigress version FJ 2.6 program [54] was used to prepare the crystal structure for docking (i.e., the hydrogen atoms were added, the co-crystallized ligand benzene-1,2,4-tricarboxylic acid was removed, and the crystallographic water molecules in one instance and the other with water molecules, HOH 814, 821 and 1078. The Scigress software suite was also used to build the inhibitors and the MM2 [55] force field was used to optimize the structures. The docking center was defined as the position of a carbon on the ring of benzene-1, 2, 4-tricarboxylic acid (x = −6.052, y = −14.428, z = 33.998) with a 10 Å radius. Fifty docking runs were allowed for each ligand with the default search efficiency (100%). The basic amino acids lysine and arginine were defined as protonated. Furthermore, aspartic and glutamic acids were assumed to be deprotonated. The GoldScore (GS) [56] and ChemScore (CS) [57,58], improved the piecewise linear potential (ChemPLP) [59] and Astex statistical potential (ASP) [60] scoring functions were implemented to validate the predicted binding modes and relative energies of the ligands using the GOLD v5.4.1 software suite. 

The QikProp 3.2 [61] software package was used to calculate the molecular descriptors of the molecules. The reliability of it is established for the calculated descriptors [62]. The known drug index (KDI) was calculated from the molecular descriptors [37].

## 4. Conclusions

Two sets of new hydrazinothiazole derivatives of usnic acid were synthesized. Most of these compounds were found to be very potent inhibitors of Tdp1 with IC_50_ in a low nanomolar range. The ability of several compounds to enhance the cytotoxicity of topotecan, an established topoisomerase 1 poison, was demonstrated. Thus, a new structural type of Tdp1 inhibitors was found. These are promising candidates for adjunctive therapy to enhance the efficacy of Top1 poisons. 

## Figures and Tables

**Table 1:** The results from the biochemical and biophysical binding assays for ligands **16a**–**r**. The inhibition concentration at 50% (IC_50_) is given as well as the dissociation constant (*K*_D_).

Code	Compound, R	(+)-enantiomer		(−)-enantiomer	
		IC_50_/nM	*K*_D_/µM	IC_50_/nM	*K*_D_/µM
**16a**	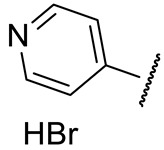	160 ± 16	139 ± 32	139 ± 38	n.d.
**16b**	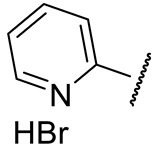	492 ± 88	77 ± 24	1480 ± 265	n.d.
**16c**	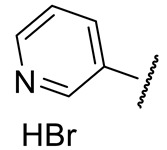	353 ± 17	n.d.^*^	154 ± 18	262 ± 25
**16d**	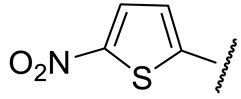	70 ± 4	597 ± 98	142 ± 4	n.d.
**16e**	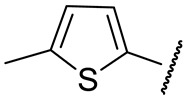	91 ± 7	n.d.	54 ± 25	n.d.
**16f**	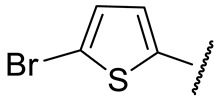	88 ± 3	95 ± 11	43 ± 1	148 ± 9
**16g**	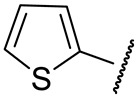	52 ± 18	n.d.	169 ± 6	281 ± 25
**16h**	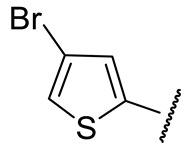	70 ± 18	n.d.	56 ± 4	355 ± 50
**16i**	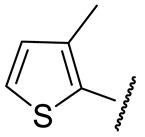	72 ± 3	n.d.	121 ± 25	n.d.
**16j**	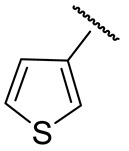	107 ± 31	65 ± 7	40 ± 3	771 ± 46
**16k**	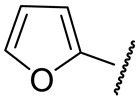	120 ± 15	67 ± 9	46 ± 24	250 ± 24
**16l**	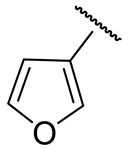	77 ± 6	n.d.	178 ± 4	233 ± 9
**16m**	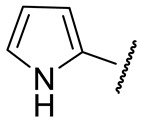	188 ± 3	n.d.	18 ± 1	354 ± 40
**16n**	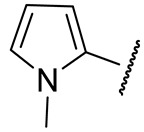	151 ± 14	719 ± 17	29 ± 9	n.d.
**16o**	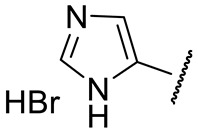	1690 ± 890	38 ± 2	570 ± 109	51 ± 4
**16p**	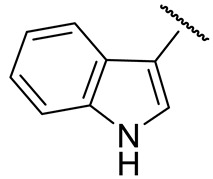	138 ± 3	268 ± 11	57 ± 1	n.d.
**16q**	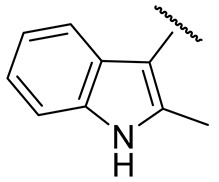	21 ± 6	n.d.	81 ± 20	58±4
**16r**	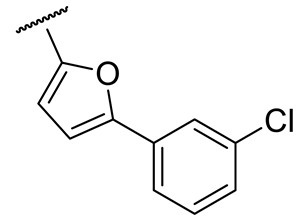	55 ± 8	113 ± 52	53 ± 3	n.d.

^*^n.d. – not determined.

**Table 2:** The results from the biochemical and biophysical binding assays for ligands **17a**–**k**. The inhibition concentration at 50% (IC_50_) is given as well as the dissociation constant (K_D_).

Code	R	(+)-enantiomer		(−)-enantiomer	
		IC_50_/nM	*K*_D_/µM	IC_50_/nM	*K*_D_/µM
**17a**	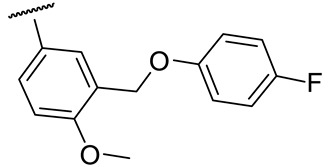	26 ± 8	212 ± 25	54 ± 3	n.d.^*^
**17b**	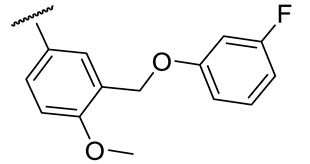	26 ± 4	131 ± 15	78 ± 3	n.d.
**17c**	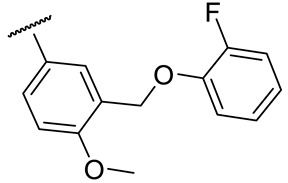	41 ± 7	204 ± 23	37 ± 10	74 ± 4
**17d**	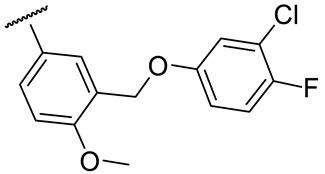	77 ± 3	130 ± 29	18 ± 1	76 ± 5
**17e**	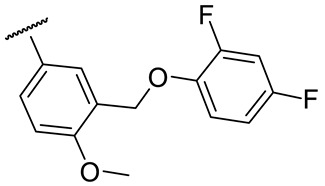	64 ± 6	213 ± 4	30 ± 11	130 ± 8
**17f**	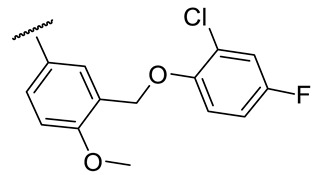	60 ± 1	127 ± 14	21 ± 5	536 ± 90
**17g**	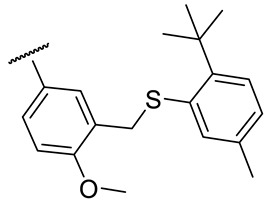	122 ± 25	223 ± 42	94 ± 20	n.d.
**17h**	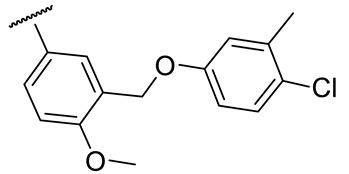	74 ± 1	188 ± 22	48 ± 2	n.d.
**17i**	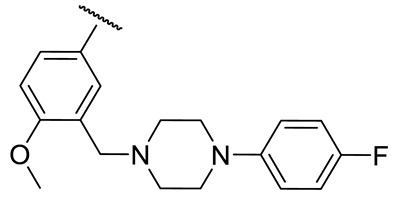	80 ± 7	161 ± 22	59 ± 5	n.d.
**17j**	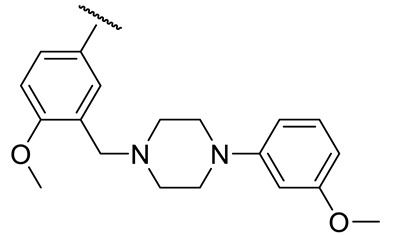	69 ± 14	69 ± 14	71 ± 15	n.d.
**17k**	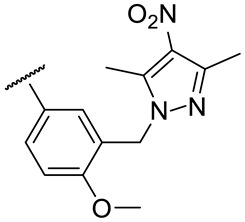	54 ± 8	156 ± 8	46 ± 5	142 ± 11

^*^n.d. – not determined.

**Table 3:**
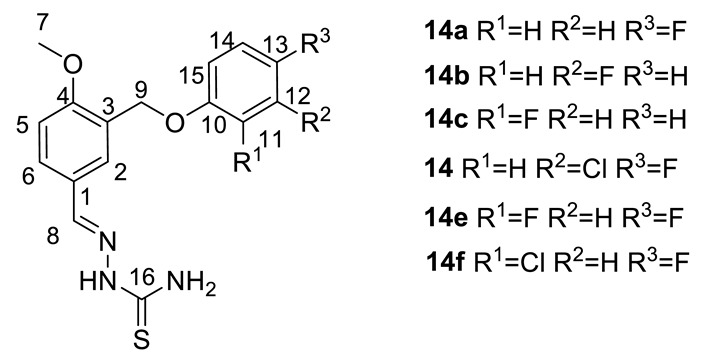
NMR ^1^H spectra of **14a**–**14f** (DMSO-*d6*, δ).

№	14a	14b	14c	14d	14e	14f
H-2	s 8.00	s 8.01	s 8.01	s 8.00	s 8.01	s 8.01
H-5	m 7.00–7.15	m 7.08–7.30	d 7.09 (J = 8.7 Hz)	d 7.07 (J = 8.6 Hz)	d 7.01 (J = 8.6 Hz)	d 7.01 (J = 8.6 Hz)
H-6	m 7.72	m 7.75	m 7.77	m 7.72	m 7.75	m 7.77
H-7	s 3.86	s 3.86	s 3.85	s 3.85	s 3.86	s 3.86
H-8	s 8.12	s 8.13	s 8.16	s 8.14	s 8.13	s 8.15
H-9	s 5.00	s 5.09	s 5.09	s 5.02	s 5.04	s 5.09
H-11	m 7.00–7.15	m 7.08–7.30		t 7.35 (J = 9.1 Hz)		
H-12	m 7.00–7.15		m 7.21		m 6.77	m 7.42
H-13		m 7.08–7.30	m 7.28			
H-14	m 7.00–7.15	m 7.08–7.30	t 7.11 (J = 7.7 Hz)	m 7.27	m 6.77	m 7.28
H-15	m 7.00–7.15	m 6.95	m 6.95	m 7.02	m 6.77	m 7.17
NH	s 11.32	s 11.33	s 11.35	s 11.34	s 11.33	s 11.33
NH_2_	m 7.90	m 7.89	m 7.90	m 7.92	m 7.93	m 7.87

**Table 4:** NMR ^13^C spectra of 14a–14f (DMSO-*d6*, δ).

№	14a	14b	14c	14d	14e	14f
C-1	125.39	124.99	124.57	124.94	124.53	124.93
C-2	129.11	129.54	129.12	129.27	129.57	129.20
C-3	126.95	126.97	126.56	126.99	127.01	126.98
C-4	159.03	159.18	158.78	159.07	159.17	159.10
C-5	111.60	111.74	111.32	111.65	111.68	111.78
C-6	129.85	129.99	129.57	130.08	130.30	129.83
C-7	56.23	56.27	55.86	56.25	56.26	56.29
C-8	142.52	142.41	141.99	142.42	142.33	142.44
C-9	65.62	66.17	65.75	66.03	66.12	66.78
C-10	155.33	d 146.83 (J = 10 Hz)	d 146.42 (J = 11 Hz)	d 155.62 (J = 2.4 Hz)	t 161.17 (J = 14 Hz)	d 151.14 (J = 2.4 Hz)
C-11	d 116.37 (J = 4 Hz)	115.69	d 150.60 (J = 248 Hz)	d 115.53 (J = 7 Hz)	d 162.40 (J = 244 Hz)	d 122.50 (J = 10 Hz)
C-12	d 116.19 (J = 11 Hz)	d151 (J = 244 Hz)	d 115.96 (J = 18 Hz)	d 120.08 (J = 17 Hz)	t 96.65 (J = 27 Hz)	d 117.51 (J = 28 Hz)
C-13	d 155.83 (J = 240 Hz)	d 116.38 (J = 18 Hz)	d 121.21 (J = 7 Hz)	d 151.12 (J = 247 Hz)	d 162.25 (J = 244 Hz)	d 155.08 (J = 247 Hz)
C-14	d 116.19 (J = 11 Hz)	d 125.30 (J = 3 Hz)	d 124.88 (J = 3 Hz)	d 117.56 (J = 22 Hz)	m 99.15	d 115.01 (J = 21 Hz)
C-15	d 116.37 (J = 4 Hz)	d 121.63 (J = 7 Hz)	115.27	116.63	m 99.43	d 115.54 (J = 9.4 Hz)
C-16	178.04	178.06	177.64	178.06	178.07	178.09

**Table 5:**
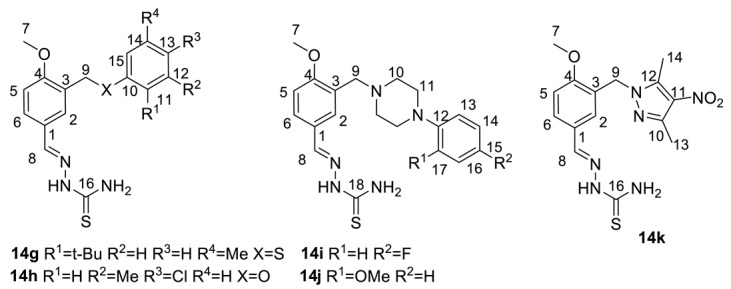
NMR ^1^H spectra of 14f–14k (DMSO-*d6*, δ).

№	14g	14h	14i	14j	14k
H-2	s 7.91	s 7.99	s 8.00	s 8.00	s 7.89
H-5	d 7.00 (J = 9.3 Hz)	m 7.04	m 7.02	d 7.05 (J = 8.5 Hz)	d 7.03 (J = 8.5 Hz)
H-6	m 7.62	m 7.72	m 7.69	bs 7.75	m 7.63
H-7	s 3.80	s 3.85	s 3.83	s 3.83	s 3.81
H-8	s 8.14	s 8.14	s 8.11	s 8.15	s 7.97
H-9	s 4.09	s 5.00	s 3.55	s 3.68	s 5.21
H-10			bs 2.57	bs 2.69	
H-11	s 1.19 (t-Bu)		bs 3.07	bs 2.93	
H-12	s 7.09	s 2.28 (Me)			
H-13	s 7.18	m 7.06	m 6.90	m 6.87–6.94	2.34
H-14	s 2.21 (Me)	m 7.28	m 7.02	m 6.87–6.94	2.67
H-15	s 7.09	m 6.86		m 6.87–6.94	
H-16			m 7.02	m 6.87–6.94	
H-17			m 6.90	s 3.74 (OMe)	
NH	s 11.32	s 11.33	s 11.29	s 11.34	s 11.33
NH_2_	s 7.62 and s 7.80	s 7.89 and s 7.93	m 7.69 and s 7.85	bs 7.72 and bs 7.90	s 7.75 and s 7.78

**Table 6:**
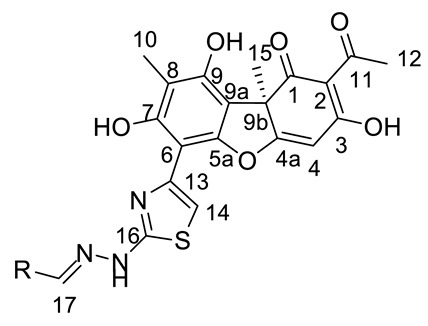
NMR ^13^C spectra of **14f**–**14k** (DMSO-*d6*, δ).

№	14g	14h	14i	14j	14k
C-1	125.87	124.88	127.95	126.36	123.74
C-2	129.13	129.36	126.19	128.64	129.05
C-3	126.31	126.52	127.95	126.36	126.59
C-4	158.47	158.57	159.03	159.28	158.26
C-5	111.11	111.19	111.16	111.38	111.30
C-6	129.59	129.50	129.64	130.46	129.37
C-7	55.77	55.80	55.67	55.89	55.83
C-8	142.02	142.12	142.24	142.42	141.87
C-9	31.52	64.84	48.73	49.30	48.10
C-10	134.24	157.29	55.06	55.05	145.04
C-11	148.8 (C, Ar) 30.88 (Me t-Bu) 34.14 (C, t-Bu)	117.36	52.36	52.55	130.02
C-12	128.25	128.60 (C, Ar) 19.79 (Me)	147.75	152.02	141.38
C-13	125.88	136.52	117.02 (J = 7 Hz)	111.92	11.39
C-14	134.58 (C, Ar) 19.32 (Me)	124.70	115.06 (J = 22 Hz)	118.13	13.90
C-15	123.05	113.82	d 155.00 (J = 232 Hz)	122.77	
C-16	177.58	177.66	115.06 (J = 22 Hz)	120.92	177.67
C-17			117.02 (J = 7 Hz)	140.08	
C-18			177.60	177.64	

NMR ^1^H (**CDCl_3_**, δ): 1.67 (3H, s, H-15), 2.13 (3H, s, H-10), 2.61 (3H, s, H-12), 5.88 (1H, s, H-4). NMR ^13^C (**CDCl_3_**, δ): 8.3 (C-10), 27.6 (C-12), 32.0 (C-15), 59.3 (C-9b), 97.2 (C-4), 97.3 (C-9a), 103.3 (C-6), 104.4 (C-14), 105.0 (C-2), 108.8 (C-8), 143.4 (C-13), 151.3 (C-7), 151.4 (C-9), 156.3 (C-5a), 166.4 (C-16), 180.4 (C-4a), 191.5 (C-3), 197.9 (C-1), 201.2 (C-11). NMR ^1^H (**DMSO-*d6***, δ): 1.70 (3H, s, H-15), 2.03 (3H, s, H-10), 2.60 (3H, s, H-12), 6.20 (1H, s, H-4). NMR ^13^C (**DMSO-*d6***, δ): 8.3 (C-10), 27.6 (C-12), 32.0 (C-15), 59.0 (C-9b), 96.9 (C-9a),97.3 (C-4), 103.3 (C-6),105.1 (C-2), 105.6 (C-14), 107.3 (C-8), 143.0 (C-13), 151.3 (C-7), 151.4 (C-9), 156.3 (C-5a), 166.4 (C-16), 180.4 (C-4a), 191.5 (C-3), 197.9 (C-1), 201.2 (C-11).

**Table 7:** NMR^1^H spectra of **16a**–**16e** (DMSO-*d6*, δ).

№	16a	16b	16c	16d	16e
**Structure**	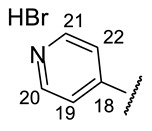	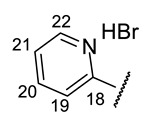	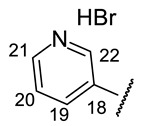	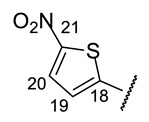	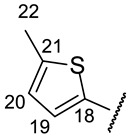
**H-14**	s 7.39	s 7.39	s 7.38	s 7.27	s 7.23
**H-17**	s 8.16	s 8.15	s 8.22	s 8.08	s 8.16
**H-19**	d 8.10 (J = 5.5 Hz)	d 8.07 (J = 8.0 Hz)	s 9.13	d 7.34 (J = 4.5 Hz)	d 7.19 (J = 4.9 Hz)
**H-20**	d 8.84 (J = 5.5 Hz)	t 7.66 (J = 5.9 Hz)	d 8.85 (J = 5.1 Hz)	d 7.98 (J = 4.5 Hz)	m 6.79
**H-21**	d 8.84 (J = 5.5 Hz)	t 8.21 (J = 5.1 Hz)	m 8.21		
**H-22**	d 8.10 (J = 5.5 Hz)	d 8.71 (J = 5.1 Hz)	d 8.70 (J = 5.1 Hz)		s 2.46
**NH**	bs 12.37	bs 12.45	---	s 12.78	---
**OH-3**	---	bs 18.82	bs 18.76	bs 18.78	bs 18.82
**OH-7**	s 13.28	s 13.05	s 12.96	s 12.49	s 12.27
**OH-9**	---	bs 10.30	bs 10.30	s 10.23	s 10.27

**Table 8:** NMR ^13^C spectra of **16a**–**16e** (DMSO-*d6*, δ).

№	16a	16b	16c	16d	16e
**Structure**	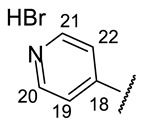	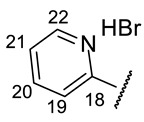	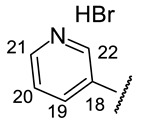	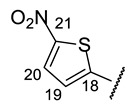	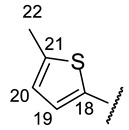
**C-17**	137.15	146.33	137.05	135.56	138.62
**C-18**	148.81	150.12	133.07	146.60	136.51
**C-19**	122.41	124.82	141.66	130.58	130.36
**C-20**	143.16	138.72	143.08	128.13	126.38
**C-21**	143.16	121.21	126.76	149.84	142.34
**C-22**	122.41	140.70	139.95		15.35

**Table 9:** NMR^1^H spectra of **16f–16j** (CDCl_3_, δ).

№	16f	16g	16h	16i	16j
**Structure**	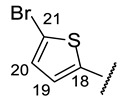	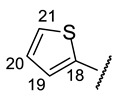	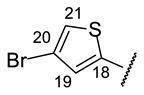	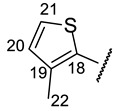	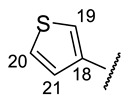
**H-14**	s 7.11	s 7.08	s 7.04	s 7.09	s 7.12
**H-17**	s 7.58	s 7.81	bs 8.12	s 7.73	s 7.78
**H-19**	d 6.77 (J = 3.8 Hz)	d 7.08 (J = 3.4 Hz)	s 7.04		s 7.43
**H-20**	d 6.86 (J = 3.8 Hz)	m 6.94		d 6.72 (J = 5.3 Hz)	d 7.46 (J = 4.9 Hz)
**H-21**		d 7.27 (J = 5.0 Hz)	s 7.19	d 7.14 (J = 4.9 Hz)	m 7.31
**H-22**				s 2.23	
**NH**	bs 9.06	bs 9.48	---	bs 9.11	bs 8.99
**OH-3**	s 18.79	s 18.78	s 18.79	s 18.78	s 18.79
**OH-7**	---	---	---	---	---
**OH-9**	s 10.29	s 10.27	s 10.40	s 10.26	s 10.28

**Table 10:** NMR ^13^C spectra of **16f**–**16j** (CDCl_3_, δ).

№	16f	16g	16h	16i	16j
**Structure**	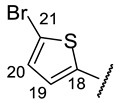	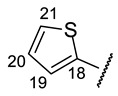	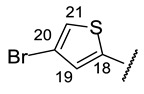	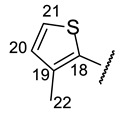	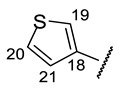
**C-17**	135.97	142.67	138.68	136.47	137.90
**C-18**	139.39	138.12	138.72	131.71	136.51
**C-19**	128.64	137.70	131.84	138.95	124.77
**C-20**	129.92	127.87	110.30	126.73	126.09
**C-21**	115.29	129.21	125.35	130.60	126.59
**C-22**				13.95	

**Table 11:** NMR^1^H spectra of **16k**–**16o** (CDCl_3_ for 16k,l,n and DMSO-*d6* for 16m,o; δ).

№	16k	16l	16m	16n	16o
**Structure**	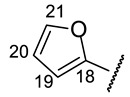	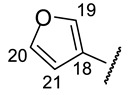	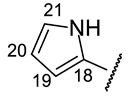	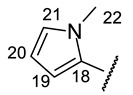	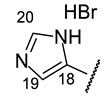
**H-14**	s 7.15	s 7.11	s 7.24	s 7.09	s 7.37
**H-17**	s 7.60	s 7.63	s 7.94	s 7.68	s 8.09
**H-19**	d 6.65 (J = 2.5 Hz)	s 7.59	s 6.46	m 6.39	s 8.00
**H-20**	m 6.45	s 7.39	s 6.14	m 6.11	s 9.17
**H-21**	d 7.49 (J = 2.2 Hz)	m 6.75	s 6.92	s 6.72	
**H-22**				s 3.91	
**NH**	s 8.88	bs 8.93	---	bs 8.68	---
**NH (HetAr)**			s 11.30		s 12.60
**OH-3**	s 18.77	s 18.79	bs 18.77	s 18.79	---
**OH-7**	bs 12.40	---	s 12.05	---	s 12.71
**OH-9**	s 10.26	s 10.95	s 10.28	s 10.28	s 10.31

**Table 12:** NMR ^13^C spectra of **16k**–**16o** (CDCl_3_ for 16k,l,n and DMSO-*d6* for 16m,o; δ).

№	16k	16l	16m	16n	16o
**Structure**	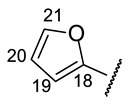	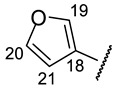	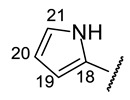	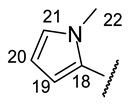	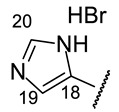
**C-17**	132.37	135.09	136.59	136.37	130.90
**C-18**	148.62	121.97	126.80	126.39	128.58
**C-19**	112.28	143.42	109.33	115.74	119.55
**C-20**	111.72	144.00	112.13	108.03	132.36
**C-21**	144.26	107.19	122.14	128.01	
**C-22**				37.09	

**Table 13:** NMR^1^H spectra of **16p**–**16r** (CDCl_3_ for 16o and DMSO-*d6* for 16p,q; δ).

№	16p	16q	16r
**Structure**	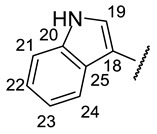	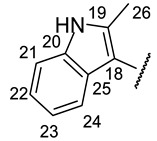	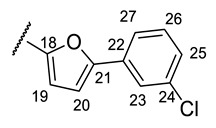
**H-14**	s 7.24	s 7.22	s 7.12
**H-17**	s 8.30	s 8.33	s 7.68
**H-19**	m 8.21		d 6.69 (J = 3.3 Hz, AB-system)
**H-20**			d 6.66 (J = 3.3 Hz, AB-system)
**H-21**	s 7.45	m 7.34	
**H-22**	m 7.21	m 7.13	
**H-23**	m 7.21	m 7.13	s 7.62
**H-24**	d 7.83 (J = 2.7 Hz)	m 8.10	
**H-25**			d 7.52 (J = 7.5 Hz)
**H-26**		s 2.51	m 7.21–7.30
**H-27**			m 7.21–7.30
**NH**	s 12.08	s 12.00	---
**NH (HetAr)**	s 11.59	s 11.51	
**OH-3**	bs 18.82	bs 18.82	s 18.76
**OH-7**	s 13.03	bs 13.05	---
**OH-9**	s 10.30	s 10.31	s 10.26

**Table 14:** NMR ^13^C spectra of **16p**–**16r** (CDCl_3_ for 16o and DMSO-*d6* for 16p,q; δ).

№	16n	16o	16r
**Structure**	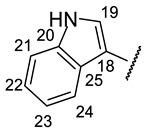	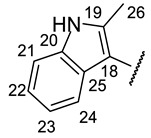	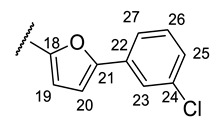
**C-17**	140.39	140.29	132.95
**C-18**	111.35	107.42	148.80
**C-19**	130.13	135.76	108.17
**C-20**	137.11	139.52	114.29
**C-21**	111.95	110.99	153.82
**C-22**	121.59	120.63	131.35
**C-23**	120.59	120.46	123.92
**C-24**	122.64	121.83	134.78
**C-25**	124.02	124.02	127.90
**C-26**		11.53	129.91
**C-27**			122.03

**Table 15:**
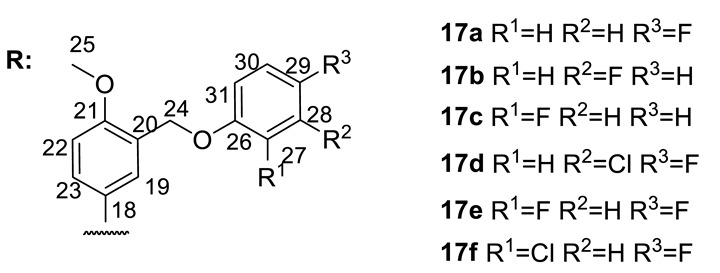
NMR^1^H spectra of **17a**–**17f** (CDCl_3_, δ).

№	17a	17b	17c	17d	17e	17f
H-14	s 7.09	s 7.02	m 7.05	s 7.06	s 7.09	s 7.07
H-17	m 7.45–7.65	s 7.81	s 7.68	s 7.64	s 7.60	s 7.85
H-19	m 7.45–7.65	s 7.71	s 7.62	s 7.61	s 7.56	s 7.71
H-22	m 6.82–6.97	d 6.77 (J = 8.6 Hz)	d 6.78 (J = 7.0 Hz)	m 6.82	d 6.51 (J = 7.9 Hz)	m 6.90
H-23	m 7.45–7.65	d 7.44 (J = 8.6 Hz)	d 7.44 (J = 7.0 Hz)	d 7.48 (J = 7.3 Hz)	d 7.48 (J = 8.3 Hz)	d 7.41 (J = 8.1 Hz)
H-24	s 5.00	s 5.07	s 5.09	s 4.97	s 5.00	s 5.04
H-25	s 3.83	s 3.79	s 3.79	s 3.82	s 3.84	s 3.83
H-27	m 6.82–6.97	m 6.90–7.10	s 6.91	m 7.01		d 8.47 (J = 8.5 Hz)
H-28	m 6.82–6.97	m 6.90–7.10	m 7.05		d 6.82 (J = 7.9 Hz)	m 6.90
H-29		m 6.90–7.10	m 7.05			
H-30	m 6.82–6.97		m 7.05	m 7.01	t 6.41 (J = 8.4 Hz)	m 7.12
H-31	m 6.82–6.97	m 6.90–7.10		m 6.82		
NH	bs 8.92	---	bs 9.45	bs 9.45	bs 8.93	bs 9.60
OH-3	s 18.78	s 18.77	s 18.76	s 18.77	s 18.78	s 18.75
OH-7	---	---	---	---	bs 12.21	---
OH-9	s 10.24	s 10.29	s 10.23	s 10.24	s 10.23	s 10.22

**Table 16:** NMR ^13^C spectra of **17a**–**17f** (CDCl_3_, δ).

№	17a	17b	17c	17d	17e	17f
C-17	142.43	143.82	143.09	142.48	142.30	142.35
C-18	125.64	125.43	125.30	125.01	124.84	125.12
C-19	127.14	127.14	127.13	127.07	127.11	127.14
C-20	126.07	125.93	126.00	126.04	126.20	126.28
C-21	158.06	158.19	158.08	158.03	158.10	158.05
C-22	110.26	110.21	110.14	110.25	110.41	110.34
C-23	127.69	128.96	127.93	127.96	128.06	128.65
C-24	65.26	65.91	65.88	65.32	65.16	65.22
C-25	55.47	55.46	55.36	55.46	55.53	55.78
C-26	d 154.74 (J = 2.2 Hz)	d 146.61 (J = 10 Hz)	d 146.61 (J = 11 Hz)	d 154.81 (J = 2.4 Hz)	t 160.57 (J = 13.4 Hz)	d 150.14 (J = 2.4 Hz)
C-27	d 115.56 (J = 12 Hz)	115.50	d 151.42 (J = 247 Hz)	116.44	d 162.68 (J = 244 Hz)	d 121.50 (J = 10 Hz)
C-28	d 115.83 (J = 3.2 Hz)	d 151.84 (J = 249 Hz)	d 116 (J = 16 Hz)	120.83 (J = 19 Hz)	t 96.47 (J = 29 Hz)	d 118.51 (J = 28 Hz)
C-29	d 155.68 (J = 239 Hz)	d 116.00 (J = 19 Hz)	d 121.24 (J = 7 Hz)	d 151.54 (J = 247 Hz)	d 162.55 (J = 244 Hz)	d 156.08 (J = 247 Hz)
C-30	d 115.83 (J = 3.2 Hz)	d 124.08 (J = 3.5 Hz)	d 124.10 (J = 3.4 Hz)	d 116.51 (J = 22 Hz)	m 98.50	d 114.06 (J = 21 Hz)
C-31	d 115.56 (J = 12 Hz)	d 121.25 (J = 8 Hz)	115.41	d 114.30 (J = 6.7 Hz)	m 98.50	d 114.67 (J = 9.4 Hz)

**Table 17:**
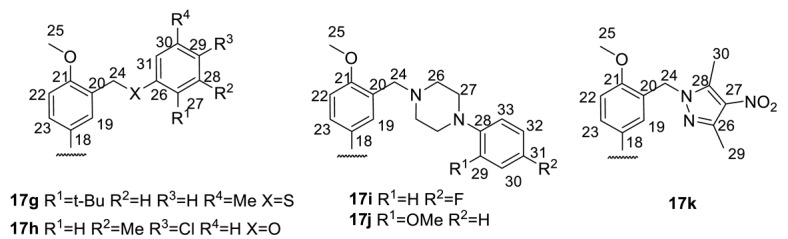
NMR^1^H spectra of **17g**–**17k** (CDCl_3_ for g–j and DMSO-*d6* for k, δ).

№	17g	17h	17i	17j	17k
H-14	s 7.08	s 7.07	s 7.00	s 6.98	s 7.23
H-17	s 7.49	s 7.69	bs 7.98	s 8.13	s 7.26
H-19	s 7.32	s 7.64	bs 7.98	s 8.05	s 7.67
H-22	d 6.76 (J = 8.5 Hz)	m 6.76	d 6.56 (J = 8.0 Hz)	d 6.46 (J = 8.1 Hz)	d 7.08 (J = 8.4 Hz)
H-23	m 7.40	d 7.51 (J = 8.3 Hz)	m 7.34	m 6.84-7.03	d 7.50 (J = 8.4 Hz)
H-24	s 4.04	s 4.99	s 4.11	s 4.19	s 5.27
H-25	s 3.77	s 3.84	s 3.61	bs 3.51	s 3.85
H-26			s 3.29	bs 3.51	
H-27	s 1.22 (t-Bu)	d 6.88 (J = 2.4 Hz)	s 3.48	m 3.85	
H-28	m 7.23	s 2.34 (Me)			
H-29	m 7.08		m 6.83–6.94	m 6.84–7.03	s 2.40
H-30	s 2.33 (Me)	d 7.21 (J = 8.6 Hz)	m 6.83–6.94	m 3.85 (OMe)	s 2.59
H-31	m 7.32	d 6.83 (J = 8.5 Hz)		m 6.84–7.03	
H-32			m 6.83–6.94	m 6.84–7.03	
H-33			m 6.83–6.94	m 6.84–7.03	
NH	bs 9.09	bs 9.56	bs 11.22	bs 11.22	bs 12.28
OH-3	s 18.79	s 18.79	bs 18.7	bs 18.76	bs 18.80
OH-7	---	---	bs 12.64	bs 12.63	bs 12.77
OH-9	s 10.26	s 10.27	s 10.24	s 10.23	s 10.30

**Table 18:** NMR ^13^C spectra of **17g**–**17k** (CDCl_3_ for g–j and DMSO-*d6* for k, δ).

№	17g	17h	17i	17j	17k
C-17	142.74	143.61	141.77	141.03	142.40
C-18	125.70	125.47	127.27	127.72	123.52
C-19	127.77	127.19	129.01	129.77	126.09
C-20	126.47	125.90	127.27	127.72	126.99
C-21	158.61	158.08	158.70	158.36	157.16
C-22	110.38	110.21	110.52	110.89	110.26
C-23	128.79	129.46	131.17	131.96	128.05
C-24	32.56	64.74	48.25	47.48	47.97
C-25	55.50	55.45	55.35	55.26	55.41
C-26	134.44	157.12	54.52	54.64	140.77
C-27	149.06 (C_Ar_) 31.09 (t-Bu)	113.28	51.98	52.05	145.97
C-28	129.57	127.92 20.25 (Me)	146.65	151.77	130.90
C-29	128.79	136.97	d 115.45 (J = 22 Hz)	139.11 (C_Ar_) 54.96 (O-Me)	11.32
C-30	135.72 (C_Ar_) 19.73 (Me)	126.01	d 118.53 (J = 7.43 Hz)	110.89	14.07
C-31	123.63	117.22	d 156.02 (J = 240 Hz)	118.75	
C-32			d 118.53 (J = 7.43 Hz)	124.02	
C-33			d 115.45 (J = 22 Hz)	121.04	

## Supplementary Materials

The following are available online. Tables S1–S16 (NMR data), Copies of NMR spectra of the products, Figures with changes in intrinsic fluorescence intensity of Tdp1 upon the addition of compounds, **Table S17**: RMSD values of for heavy atoms between the co-crystallized benzene-1,2,4-tricarboxylic acid (6DIE) and the docked molecule, **Table S18**: Scores of the scoring function from the docked ligands with and without water, **Table S19**: The calculated molecular descriptors for the ligands, **Table S20**: Definition of lead-like, drug-like and Known drug space (KDS) in terms of molecular descriptors. The values given are the maxima for each descriptor for the volumes of chemical space used, Table S21: Known drug index calculated.
