# Supplementary material for: New Hydrazinothiazole Derivatives of Usnic Acid as Potent Tdp1 Inhibitors
Source: Molecules. 2019 Oct 15;24(20):3711. doi: 10.3390/molecules24203711 (PMC6832265; doi:10.3390/molecules24203711)
Supplement: Supplementary file 1 [file molecules-24-03711-s001.pdf]

Supplementary Material

New Hydrazinothiazole Derivatives of Usnic Acid as Potent Tdp1 Inhibitors

Aleksander S. Filimonov [1,3], Arina A. Chepanova [2], Olga A. Luzina [1], Alexandra L. Zakharenko [2], Olga D. Zakharova [2], Ekaterina S. Ilina [2], Nadezhda S. Dyrkheeva [2], Maxim S. Kuprushkin [2], Anton V. Kolotaev [4], Derenik S. Khachatryan [4], Jinal Patel [5], Ivanhoe K.H. Leung [5], Raina Chand [5], Daniel M. Ayine-Tora [5], Johannes Reynisson [6], Konstantin P. Volcho [1,3,*], Nariman F. Salakhutdinov [1,3] and Olga I. Lavrik [1,3]

[1] N. N. Vorozhtsov Novosibirsk Institute of Organic Chemistry, Siberian Branch of the Russian Academy of Sciences, 9, Akademika Lavrentieva Ave., Novosibirsk 630090, Russian Federation

[2] Novosibirsk Institute of Chemical Biology and Fundamental Medicine, Siberian Branch of the Russian Academy of Sciences, 8, Akademika Lavrentieva Ave., Novosibirsk 630090, Russian Federation

[3] Novosibirsk State University, Pirogova str. 1, Novosibirsk 630090, Russian Federation

[4] The Federal State Unitary Enterprise, Institute of Chemical Reagents and High Purity Chemical Substances of National Research Centre, Kurchatov Institute, Moscow 107076, Russia

[5] School of Chemical Sciences, The University of Auckland, New Zealand

[6] School of Pharmacy and Bioengineering, Keele University, Hornbeam Building, Staffordshire ST5 5BG, UK

* Correspondence: volcho@nioch.nsc.ru (V.K.P.), lavrik@niboch.nsc.ru (O.I.L.); Tel.: +7-383-3308870 (V.K.P.); + 7-383-3635195 (O.I.L.)

Content

1. Tables S1-S16
2. NMR spectra of the products
3. Changes in intrinsic fluorescence intensity of Tdp1 (10 μM) upon the addition of compounds
4. Tables S17-S21

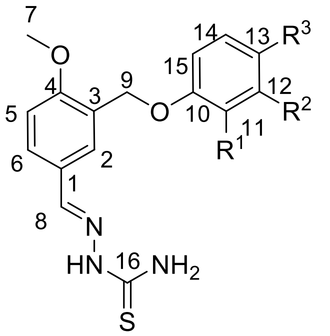

12a R^1=H R^2=H R^3=F
12b R^1=H R^2=F R^3=H
12c R^1=F R^2=H R^3=H
12d R^1=H R^2=Cl R^3=F
12e R^1=F R^2=H R^3=F
12f R^1=Cl R^2=H R^3=F

Table S1: Spectra NMR^{1}H of **12a-12f** (DMSO-d_6, δ):

№	12a	12b	12c	12d	12e	12f
H-2	s 8.00	s 8.01	s 8.01	s 8.00	s 8.01	s 8.01
H-5	m 7.00-7.15	m 7.08-7.30	d 7.09 (J=8.7 Hz)	d 7.07 (J=8.6 Hz)	d 7.01 (J=8.6 Hz)	d 7.01 (J=8.6 Hz)
H-6	m 7.72	m 7.75	m 7.77	m 7.72	m 7.75	m 7.77
H-7	s 3.86	s 3.86	s 3.85	s 3.85	s 3.86	s 3.86
H-8	s 8.12	s 8.13	s 8.16	s 8.14	s 8.13	s 8.15
H-9	s 5.00	s 5.09	s 5.09	s 5.02	s 5.04	s 5.09
H-11	m 7.00-7.15	m 7.08-7.30		t 7.35 (J=9.1 Hz)		
H-12	m 7.00-7.15		m 7.21		m 6.77	m 7.42
H-13		m 7.08-7.30	m 7.28			
H-14	m 7.00-7.15	m 7.08-7.30	t 7.11 (J=7.7 Hz)	m 7.27	m 6.77	m 7.28
H-15	m 7.00-7.15	m 6.95	m 6.95	m 7.02	m 6.77	m 7.17
NH	s 11.32	s 11.33	s 11.35	s 11.34	s 11.33	s 11.33
NH_2	m 7.90	m 7.89	m 7.90	m 7.92	m 7.93	m 7.87

Table S2: Spectra NMR ^{13}C of **12a-12f** (DMSO-d_6, δ):

№	12a	12b	12c	12d	12e	5f
C-1	125.39	124.99	124.57	124.94	124.53	124.93
C-2	129.11	129.54	129.12	129.27	129.57	129.20
C-3	126.95	126.97	126.56	126.99	127.01	126.98
C-4	159.03	159.18	158.78	159.07	159.17	159.10
C-5	111.60	111.74	111.32	111.65	111.68	111.78
C-6	129.85	129.99	129.57	130.08	130.30	129.83
C-7	56.23	56.27	55.86	56.25	56.26	56.29
C-8	142.52	142.41	141.99	142.42	142.33	142.44
C-9	65.62	66.17	65.75	66.03	66.12	66.78
C-10	155.33	d 146.83 (J=10 Hz)	d 146.42 (J=11 Hz)	d 155.62 (J=2.4 Hz)	t 161.17 (J=14 Hz)	d 151.14 (J=2.4 Hz)
C-11	d 116.37(J=4 Hz)	115.69	d 150.60 (J=248 Hz)	d 115.53 (J=7 Hz)	d 162.40 (J=244 Hz)	d 122.50 (J= 10Hz)
C-12	d 116.19(J=11 Hz)	d151 (J=244 Hz)	d 115.96(J=18 Hz)	d 120.08 (J=17 Hz)	t 96.65 (J=27 Hz)	d 117.51 (J=28 Hz)
C-13	d 155.83(J=240 Hz)	d 116.38(J=18 Hz)	d 121.21 (J=7 Hz)	d 151.12 (J=247 Hz)	d 162.25 (J=244 Hz)	d 155.08 (J=247 Hz)
C-14	d 116.19(J=11 Hz)	d 125.30 (J=3 Hz)	d 124.88 (J=3 Hz)	d 117.56 (J=22 Hz)	m 99.15	d 115.01 (J=21 Hz)
C-15	d 116.37(J=4 Hz)	d 121.63 (J=7 Hz)	115.27	116.63	m 99.43	d 115.54 (J=9.4 Hz)
C-16	178.04	178.06	177.64	178.06	178.07	178.09

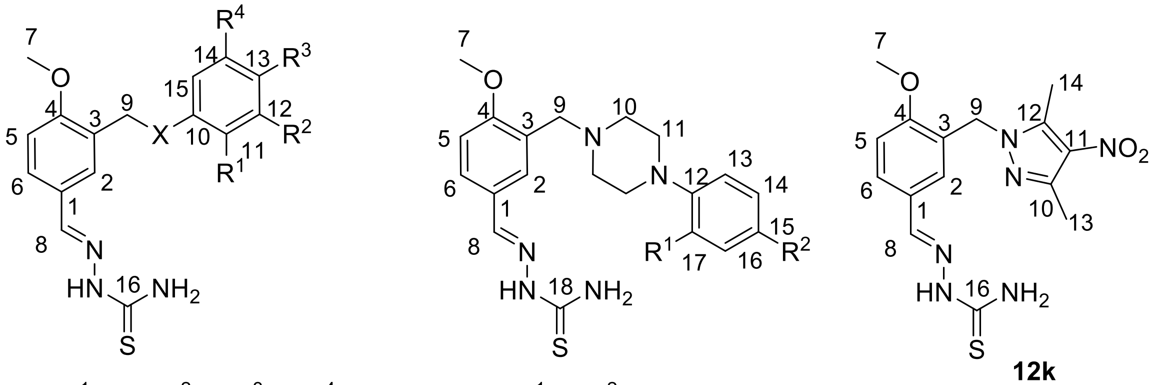

12k

12g R^1=t-Bu R^2=H R^3=H R^4=Me X=S **12i** R^1=H R^2=F

12h R^1=H R^2=Me R^3=Cl R^4=H X=O **12j** R^1=OMe R^2=H

Table S3: Spectra NMR^{1}H of **12f-12k** (DMSO-d$_6$, δ):

№	12g	12h	12i	12j	12k
H-2	s 7.91	s 7.99	s 8.00	s 8.00	s 7.89
H-5	d 7.00 (J=9.3 Hz)	m 7.04	m 7.02	d 7.05 (J=8.5 Hz)	d 7.03 (J=8.5 Hz)
H-6	m 7.62	m 7.72	m 7.69	bs 7.75	m 7.63
H-7	s 3.80	s 3.85	s 3.83	s 3.83	s 3.81
H-8	s 8.14	s 8.14	s 8.11	s 8.15	s 7.97
H-9	s 4.09	s 5.00	s 3.55	s 3.68	s 5.21
H-10			bs 2.57	bs 2.69	
H-11	s 1.19 (t-Bu)		bs 3.07	bs 2.93	
H-12	s 7.09	s 2.28 (Me)			
H-13	s 7.18	m 7.06	m 6.90	m 6.87-6.94	2.34
H-14	s 2.21 (Me)	m 7.28	m 7.02	m 6.87-6.94	2.67
H-15	s 7.09	m 6.86		m 6.87-6.94	
H-16			m 7.02	m 6.87-6.94	
H-17			m 6.90	s 3.74 (OMe)	
NH	s 11.32	s 11.33	s 11.29	s 11.34	s 11.33
NH_2	s 7.62 and s 7.80	s 7.89 and s 7.93	m 7.69 and s 7.85	bs 7.72 and bs 7.90	s 7.75 and s 7.78

Table S4: Spectra NMR ^{13}C of **12f-12k** (DMSO-d_6, δ):

№	12g	12h	12i	12j	12k
C-1	125.87	124.88	127.95	126.36	123.74
C-2	129.13	129.36	126.19	128.64	129.05
C-3	126.31	126.52	127.95	126.36	126.59
C-4	158.47	158.57	159.03	159.28	158.26
C-5	111.11	111.19	111.16	111.38	111.30
C-6	129.59	129.50	129.64	130.46	129.37
C-7	55.77	55.80	55.67	55.89	55.83
C-8	142.02	142.12	142.24	142.42	141.87
C-9	31.52	64.84	48.73	49.30	48.10
C-10	134.24	157.29	55.06	55.05	145.04
C-11	148.8 (C, Ar)<br>30.88 (Me t-Bu)<br>34.14 (C, t-Bu)	117.36	52.36	52.55	130.02
C-12	128.25	128.60 (C, Ar)<br>19.79 (Me)	147.75	152.02	141.38
C-13	125.88	136.52	117.02<br>(J=7 Hz)	111.92	11.39
C-14	134.58 (C, Ar)<br>19.32 (Me)	124.70	115.06<br>(J=22 Hz)	118.13	13.90
C-15	123.05	113.82	d 155.00<br>(J=232 Hz)	122.77	
C-16	177.58	177.66	115.06<br>(J=22 Hz)	120.92	177.67
C-17			117.02<br>(J=7 Hz)	140.08	
C-18			177.60	177.64	

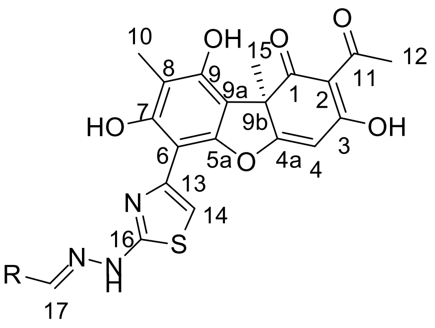

Spectra NMR ^{1}H (**$CDCl_3$**, δ): 1.67 (3H, s, H-15), 2.13 (3H, s, H-10), 2.61 (3H, s, H-12), 5.88 (1H, s, H-4). Spectra NMR ^{13}C (**$CDCl_3$**, δ): 8.3 (C-10), 27.6 (C-12), 32.0 (C-15), 59.3 (C-9b), 97.2 (C-4), 97.3 (C-9a), 103.3 (C-6), 104.4 (C-14), 105.0 (C-2), 108.8 (C-8), 143.4 (C-13), 151.3 (C-7), 151.4 (C-9), 156.3 (C-5a), 166.4 (C-16), 180.4 (C-4a), 191.5 (C-3), 197.9 (C-1), 201.2 (C-11).

Spectra NMR ^{1}H (**DMSO-d_6**, δ): 1.70 (3H, s, H-15), 2.03 (3H, s, H-10), 2.60 (3H, s, H-12), 6.20 (1H, s, H-4). Spectra NMR ^{13}C (**DMSO-d_6**, δ): 8.3 (C-10), 27.6 (C-12), 32.0 (C-15), 59.0 (C-9b), 96.9 (C-9a),97.3 (C-4), 103.3 (C-6),105.1 (C-2), 105.6 (C-14), 107.3 (C-8), 143.0 (C-13), 151.3 (C-7), 151.4 (C-9), 156.3 (C-5a), 166.4 (C-16), 180.4 (C-4a), 191.5 (C-3), 197.9 (C-1), 201.2 (C-11).

Table S5: Spectra NMR^{1}H of **16a-16e** (DMSO-d_6, δ):

**№**	**16a**	**16b**	**16c**	**16d**	**16e**
**Structure**	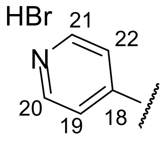	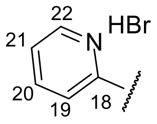	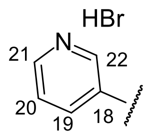	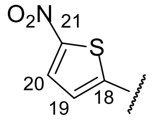	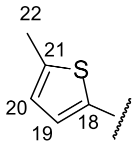
**H-14**	s 7.39	s 7.39	s 7.38	s 7.27	s 7.23
**H-17**	s 8.16	s 8.15	s 8.22	s 8.08	s 8.16
**H-19**	d 8.10 (J=5.5 Hz)	d 8.07 (J=8.0 Hz)	s 9.13	d 7.34 (J=4.5 Hz)	d 7.19 (J=4.9 Hz)
**H-20**	d 8.84 (J=5.5 Hz)	t 7.66 (J=5.9 Hz)	d 8.85 (J=5.1 Hz)	d 7.98 (J=4.5 Hz)	m 6.79
**H-21**	d 8.84 (J=5.5 Hz)	t 8.21 (J=5.1 Hz)	m 8.21		
**H-22**	d 8.10 (J=5.5 Hz)	d 8.71 (J=5.1 Hz)	d 8.70 (J=5.1 Hz)		s 2.46
**NH**	bs 12.37	bs 12.45	---	s 12.78	---
**OH-3**	---	bs 18.82	bs 18.76	bs 18.78	bs 18.82
**OH-7**	s 13.28	s 13.05	s 12.96	s 12.49	s 12.27
**OH-9**	---	bs 10.30	bs 10.30	s 10.23	s 10.27

Table S6: Spectra NMR ^{13}C of **16a-16e** (DMSO-d_6, δ):

**№**	**16a**	**16b**	**16c**	**16d**	**16e**
**Structure**	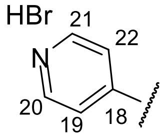	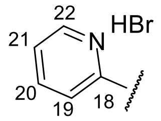	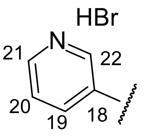	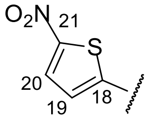	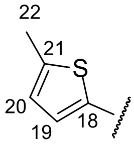
**C-17**	137.15	146.33	137.05	135.56	138.62
**C-18**	148.81	150.12	133.07	146.60	136.51
**C-19**	122.41	124.82	141.66	130.58	130.36
**C-20**	143.16	138.72	143.08	128.13	126.38
**C-21**	143.16	121.21	126.76	149.84	142.34
**C-22**	122.41	140.70	139.95		15.35

Table S7: Spectra NMR^{1}H of **16f-16j** ($CDCl_3$, δ):

**№**	**16f**	**16g**	**16h**	**16i**	**16j**
**Structure**	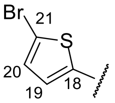	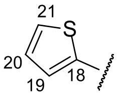	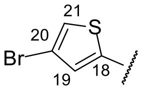	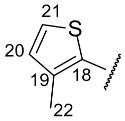	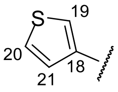
**H-14**	s 7.11	s 7.08	s 7.04	s 7.09	s 7.12
**H-17**	s 7.58	s 7.81	bs 8.12	s 7.73	s 7.78
**H-19**	d 6.77 (J=3.8 Hz)	d 7.08 (J=3.4 Hz)	s 7.04		s 7.43
**H-20**	d 6.86 (J=3.8 Hz)	m 6.94		d 6.72 (J=5.3 Hz)	d 7.46 (J=4.9 Hz)
**H-21**		d 7.27 (J=5.0 Hz)	s 7.19	d 7.14 (J=4.9 Hz)	m 7.31
**H-22**				s 2.23	
**NH**	bs 9.06	bs 9.48	---	bs 9.11	bs 8.99
**OH-3**	s 18.79	s 18.78	s 18.79	s 18.78	s 18.79
**OH-7**	---	---	---	---	---
**OH-9**	s 10.29	s 10.27	s 10.40	s 10.26	s 10.28

Table S8: Spectra NMR ^{13}C of **16f-16j** ($CDCl_3$, δ):

**№**	**16f**	**16g**	**16h**	**16i**	**16j**
**Structure**	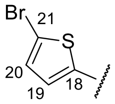	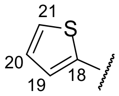	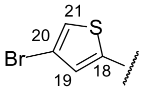	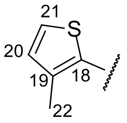	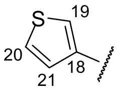
**C-17**	135.97	142.67	138.68	136.47	137.90
**C-18**	139.39	138.12	138.72	131.71	136.51
**C-19**	128.64	137.70	131.84	138.95	124.77
**C-20**	129.92	127.87	110.30	126.73	126.09
**C-21**	115.29	129.21	125.35	130.60	126.59
**C-22**				13.95	

Table S9: Spectra NMR^{1}H of **16k-16o** ($CDCl_3$ for 16k,l,n and DMSO-d_6 for 16m,o; δ):

**№**	**16k**	**16l**	**16m**	**16n**	**16o**
**Structure**	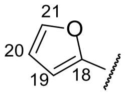	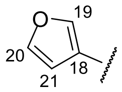	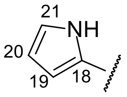	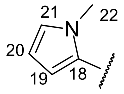	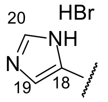
**H-14**	s 7.15	s 7.11	s 7.24	s 7.09	s 7.37
**H-17**	s 7.60	s 7.63	s 7.94	s 7.68	s 8.09
**H-19**	d 6.65 (J=2.5 Hz)	s 7.59	s 6.46	m 6.39	s 8.00
**H-20**	m 6.45	s 7.39	s 6.14	m 6.11	s 9.17
**H-21**	d 7.49 (J=2.2 Hz)	m 6.75	s 6.92	s 6.72	
**H-22**				s 3.91	
**NH**	s 8.88	bs 8.93	---	bs 8.68	---
**NH (HetAr)**			s 11.30		s 12.60
**OH-3**	s 18.77	s 18.79	bs 18.77	s 18.79	---
**OH-7**	bs 12.40	---	s 12.05	---	s 12.71
**OH-9**	s 10.26	s 10.95	s 10.28	s 10.28	s 10.31

Table S10: Spectra NMR ^{13}C of **16k-16o** ($CDCl_3$ for 16k,l,n and DMSO-d_6 for 16m,o; δ):

**№**	**16k**	**16l**	**16m**	**16n**	**16o**
**Structure**	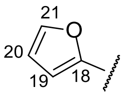	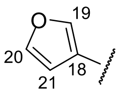	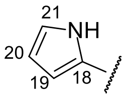	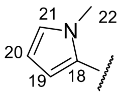	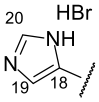
**C-17**	132.37	135.09	136.59	136.37	130.90
**C-18**	148.62	121.97	126.80	126.39	128.58
**C-19**	112.28	143.42	109.33	115.74	119.55
**C-20**	111.72	144.00	112.13	108.03	132.36
**C-21**	144.26	107.19	122.14	128.01	
**C-22**				37.09	

Table S11: Spectra NMR^{1}H of **16p-16r** ($CDCl_3$ for 16o and DMSO-d_6 for 16p,q; δ):

№	16p	16q	16r
**Structure**	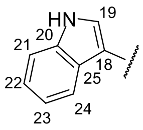	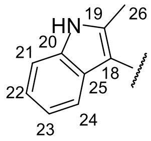	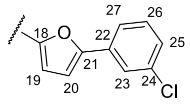
**H-14**	s 7.24	s 7.22	s 7.12
**H-17**	s 8.30	s 8.33	s 7.68
**H-19**	m 8.21		d 6.69 (J=3.3 Hz, AB-system)
**H-20**			d 6.66 (J=3.3 Hz, AB-system)
**H-21**	s 7.45	m 7.34	
**H-22**	m 7.21	m 7.13	
**H-23**	m 7.21	m 7.13	s 7.62
**H-24**	d 7.83 (J=2.7 Hz)	m 8.10	
**H-25**			d 7.52 (J=7.5 Hz)
**H-26**		s 2.51	m 7.21-7.30
**H-27**			m 7.21-7.30
**NH**	s 12.08	s 12.00	---
**NH (HetAr)**	s 11.59	s 11.51	
**OH-3**	bs 18.82	bs 18.82	s 18.76
**OH-7**	s 13.03	bs 13.05	---
**OH-9**	s 10.30	s 10.31	s 10.26

Table S12: Spectra NMR ^{13}C of **16p-16r** ($CDCl_3$ for 16o and DMSO-d_6 for 16p,q; δ):

№	16n	16o	16r
**Structure**	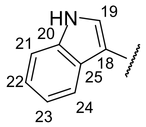	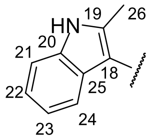	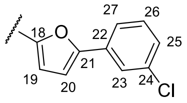
**C-17**	140.39	140.29	132.95
**C-18**	111.35	107.42	148.80
**C-19**	130.13	135.76	108.17
**C-20**	137.11	139.52	114.29
**C-21**	111.95	110.99	153.82
**C-22**	121.59	120.63	131.35
**C-23**	120.59	120.46	123.92
**C-24**	122.64	121.83	134.78
**C-25**	124.02	124.02	127.90
**C-26**		11.53	129.91
**C-27**			122.03

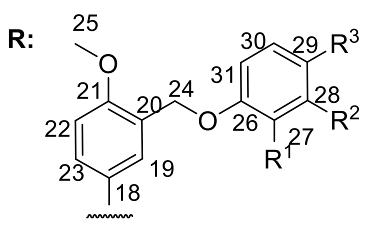

17a R^1=H R^2=H R^3=F
17b R^1=H R^2=F R^3=H
17c R^1=F R^2=H R^3=H
17d R^1=H R^2=Cl R^3=F
17e R^1=F R^2=H R^3=F
17f R^1=Cl R^2=H R^3=F

Table S13: Spectra NMR^{1}H of **17a-17f** ($CDCl_3$, δ):

№	17a	17b	17c	17d	17e	17f
H-14	s 7.09	s 7.02	m 7.05	s 7.06	s 7.09	s 7.07
H-17	m 7.45-7.65	s 7.81	s 7.68	s 7.64	s 7.60	s 7.85
H-19	m 7.45-7.65	s 7.71	s 7.62	s 7.61	s 7.56	s 7.71
H-22	m 6.82-6.97	d 6.77 (J=8.6 Hz)	d 6.78 (J=7.0 Hz)	m 6.82	d 6.51 (J=7.9 Hz)	m 6.90
H-23	m 7.45-7.65	d 7.44 (J=8.6 Hz)	d 7.44 (J=7.0 Hz)	d 7.48 (J=7.3 Hz)	d 7.48 (J=8.3 Hz)	d 7.41 (J=8.1 Hz)
H-24	s 5.00	s 5.07	s 5.09	s 4.97	s 5.00	s 5.04
H-25	s 3.83	s 3.79	s 3.79	s 3.82	s 3.84	s 3.83
H-27	m 6.82-6.97	m 6.90-7.10	s 6.91	m 7.01		d 8.47 (J=8.5 Hz)
H-28	m 6.82-6.97	m 6.90-7.10	m 7.05		d 6.82 (J=7.9 Hz)	m 6.90
H-29		m 6.90-7.10	m 7.05			
H-30	m 6.82-6.97		m 7.05	m 7.01	t 6.41 (J=8.4 Hz)	m 7.12
H-31	m 6.82-6.97	m 6.90-7.10		m 6.82		
NH	bs 8.92	---	bs 9.45	bs 9.45	bs 8.93	bs 9.60
OH-3	s 18.78	s 18.77	s 18.76	s 18.77	s 18.78	s 18.75
OH-7	---	---	---	---	bs 12.21	---
OH-9	s 10.24	s 10.29	s 10.23	s 10.24	s 10.23	s 10.22

Table S14: Spectra NMR ^{13}C of **17a-17f** ($CDCl_3$, δ):

№	**17a**	**17b**	**17c**	**17d**	**17e**	**17f**
C-17	142.43	143.82	143.09	142.48	142.30	142.35
C-18	125.64	125.43	125.30	125.01	124.84	125.12
C-19	127.14	127.14	127.13	127.07	127.11	127.14
C-20	126.07	125.93	126.00	126.04	126.20	126.28
C-21	158.06	158.19	158.08	158.03	158.10	158.05
C-22	110.26	110.21	110.14	110.25	110.41	110.34
C-23	127.69	128.96	127.93	127.96	128.06	128.65
C-24	65.26	65.91	65.88	65.32	65.16	65.22
C-25	55.47	55.46	55.36	55.46	55.53	55.78
C-26	d 154.74 (J=2.2 Hz)	d 146.61 (J=10 Hz)	d 146.61 (J=11 Hz)	d 154.81 (J=2.4 Hz)	t 160.57 (J=13.4 Hz)	d 150.14 (J=2.4 Hz)
C-27	d 115.56 (J=12 Hz)	115.50	d 151.42 (J=247 Hz)	116.44	d 162.68 (J=244 Hz)	d 121.50 (J= 10Hz)
C-28	d 115.83 (J=3.2 Hz)	d 151.84 (J=249 Hz)	d 116 (J=16 Hz)	120.83 (J=19 Hz)	t 96.47 (J=29 Hz)	d 118.51 (J=28 Hz)
C-29	d 155.68 (J=239 Hz)	d 116.00 (J=19 Hz)	d 121.24 (J=7 Hz)	d 151.54 (J=247 Hz)	d 162.55 (J=244 Hz)	d 156.08 (J=247 Hz)
C-30	d 115.83 (J=3.2 Hz)	d 124.08 (J=3.5 Hz)	d 124.10 (J=3.4 Hz)	d 116.51 (J=22 Hz)	m 98.50	d 114.06 (J=21 Hz)
C-31	d 115.56 (J=12 Hz)	d 121.25 (J=8 Hz)	115.41	d 114.30 (J=6.7 Hz)	m 98.50	d 114.67 (J=9.4 Hz)

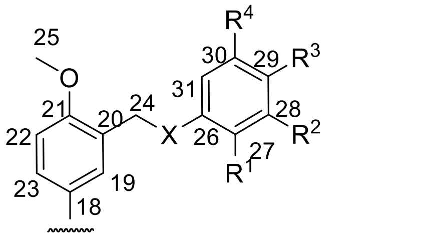

17g R^1=t-Bu R^2=H R^3=H R^4=Me X=S

17h R^1=H R^2=Me R^3=Cl R^4=H X=O

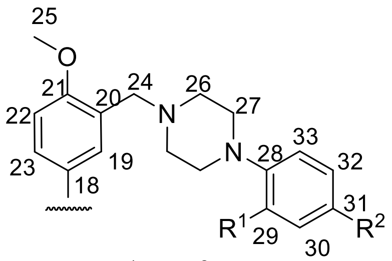

17i R^1=H R^2=F

17j R^1=OMe R^2=H

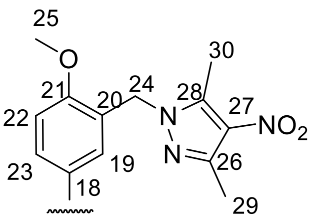

17k

Table S15: Spectra NMR^{1}H of **17g-17k**($CDCl_3$ for **g-j** and DMSO-d_6 for **k**, δ):

№	17g	17h	17i	17j	17k
H-14	s 7.08	s 7.07	s 7.00	s 6.98	s 7.23
H-17	s 7.49	s 7.69	bs 7.98	s 8.13	s 7.26
H-19	s 7.32	s 7.64	bs 7.98	s 8.05	s 7.67
H-22	d 6.76 (J=8.5 Hz)	m 6.76	d 6.56 (J=8.0 Hz)	d 6.46 (J=8.1 Hz)	d 7.08 (J=8.4 Hz)
H-23	m 7.40	d 7.51 (J=8.3 Hz)	m 7.34	m 6.84-7.03	d 7.50 (J=8.4 Hz)
H-24	s 4.04	s 4.99	s 4.11	s 4.19	s 5.27
H-25	s 3.77	s 3.84	s 3.61	bs 3.51	s 3.85
H-26			s 3.29	bs 3.51	
H-27	s 1.22 (t-Bu)	d 6.88 (J=2.4 Hz)	s 3.48	m 3.85	
H-28	m 7.23	s 2.34 (Me)			
H-29	m 7.08		m 6.83-6.94	m 6.84-7.03	s 2.40
H-30	s 2.33 (Me)	d 7.21 (J=8.6 Hz)	m 6.83-6.94	m 3.85 (OMe)	s 2.59
H-31	m 7.32	d 6.83 (J=8.5 Hz)		m 6.84-7.03	
H-32			m 6.83-6.94	m 6.84-7.03	
H-33			m 6.83-6.94	m 6.84-7.03	
NH	bs 9.09	bs 9.56	bs 11.22	bs 11.22	bs 12.28
OH-3	s 18.79	s 18.79	bs 18.7	bs 18.76	bs 18.80
OH-7	---	---	bs 12.64	bs 12.63	bs 12.77
OH-9	s 10.26	s 10.27	s 10.24	s 10.23	s 10.30

Table S16: Spectra NMR ^{13}C of **17g-17k**($CDCl_3$ for **g-j** and DMSO-d_6 for **k**, δ):

№	**17g**	**17h**	**17i**	**17j**	**17k**
C-17	142.74	143.61	141.77	141.03	142.40
C-18	125.70	125.47	127.27	127.72	123.52
C-19	127.77	127.19	129.01	129.77	126.09
C-20	126.47	125.90	127.27	127.72	126.99
C-21	158.61	158.08	158.70	158.36	157.16
C-22	110.38	110.21	110.52	110.89	110.26
C-23	128.79	129.46	131.17	131.96	128.05
C-24	32.56	64.74	48.25	47.48	47.97
C-25	55.50	55.45	55.35	55.26	55.41
C-26	134.44	157.12	54.52	54.64	140.77
C-27	149.06 (C_{Ar})<br>31.09 (t-Bu)	113.28	51.98	52.05	145.97
C-28	129.57	127.92<br>20.25 (Me)	146.65	151.77	130.90
C-29	128.79	136.97	d 115.45<br>(J=22 Hz)	139.11 (C_{Ar})<br>54.96(O-Me)	11.32
C-30	135.72 (C_{Ar})<br>19.73 (Me)	126.01	d 118.53<br>(J=7.43 Hz)	110.89	14.07
C-31	123.63	117.22	d 156.02<br>(J=240 Hz)	118.75	
C-32			d 118.53<br>(J=7.43 Hz)	124.02	
C-33			d 115.45<br>(J=22 Hz)	121.04	

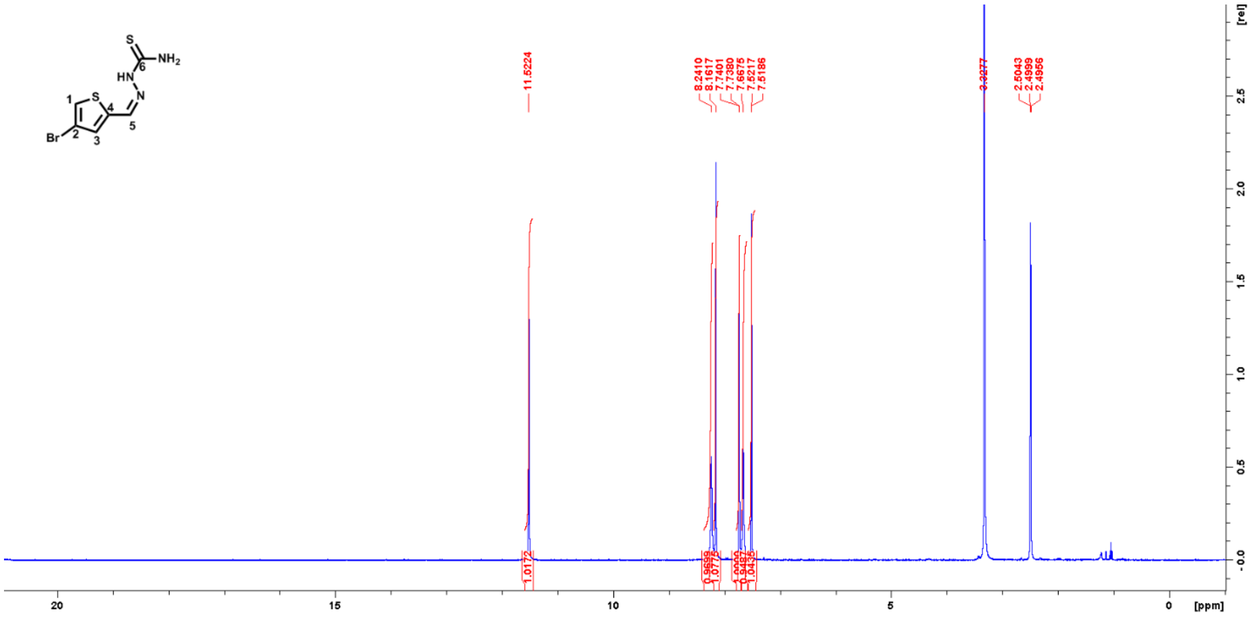

Figure S1: NMR ^1H spectra of **11h**

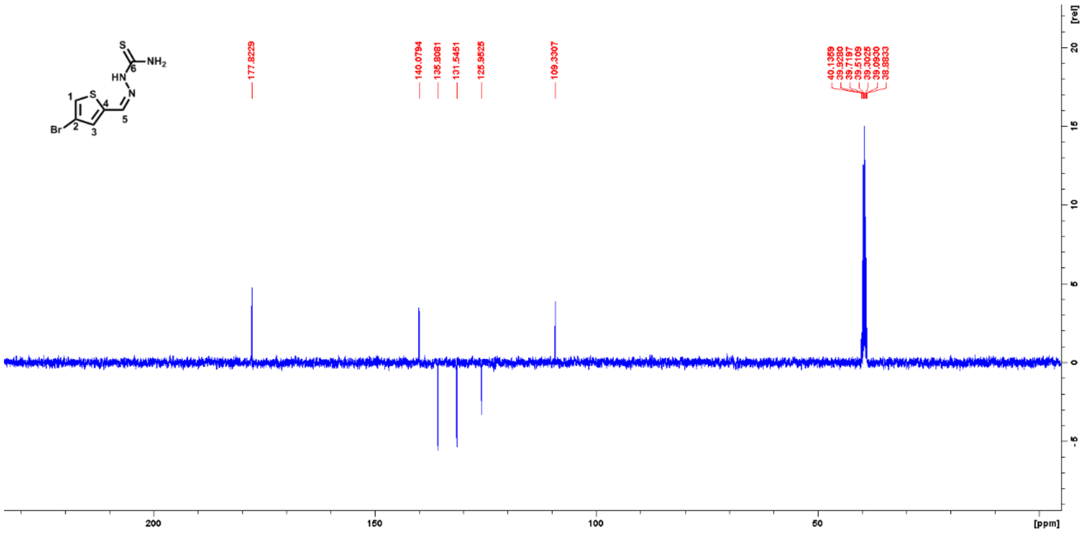

Figure S2: NMR ^{13}C (J-MOD) spectra of **11h**

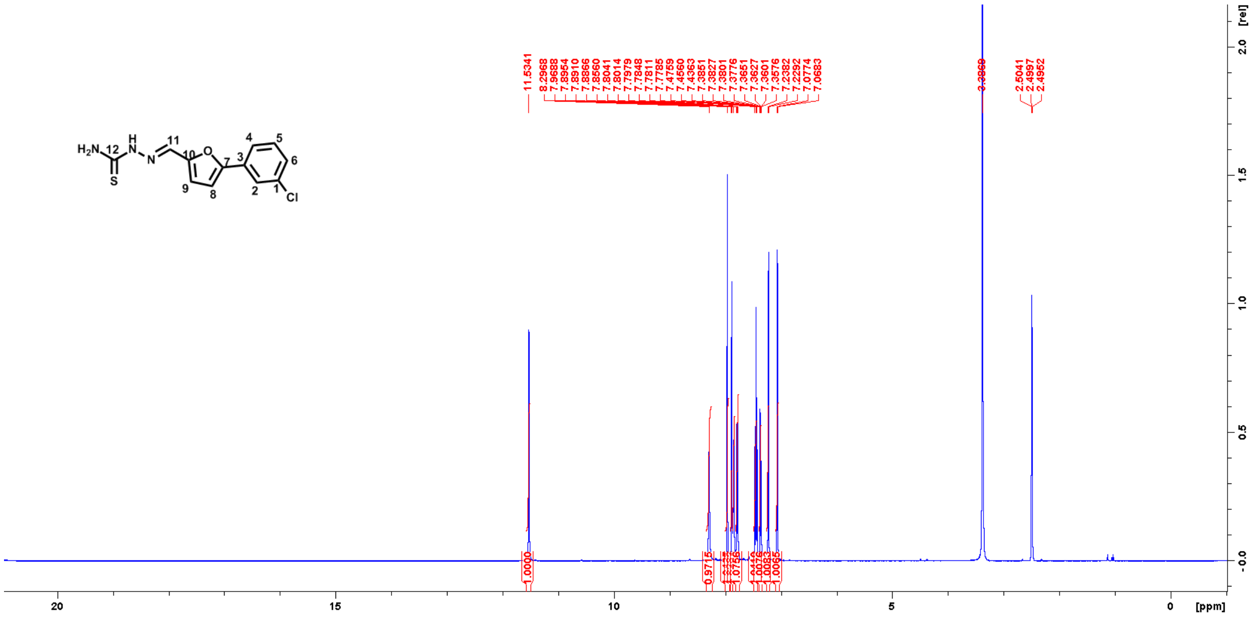

Figure S3: NMR ^{1}H spectra of **11r**

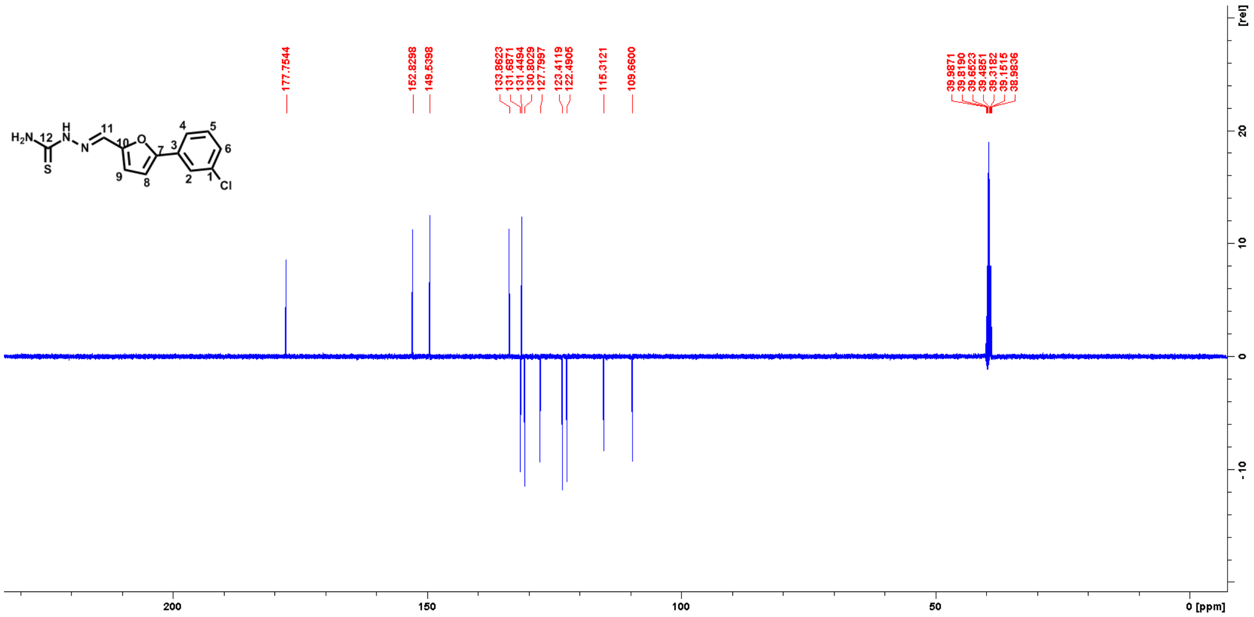

Figure S4: NMR ^{13}C (J-MOD) spectra of **11r**

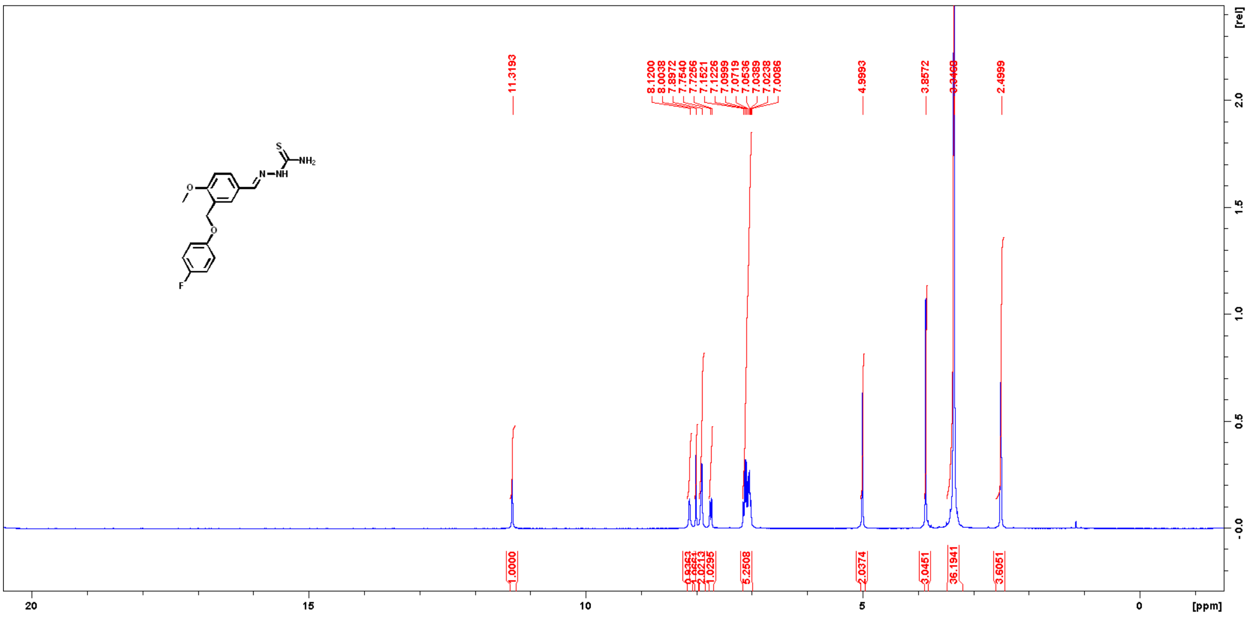

Figure S5: NMR ^{1}H spectra of **12a**

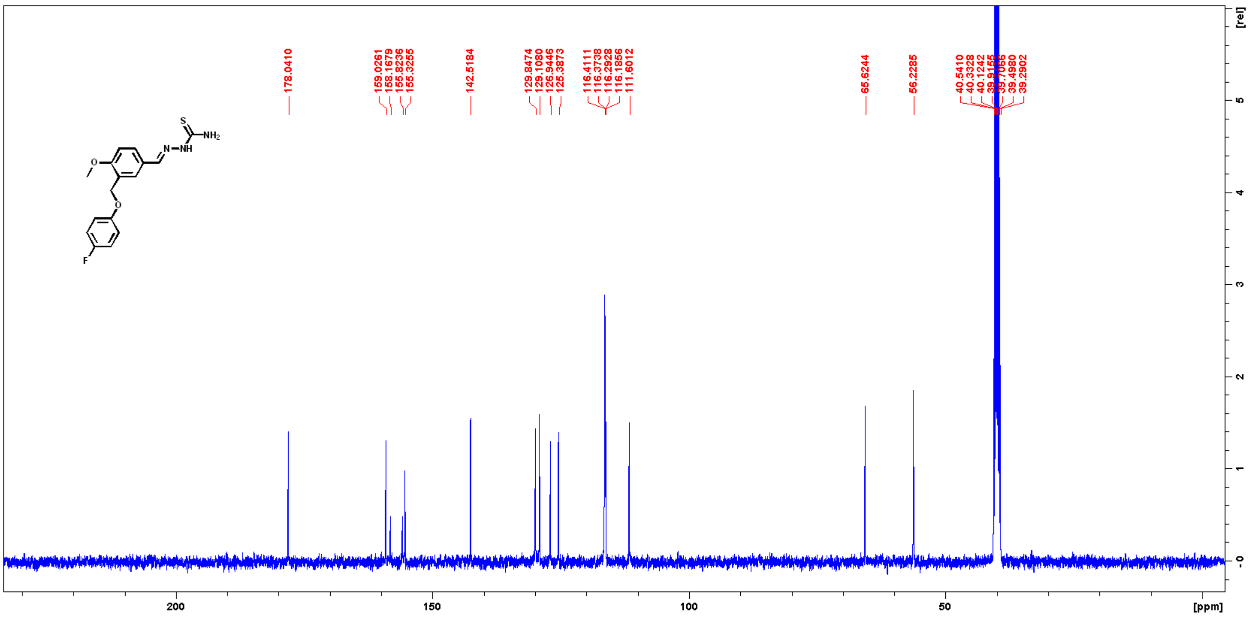

Figure S6: NMR ^{13}C spectra of **12a**

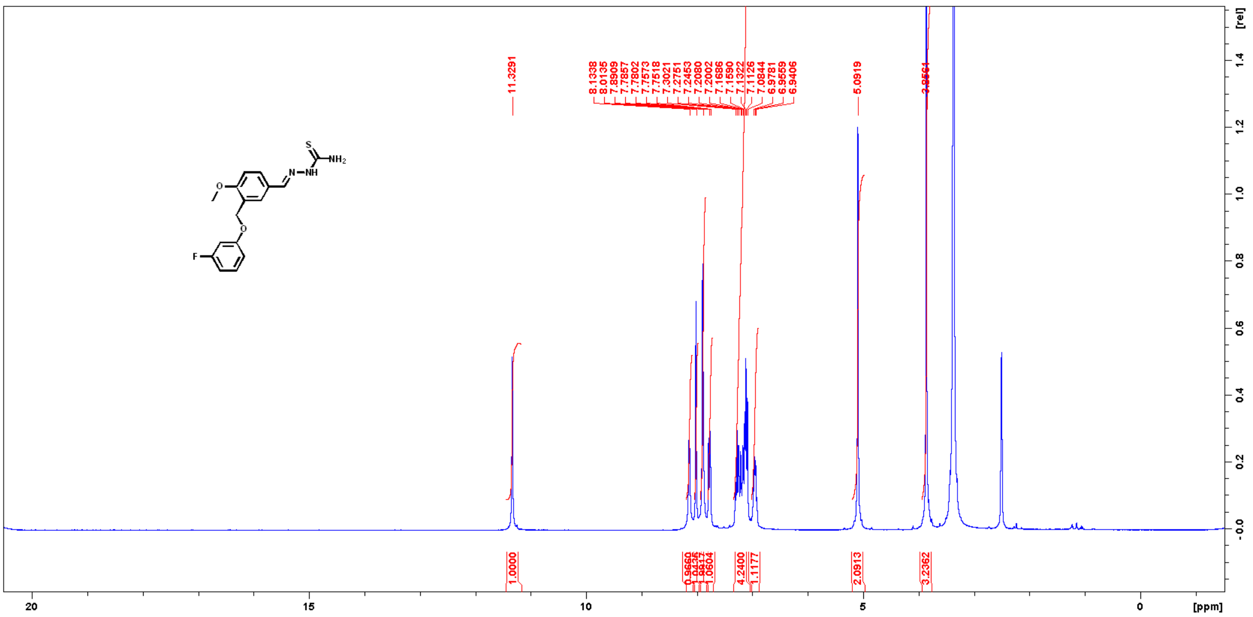

Figure S7: NMR ^{1}H spectra of **12b**

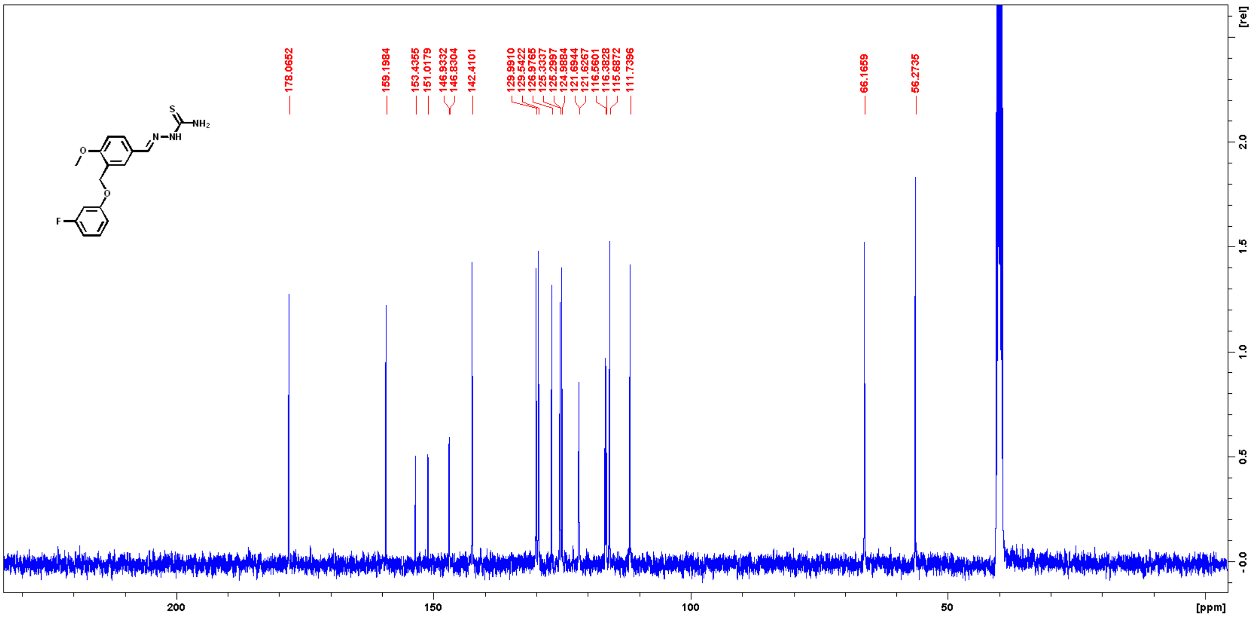

Figure S 8: NMR ^{13}C spectra of **12b**

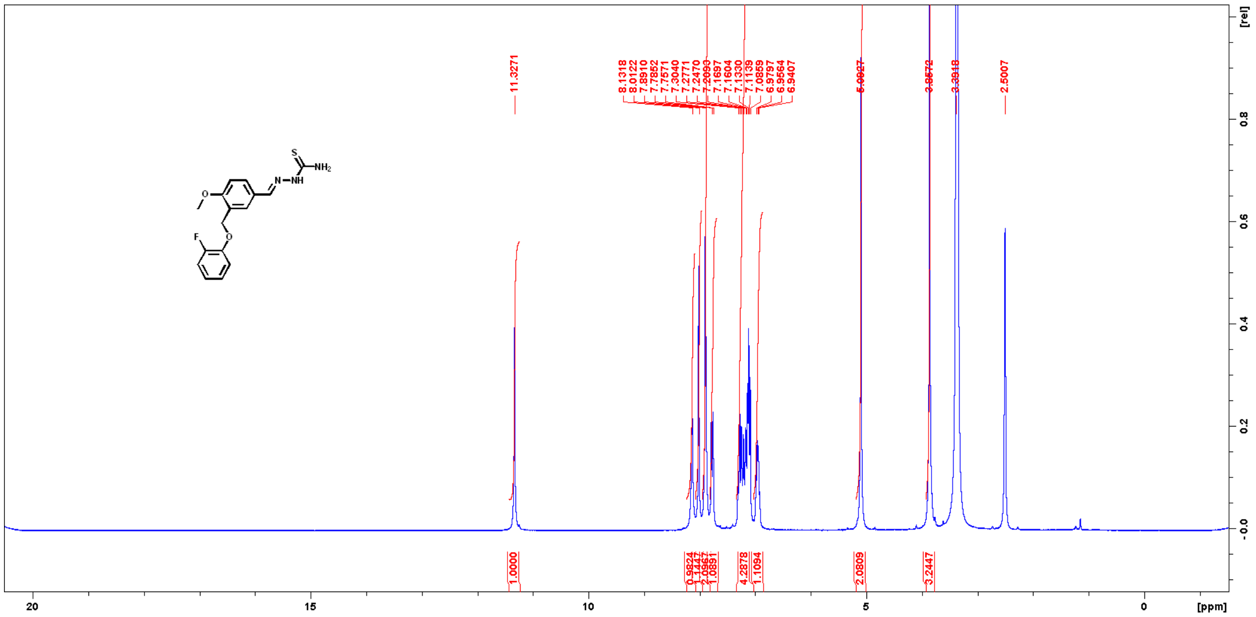

Figure S9: NMR ^{1}H spectra of **12c**

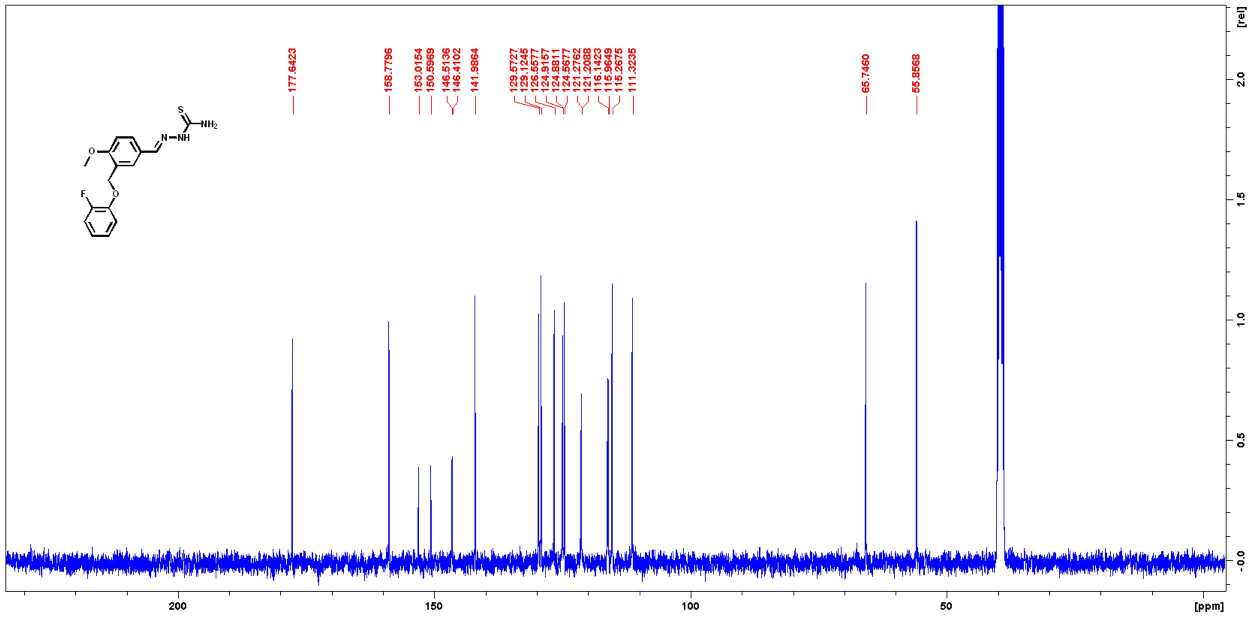

Figure S10: NMR ^{13}C spectra of **12c**

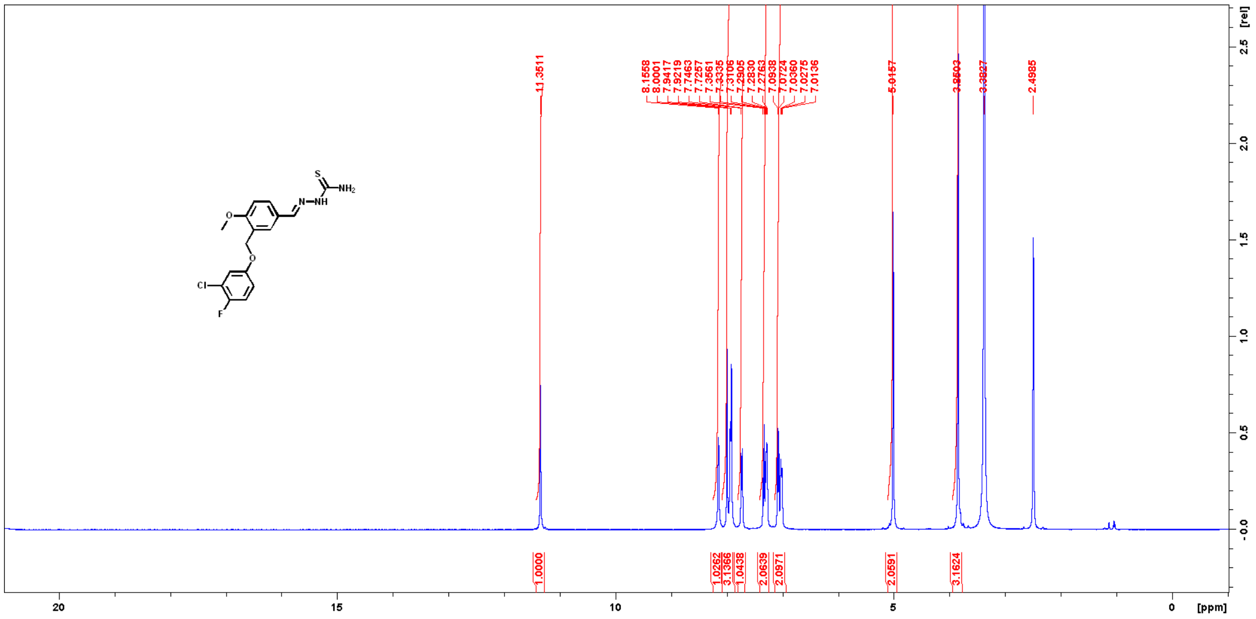

Figure S11: NMR ^{1}H spectra of **12d**

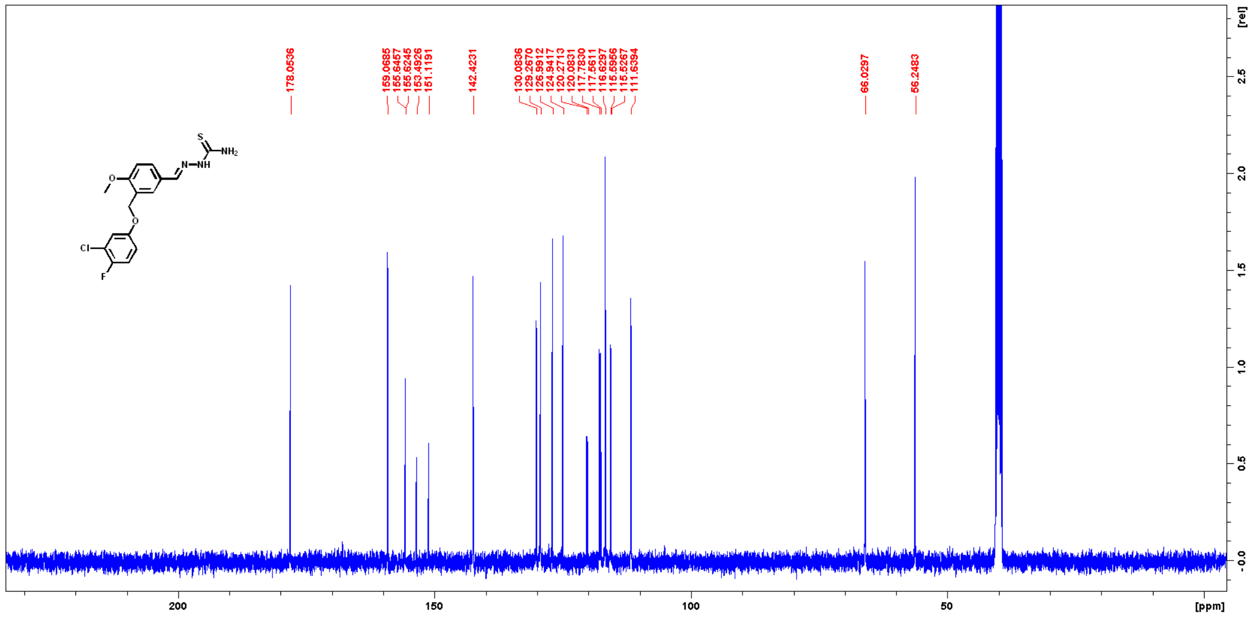

Figure S12: NMR ^{13}C spectra of **12d**

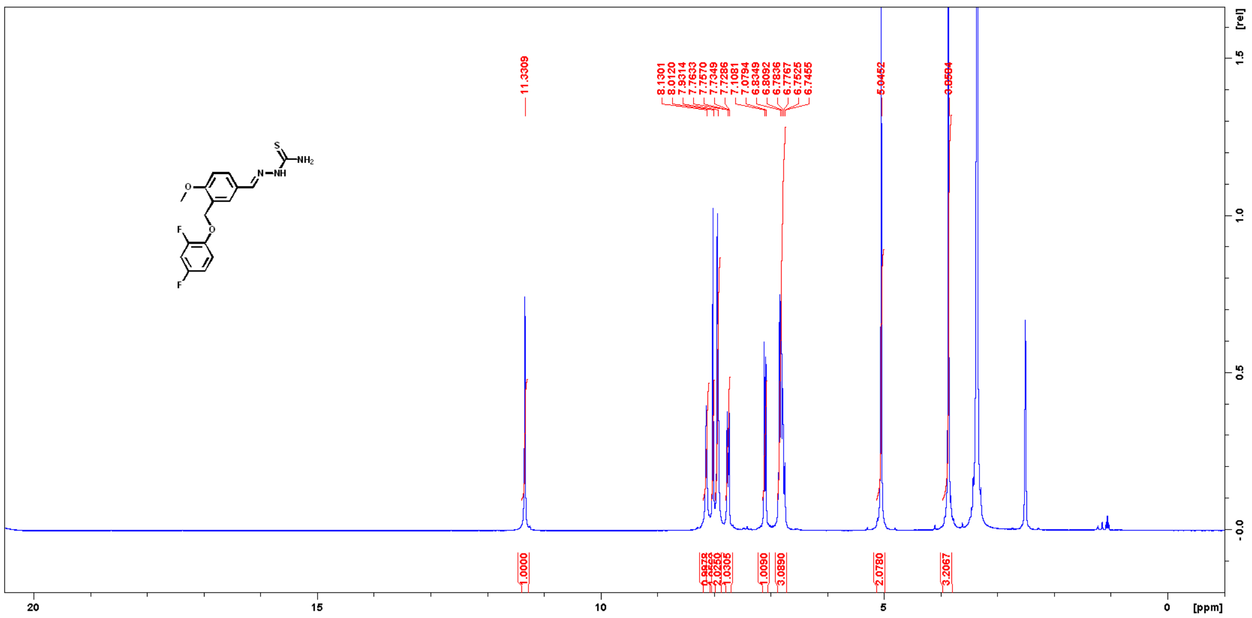

Figure S13: NMR ^{1}H spectra of **12e**

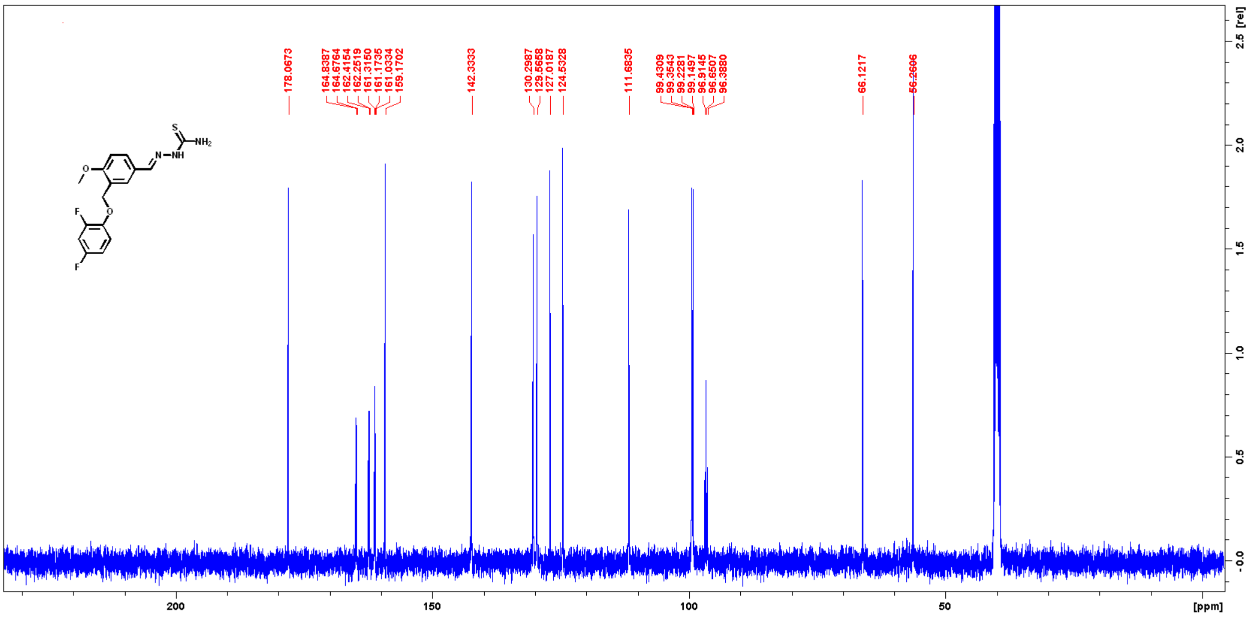

Figure S14: NMR ^{13}C spectra of **12e**

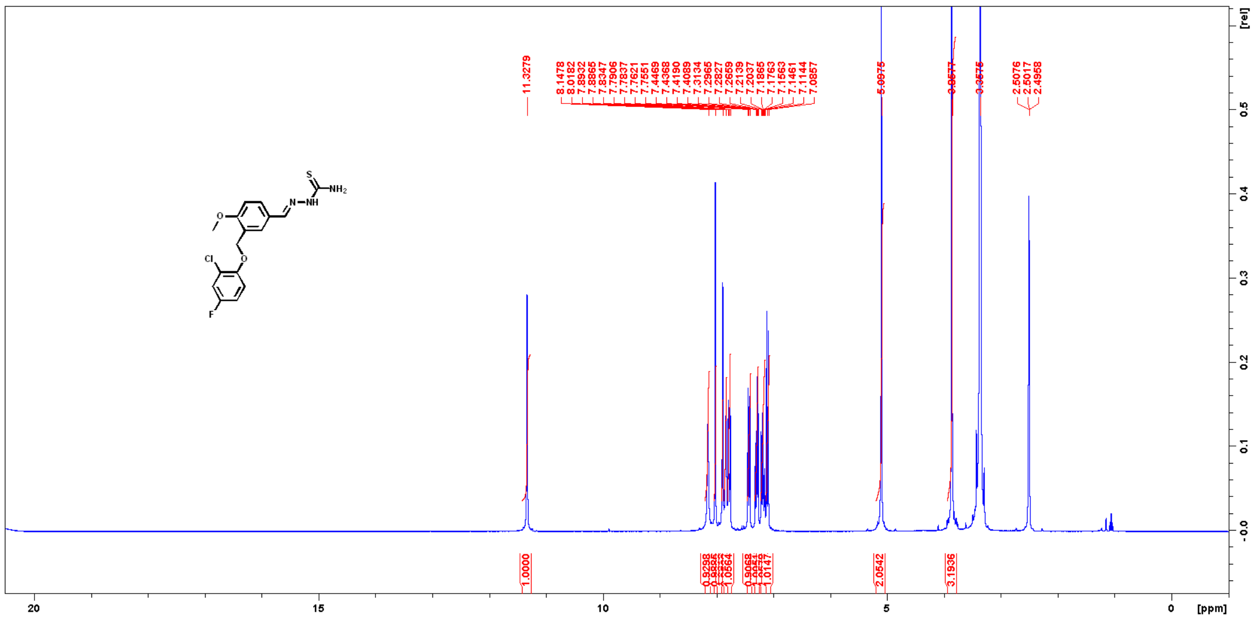

Figure S15: NMR ^{1}H spectra of **12f**

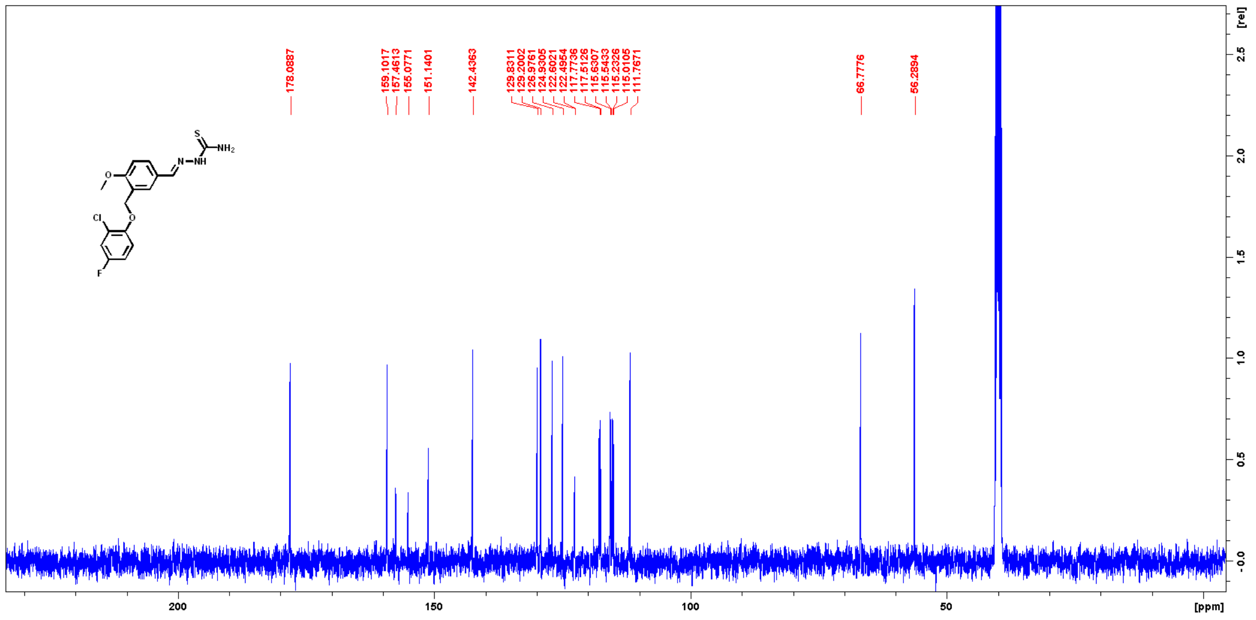

Figure S16: NMR ^{13}C spectra of **12f**

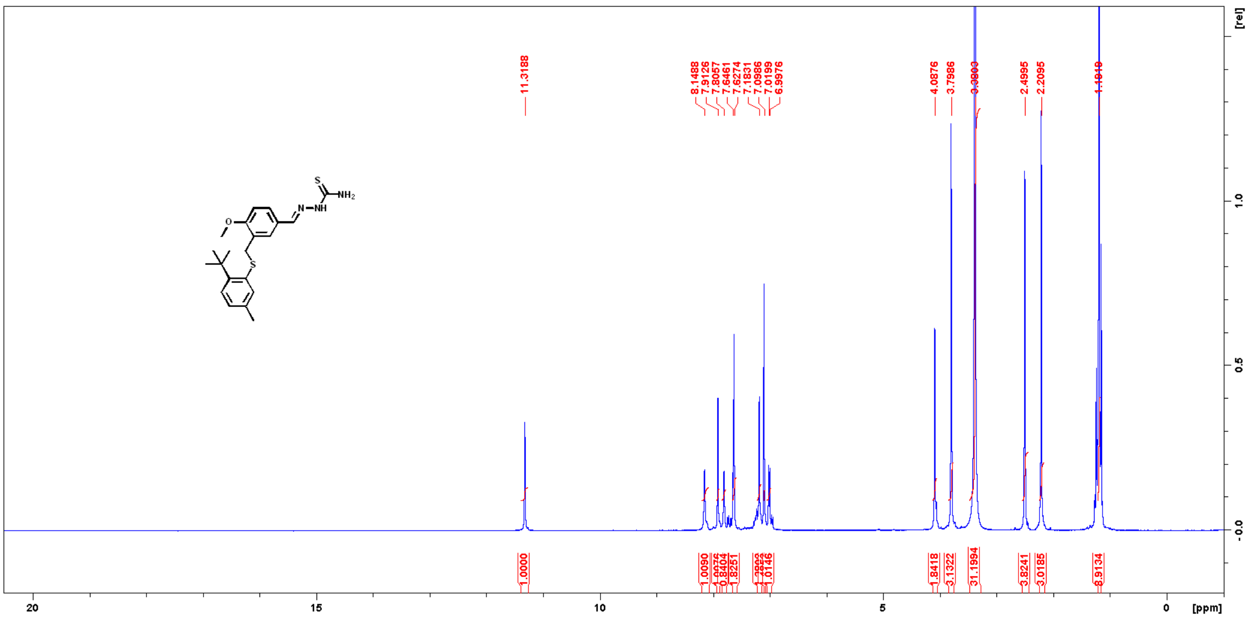

Figure S17: NMR ^{1}H spectra of **12g**

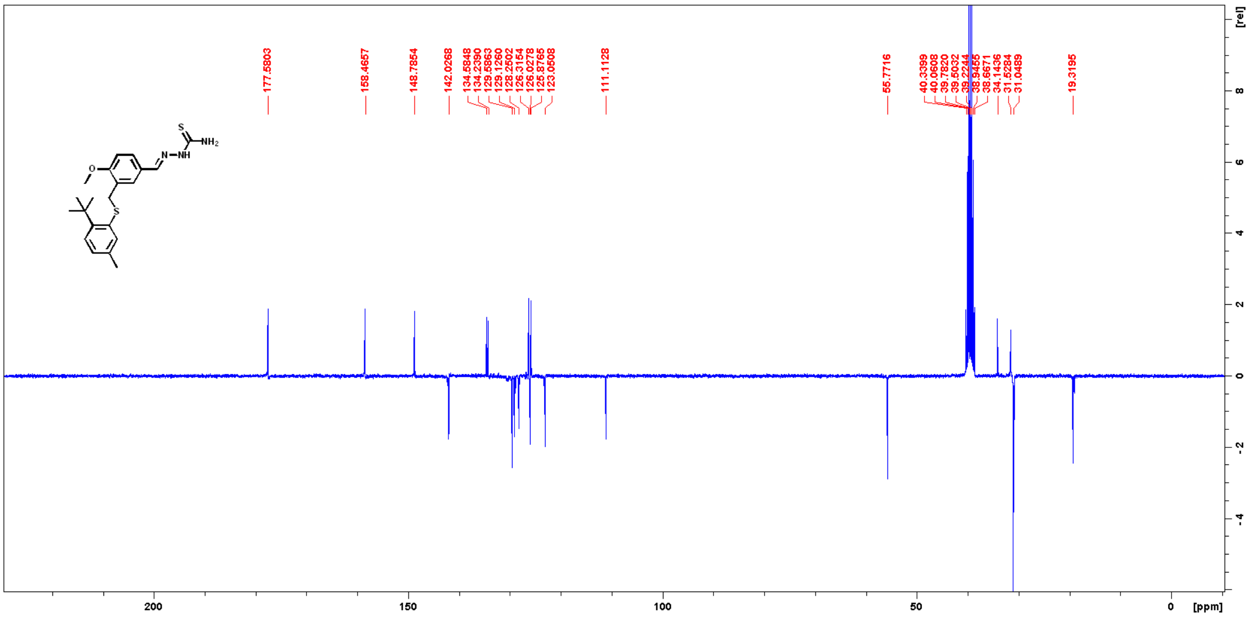

Figure S18: NMR ^{13}C (J-MOD) spectra of **12g**

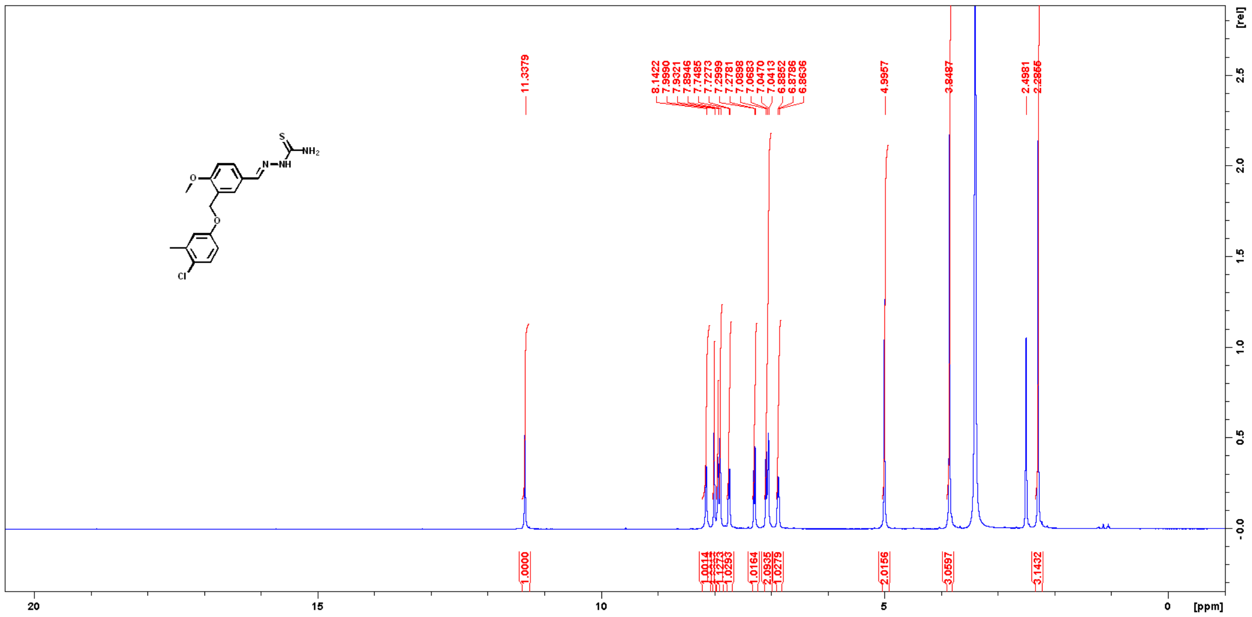

Figure S19: NMR ^{1}H spectra of **12h**

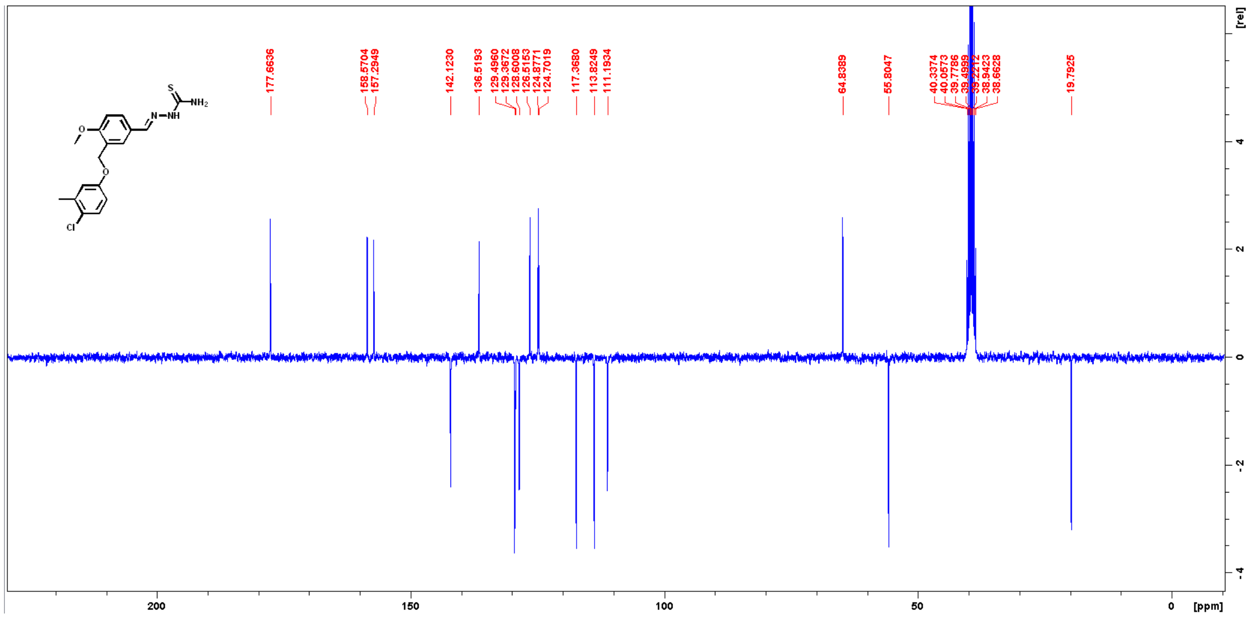

Figure S20: NMR ^{13}C (J-MOD) spectra of **12h**

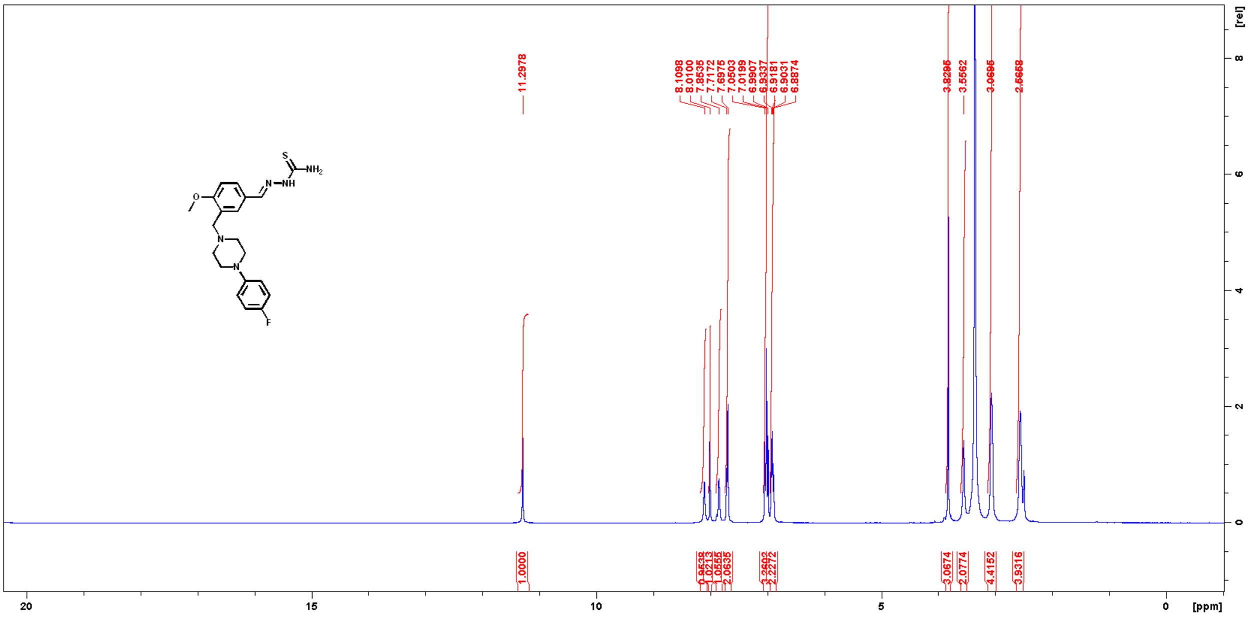

Figure S21: NMR ^{1}H spectra of **12i**

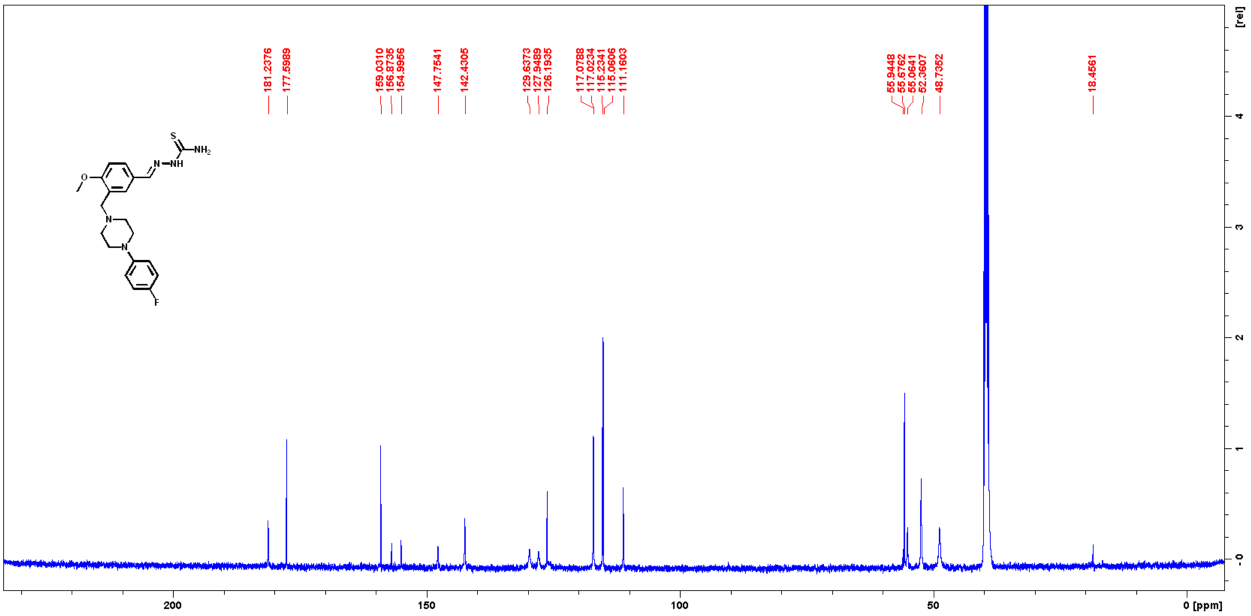

Figure S22: NMR ^{13}C spectra of **12i**

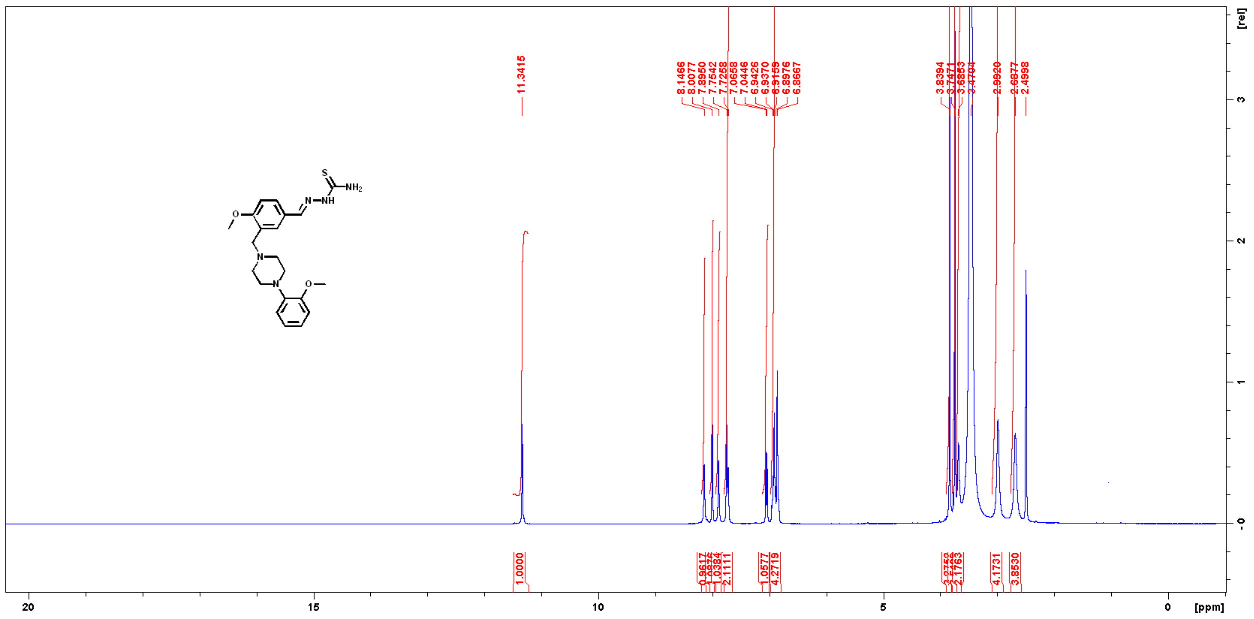

Figure S23: NMR ^{1}H spectra of **12j**

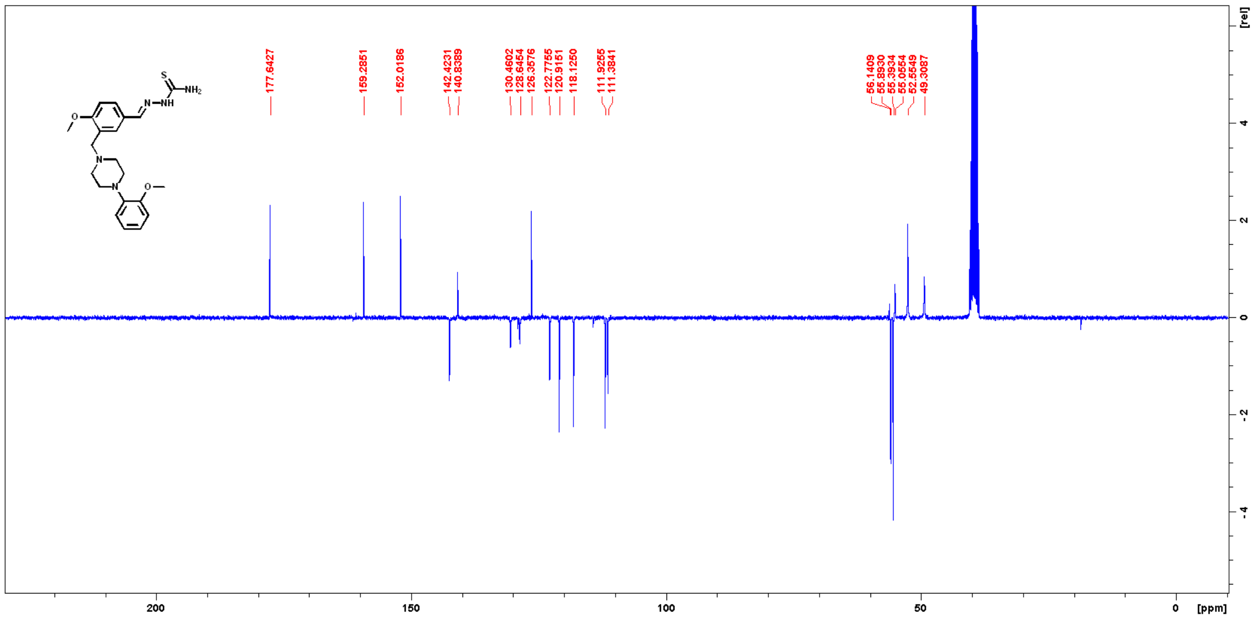

Figure S24: NMR ^{13}C (J-MOD) spectra of **12j**

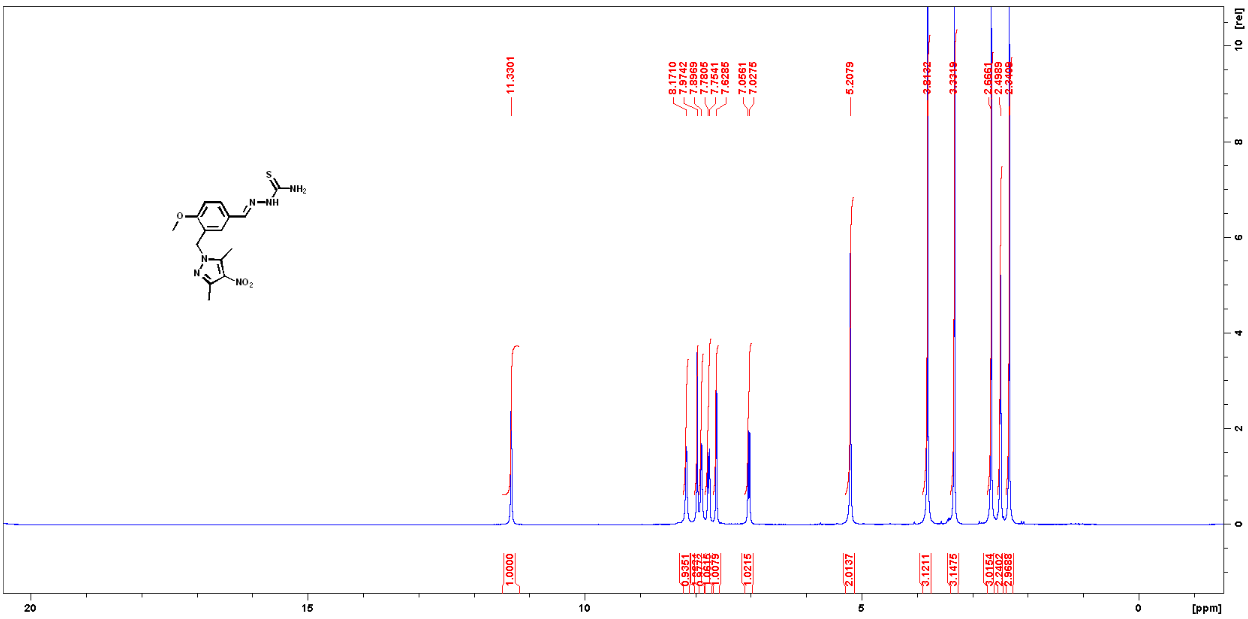

Figure S25: NMR ^{1}H spectra of **12k**

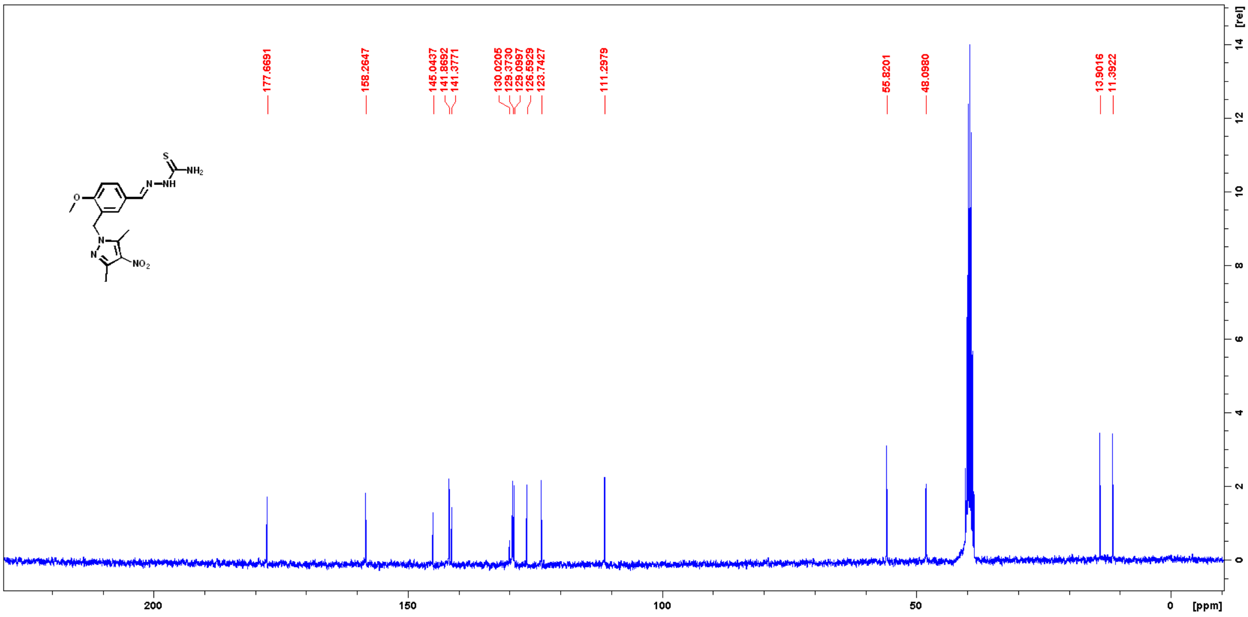

Figure S26: NMR ^{13}C spectra of **12k**

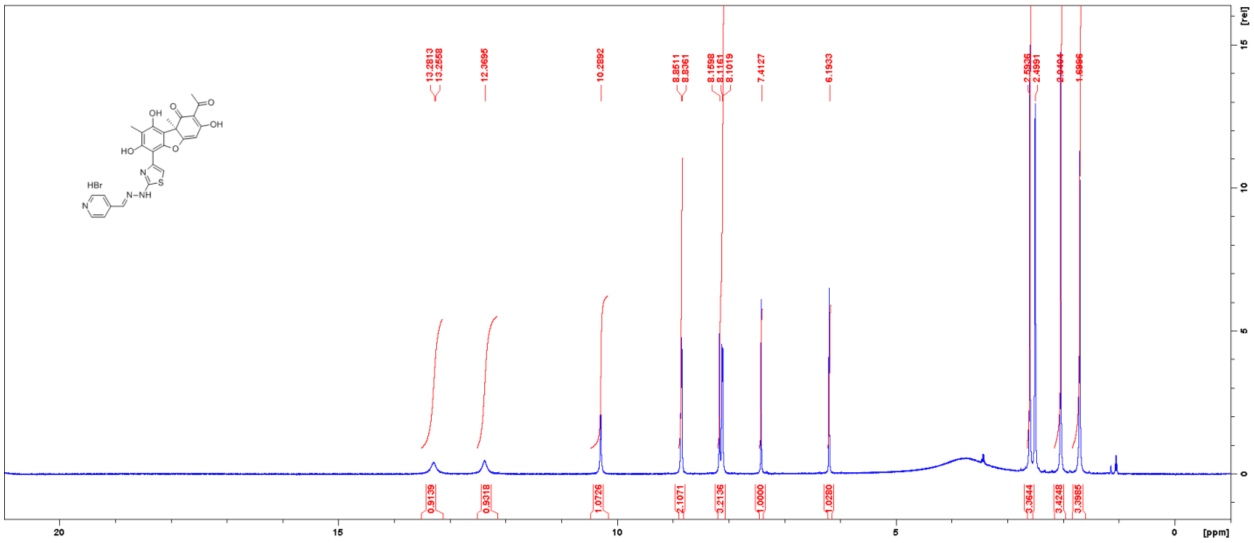

Figure S27: NMR ^1H spectra of **16a**

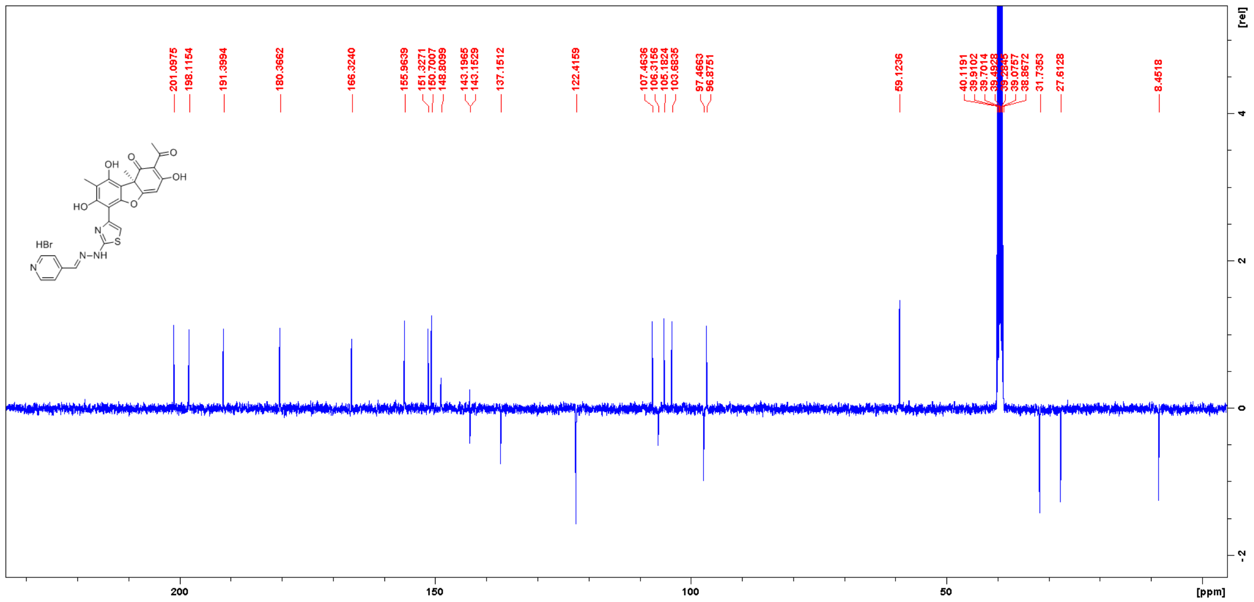

Figure S28: NMR ^{13}C (J-MOD) spectra of **16a**

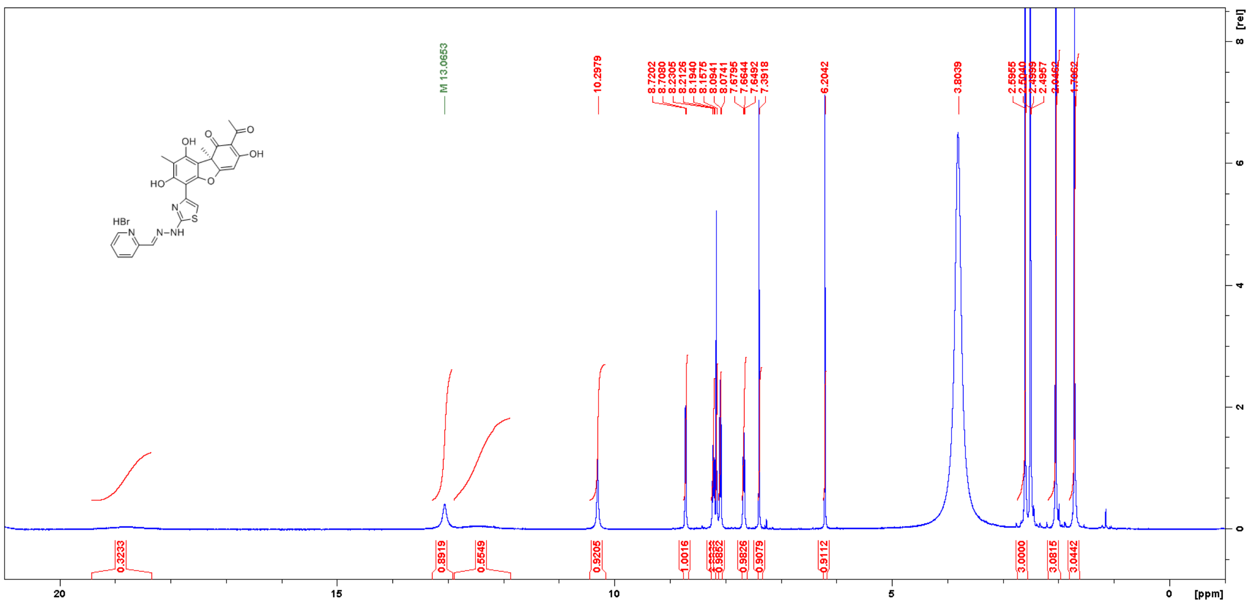

Figure S29: NMR ^{1}H spectra of **16b**

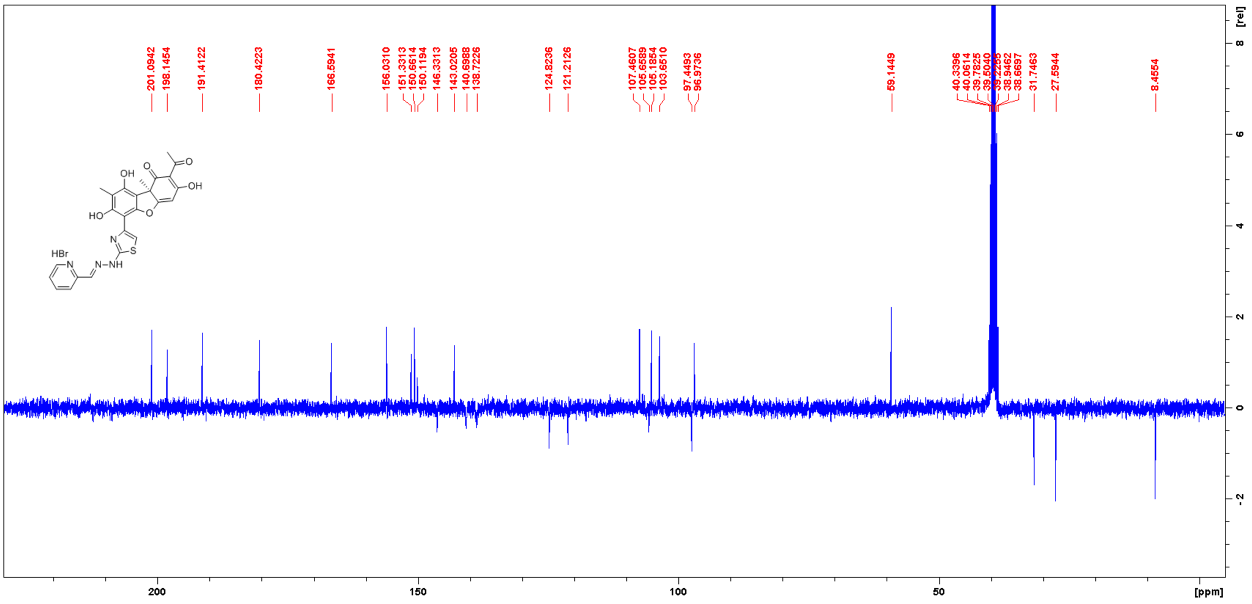

Figure S30: NMR ^{13}C (J-MOD) spectra of **16b**

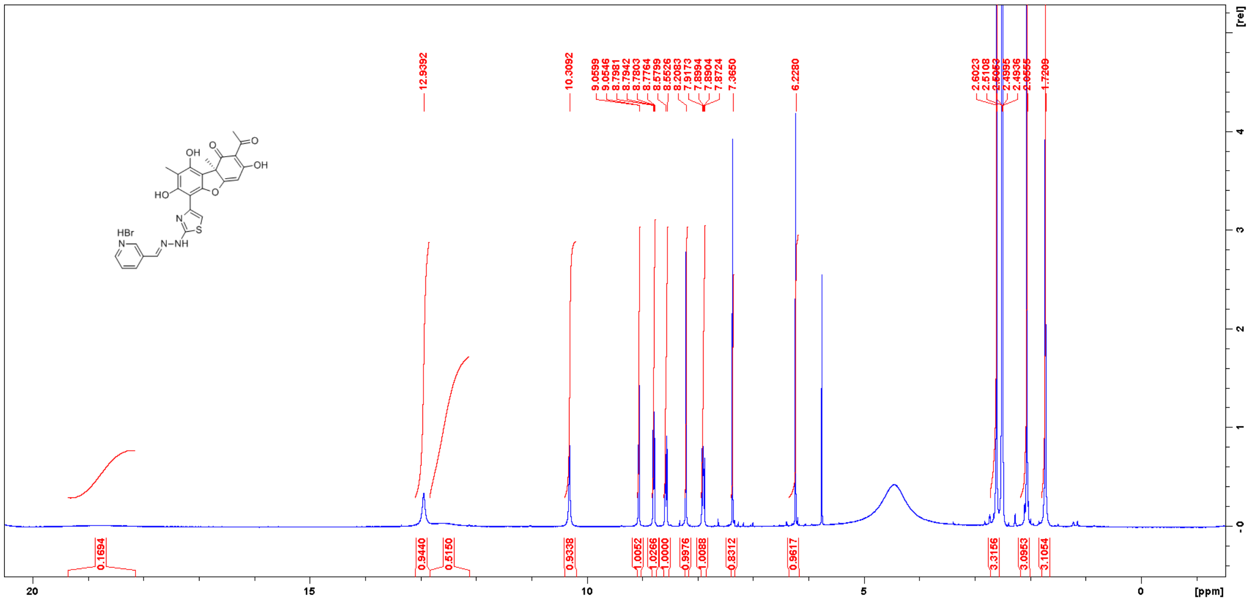

Figure S31: NMR ^{1}H spectra of **16c**

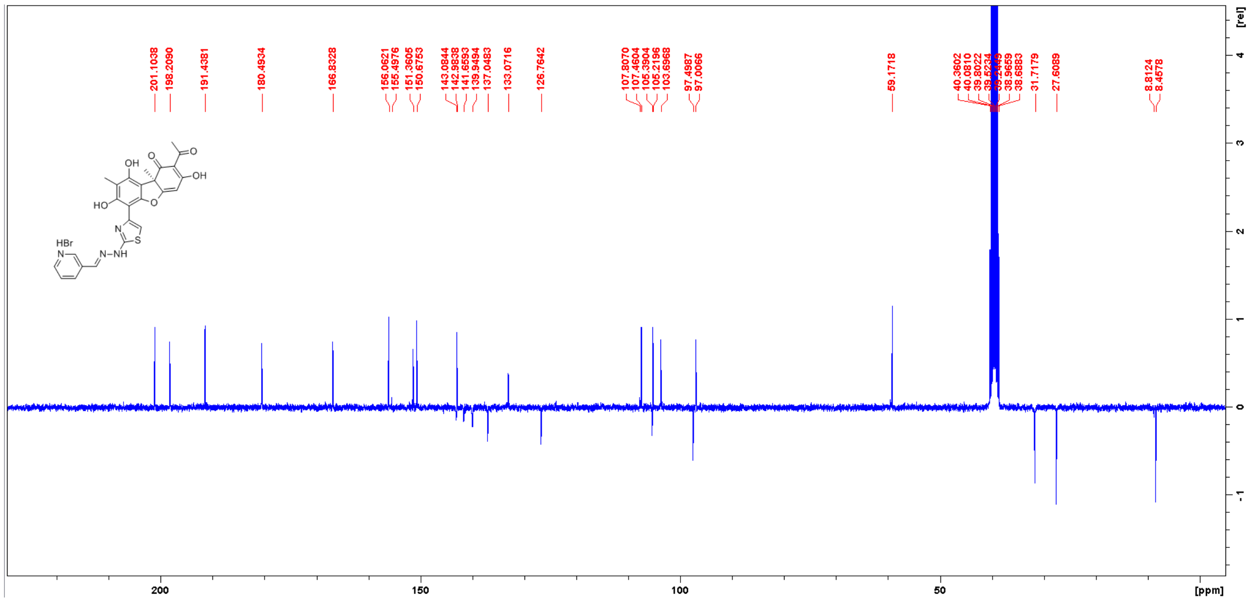

Figure S32: NMR ^{13}C (J-MOD) spectra of **16c**

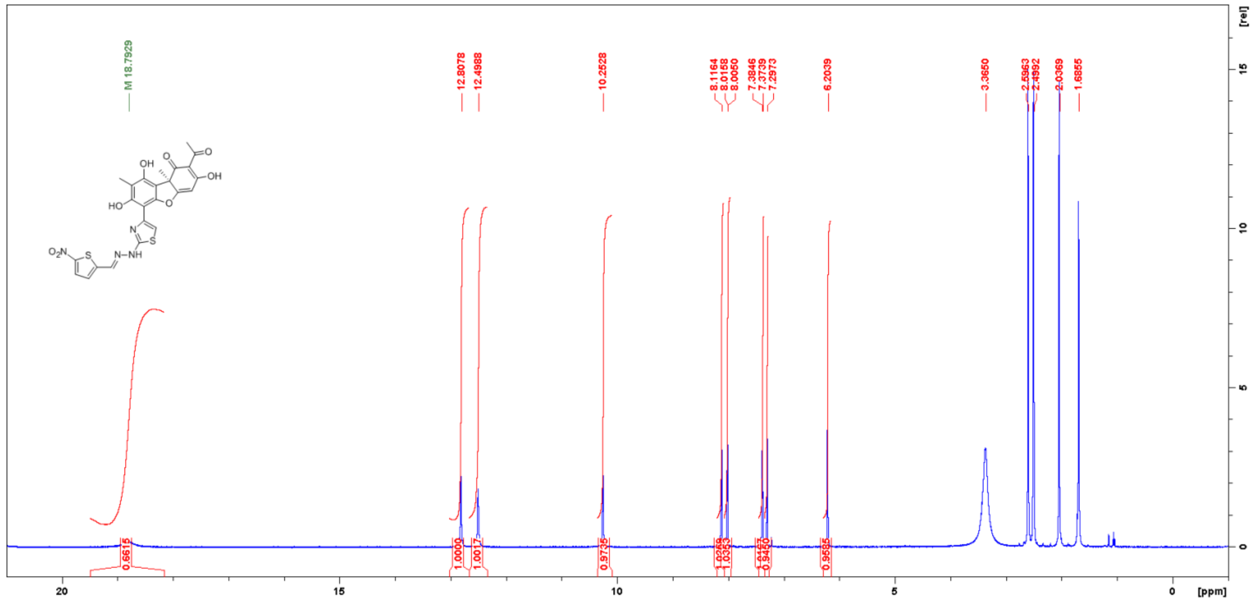

Figure S33: NMR ^{1}H spectra of **16d**

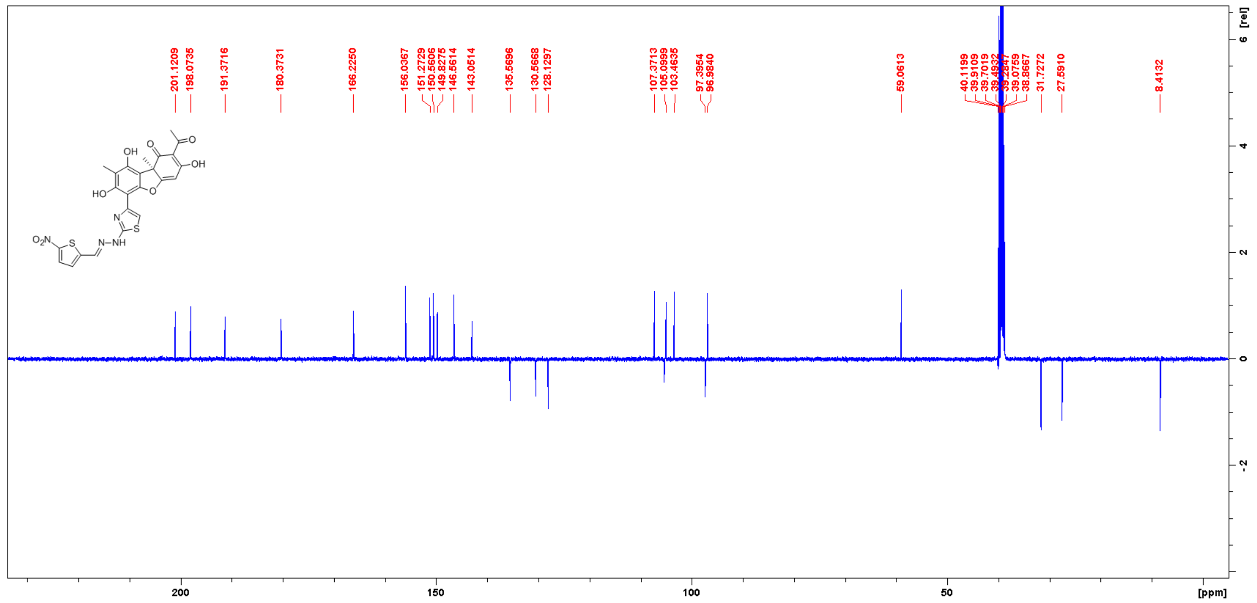

Figure S34: NMR ^{13}C (J-MOD) spectra of **16d**

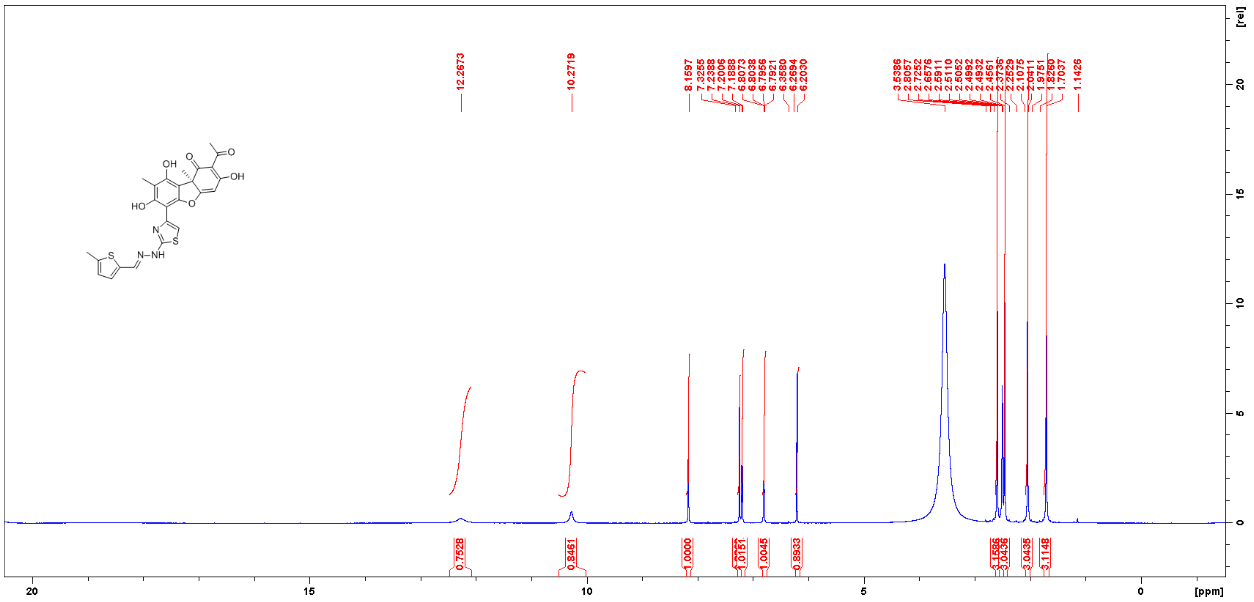

Figure S35: NMR ^{1}H spectra of **16e**

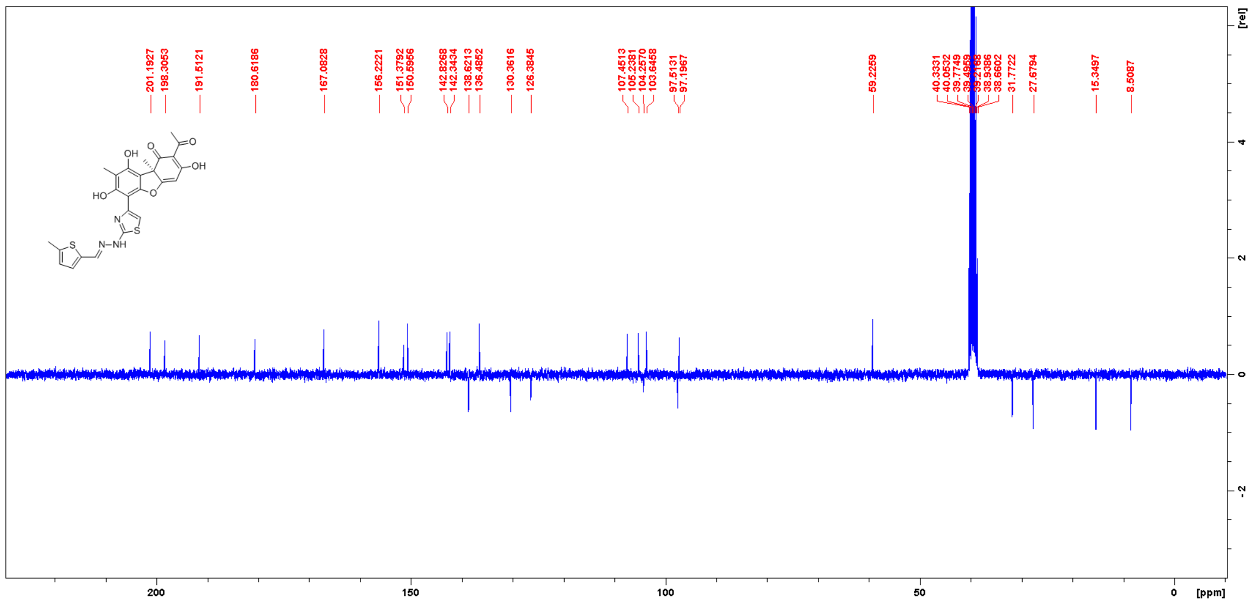

Figure S36: NMR ^{13}C (J-MOD) spectra of **16e**

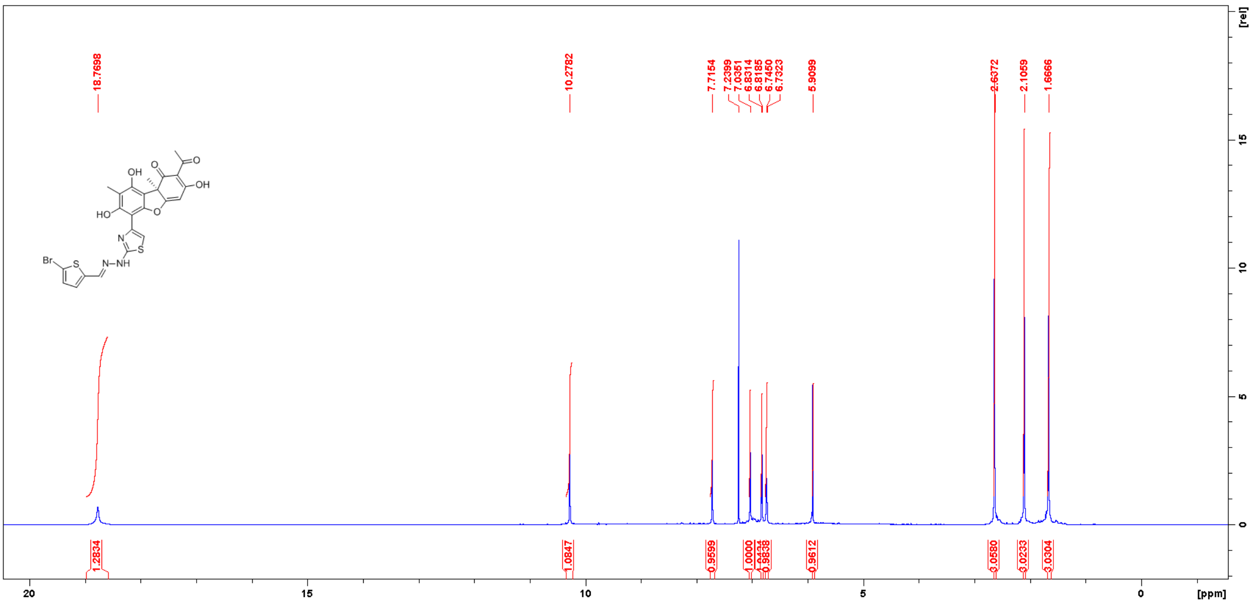

Figure S37:NMR ^{1}H spectra of **16f**

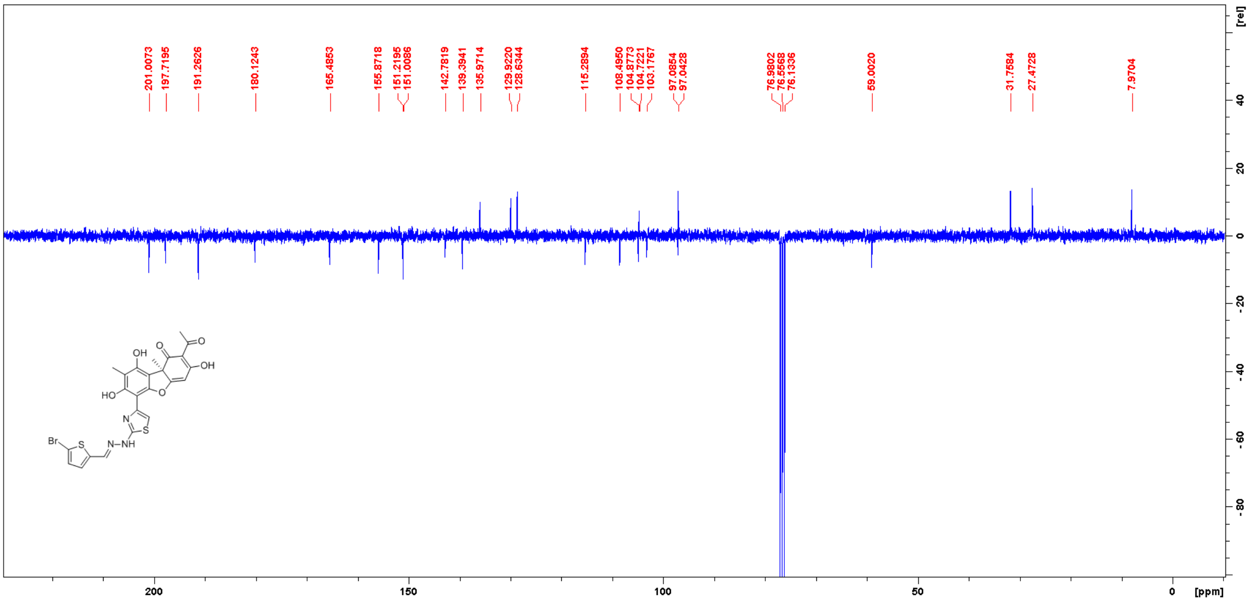

Figure S38:NMR ^{13}C (J-MOD) spectra of **16f**

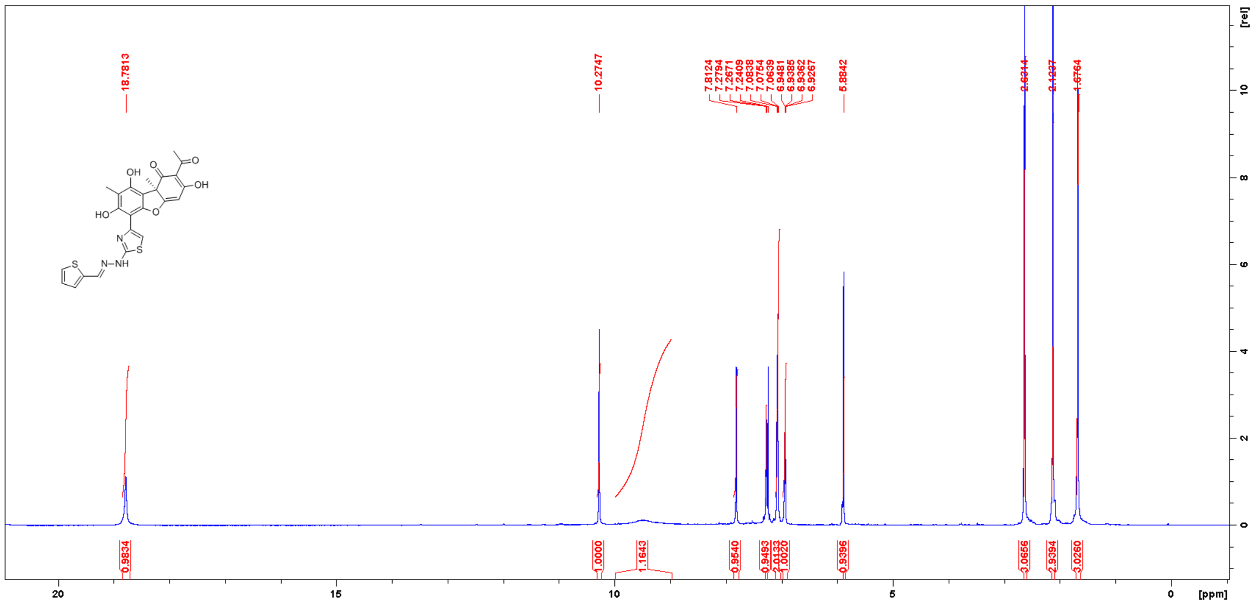

Figure S39:NMR ^{1}H spectra of **16g**

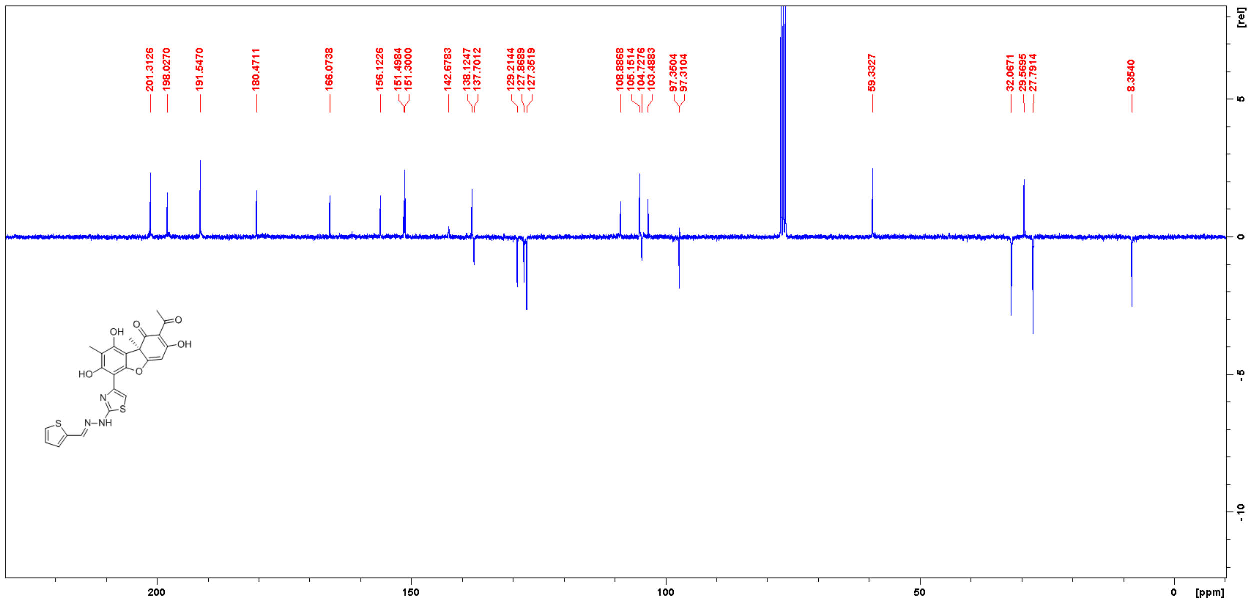

Figure S40: NMR ^{13}C (J-MOD) spectra of **16g**

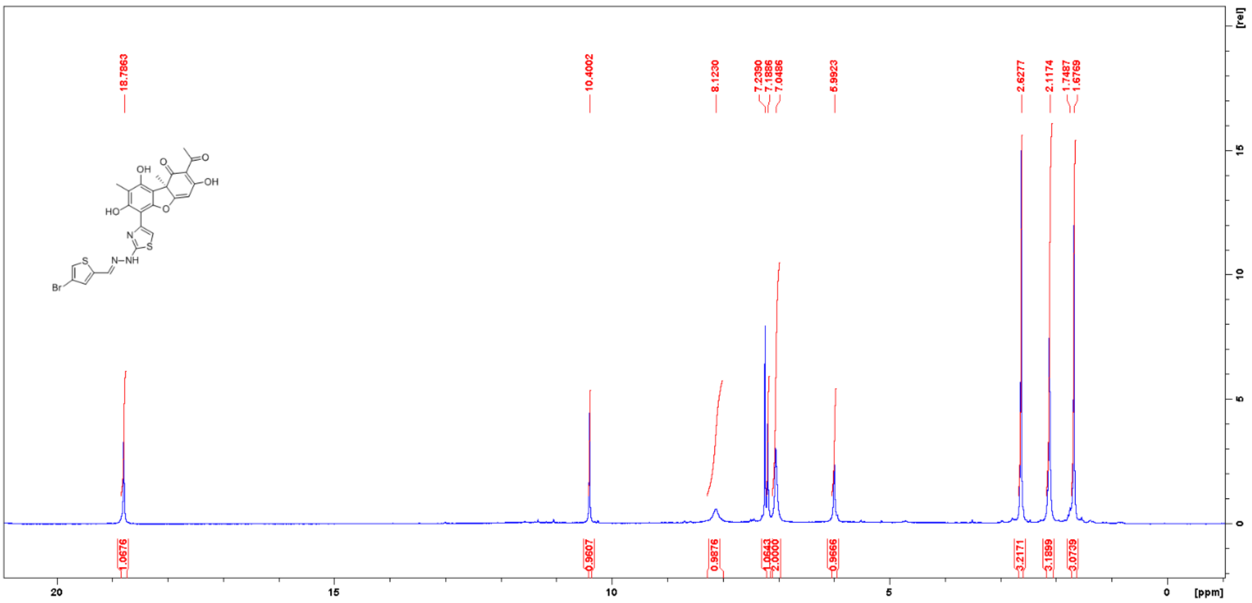

Figure S41: NMR ^{1}H spectra of **16h**

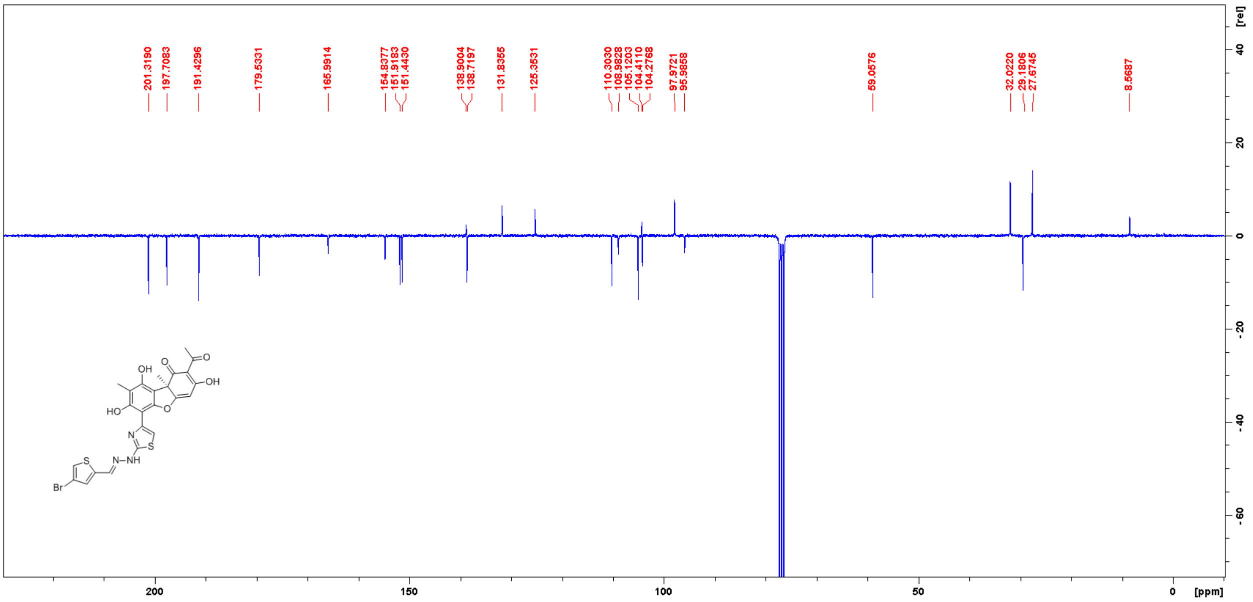

Figure S42:NMR ^{13}C (J-MOD) spectra of **16h**

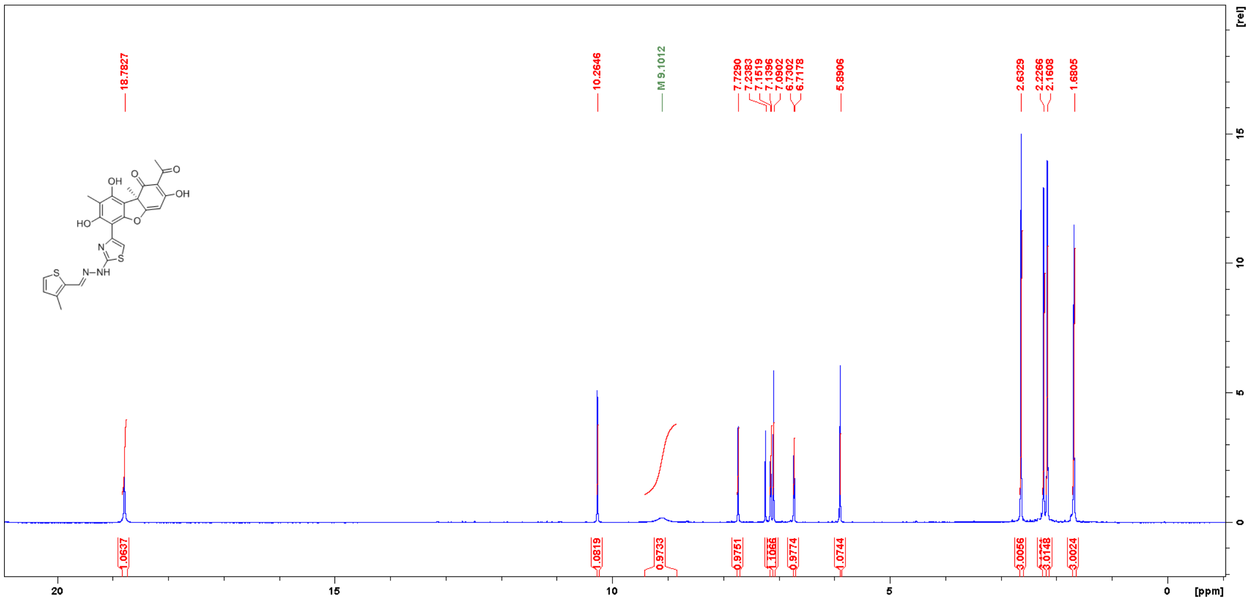

Figure S43:NMR ^{1}H spectra of **16i**

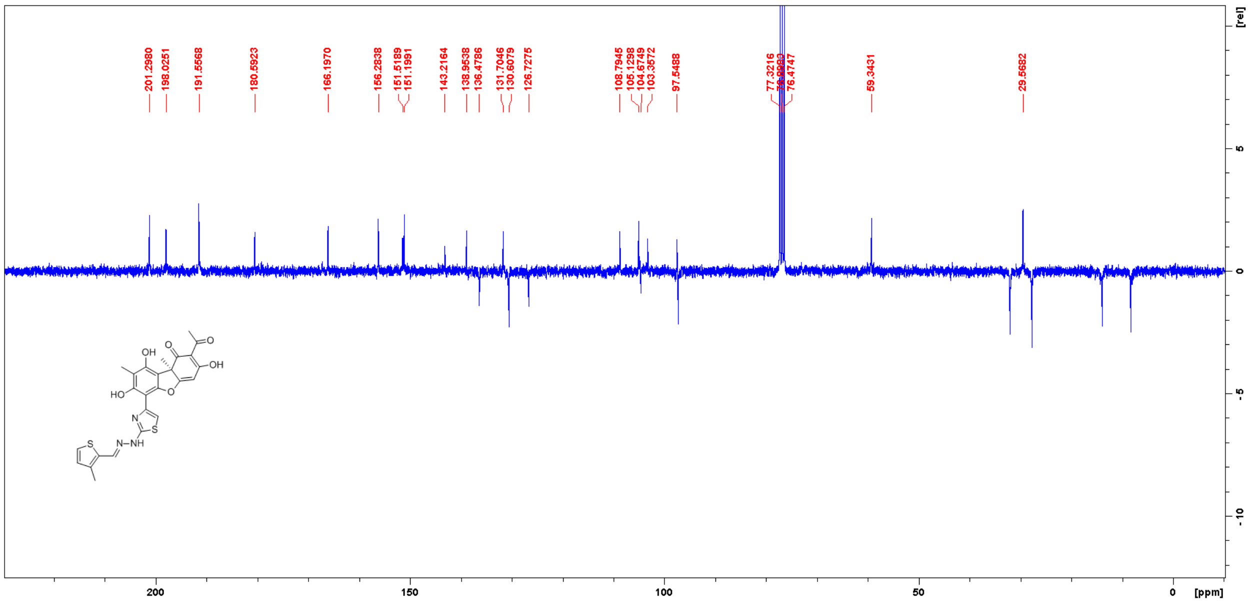

Figure S44:NMR ^{13}C (J-MOD) spectra of **16i**

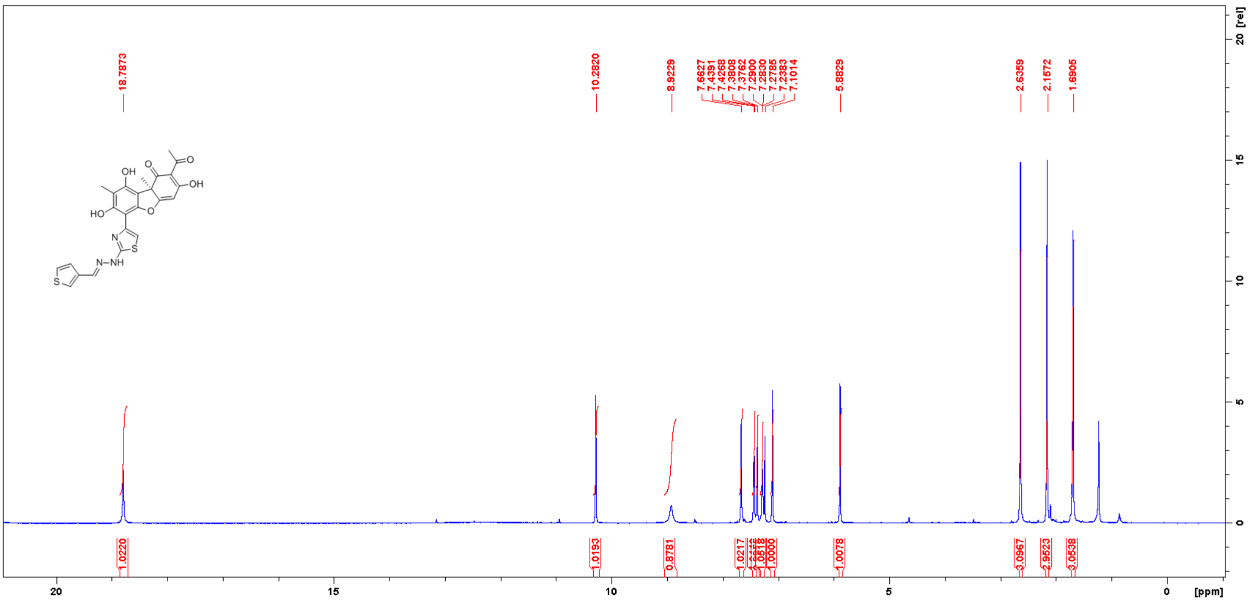

Figure S45:NMR ^{1}H spectra of **16j**

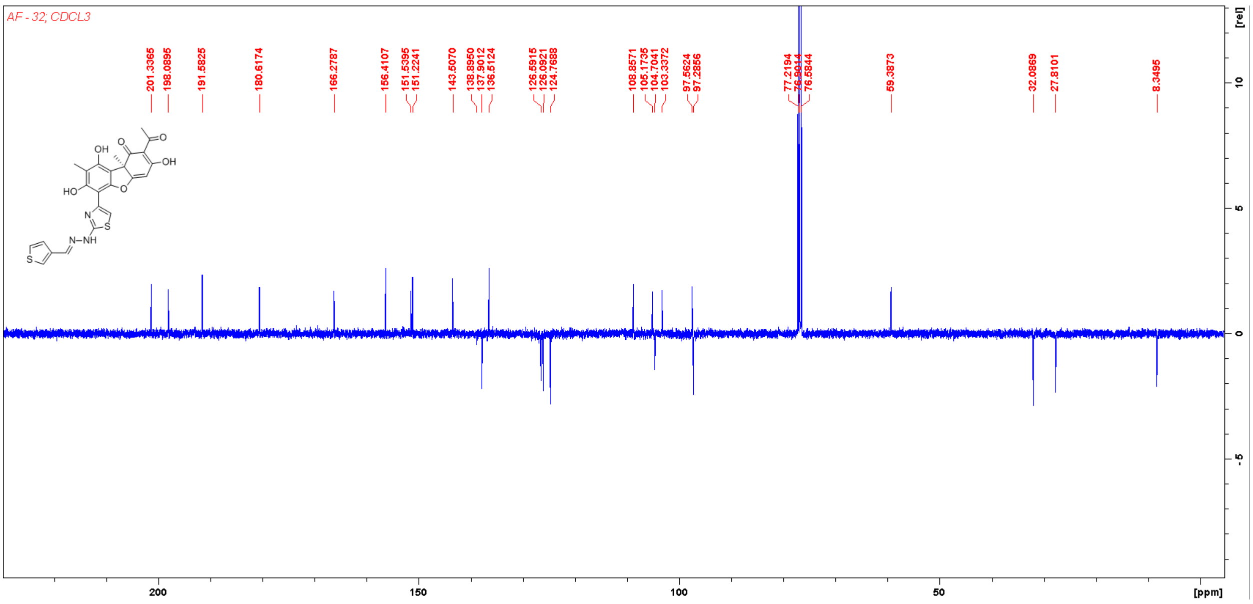

Figure S46:NMR ^{13}C (J-MOD) spectra of **16j**

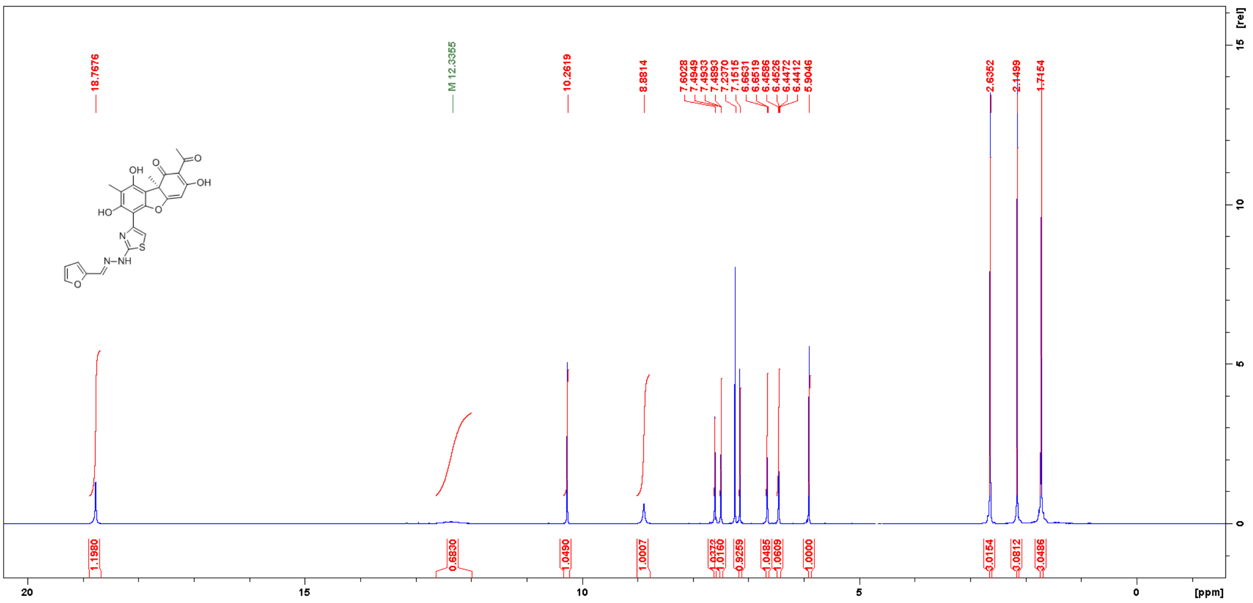

Figure S47:NMR ^{1}H spectra of **16k**

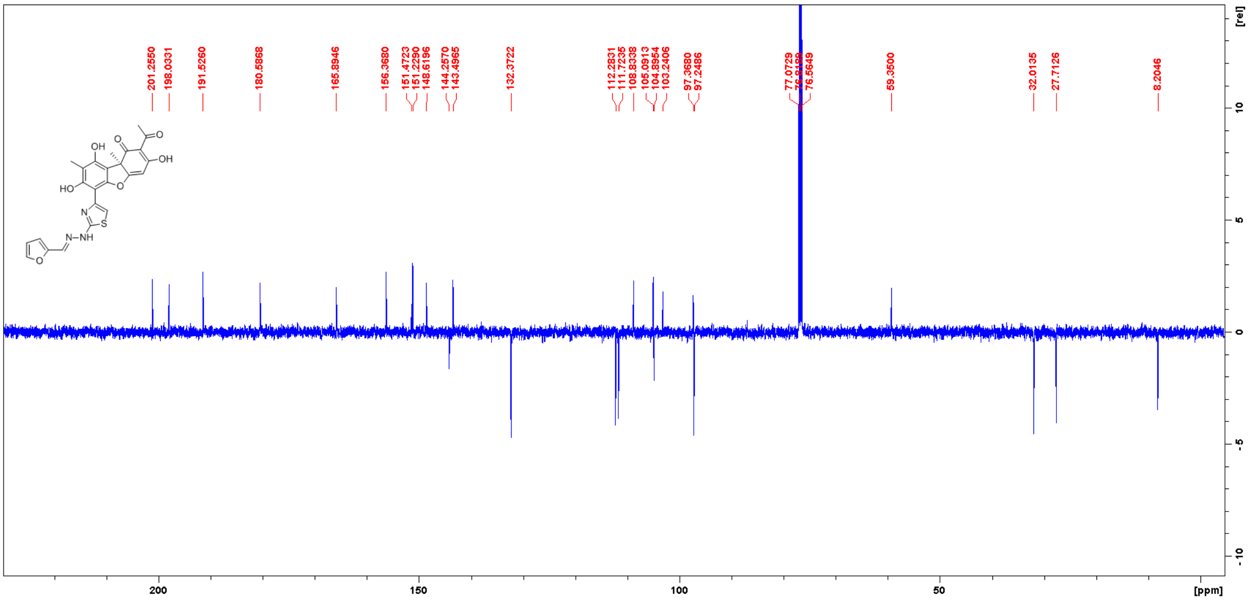

Figure S48:NMR ^{13}C (J-MOD) spectra of **16k**

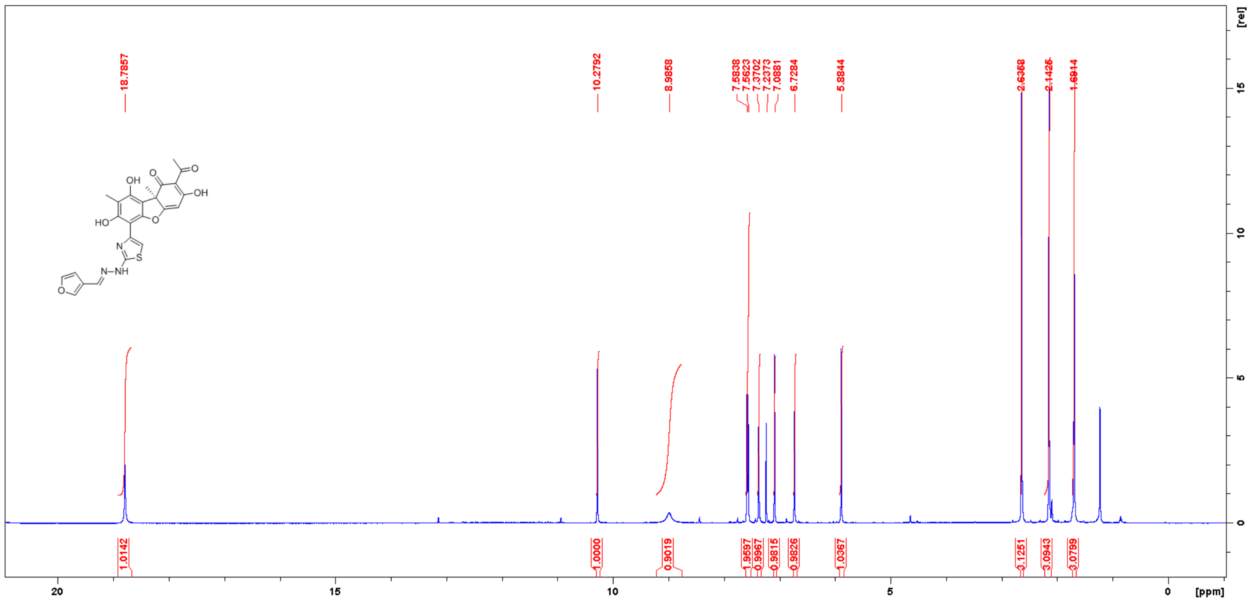

Figure S49:NMR ^{1}H spectra of **16l**

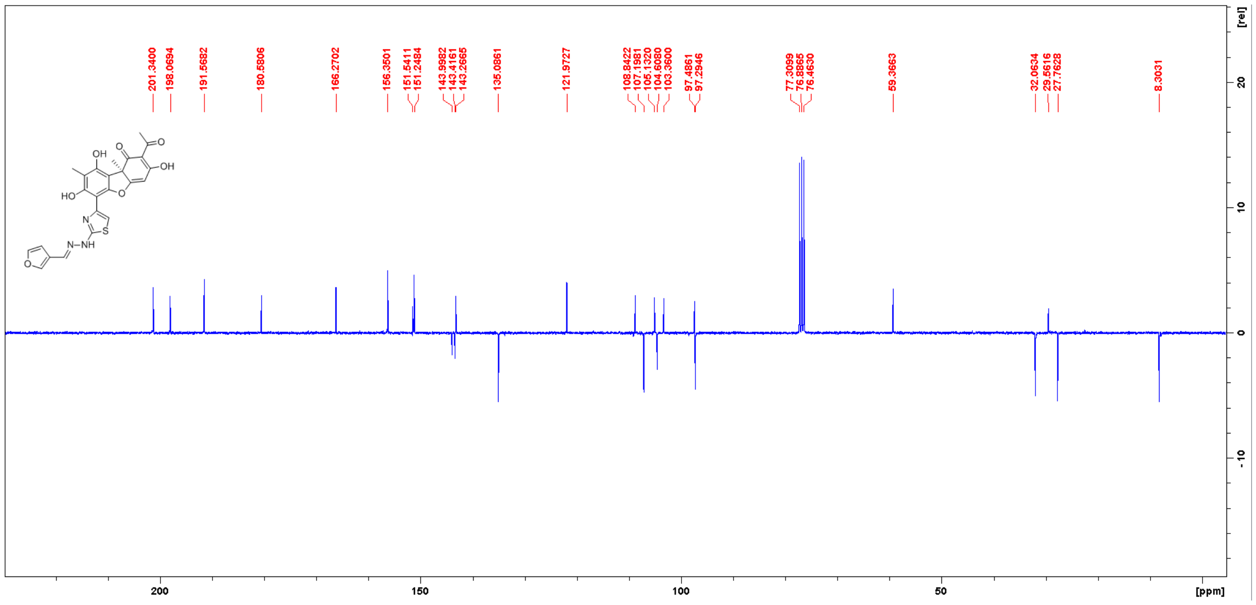

Figure S50:NMR ^{13}C (J-MOD) spectra of **16l**

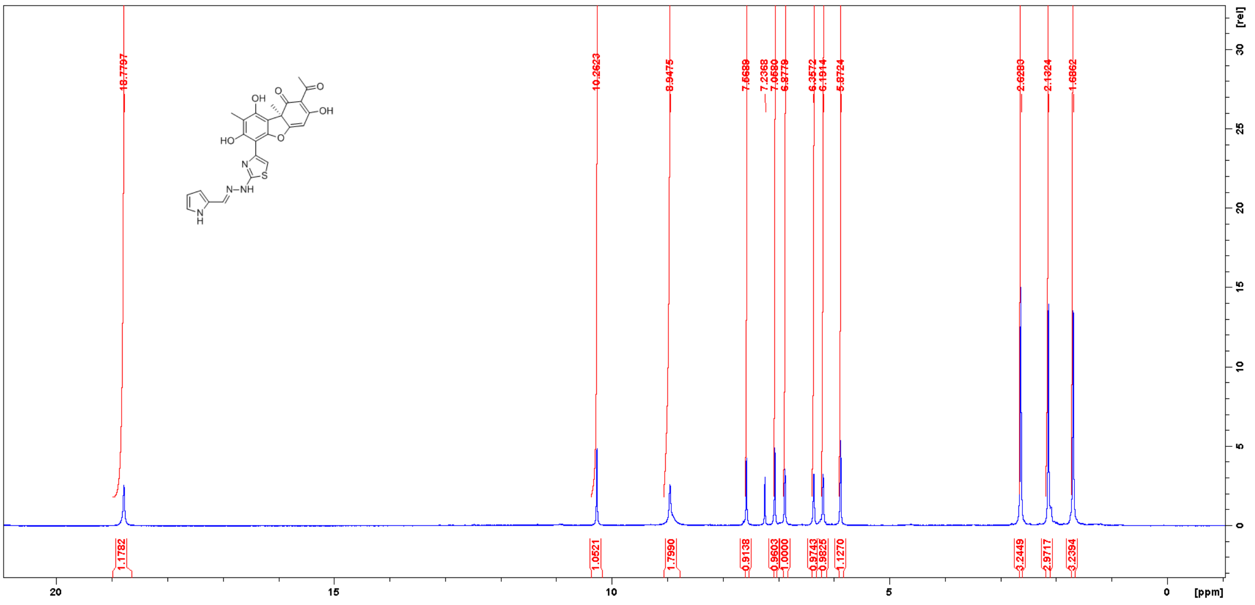

Figure S51:NMR ^{1}H spectra of **16m**

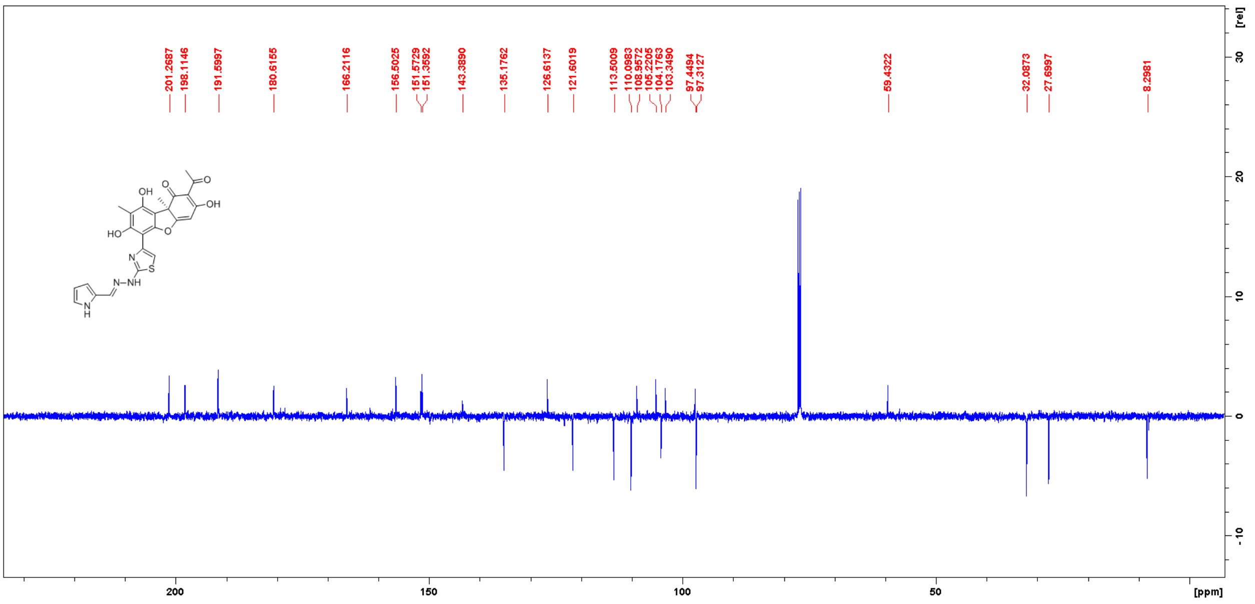

Figure S52:NMR ^{13}C (J-MOD) spectra of **16m**

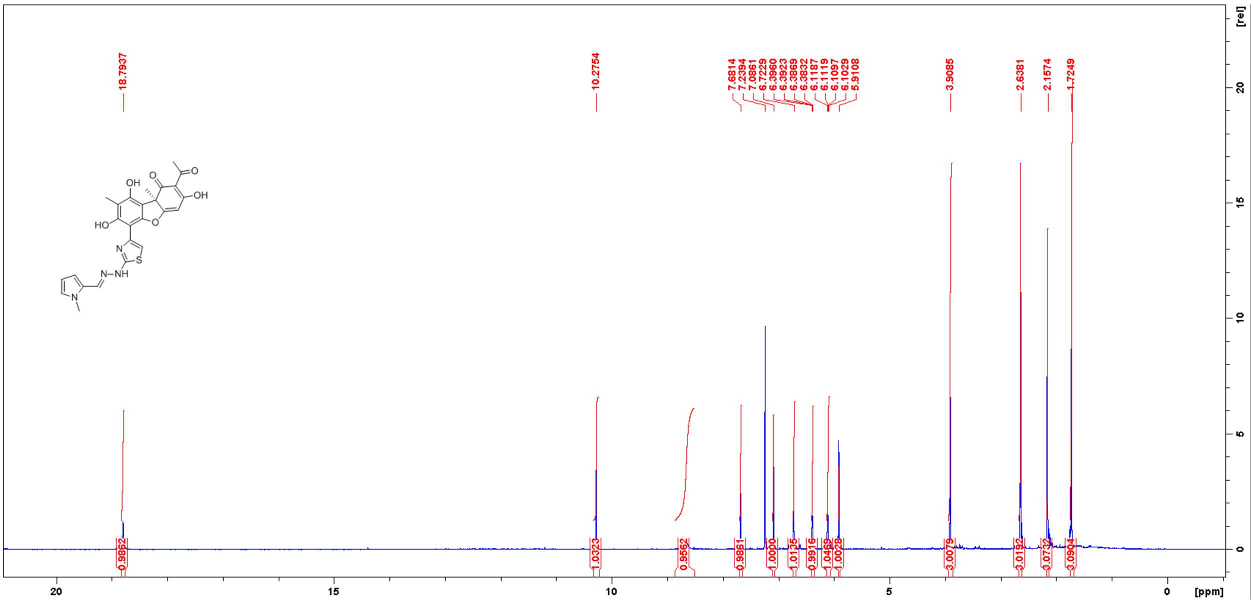

Figure S53:NMR ^{1}H spectra of **16n**

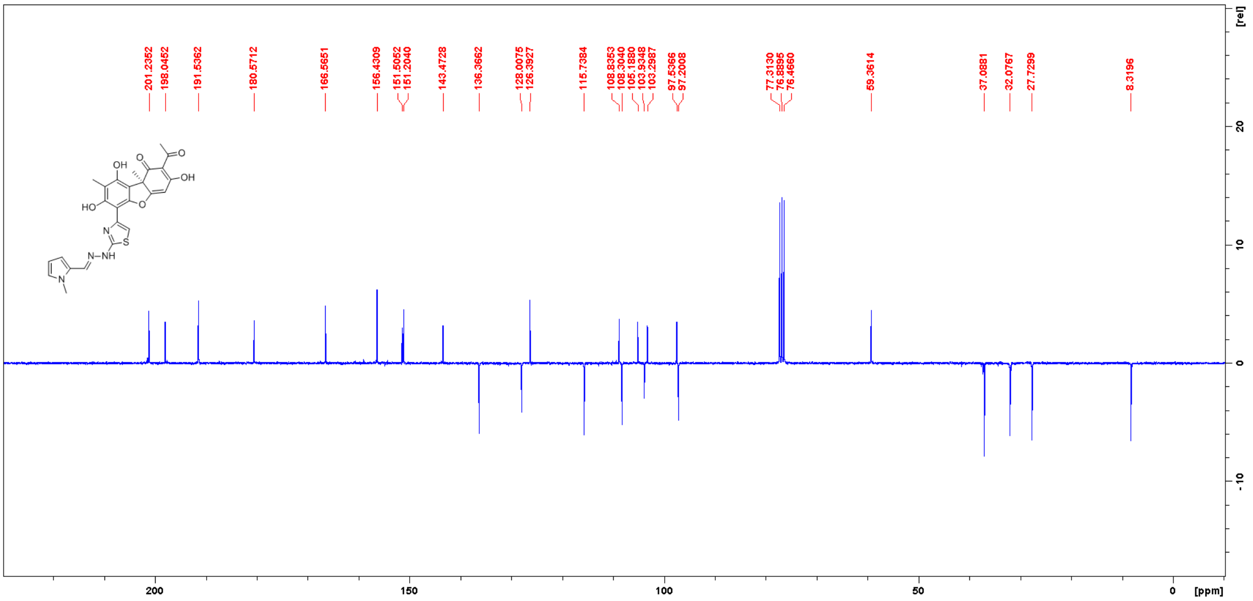

Figure S54:NMR ^{13}C (J-MOD) spectra of **16n**

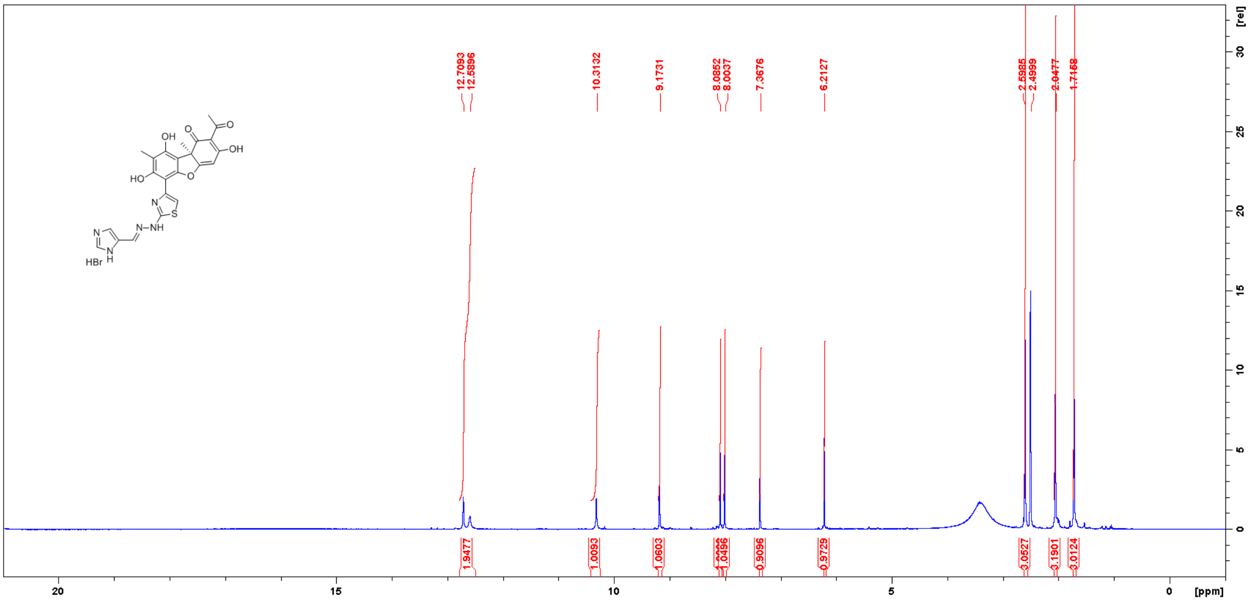

Figure S55:NMR ^{1}H spectra of **16o**

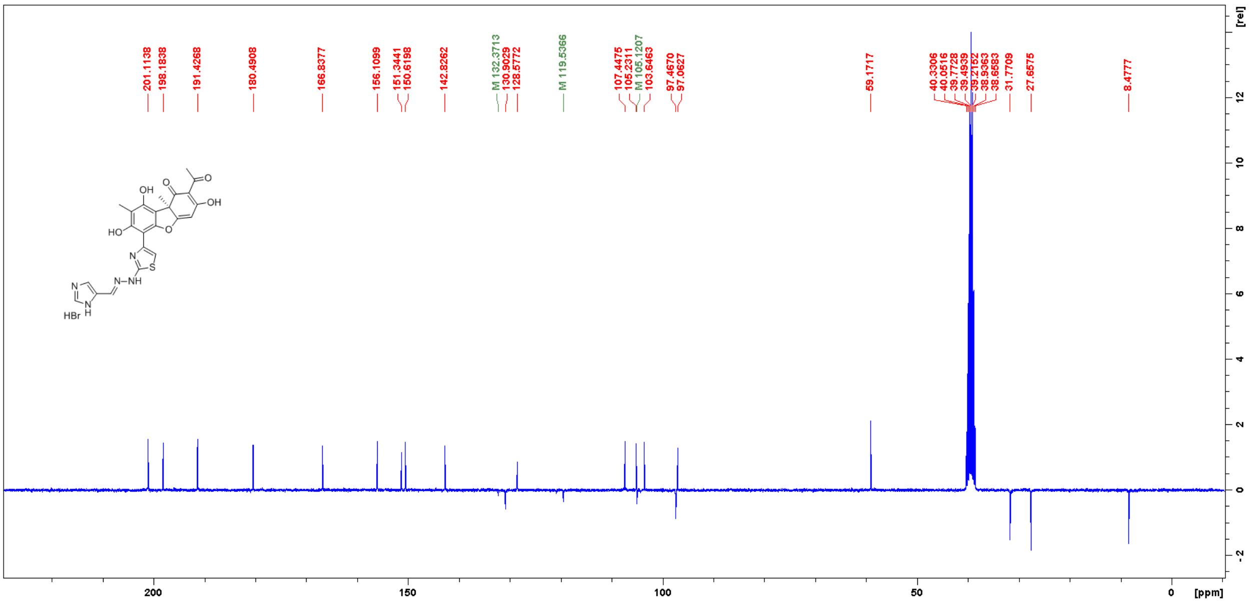

Figure S56:NMR ^{13}C (J-MOD) spectra of **16o**

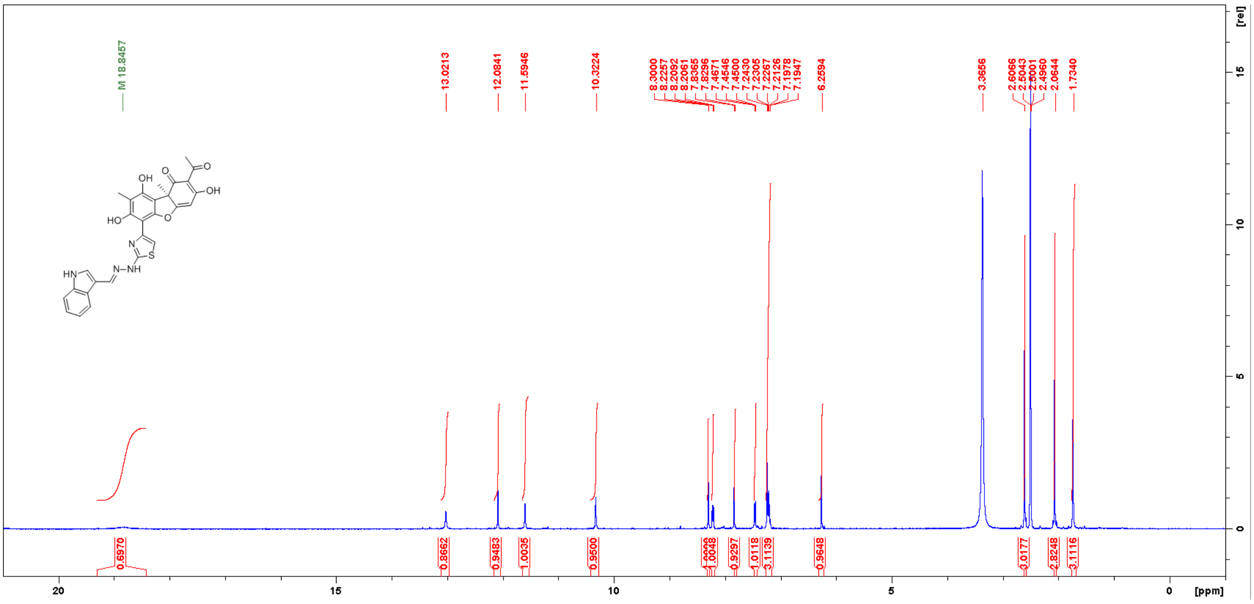

Figure S57:NMR ^{1}H spectra of **16p**

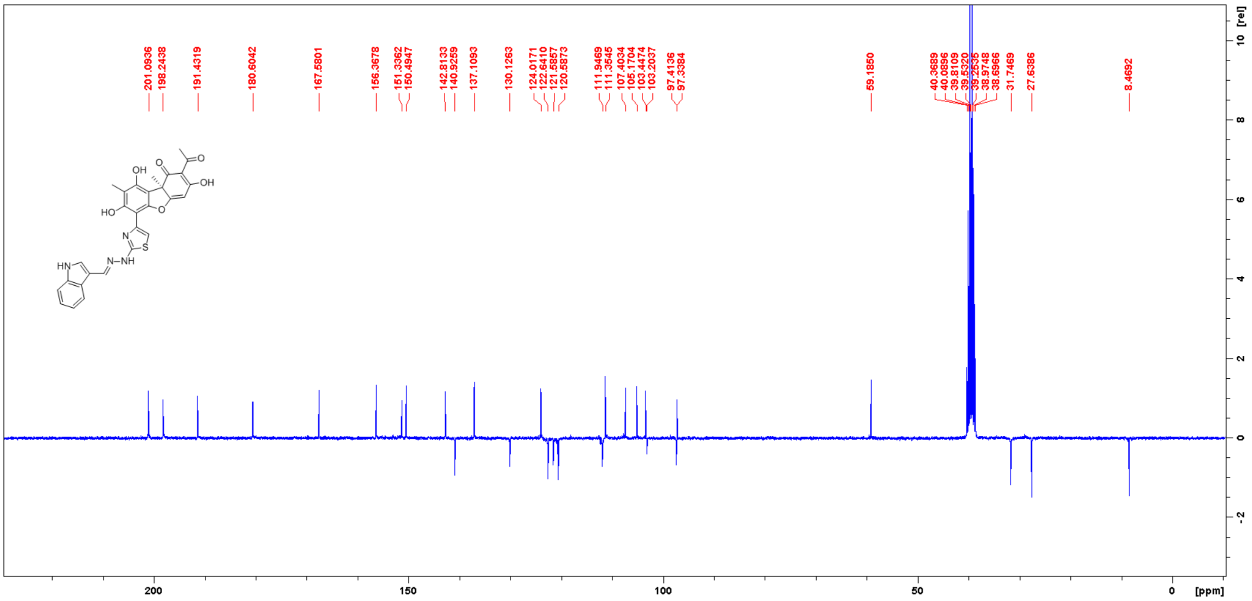

Figure S58:NMR ^{13}C (J-MOD) spectra of **16p**

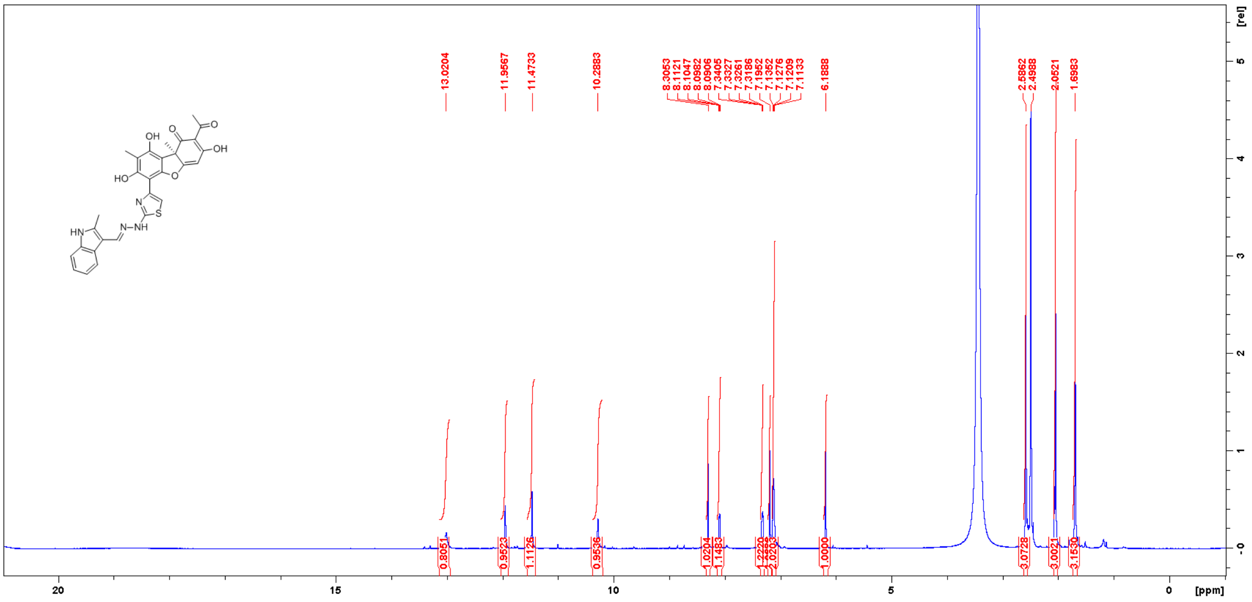

Figure S59:NMR ^{1}H spectra of **16q**

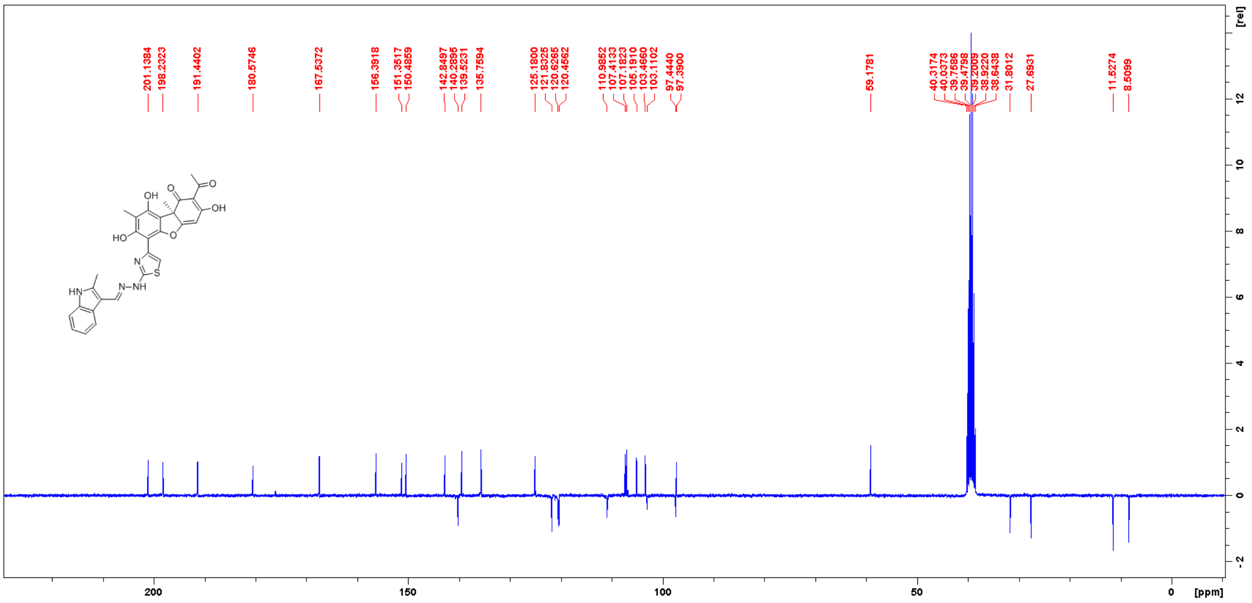

Figure S60:NMR ^{13}C (J-MOD) spectra of **16q**

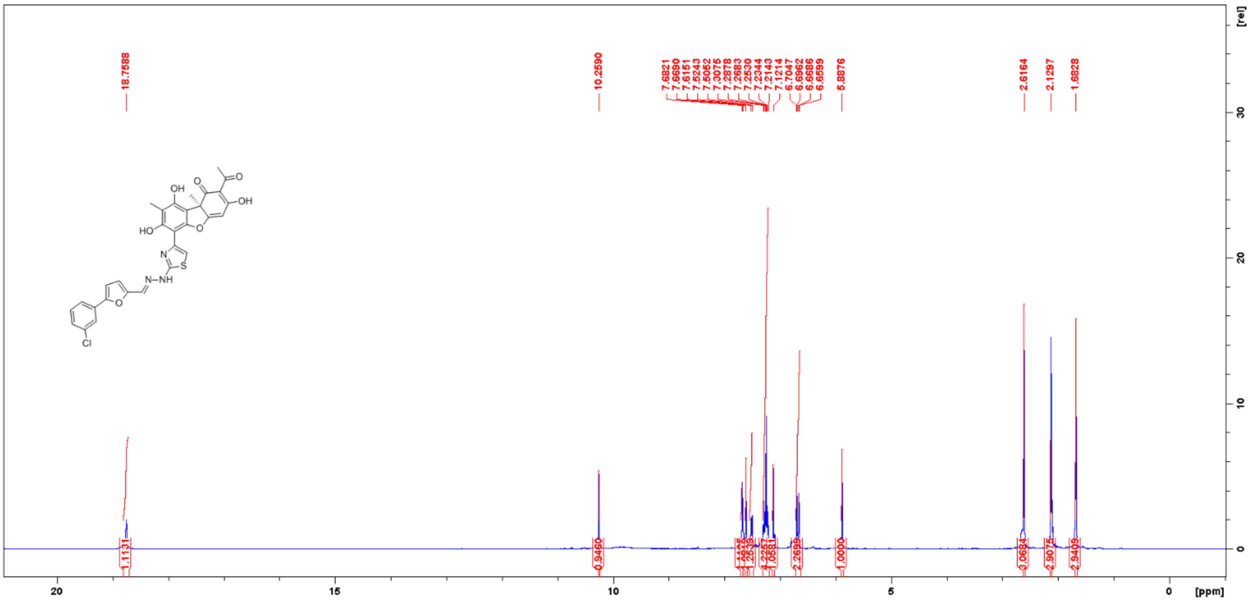

Figure S61:NMR ^{1}H spectra of **16r**

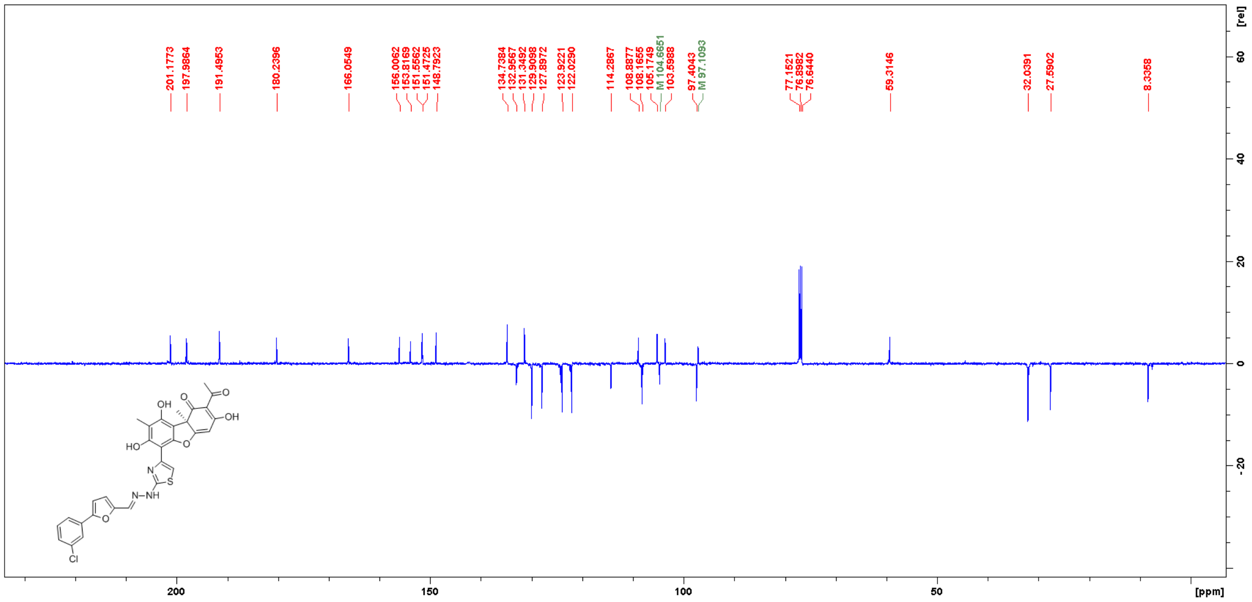

Figure S62:NMR ^{13}C (J-MOD) spectra of **16r**

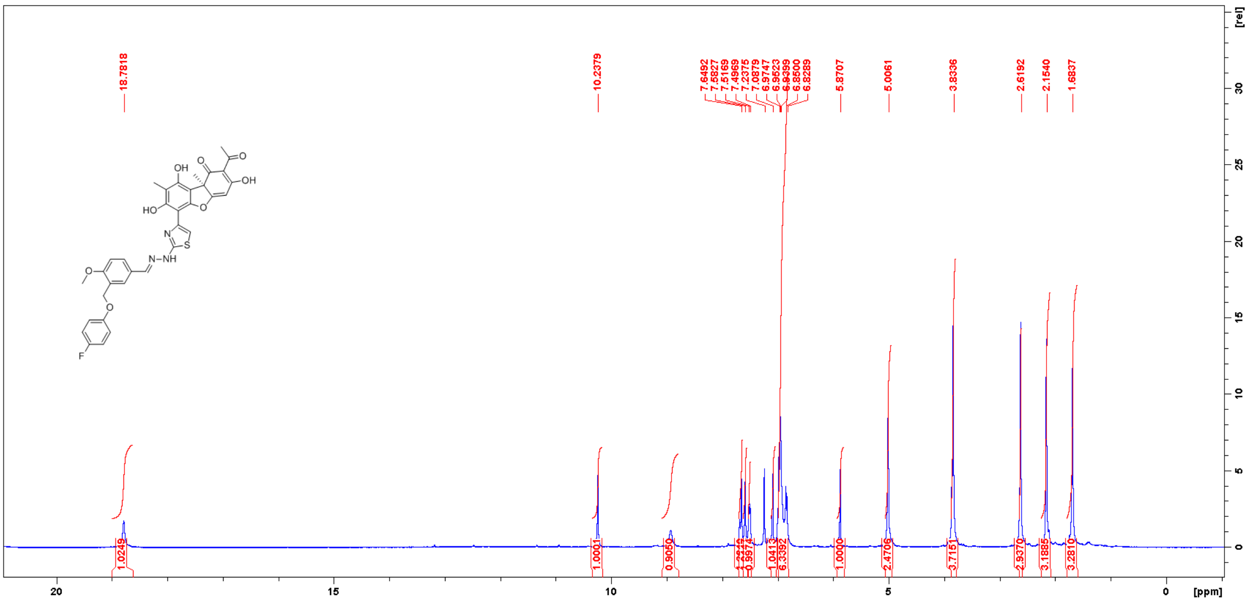

Figure S63: NMR ^{1}H spectra of **17a**

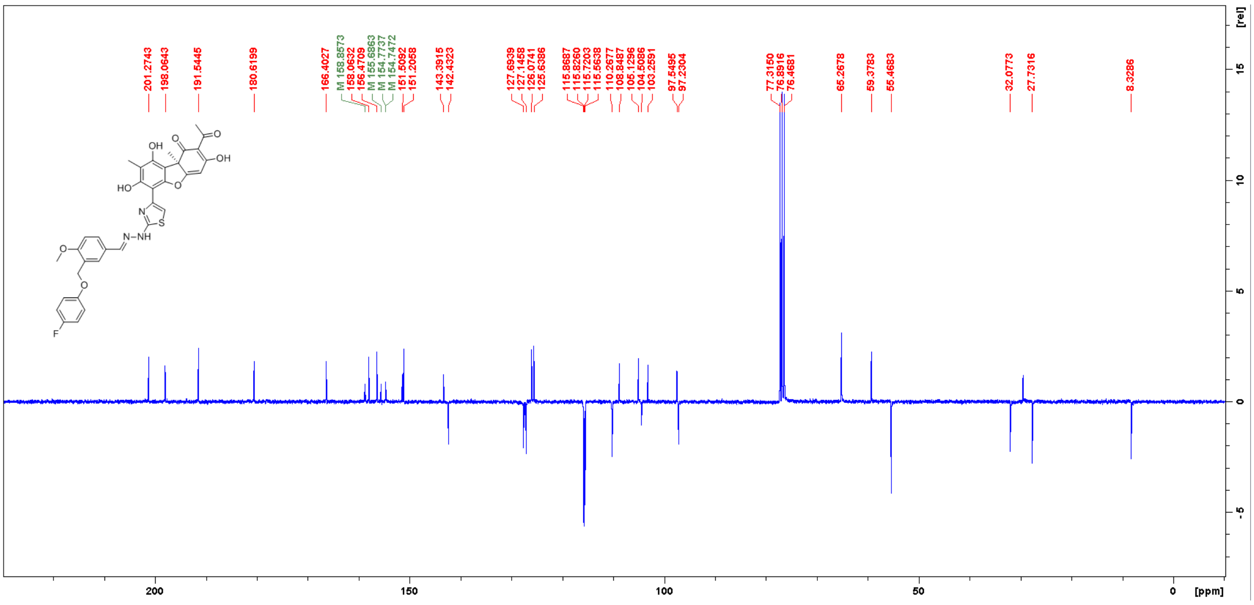

Figure S64: NMR ^{13}C (J-MOD) spectra of **17a**

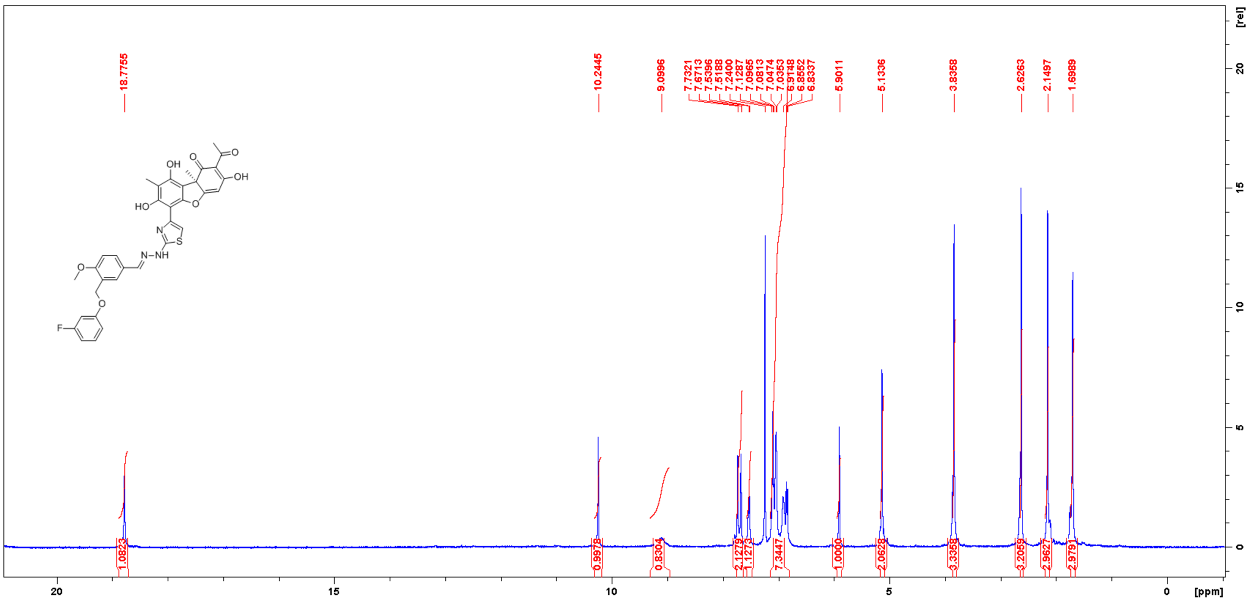

Figure S65: NMR ^{1}H spectra of **17b**

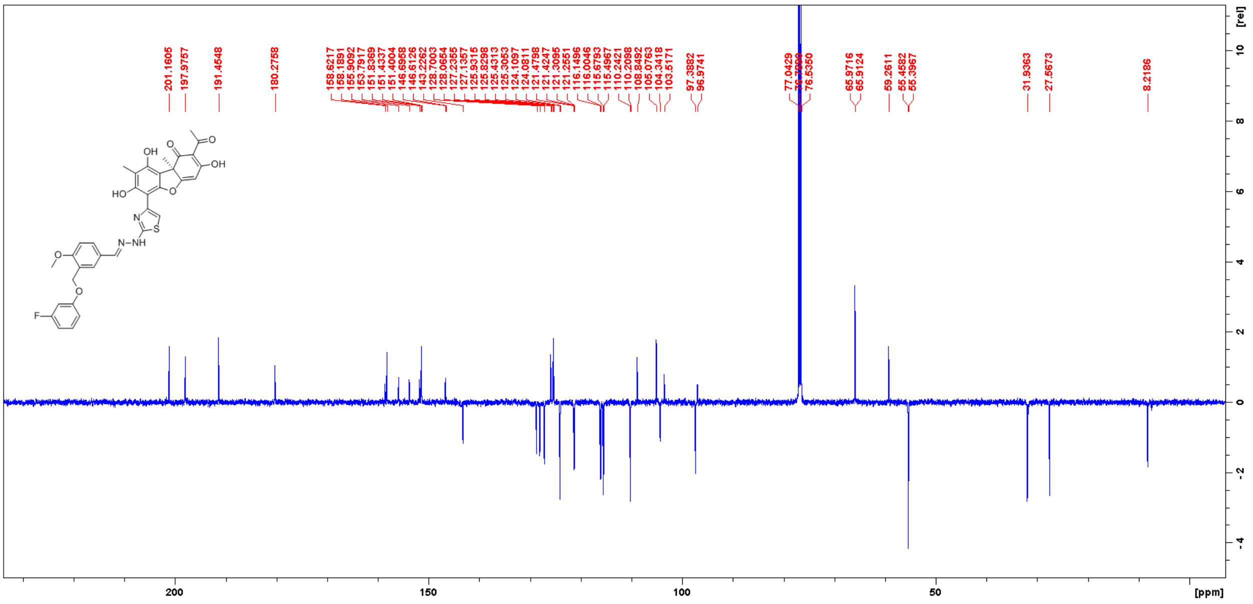

Figure S66: NMR ^{13}C (J-MOD) spectra of **17b**

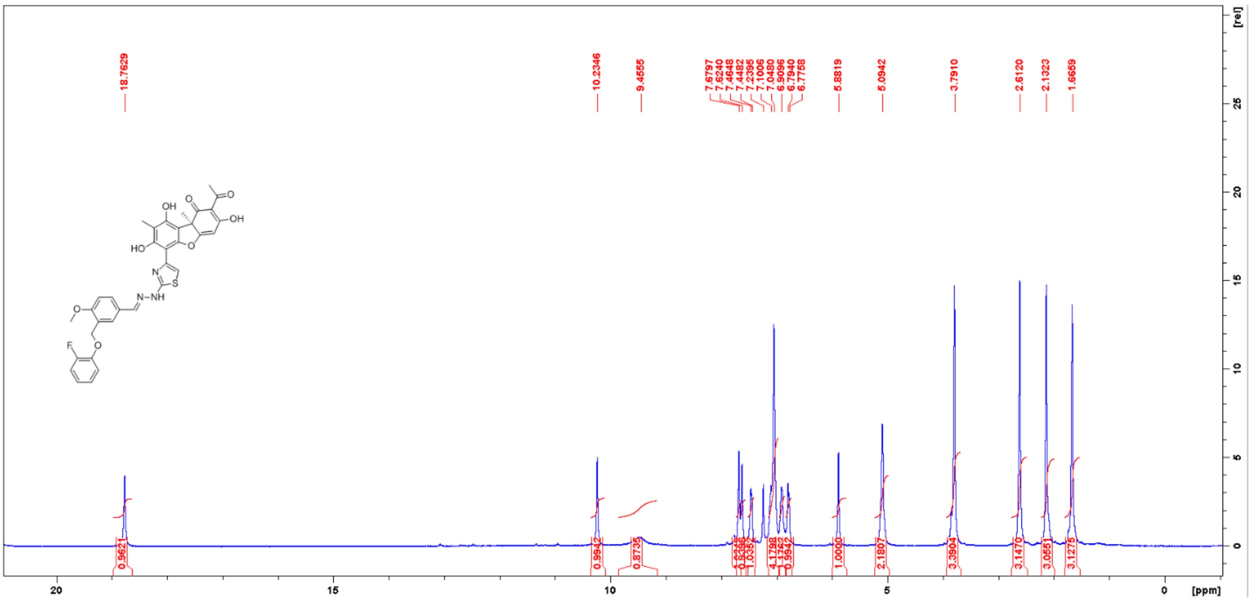

Figure S67: NMR ^{1}H spectra of **17c**

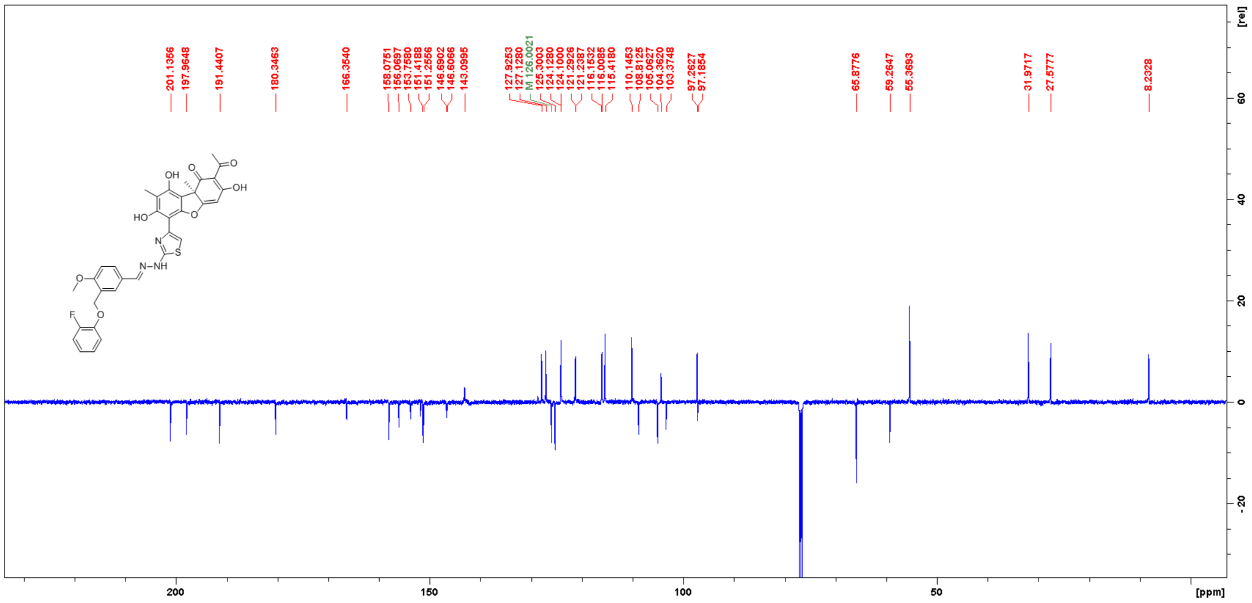

Figure S68: NMR ^{13}C (J-MOD) spectra of **17c**

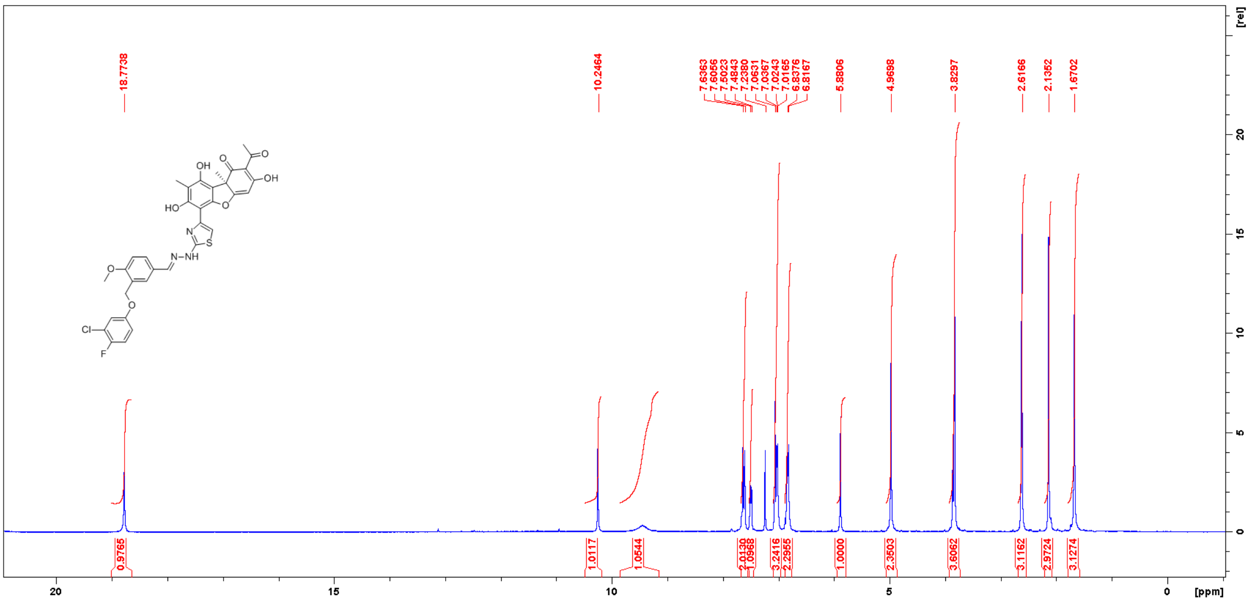

Figure S69: NMR ^{1}H spectra of **17d**

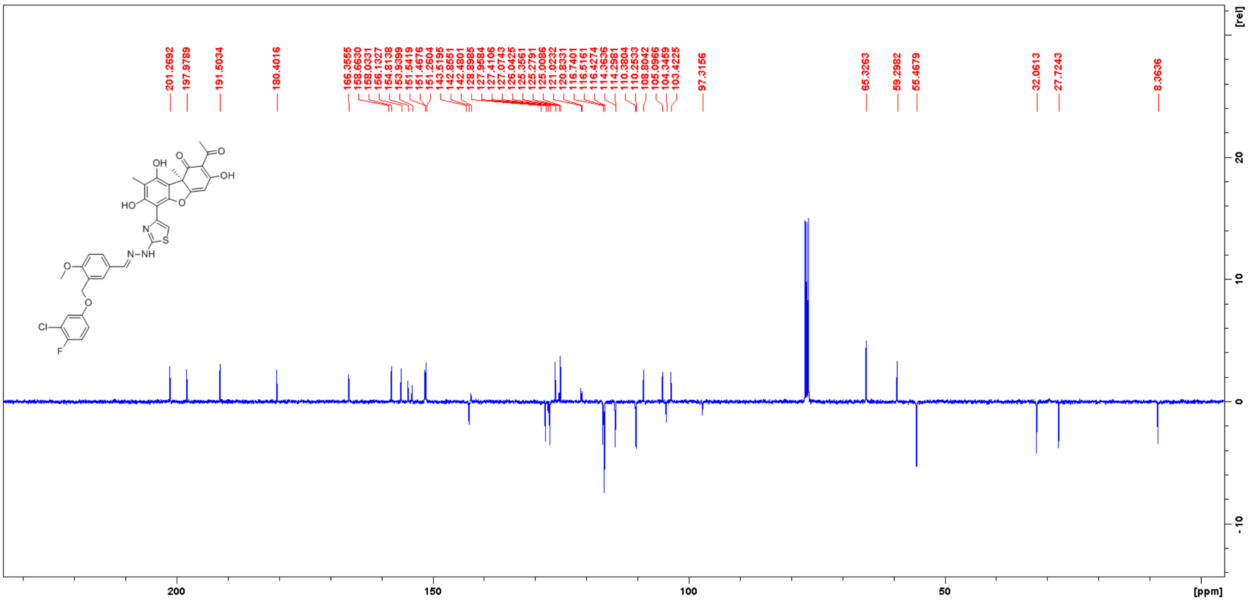

Figure S70: NMR ^{13}C (J-MOD) spectra of **17d**

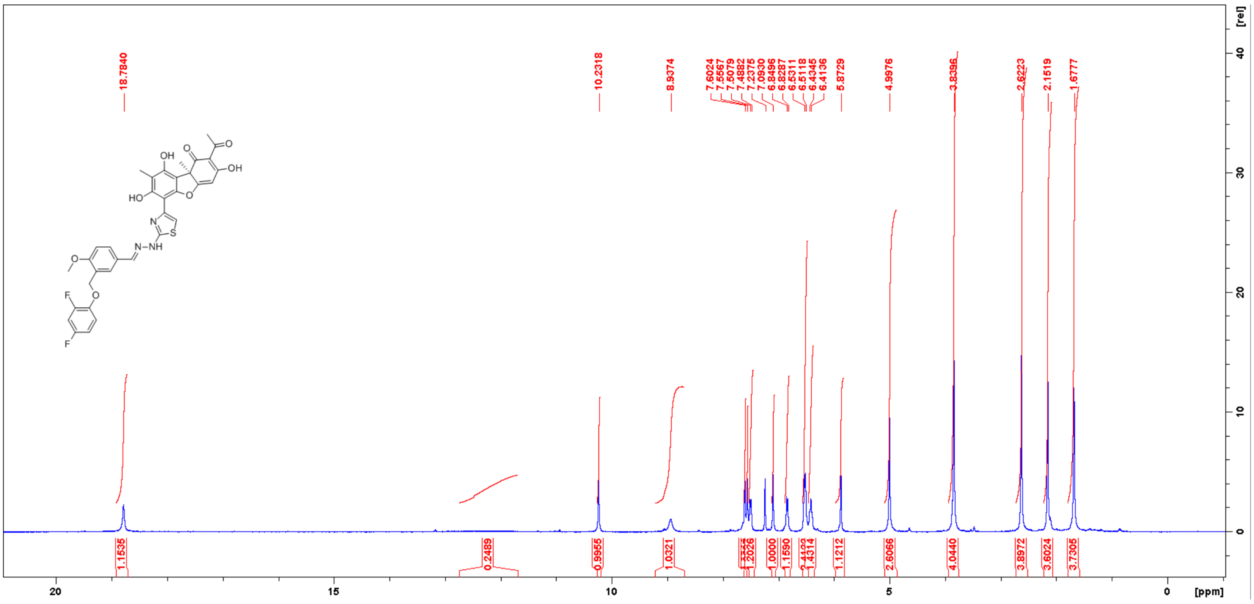

Figure S71: NMR ^{1}H spectra of **17e**

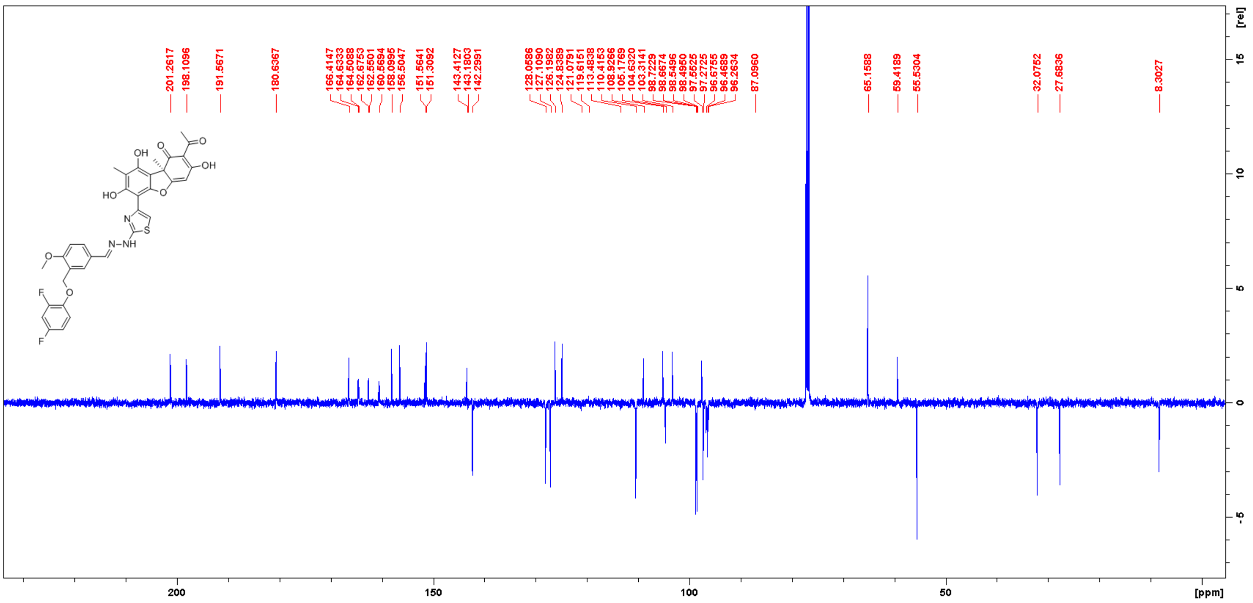

Figure S72: NMR ^{13}C (J-MOD) spectra of **17e**

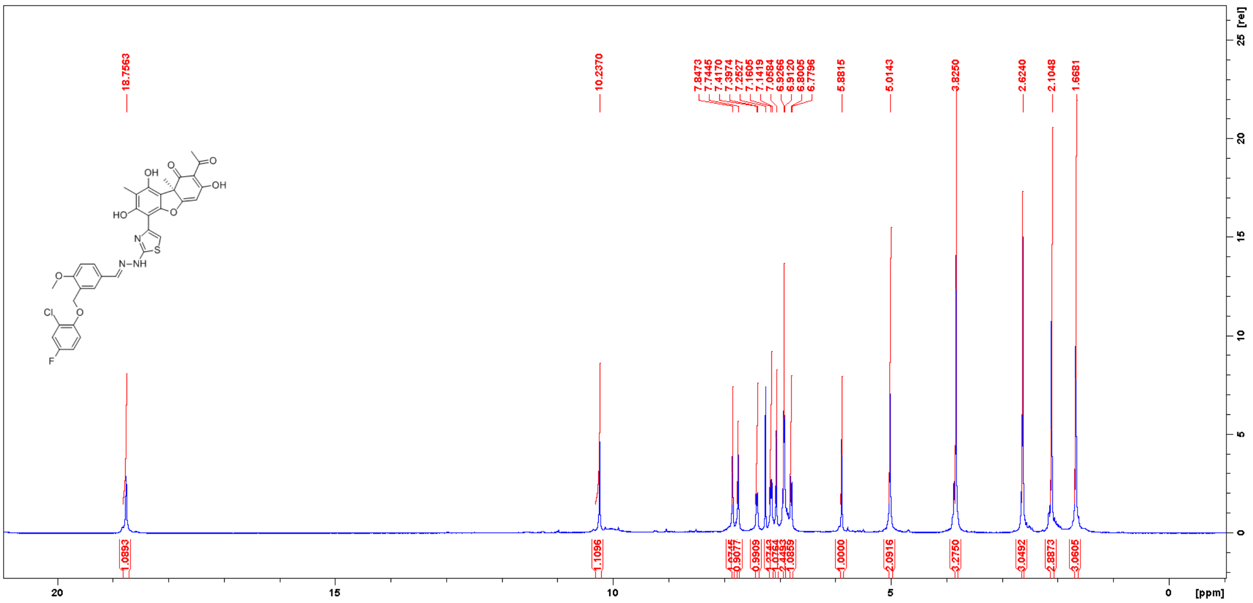

Figure S73: NMR ^{1}H spectra of **17f**

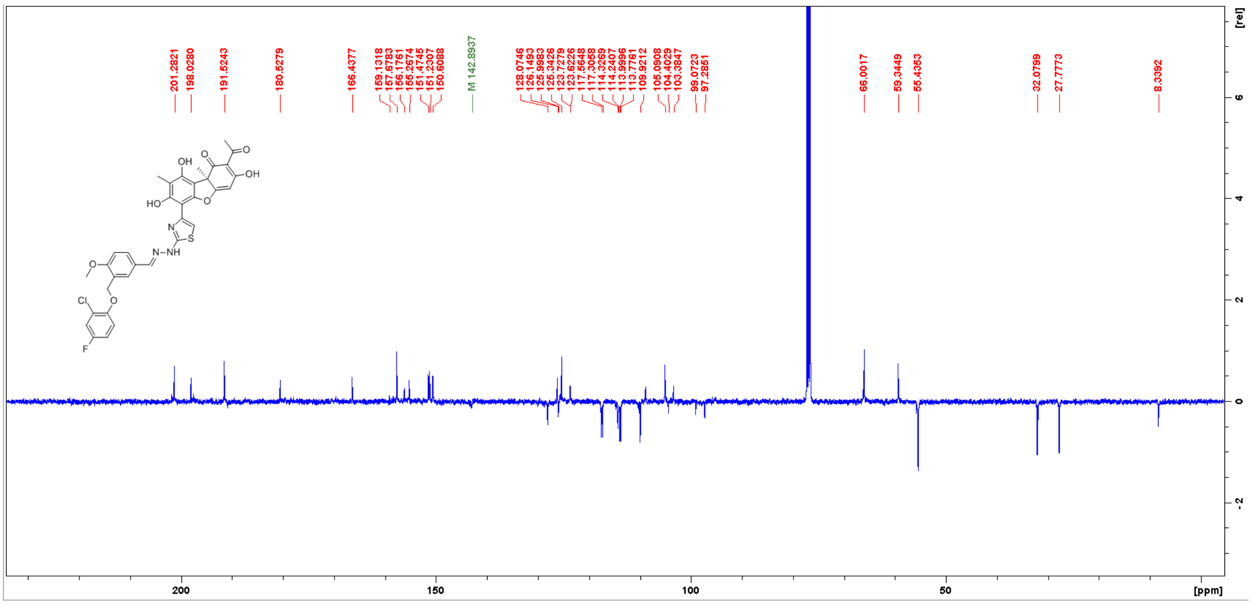

Figure S74: NMR ^{13}C (J-MOD) spectra of **17f**

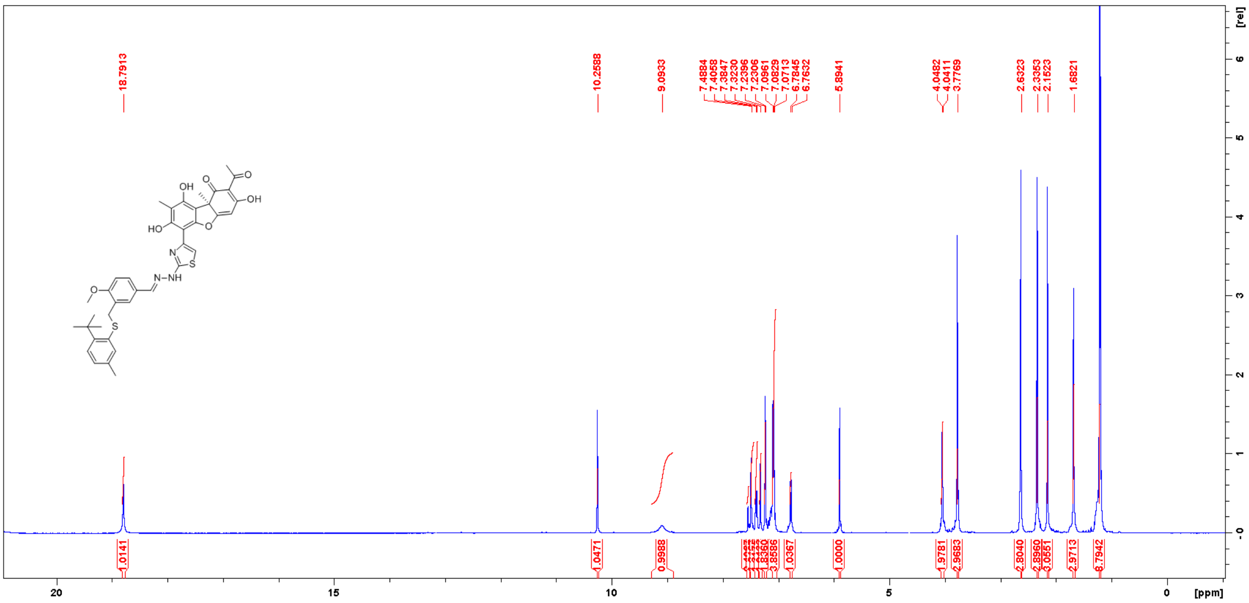

Figure S75: NMR ^{1}H spectra of **17g**

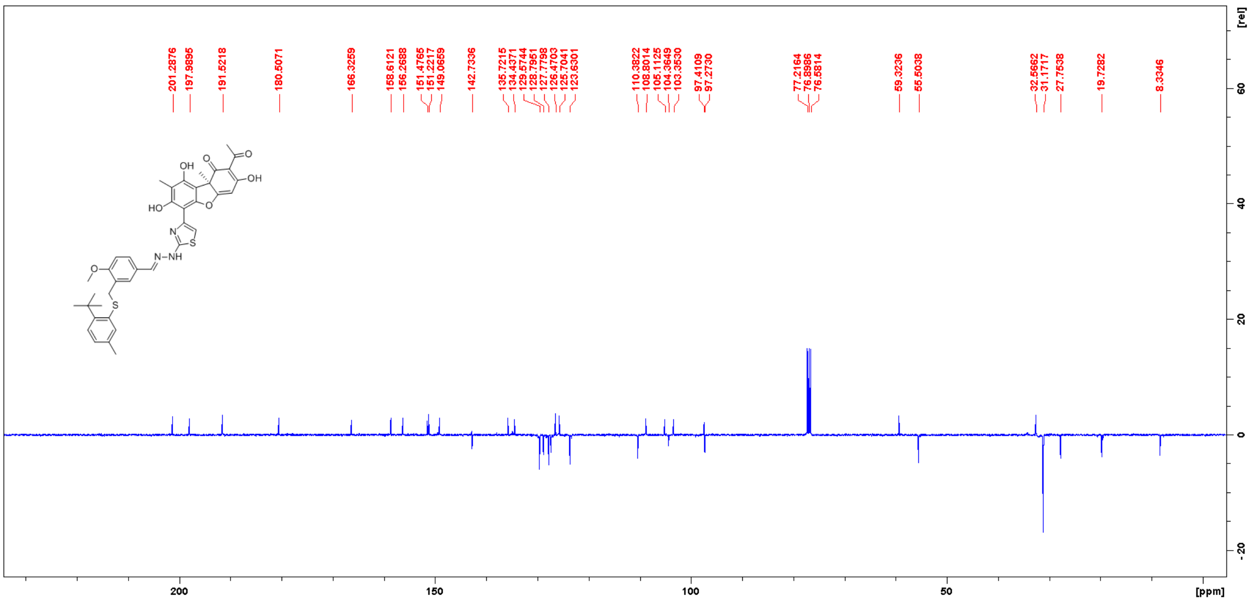

Figure S76: NMR ^{13}C (J-MOD) spectra of **17g**

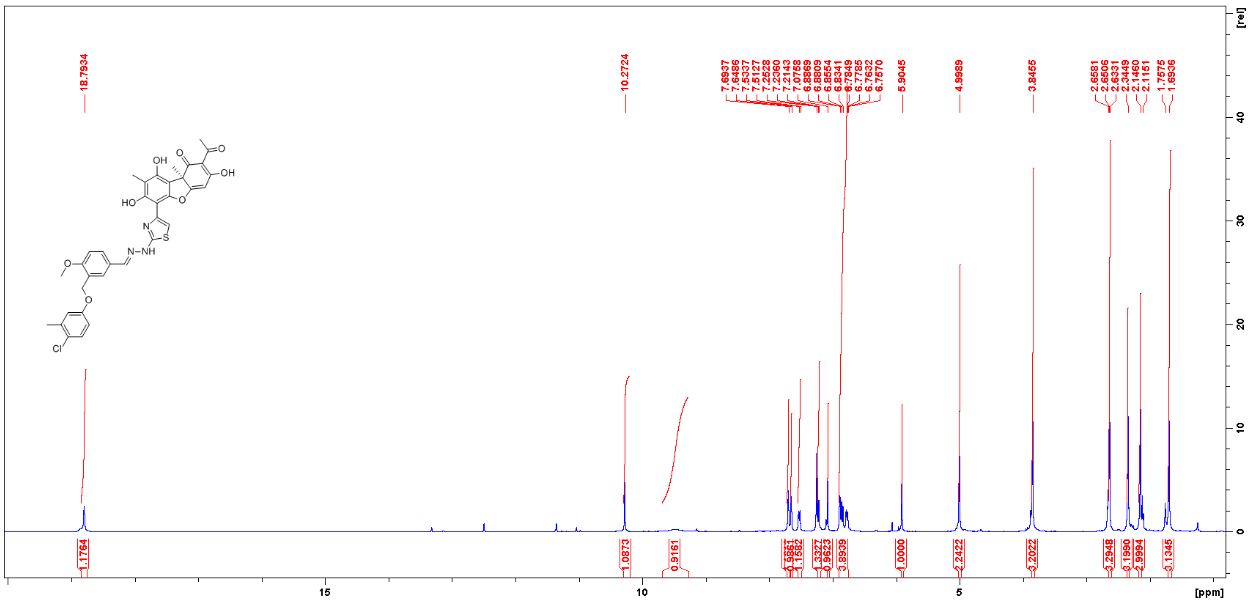

Figure S77: NMR ^{1}H spectra of **17h**

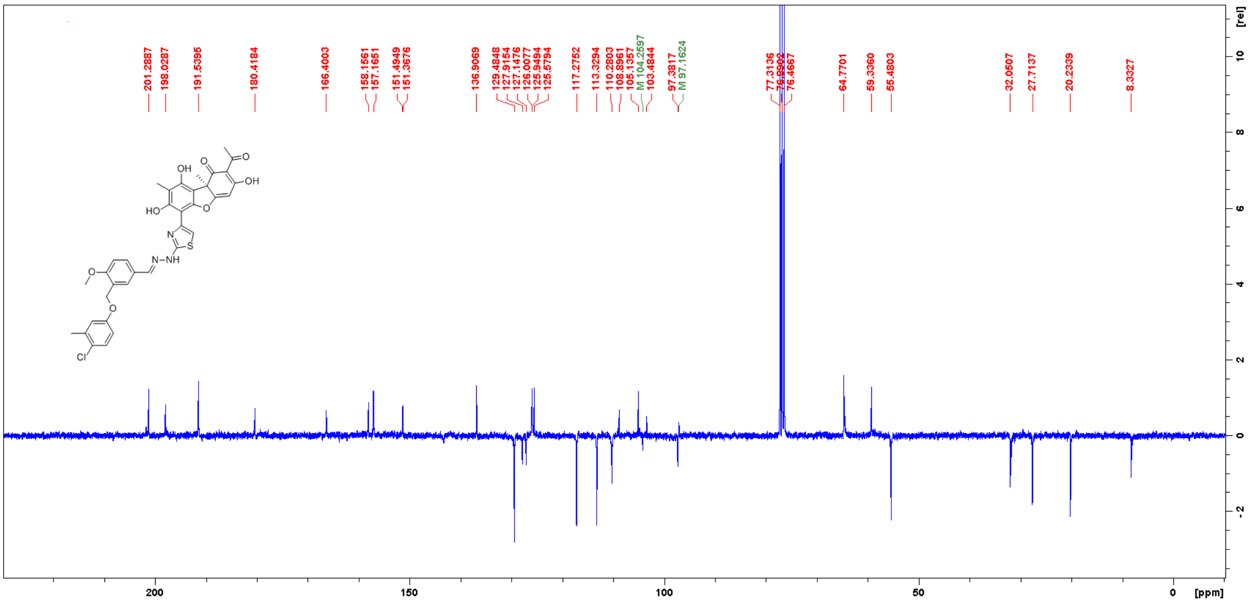

Figure S78: NMR ^{13}C (J-MOD) spectra of **17h**

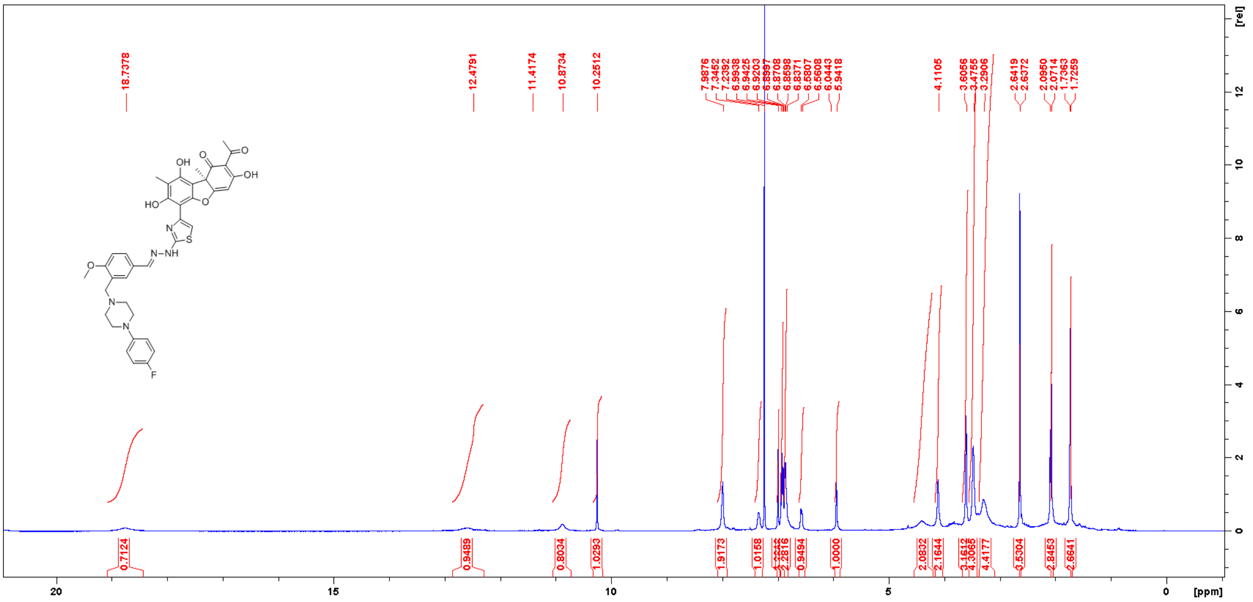

Figure S79: NMR ^{1}H spectra of **17i**

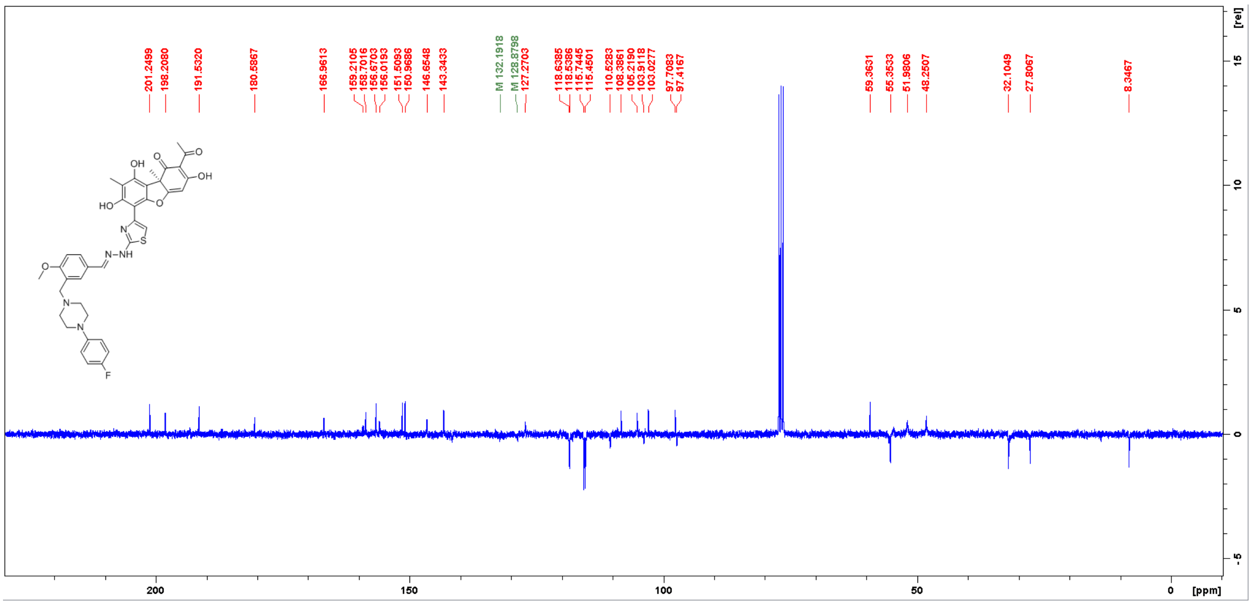

Figure S80: NMR ^{13}C (J-MOD) spectra of **17i**

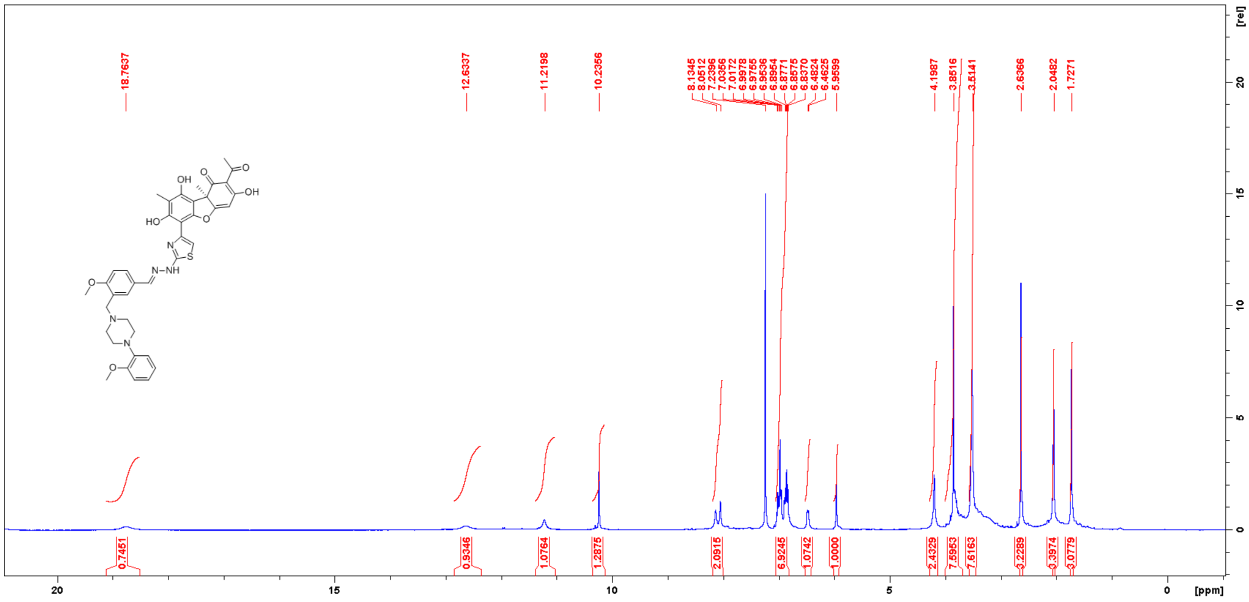

Figure S81: NMR ^{1}H spectra of **17j**

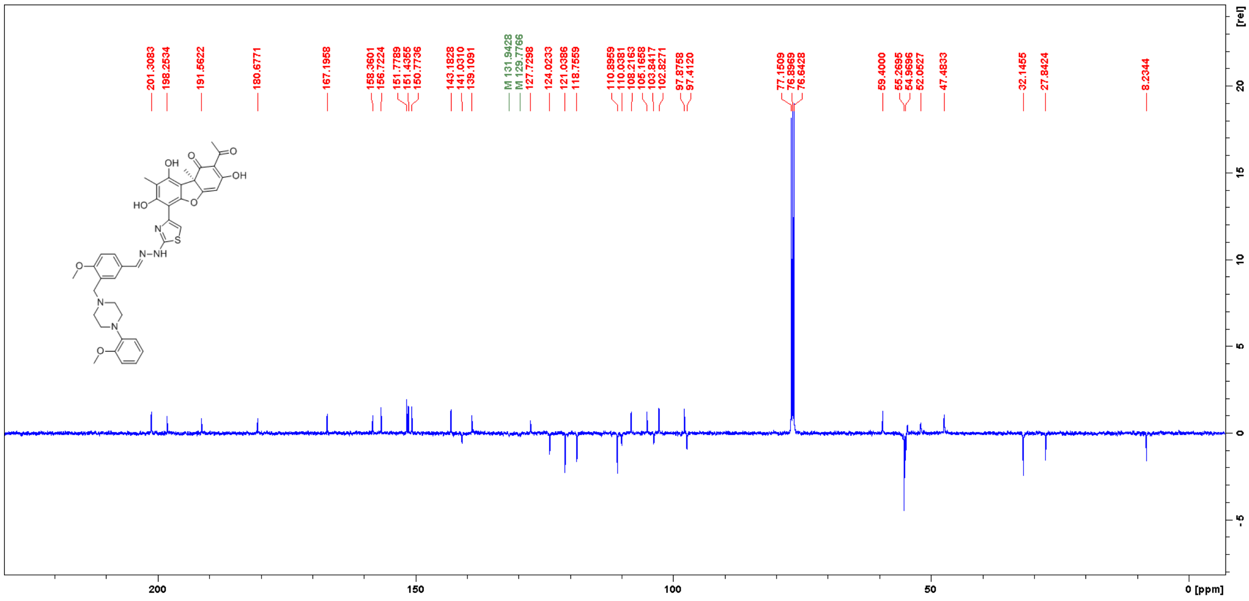

Figure S82: NMR ^{13}C (J-MOD) spectra of **17j**

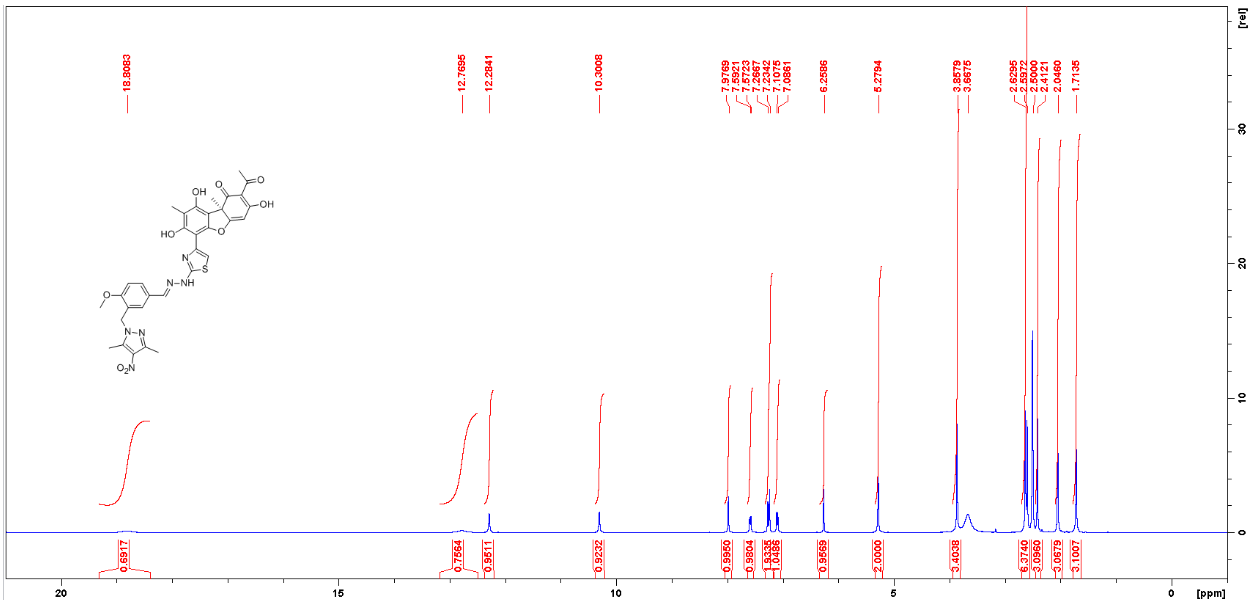

Figure S83: NMR ^{1}H spectra of **17k**

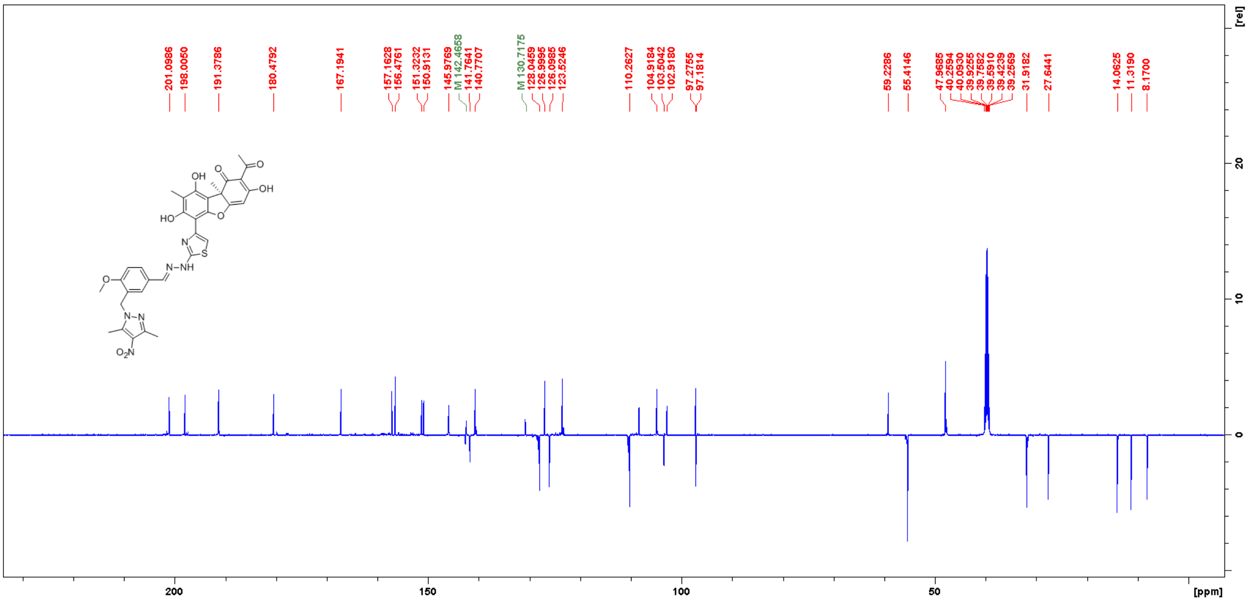

Figure S84:NMR ^{13}C (J-MOD) spectra of **17k**

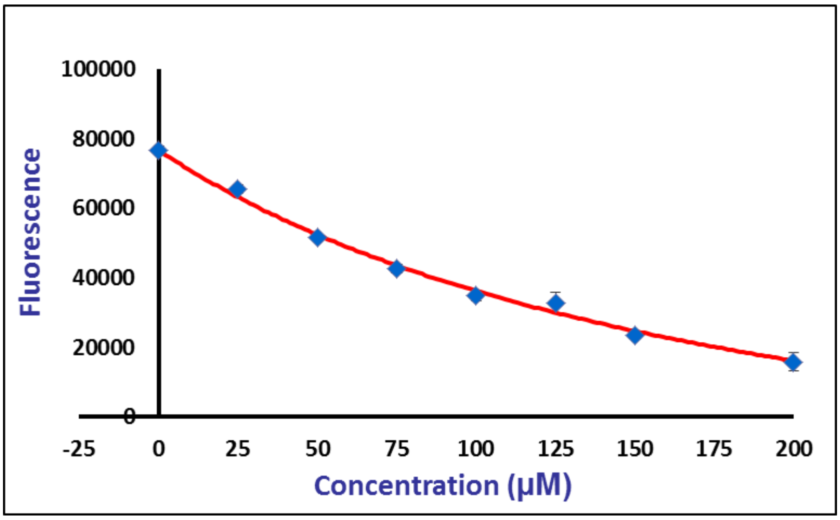

Figure S85: Changes in intrinsic fluorescence intensity of Tdp1 (10 µM) upon the addition of compound 16a(+) (25 µM, 50 µM, 75 µM, 100 µM, 125 µM, 150 µM and 200 µM). Buffer was 20 mM Tris and 250 mM NaCl (pH 8). Excitation wavelength was 280 nm and intrinsic fluorescence was measured at 350 nm. The K_D was 139 ± 32 µM. Experiment were conducted in triplicate.

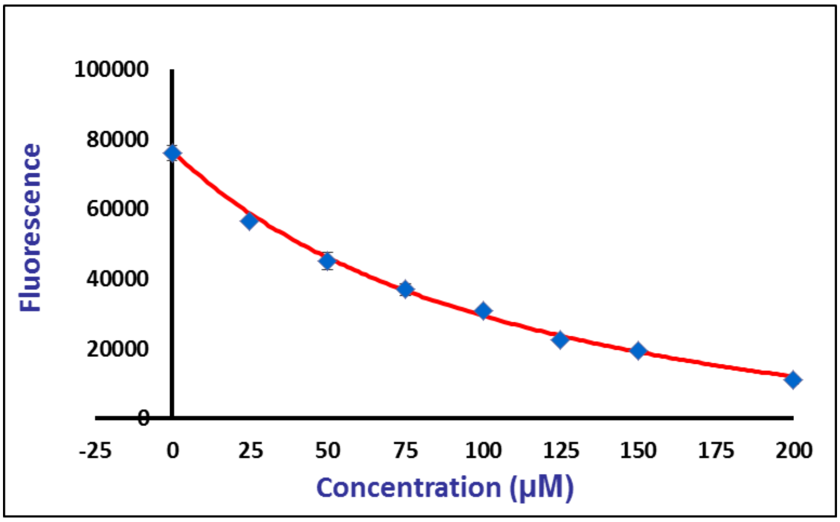

Figure S86: Changes in intrinsic fluorescence intensity of Tdp1 (10 µM) upon the addition of compound 16b(+) (25 µM, 50 µM, 75 µM, 100 µM, 125 µM, 150 µM and 200 µM). Buffer was 20 mM Tris and 250 mM NaCl (pH 8). Excitation wavelength was 280 nm and intrinsic fluorescence was measured at 350 nm. The K_D was 77 ± 24 µM. Experiment were conducted in triplicate.

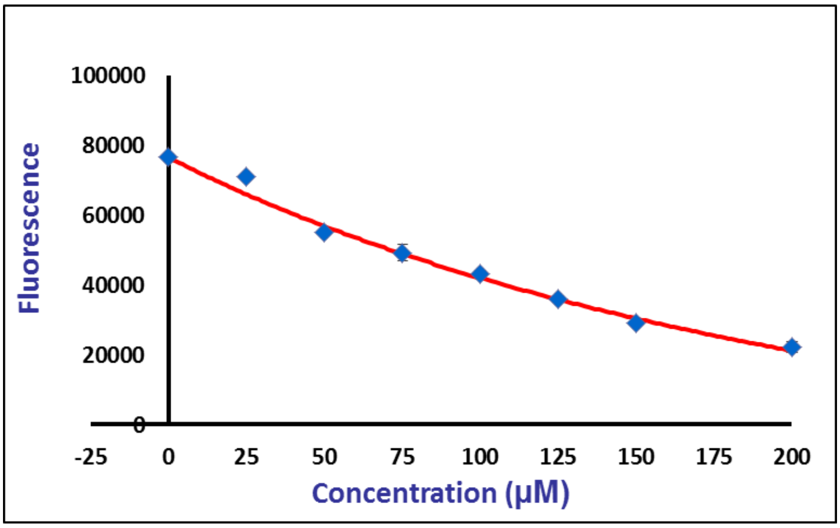

Figure S87: Changes in intrinsic fluorescence intensity of Tdp1 (10 μM) upon the addition of compound 16c(-) (25 μM, 50 μM, 75 μM, 100 μM, 125 μM, 150 μM and 200 μM). Buffer was 20 mM Tris and 250 mM NaCl (pH 8). Excitation wavelength was 280 nm and intrinsic fluorescence was measured at 350 nm. The K_D was 262 ± 25 μM. Experiment were conducted in triplicate.

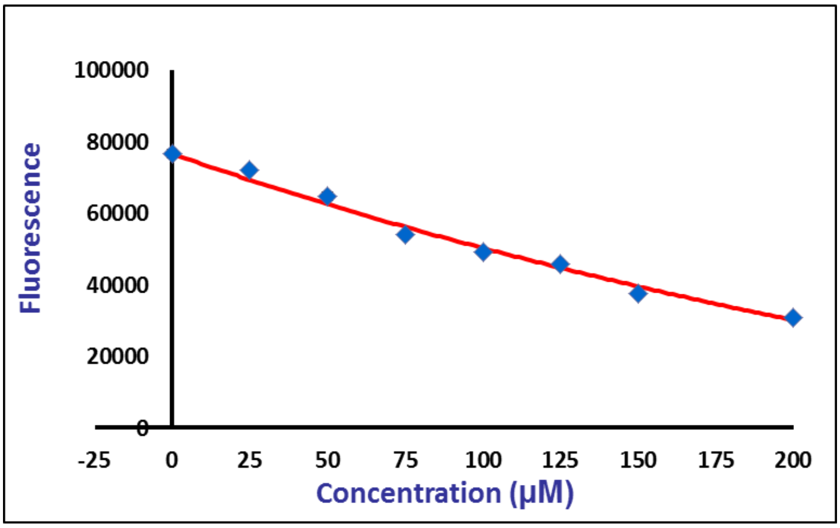

Figure S88: Changes in intrinsic fluorescence intensity of Tdp1 (10 µM) upon the addition of compound 16d(+) (25 µM, 50 µM, 75 µM, 100 µM, 125 µM, 150 µM and 200 µM). Buffer was 20 mM Tris and 250 mM NaCl (pH 8). Excitation wavelength was 280 nm and intrinsic fluorescence was measured at 350 nm. The K_D was 597 ± 98 µM. Experiment were conducted in triplicate.

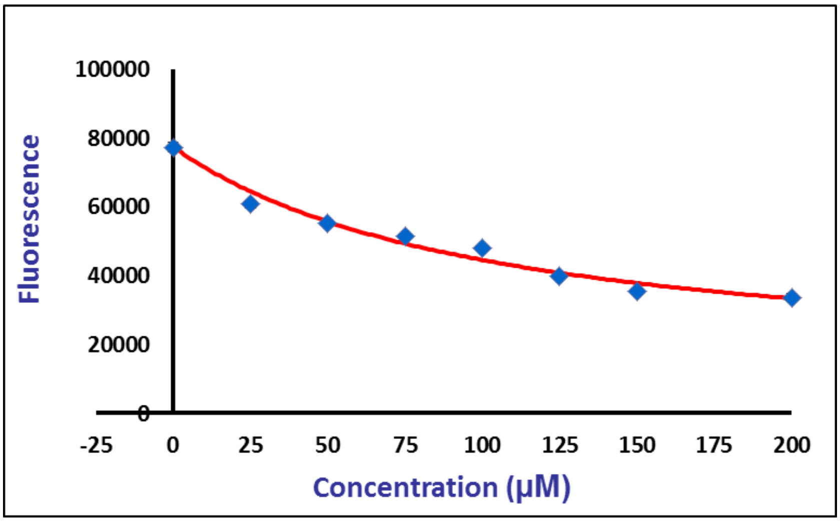

Figure S 89: Changes in intrinsic fluorescence intensity of Tdp1 (10 µM) upon the addition of compound 16f(+) (25 µM, 50 µM, 75 µM, 100 µM, 125 µM, 150 µM and 200 µM). Buffer was 20 mM Tris and 250 mM NaCl (pH 8). Excitation wavelength was 280 nm and intrinsic fluorescence was measured at 350 nm. The K_D was 95 ± 11 µM. Experiment were conducted in triplicate.

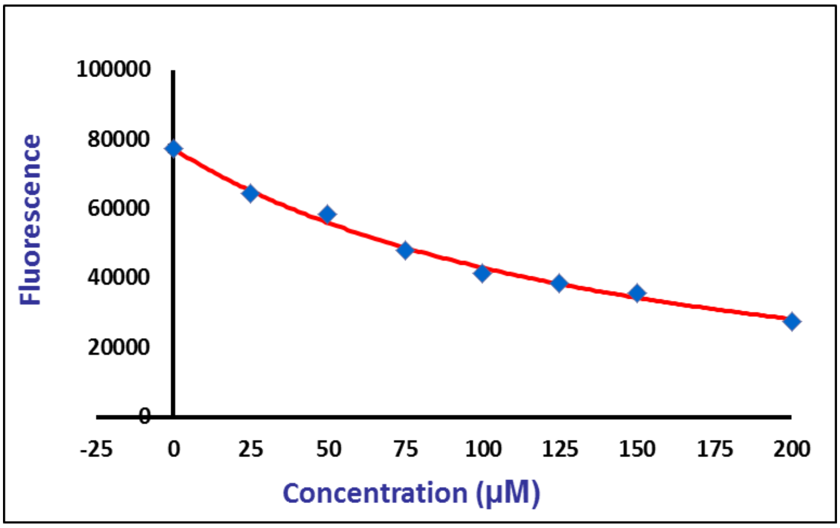

Figure S90: Changes in intrinsic fluorescence intensity of Tdp1 (10 μM) upon the addition of compound 16f(-) (25 μM, 50 μM, 75 μM, 100 μM, 125 μM, 150 μM and 200 μM). Buffer was 20 mM Tris and 250 mM NaCl (pH 8). Excitation wavelength was 280 nm and intrinsic fluorescence was measured at 350 nm. The K_D was 148 ± 9 μM. Experiment were conducted in triplicate.

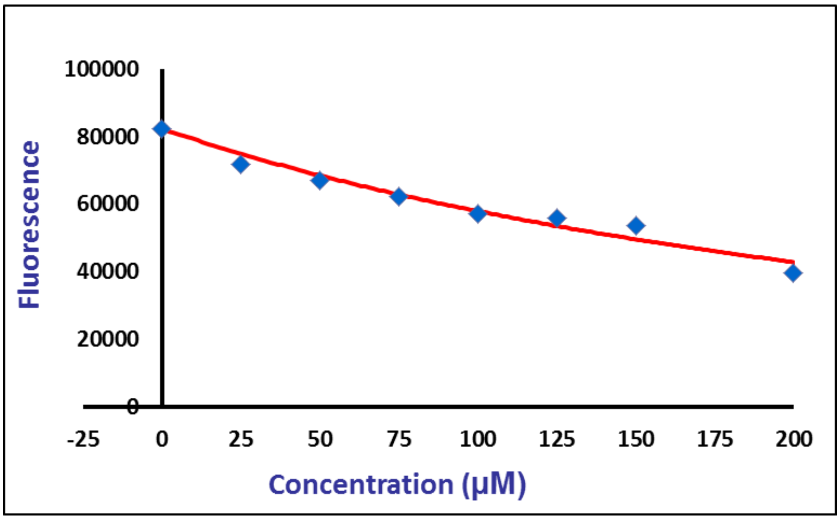

Figure S91: Changes in intrinsic fluorescence intensity of Tdp1 (10 µM) upon the addition of compound 16g(-) (25 µM, 50 µM, 75 µM, 100 µM, 125 µM, 150 µM and 200 µM). Buffer was 20 mM Tris and 250 mM NaCl (pH 8). Excitation wavelength was 280 nm and intrinsic fluorescence was measured at 350 nm. The K_D was 281 ± 25 µM. Experiment were conducted in triplicate.

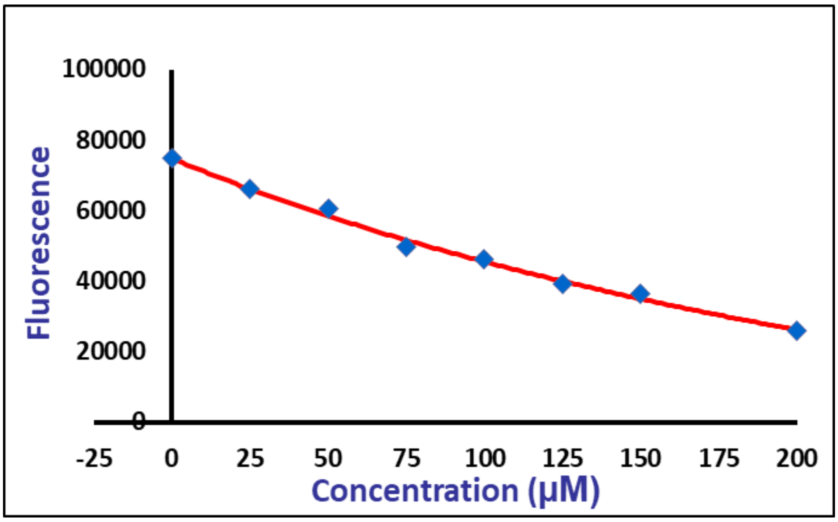

Figure S92: Changes in intrinsic fluorescence intensity of Tdp1 (10 µM) upon the addition of compound 16h(-) (25 µM, 50 µM, 75 µM, 100 µM, 125 µM, 150 µM and 200 µM). Buffer was 20 mM Tris and 250 mM NaCl (pH 8). Excitation wavelength was 280 nm and intrinsic fluorescence was measured at 350 nm. The K_D was 355 ± 50 µM. Experiment were conducted in triplicate.

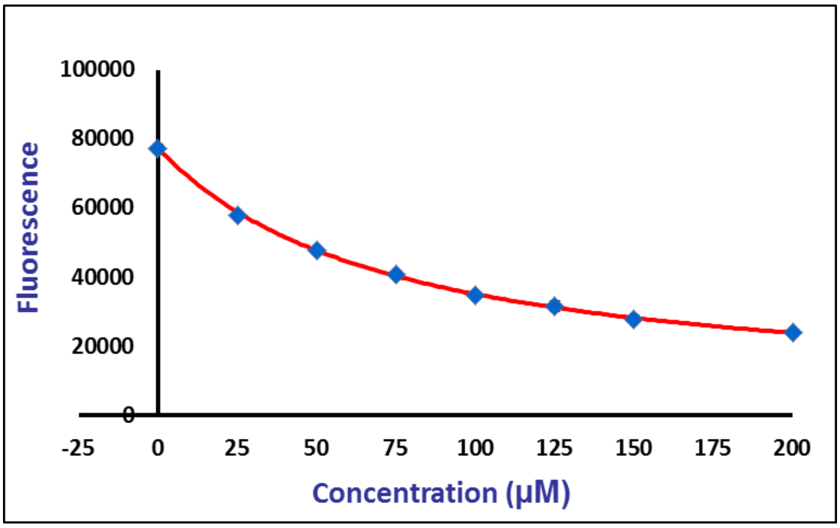

Figure S93: Changes in intrinsic fluorescence intensity of Tdp1 (10 μM) upon the addition of compound 16j(+) (25 μM, 50 μM, 75 μM, 100 μM, 125 μM, 150 μM and 200 μM). Buffer was 20 mM Tris and 250 mM NaCl (pH 8). Excitation wavelength was 280 nm and intrinsic fluorescence was measured at 350 nm. The K_D was 65 ± 7 μM. Experiment were conducted in triplicate.

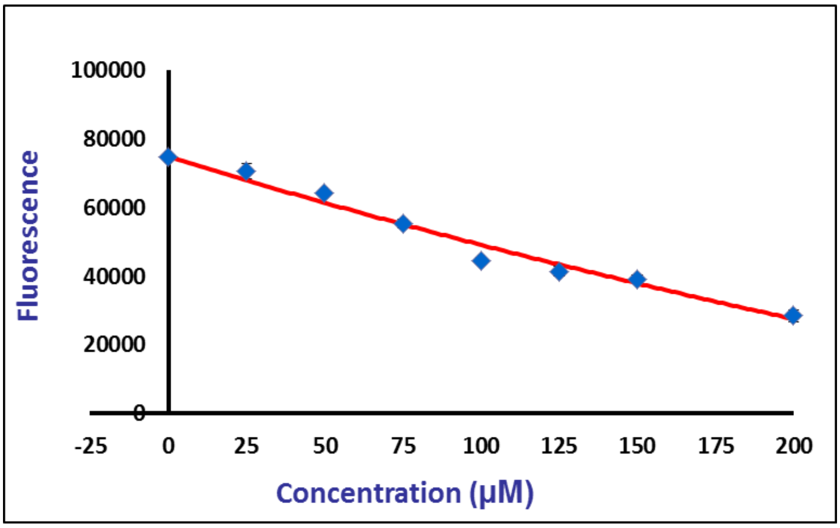

Figure S94: Changes in intrinsic fluorescence intensity of Tdp1 (10 µM) upon the addition of compound 16j(-) (25 µM, 50 µM, 75 µM, 100 µM, 125 µM, 150 µM and 200 µM). Buffer was 20 mM Tris and 250 mM NaCl (pH 8). Excitation wavelength was 280 nm and intrinsic fluorescence was measured at 350 nm. The K_D was 771 ± 46 µM. Experiment were conducted in triplicate.

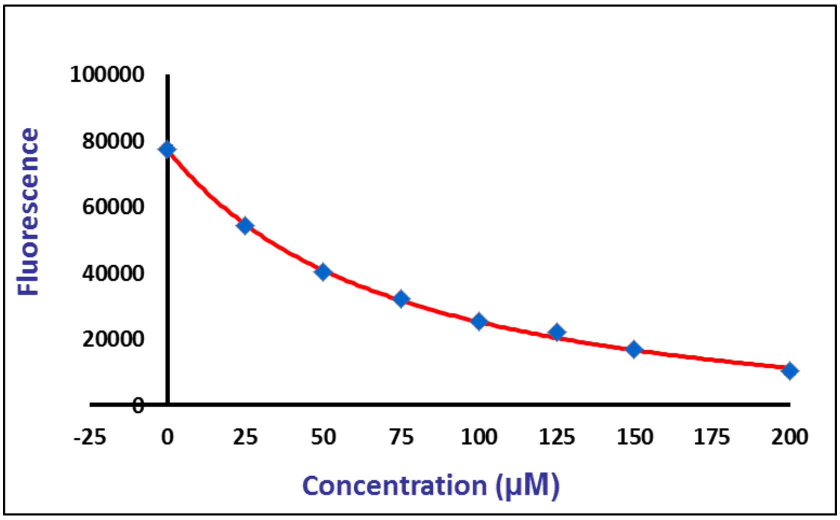

Figure S95: Changes in intrinsic fluorescence intensity of Tdp1 (10 μM) upon the addition of compound 16k(+) (25 μM, 50 μM, 75 μM, 100 μM, 125 μM, 150 μM and 200 μM). Buffer was 20 mM Tris and 250 mM NaCl (pH 8). Excitation wavelength was 280 nm and intrinsic fluorescence was measured at 350 nm. The K_D was 67 ± 9 μM. Experiment were conducted in triplicate.

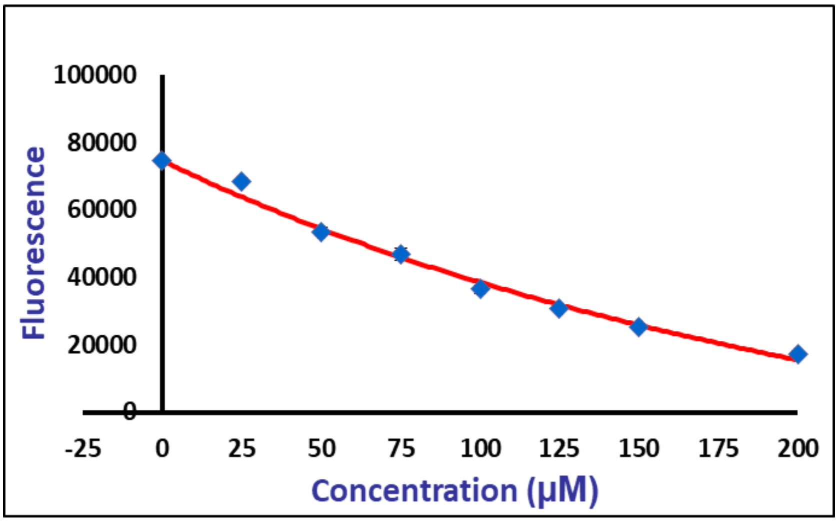

Figure S96: Changes in intrinsic fluorescence intensity of Tdp1 (10 µM) upon the addition of compound 16k(-) (25 µM, 50 µM, 75 µM, 100 µM, 125 µM, 150 µM and 200 µM). Buffer was 20 mM Tris and 250 mM NaCl (pH 8). Excitation wavelength was 280 nm and intrinsic fluorescence was measured at 350 nm. The K_D was 250 ± 24 µM. Experiment were conducted in triplicate.

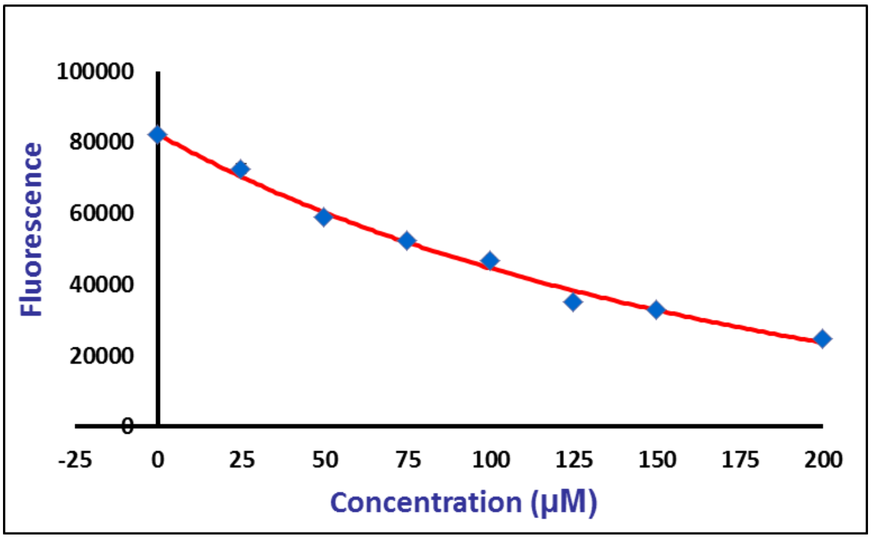

Figure S97: Changes in intrinsic fluorescence intensity of Tdp1 (10 µM) upon the addition of compound 16l(-) (25 µM, 50 µM, 75 µM, 100 µM, 125 µM, 150 µM and 200 µM). Buffer was 20 mM Tris and 250 mM NaCl (pH 8). Excitation wavelength was 280 nm and intrinsic fluorescence was measured at 350 nm. The K_D was 233 ± 9 µM. Experiment were conducted in triplicate.

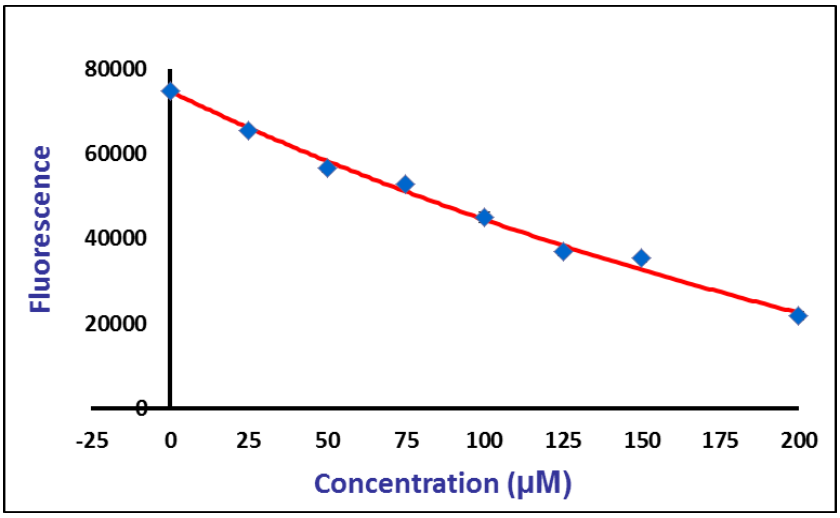

Figure S98: Changes in intrinsic fluorescence intensity of Tdp1 (10 µM) upon the addition of compound 16m(-) (25 µM, 50 µM, 75 µM, 100 µM, 125 µM, 150 µM and 200 µM). Buffer was 20 mM Tris and 250 mM NaCl (pH 8). Excitation wavelength was 280 nm and intrinsic fluorescence was measured at 350 nm. The K_D was 354 ± 40 µM. Experiment were conducted in triplicate.

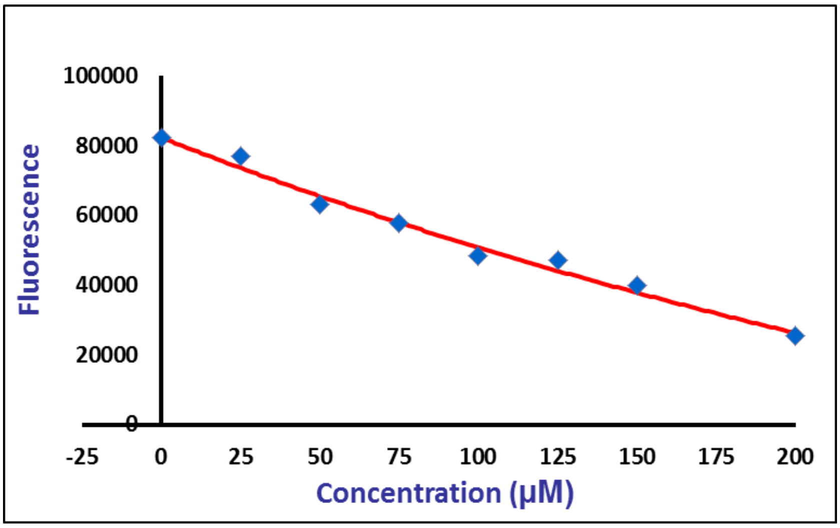

Figure S99: Changes in intrinsic fluorescence intensity of Tdp1 (10 µM) upon the addition of compound 16n(+) (25 µM, 50 µM, 75 µM, 100 µM, 125 µM, 150 µM and 200 µM). Buffer was 20 mM Tris and 250 mM NaCl (pH 8). Excitation wavelength was 280 nm and intrinsic fluorescence was measured at 350 nm. The K_D was 719 ± 17 µM. Experiment were conducted in triplicate.

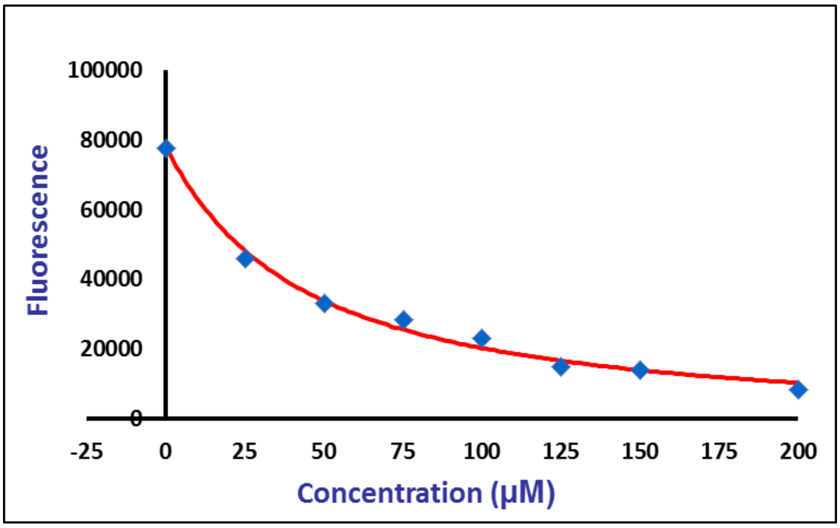

Figure S100: Changes in intrinsic fluorescence intensity of Tdp1 (10 µM) upon the addition of compound 16o(+) (25 µM, 50 µM, 75 µM, 100 µM, 125 µM, 150 µM and 200 µM). Buffer was 20 mM Tris and 250 mM NaCl (pH 8). Excitation wavelength was 280 nm and intrinsic fluorescence was measured at 350 nm. The K_D was 38 ± 2 µM. Experiment were conducted in triplicate.

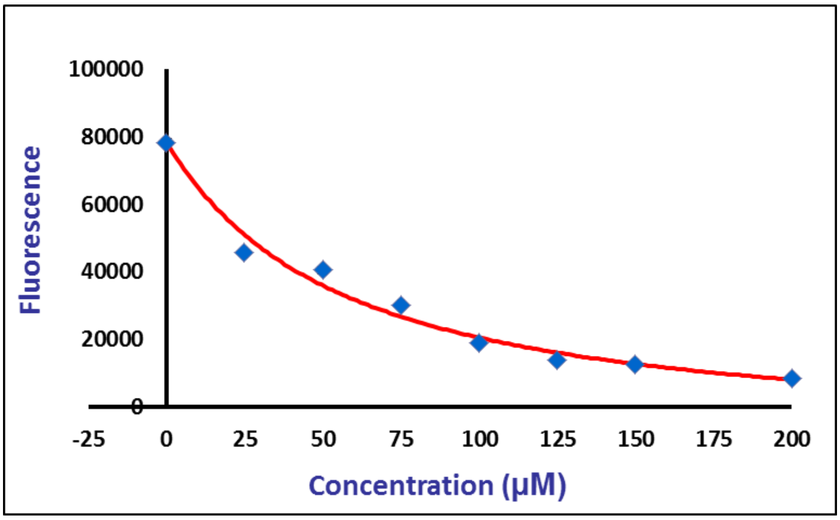

Figure S101: Changes in intrinsic fluorescence intensity of Tdp1 (10 µM) upon the addition of compound 16o(-) (25 µM, 50 µM, 75 µM, 100 µM, 125 µM, 150 µM and 200 µM). Buffer was 20 mM Tris and 250 mM NaCl (pH 8). Excitation wavelength was 280 nm and intrinsic fluorescence was measured at 350 nm. The K_D was 51 ± 4 µM. Experiment were conducted in triplicate.

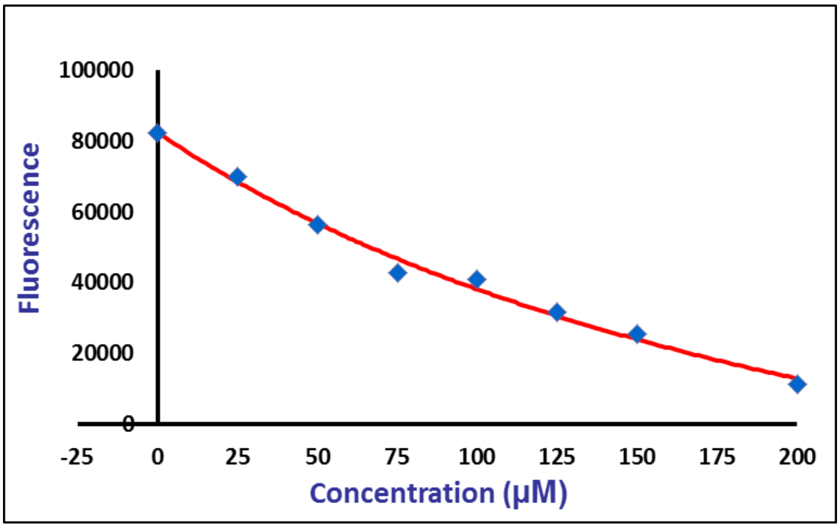

Figure S102: Changes in intrinsic fluorescence intensity of Tdp1 (10 µM) upon the addition of compound 16p(+) (25 µM, 50 µM, 75 µM, 100 µM, 125 µM, 150 µM and 200 µM). Buffer was 20 mM Tris and 250 mM NaCl (pH 8). Excitation wavelength was 280 nm and intrinsic fluorescence was measured at 350 nm. The K_D was 268 ± 11 µM. Experiment were conducted in triplicate.

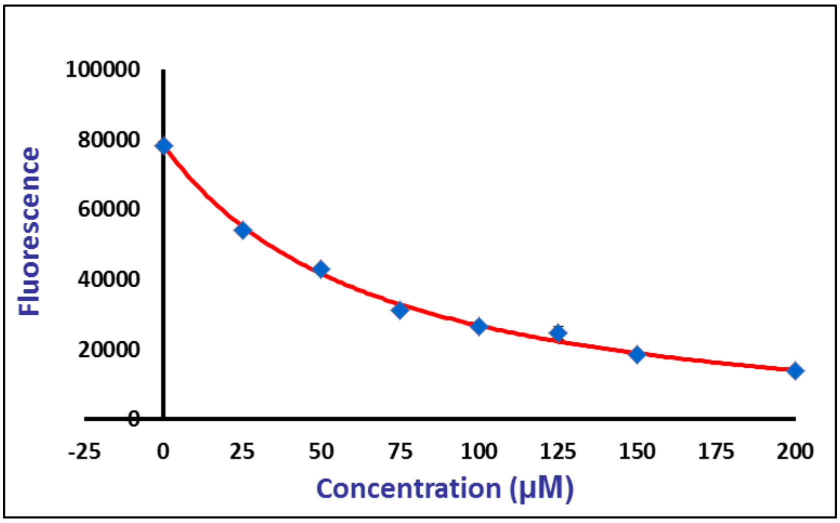

Figure S103: Changes in intrinsic fluorescence intensity of Tdp1 (10 µM) upon the addition of compound 16q(-) (25 µM, 50 µM, 75 µM, 100 µM, 125 µM, 150 µM and 200 µM). Buffer was 20 mM Tris and 250 mM NaCl (pH 8). Excitation wavelength was 280 nm and intrinsic fluorescence was measured at 350 nm. The K_D was 58 ± 4 µM. Experiment were conducted in triplicate.

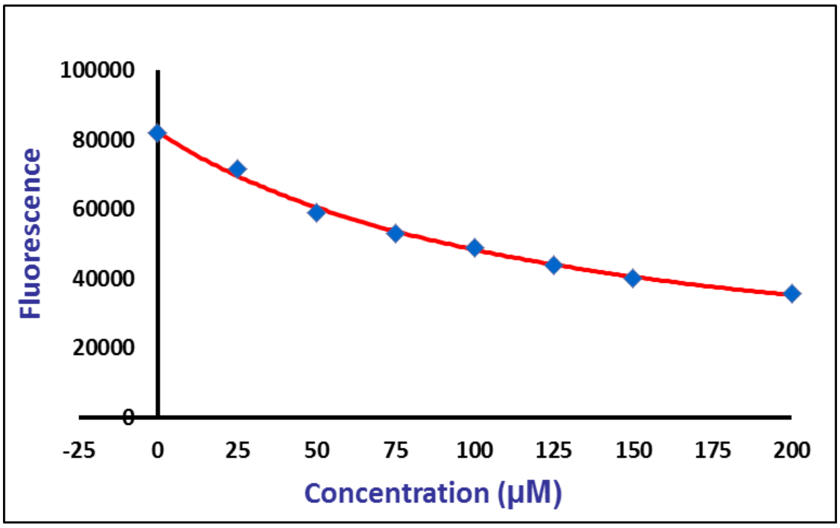

Figure S104: Changes in intrinsic fluorescence intensity of Tdp1 (10 µM) upon the addition of compound 16r(+) (25 µM, 50 µM, 75 µM, 100 µM, 125 µM, 150 µM and 200 µM). Buffer was 20 mM Tris and 250 mM NaCl (pH 8). Excitation wavelength was 280 nm and intrinsic fluorescence was measured at 350 nm. The K_D was 113 ± 52 µM. Experiment were conducted in triplicate.

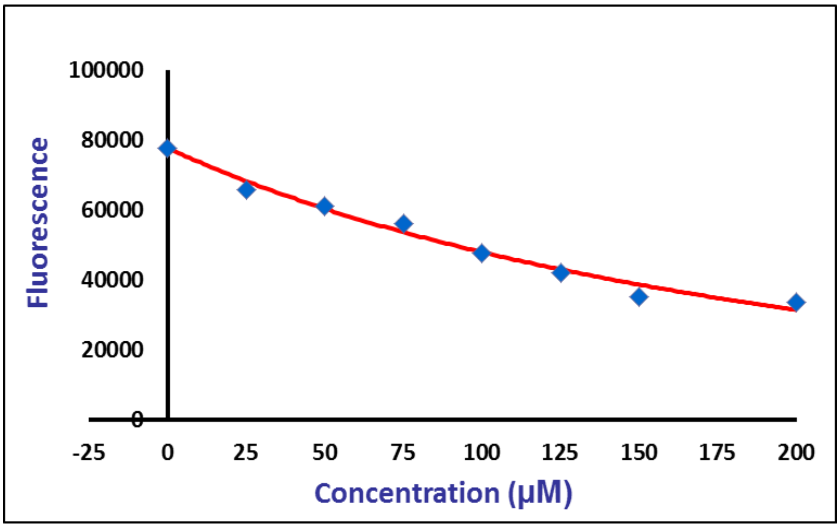

Figure S105: Changes in intrinsic fluorescence intensity of Tdp1 (10 µM) upon the addition of compound 17a(+) (25 µM, 50 µM, 75 µM, 100 µM, 125 µM, 150 µM and 200 µM). Buffer was 20 mM Tris and 250 mM NaCl (pH 8). Excitation wavelength was 280 nm and intrinsic fluorescence was measured at 350 nm. The K_D was 212 ± 25 µM. Experiment were conducted in triplicate.

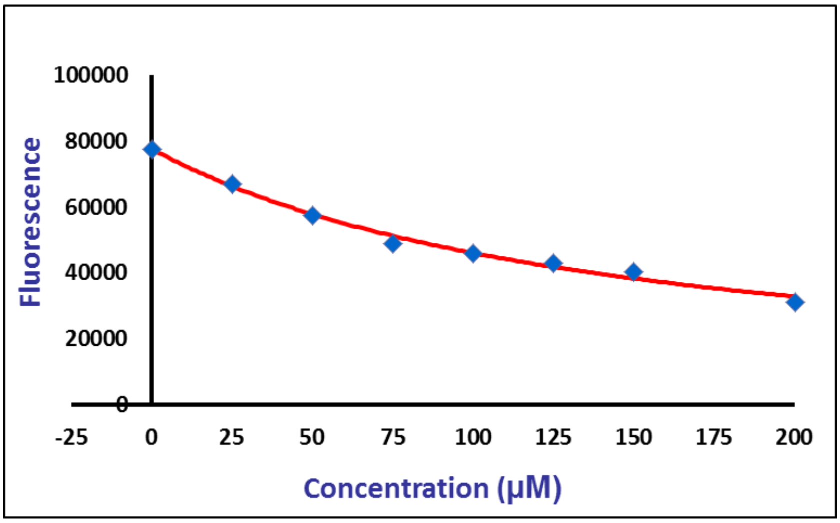

Figure S106: Changes in intrinsic fluorescence intensity of Tdp1 (10 µM) upon the addition of compound 17b(+) (25 µM, 50 µM, 75 µM, 100 µM, 125 µM, 150 µM and 200 µM). Buffer was 20 mM Tris and 250 mM NaCl (pH 8). Excitation wavelength was 280 nm and intrinsic fluorescence was measured at 350 nm. The K_D was 131 ± 15 µM. Experiment were conducted in triplicate.

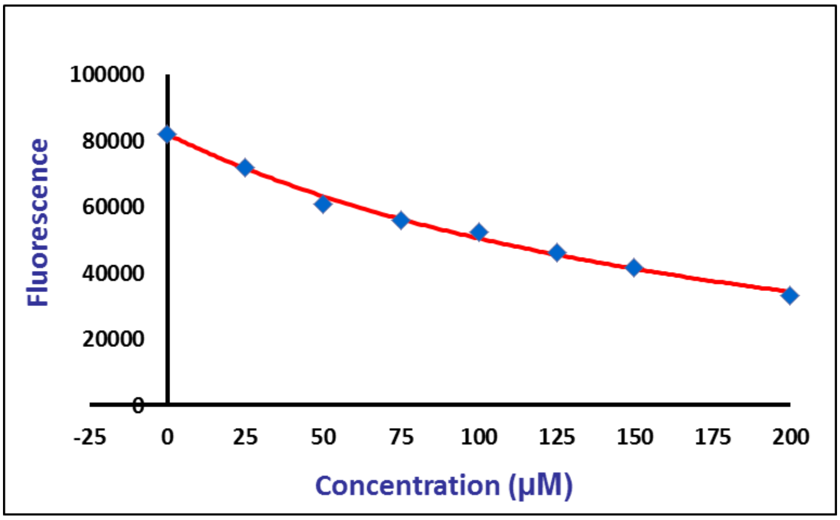

Figure S107: Changes in intrinsic fluorescence intensity of Tdp1 (10 µM) upon the addition of compound 17c(+) (25 µM, 50 µM, 75 µM, 100 µM, 125 µM, 150 µM and 200 µM). Buffer was 20 mM Tris and 250 mM NaCl (pH 8). Excitation wavelength was 280 nm and intrinsic fluorescence was measured at 350 nm. The K_D was 204 ± 23 µM. Experiment were conducted in triplicate.

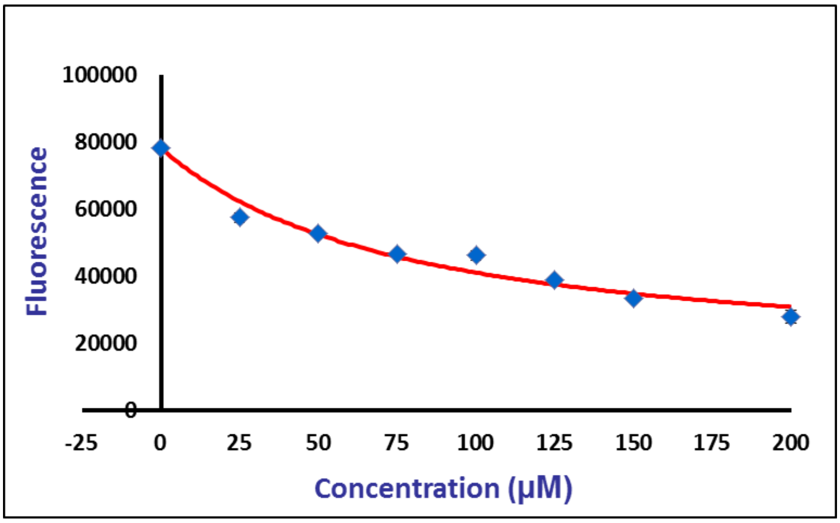

Figure S108: Changes in intrinsic fluorescence intensity of Tdp1 (10 μM) upon the addition of compound 17c(-) (25 μM, 50 μM, 75 μM, 100 μM, 125 μM, 150 μM and 200 μM). Buffer was 20 mM Tris and 250 mM NaCl (pH 8). Excitation wavelength was 280 nm and intrinsic fluorescence was measured at 350 nm. The K_D was 74 ± 4 μM. Experiment were conducted in triplicate.

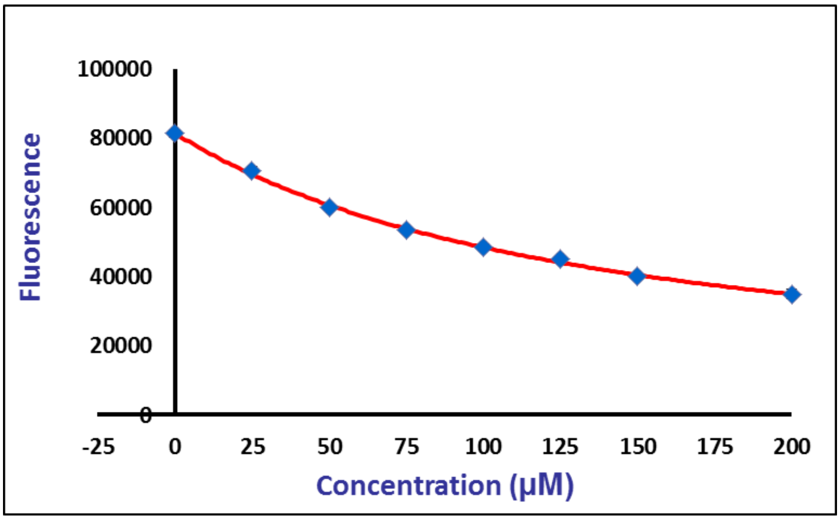

Figure S109: Changes in intrinsic fluorescence intensity of Tdp1 (10 µM) upon the addition of compound 17d(+) (25 µM, 50 µM, 75 µM, 100 µM, 125 µM, 150 µM and 200 µM). Buffer was 20 mM Tris and 250 mM NaCl (pH 8). Excitation wavelength was 280 nm and intrinsic fluorescence was measured at 350 nm. The K_D was 130 ± 29 µM. Experiment were conducted in triplicate.

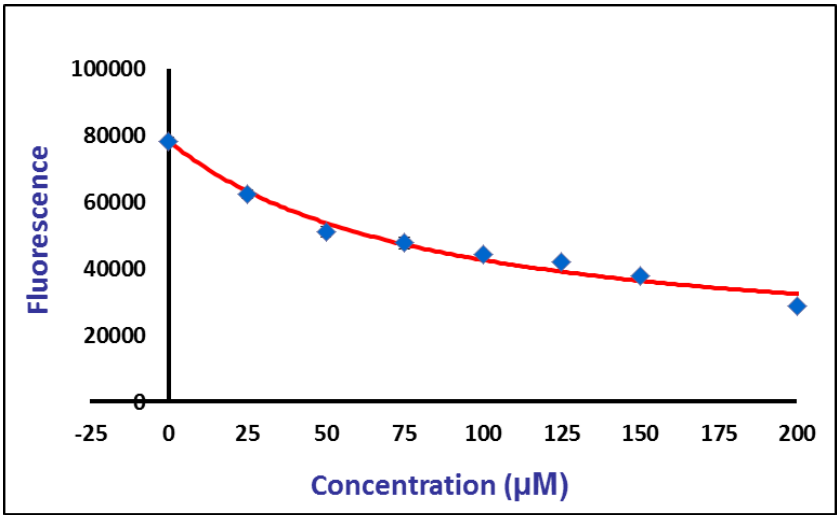

Figure S110: Changes in intrinsic fluorescence intensity of Tdp1 (10 µM) upon the addition of compound 17d(-) (25 µM, 50 µM, 75 µM, 100 µM, 125 µM, 150 µM and 200 µM). Buffer was 20 mM Tris and 250 mM NaCl (pH 8). Excitation wavelength was 280 nm and intrinsic fluorescence was measured at 350 nm. The K_D was 76 ± 5 µM. Experiment were conducted in triplicate.

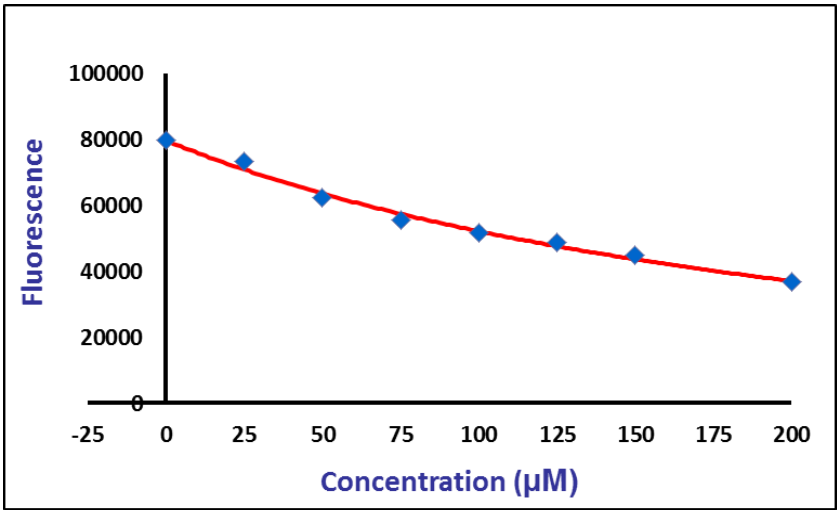

Figure S111: Changes in intrinsic fluorescence intensity of Tdp1 (10 µM) upon the addition of compound 17e(+) (25 µM, 50 µM, 75 µM, 100 µM, 125 µM, 150 µM and 200 µM). Buffer was 20 mM Tris and 250 mM NaCl (pH 8). Excitation wavelength was 280 nm and intrinsic fluorescence was measured at 350 nm. The K_D was 213 ± 4 µM. Experiment were conducted in triplicate.

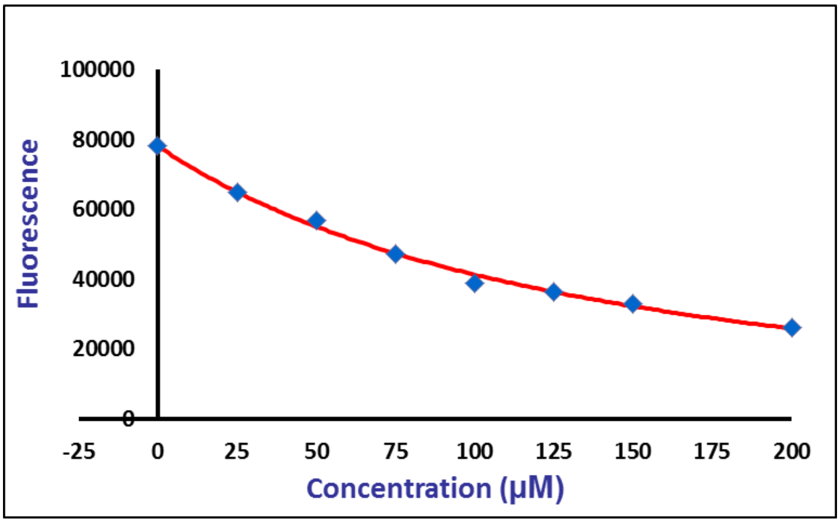

Figure S 112: Changes in intrinsic fluorescence intensity of Tdp1 (10 µM) upon the addition of compound 17e(-) (25 µM, 50 µM, 75 µM, 100 µM, 125 µM, 150 µM and 200 µM). Buffer was 20 mM Tris and 250 mM NaCl (pH 8). Excitation wavelength was 280 nm and intrinsic fluorescence was measured at 350 nm. The K_D was 130 ± 8 µM. Experiment were conducted in triplicate.

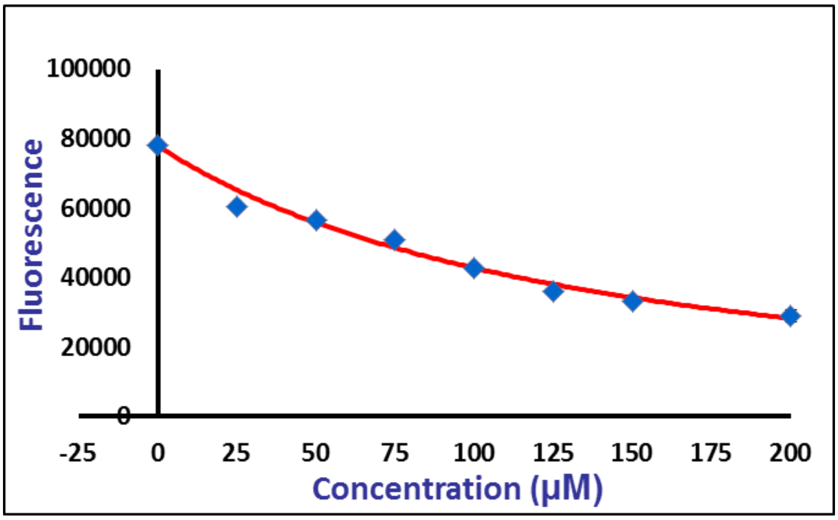

Figure S113: Changes in intrinsic fluorescence intensity of Tdp1 (10 µM) upon the addition of compound 17f(+) (25 µM, 50 µM, 75 µM, 100 µM, 125 µM, 150 µM and 200 µM). Buffer was 20 mM Tris and 250 mM NaCl (pH 8). Excitation wavelength was 280 nm and intrinsic fluorescence was measured at 350 nm. The K_D was 127 ± 14 µM. Experiment were conducted in triplicate.

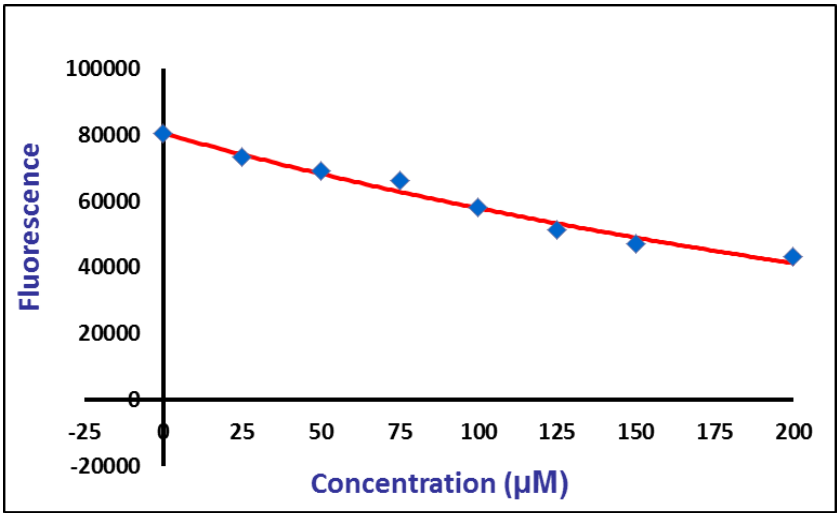

Figure S114: Changes in intrinsic fluorescence intensity of Tdp1 (10 µM) upon the addition of compound 17f(-) (25 µM, 50 µM, 75 µM, 100 µM, 125 µM, 150 µM and 200 µM). Buffer was 20 mM Tris and 250 mM NaCl (pH 8). Excitation wavelength was 280 nm and intrinsic fluorescence was measured at 350 nm. The K_D was 536 ± 90 µM. Experiment were conducted in triplicate.

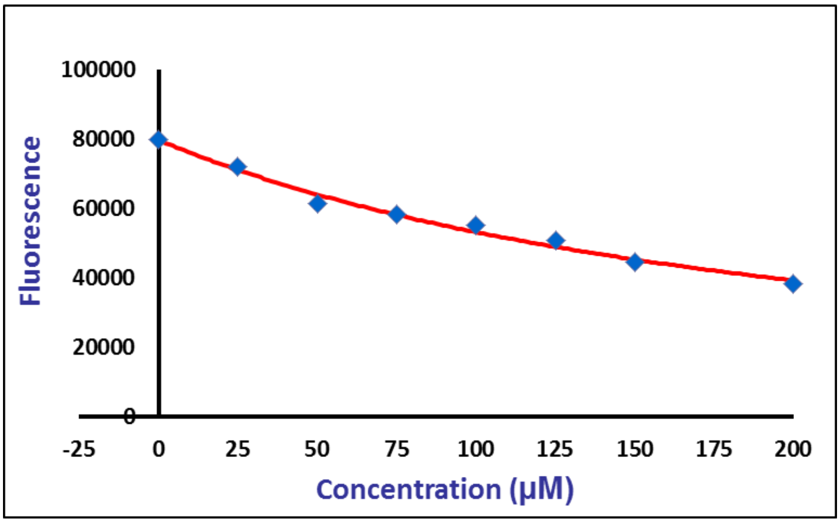

Figure S115: Changes in intrinsic fluorescence intensity of Tdp1 (10 µM) upon the addition of compound 17g(+) (25 µM, 50 µM, 75 µM, 100 µM, 125 µM, 150 µM and 200 µM). Buffer was 20 mM Tris and 250 mM NaCl (pH 8). Excitation wavelength was 280 nm and intrinsic fluorescence was measured at 350 nm. The K_D was 223 ± 42 µM. Experiment were conducted in triplicate.

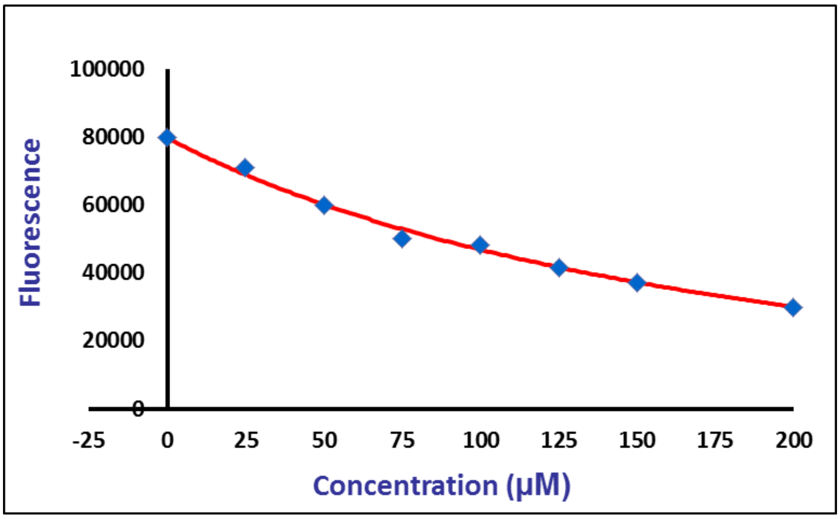

Figure S116: Changes in intrinsic fluorescence intensity of Tdp1 (10 µM) upon the addition of compound 17h(+) (25 µM, 50 µM, 75 µM, 100 µM, 125 µM, 150 µM and 200 µM). Buffer was 20 mM Tris and 250 mM NaCl (pH 8). Excitation wavelength was 280 nm and intrinsic fluorescence was measured at 350 nm. The K_D was 188 ± 22 µM. Experiment were conducted in triplicate.

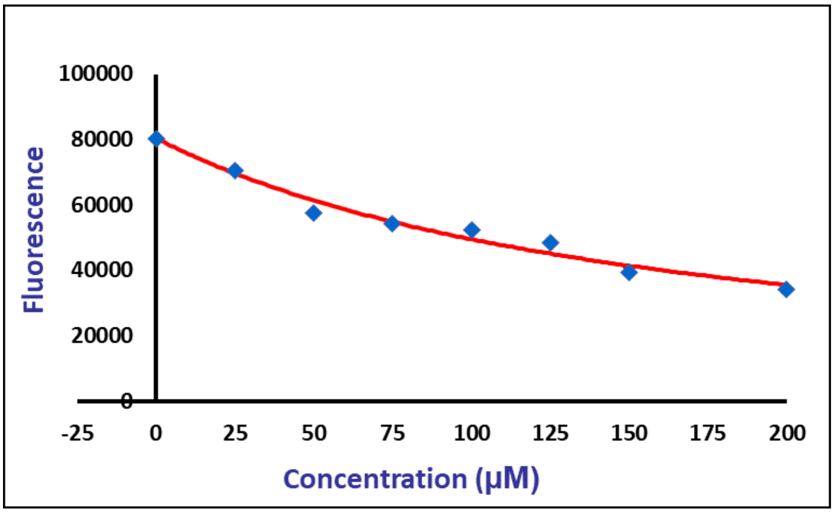

Figure S117: Changes in intrinsic fluorescence intensity of Tdp1 (10 µM) upon the addition of compound 17i(+) (25 µM, 50 µM, 75 µM, 100 µM, 125 µM, 150 µM and 200 µM). Buffer was 20 mM Tris and 250 mM NaCl (pH 8). Excitation wavelength was 280 nm and intrinsic fluorescence was measured at 350 nm. The K_D was 161 ± 22 µM. Experiment were conducted in triplicate.

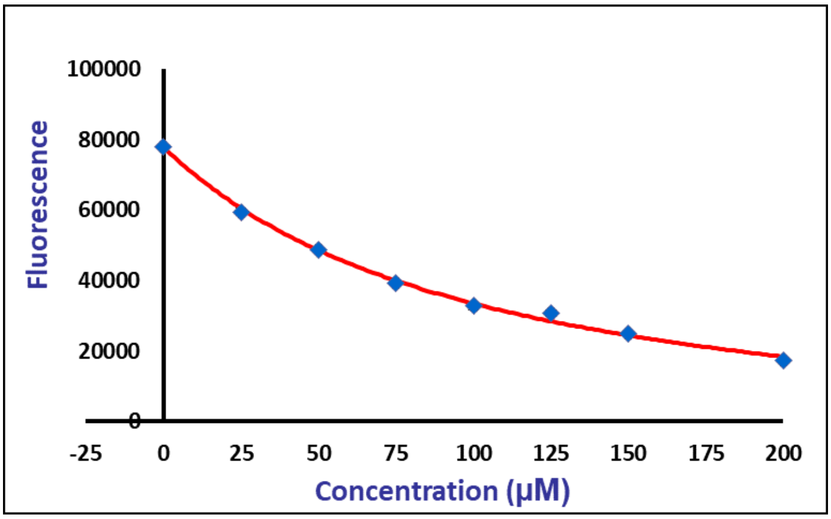

Figure S 118: Changes in intrinsic fluorescence intensity of Tdp1 (10 µM) upon the addition of compound 17j(+) (25 µM, 50 µM, 75 µM, 100 µM, 125 µM, 150 µM and 200 µM). Buffer was 20 mM Tris and 250 mM NaCl (pH 8). Excitation wavelength was 280 nm and intrinsic fluorescence was measured at 350 nm. The K_D was 69 ± 14 µM. Experiment were conducted in triplicate.

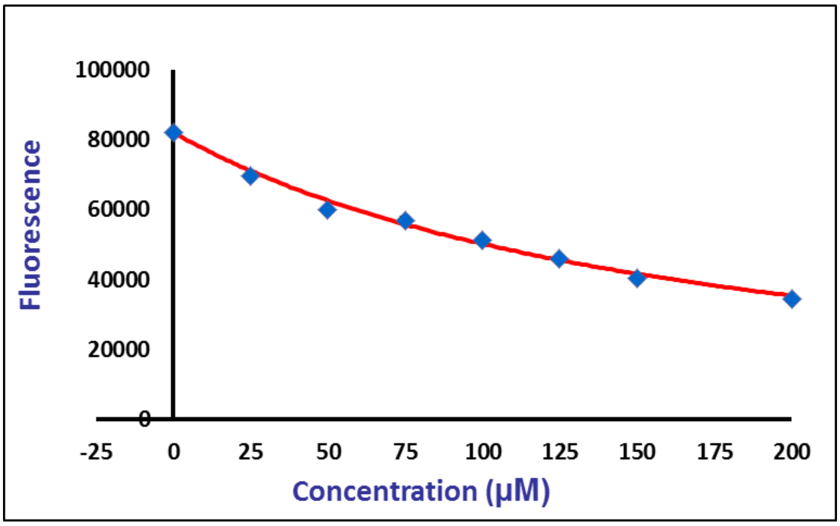

Figure S 119: Changes in intrinsic fluorescence intensity of Tdp1 (10 µM) upon the addition of compound 17k(+) (25 µM, 50 µM, 75 µM, 100 µM, 125 µM, 150 µM and 200 µM). Buffer was 20 mM Tris and 250 mM NaCl (pH 8). Excitation wavelength was 280 nm and intrinsic fluorescence was measured at 350 nm. The K_D was 156 ± 8 µM. Experiment were conducted in triplicate.

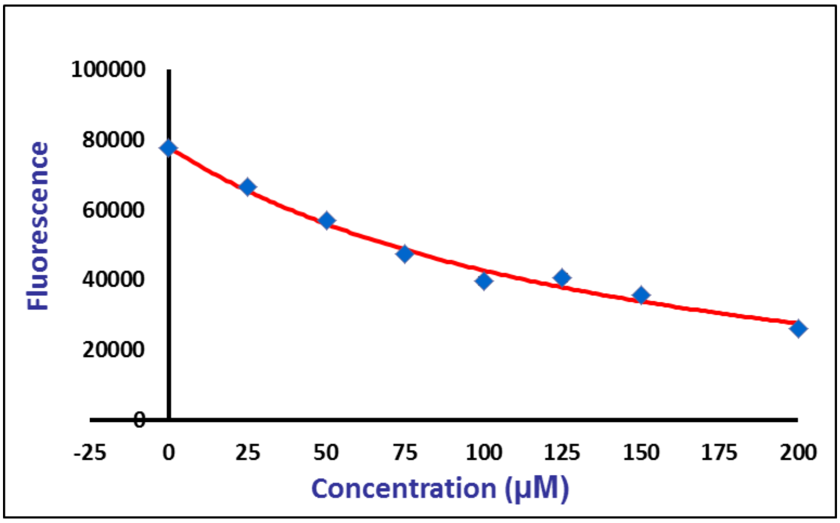

Figure S120: Changes in intrinsic fluorescence intensity of Tdp1 (10 µM) upon the addition of compound 17k(-) (25 µM, 50 µM, 75 µM, 100 µM, 125 µM, 150 µM and 200 µM). Buffer was 20 mM Tris and 250 mM NaCl (pH 8). Excitation wavelength was 280 nm and intrinsic fluorescence was measured at 350 nm. The K_D was 142 ± 11 µM. Experiment were conducted in triplicate.

Table S17: RMSD values of for heavy atoms between the co-crystallized benzene-1,2,4-tricarboxylic acid (6DIE) and the docked molecule.

	ASP		CHEMPLP		CHEMSCORE		GOLDSCORE	
POSE	WATER	NO WATER	WATER	NO WATER	WATER	NO WATER	WATER	NO WATER
1	2.890	2.912	2.748	4.825	0.568	8.145	9.505	4.019
2	2.869	2.906	2.956	4.774	0.631	2.773	9.486	3.922
3	2.929	2.785	2.752	3.668	0.639	2.751	9.797	4.132
Average	**2.896**	**2.868**	**2.818**	**4.422**	**0.613**	**4.557**	**9.596**	**4.024**

Table S18: Scores of the scoring function from the docked ligands with and without water.

	ASP		CHEMPLP		CHEMSCORE		GOLDSCORE	
COMPOUND	WATER	NO WATER	WATER	NO WATER	WATER	NO WATER	WATER	NO WATER
(+)-16a	38.9	36.1	69.7	64.5	28.9	27.9	66.6	69.0
(-)-16a	38.0	33.2	66.3	54.5	29.3	23.7	71.1	69.5
(+)-16b	39.4	33.1	66.9	57.9	30.0	28.6	67.7	66.5
(-)-16b	33.2	33.8	70.9	61.0	27.7	23.1	74.7	67.9
(+)-16c	38.8	34.0	64.8	55.4	29.2	28.7	66.0	66.6
(-)-16c	37.2	33.1	73.2	63.3	30.5	23.1	67.6	70.1
(+)-16d	40.4	37.6	63.7	63.7	30.8	26.2	72.8	68.6
(-)-16d	41.0	38.7	71.3	68.7	29.1	28.6	81.3	59.9
(+)-16e	38.7	34.4	65.6	67.7	31.4	26.9	73.9	68.6
(-)-16e	38.8	35.5	71.6	65.6	31.2	29.2	78.4	66.9
(+)-16f	38.4	32.8	65.7	62.5	31.7	28.2	78.7	67.8
(-)-16f	38.6	34.8	73.0	67.4	30.9	28.3	79.4	72.5
(+)-16g	38.1	32.9	69.5	61.6	29.8	26.3	71.5	67.0
(-)-16g	39.1	34.6	71.9	67.8	31.6	29.0	77.3	60.3
(+)-16h	37.6	33.0	68.9	64.7	32.0	28.4	71.8	70.5
(-)-16h	39.7	36.3	70.5	75.9	31.8	27.0	78.6	61.6
(+)-16i	38.2	33.1	65.5	62.5	31.2	28.0	72.8	68.7
(-)-16i	38.4	35.5	70.8	66.8	32.2	28.4	75.3	65.5
(+)-16j	37.9	31.3	68.4	60.2	28.3	25.1	68.8	65.3
(-)-16j	35.4	35.2	65.4	68.9	30.3	29.0	74.5	63.0
(+)-16k	39.7	32.4	64.7	56.7	31.1	26.6	69.4	65.0
(-)-16k	38.6	34.8	68.2	65.4	30.5	27.8	74.2	59.7
(+)-16l	41.0	33.3	64.4	60.2	30.4	24.7	66.1	63.0
(-)-16l	38.7	36.0	67.8	63.4	28.6	26.3	73.7	61.0
(+)-16m	38.0	32.5	66.7	63.2	31.9	26.8	66.6	63.6
(-)-16m	39.5	35.4	70.7	65.9	30.9	29.6	75.4	59.2

(+)-16n	40.4	33.0	72.2	63.5	29.2	26.6	71.3	63.5
(-)-16n	38.3	35.4	74.0	67.6	31.4	27.1	74.9	65.5
(+)-16o	42.7	37.1	64.3	56.4	29.2	23.2	67.6	64.6
(-)-16o	40.7	39.3	66.0	60.0	30.5	28.5	75.2	57.8
(+)-16p	42.7	37.0	75.9	71.4	34.1	29.2	76.3	74.1
(-)-16p	41.7	38.9	79.1	79.2	28.8	32.5	79.9	59.0
(+)-16q	43.1	35.8	67.4	68.5	30.4	28.9	70.9	77.6
(-)-16q	41.8	38.6	74.8	75.7	31.2	32.3	84.1	74.8
(+)-16r	42.2	35.6	76.7	68.7	33.0	28.2	89.9	79.3
(-)-16r	44.8	39.6	76.1	81.7	33.7	31.5	89.2	85.5
(+)-17a	46.7	40.2	82.8	79.2	33.7	24.5	79.4	79.7
(-)-17a	49.0	40.2	81.2	81.4	30.5	35.8	90.0	78.0
(+)-17b	47.9	38.2	88.7	75.1	31.2	26.9	82.6	74.0
(-)-17b	46.9	41.9	81.1	82.6	29.2	35.1	92.1	76.5
(+)-17c	47.1	38.1	82.7	78.5	29.2	28.2	78.0	81.0
(-)-17c	46.9	42.7	79.4	79.8	30.8	30.7	91.8	84.9
(+)-17d	47.7	38.4	84.2	76.9	31.8	28.1	88.5	75.1
(-)-17d	47.5	43.7	85.9	83.5	29.2	36.0	94.7	78.1
(+)-17e	47.4	40.9	82.8	80.4	28.5	26.5	81.1	75.6
(-)-17e	51.4	40.9	82.5	83.0	28.9	31.8	87.4	70.8
(+)-17f	48.3	39.2	78.1	76.5	33.2	26.7	90.4	75.5
(-)-17f	46.9	42.3	79.9	81.3	30.9	31.2	96.0	73.8
(+)-17g	46.7	35.8	85.1	81.7	33.4	30.2	88.7	78.3
(-)-17g	48.4	41.6	85.6	80.0	32.1	35.0	93.4	95.8
(+)-17h	46.3	37.3	85.5	79.2	33.5	30.2	78.5	80.6
(-)-17h	48.3	39.1	85.1	78.5	34.4	32.3	90.1	89.2
(+)-17i	49.2	41.8	93.3	83.8	30.9	31.5	81.1	78.4
(-)-17i	49.9	39.0	84.1	74.3	31.6	31.3	83.4	78.1
(+)-17j	53.6	40.4	88.4	86.7	33.2	28.3	83.0	82.4
(+)-17k	47.6	43.1	78.7	73.9	30.4	24.9	76.9	84.9
(-)-17j	47.7	38.0	83.8	80.8	30.9	28.4	94.8	87.1
(-)-17k	48.5	41.4	83.5	77.3	27.9	30.5	77.5	80.1

Table S19: The calculated molecular descriptors for the ligands.

Compound	MW	HB Donor	HB Acceptor	Log*P*	PSA	Rot. bonds
(+)-16a	504.5	3	11.3	2.0	147.5	8
(-)-16a	504.5	3	11.3	2.0	146.0	8
(+)-16b	504.5	3	10.8	2.4	146.7	8
(-)-16b	504.5	3	10.8	2.3	145.0	8
(+)-16c	504.5	3	11.3	2.0	147.5	8
(-)-16c	504.5	3	11.3	2.0	145.9	8
(+)-16d	554.5	3	10.8	2.1	181.6	9
(-)-16d	554.5	3	10.8	2.0	180.4	9
(+)-16e	523.6	3	9.8	3.0	136.2	8
(-)-16e	523.6	3	9.8	3.0	134.5	8
(+)-16f	588.4	3	9.8	3.3	136.2	8
(-)-16f	588.4	3	9.8	3.2	134.4	8
(+)-16g	509.6	3	9.8	2.7	136.3	8
(-)-16g	509.6	3	9.8	2.7	134.6	8
(+)-16h	588.5	3	9.8	3.3	136.2	8
(-)-16h	588.4	3	9.8	3.2	134.6	8
(+)-16i	523.6	3	9.8	3.0	136.2	8
(-)-16i	523.6	3	9.8	2.9	134.5	8
(+)-16j	509.6	3	9.8	2.7	135.7	8
(-)-16j	509.6	3	9.8	3.0	132.5	8
(+)-16k	493.5	3	10.3	2.4	144.1	8
(-)-16k	493.5	3	10.3	2.4	142.5	8
(+)-16l	493.5	3	10.3	2.2	143.5	8
(-)-16l	493.5	3	10.3	2.2	141.9	8
(+)-16m	492.5	4	9.8	2.4	148.2	8
(-)-16m	492.5	4	9.8	2.4	146.7	8
(+)-16n	506.5	3	9.8	3.1	135.8	8
(-)-16n	506.5	3	9.8	3.2	134.4	8
(+)-16o	493.5	4	11.3	1.5	162.2	8
(-)-16o	493.5	4	11.3	1.5	160.7	8
(+)-16p	542.6	4	9.8	2.9	147.3	8
(-)-16p	542.6	4	9.8	2.8	145.9	8
(+)-16q	556.6	4	9.8	3.2	146.7	8
(-)-16q	556.6	4	9.8	3.2	145.4	8
(+)-16r	604.0	3	10.3	3.8	138.0	8
(-)-16r	604.0	3	10.3	4.7	151.2	8
(+)-17a	657.7	3	11.3	4.4	140.9	12
(-)-17a	657.7	3	11.3	4.5	140.9	12
(+)-17b	657.7	3	11.3	4.4	141.3	12
(-)-17b	657.7	3	11.3	4.5	141.0	12
(+)-17c	657.7	3	11.3	4.3	141.7	12

(-)-17c	657.7	3	11.3	4.5	140.7	12
(+)-17d	692.1	3	11.3	4.7	140.9	12
(-)-17d	692.1	3	11.3	5.0	140.4	12
(+)-17e	675.7	3	11.3	4.5	141.7	12
(-)-17e	675.7	3	11.3	4.7	140.7	12
(+)-17f	692.1	3	11.3	4.7	142.5	12
(-)-17f	692.1	3	11.3	4.8	141.2	12
(+)-17g	725.9	3	11.0	5.6	134.3	13
(-)-17g	725.9	3	11.0	7.1	152.0	13
(+)-17h	688.2	3	11.3	4.8	140.6	12
(-)-17h	688.2	3	11.3	6.0	160.4	12
(+)-17i	740.8	4	14.0	3.7	151.3	12
(-)-17i	725.8	3	13.5	5.4	160.5	11
(+)-17j	752.8	4	14.8	3.6	156.6	13
(-)-17j	737.8	3	14.3	5.2	168.7	12
(+)-17k	701.7	4	15.0	2.1	189.2	13
(-)-17k	701.7	4	15.0	2.5	198.4	13

Table S20: Definition of lead-like, drug-like and Known drug space (KDS) in terms of molecular descriptors. The values given are the maxima for each descriptor for the volumes of chemical space used.

	**Lead-like Space**	**Drug-like Space**	**Known Drug Space**
Molecular weight (g mol^{-1})	300	500	800
Lipophilicity (Log P)	3	5	6.5
Hydrogen bond donors (HD)	3	5	7
Hydrogen bond acceptors (HA)	3	10	15
Polar surface area ($Å^2$) (PSA)	60	140	180
Rotatable bonds (RB)	3	10	17

Table S21: Known drug index calculated

**Compound**	KDI-2A	KDI-2B
(+)-16a	4.41	0.12
(-)-16a	4.41	0.12
(+)-16b	4.52	0.15
(-)-16b	4.42	0.13
(+)-16c	5.50	0.57
(-)-16c	4.51	0.15
(+)-16d	4.42	0.13
(-)-16d	4.48	0.16
(+)-16e	4.67	0.20
(-)-16e	4.47	0.16
(+)-16f	4.73	0.22
(-)-16f	4.64	0.19
(+)-16g	4.42	0.13
(-)-16g	4.44	0.13
(+)-16h	4.63	0.19
(-)-16h	4.65	0.19
(+)-16i	3.41	0.01
(-)-16i	4.73	0.22
(+)-16j	3.92	0.06
(-)-16j	4.44	0.13
(+)-16k	4.42	0.13
(-)-16k	4.68	0.21
(-)-16m	4.75	0.23
(+)-16m	3.92	0.06
(+)-16l	4.67	0.20
(-)-16l	4.68	0.21
(+)-16n	4.65	0.19
(-)-16n	4.25	0.11
(+)-16o	4.29	0.12
(-)-16o	4.74	0.23
(+)-16p	4.24	0.11
(-)-16p	2.40	0.00
(+)-16q	4.73	0.22
(-)-16q	4.31	0.12
(+)-16r	3.29	0.01
(-)-16r	2.91	0.01
(+)-17a	4.06	0.08
(-)-17a	3.28	0.01
(+)-17b	3.41	0.01
(-)-17b	2.62	0.00
(+)-17c	2.43	0.00
(-)-17c	4.07	0.08
(+)-17d	3.42	0.01
(-)-17d	3.40	0.01
(+)-17e	3.09	0.00
(-)-17e	3.25	0.01
(+)-17f	3.30	0.01
(-)-17f	3.33	0.01
(+)-17g	3.18	0.01

(-)-17g	3.39	0.01
(+)-17h	3.35	0.01
(-)-17h	2.87	0.00
(+)-17i	3.40	0.01
(-)-17i	2.67	0.00
(+)-17j	3.28	0.01
(-)-17j	4.03	0.07
(+)-17k	4.25	0.09
(-)-17k	4.72	0.22

RMSD of other crystal structures of Tdp1 (docked without water)

1. 6dim

Pose	ASP	ChemPLP	CS	GS
1	0.623	2.799	1.624	5.881
2	0.832	2.710	2.184	5.846
3	1.910	2.835	1.717	5.986
Average	1.122	2.781	1.842	5.904

2. 6djd

Pose	ASP	ChemPLP	CS	GS
1	2.792	2.839	2.788	5.969
2	2.800	2.816	2.862	5.923
3	2.593	2.739	2.949	6.407
Average	2.728	2.798	2.867	6.100

3. 6dje

Pose	ASP	ChemPLP	CS	GS
1	1.878	0.920	2.508	5.469
2	2.011	0.508	1.686	5.488
3	2.326	0.757	1.713	5.831
Average	2.071	0.728	1.969	5.596

4.6djf

Pose	ASP	ChemPLP	CS	GS
1	1.800	5.279	5.167	5.830
2	1.702	5.216	5.191	5.792
3	2.070	5.260	5.273	5.828
Average	1.857	5.252	5.210	5.817

5. 6djg

Pose	ASP	ChemPLP	CS	GS
1	2.037	5.187	1.891	5.892
2	2.178	5.198	1.881	5.824

3	1.917	5.209	2.266	5.837
Average	2.044	5.198	2.012	5.851

6. 6djh

Pose	ASP	ChemPLP	CS	GS
1	1.622	0.800	5.400	5.680
2	1.999	0.796	5.281	5.633
3	1.949	0.857	5.272	5.527
Average	1.857	0.818	5.318	5.614

7. 6dji

Pose	ASP	ChemPLP	CS	GS
1	3.563	3.547	5.185	2.860
2	3.551	3.533	5.164	2.705
3	3.607	3.603	5.205	2.627
Average	3.574	3.561	5.184	2.730

8. 6mj5

Pose	ASP	ChemPLP	CS	GS
1	1.868	0.862	1.781	5.789
2	1.901	0.782	1.917	5.861
3	1.957	0.794	1.571	5.857
Average	1.908	0.813	1.756	5.836

9. 6n17

Pose	ASP	ChemPLP	CS	GS
1	12.490	13.368	12.501	11.456
2	12.192	13.356	12.329	11.535
3	12.498	12.616	12.432	11.886
Average	12.393	13.113	12.421	11.626

10. 6n19

Pose	ASP	ChemPLP	CS	GS
1	8.577	2.595	2.340	3.821
2	2.385	2.472	2.448	4.010
3	7.805	1.610	2.137	4.011
Average	6.256	2.226	2.309	3.947
